# Supplementary material for: Proteomic Analysis of Human Immune Responses to Live-Attenuated Tularemia Vaccine
Source: Vaccines (Basel). 2020 Jul 24;8(3):413. doi: 10.3390/vaccines8030413 (PMC7564149; doi:10.3390/vaccines8030413)
Supplement: Supplementary file 1 [file vaccines-08-00413-s001.pdf]

Supplementary Text Manuscript Appendix for "2D-DIGE and LC/MS/MS analysis of immune responses to live-attenuated tularemia vaccine"

May 7, 2020

Table of Contents

1 Introduction **10**

2 Supplemental Methods **10**

2.1 Proteomics experiments 10

2.1.1 Protein spike-in controls 10

2.1.2 Shared protein sequence database and protein families 10

2.1.3 LC-MS/MS proteomics experiment and data processing 10

2.1.4 2D-DIGE/MS proteomics experiment and data processing 11

2.2 Statistical analysis 12

2.2.1 Data normalization 12

2.2.2 Missing value imputation and log fold change from baseline calculation 12

2.2.3 Identification of differentially abundant proteins 13

2.2.4 Pathway enrichment analysis 13

2.3 Comparison of shared proteins 13

2.4 Statistical analysis plan deviations 14

2.5 Software 14

3 Supplemental Results **14**

3.1 Data normalization and quality control 14

3.1.1 Summary statistics 14

3.1.2 Data normalization 14

3.1.3 Comparison of spike-in control measurements 15

3.1.4 Missing observations 16

3.1.5 Global protein abundance patterns and outlying samples 16

3.2 Identification and comparison of differentially abundant proteins 16

3.3 Determination of higher order organization of differentially abundant proteins 16

3.4 Comparison of shared proteins 17

4 References **17**

List of Figures

Figure S1 Representative 2D-DIGE gel images. 19
Figure S2 Boxplots of proteomics sample metrics (LC-MS/MS) 19
Figure S3 Boxplots of proteomics sample metrics (2D-DIGE/MS) 20
Figure S4 Starplots of proteomics sample metrics (LC-MS/MS) 21
Figure S5 Boxplots of log_2 LFQ intensity before median normalization (LC-MS/MS) 22
Figure S6 Boxplots of log_2 LFQ intensity after median normalization (LC-MS/MS) 23
Figure S7 Boxplots of log_2 spot volume ratios before LOESS normalization (2D-DIGE/MS) 24
Figure S8 Boxplots of log_2 spot volume ratios after LOESS normalization (2D-DIGE/MS) 25
Figure S9 ECDF plots of log_2 LFQ intensity before and after median normalization (LC-MS/MS) . . 26
Figure S10 ECDF plots of log_2 spot volume ratios before and after LOESS normalization (2D-DIGE/MS) 27
Figure S11 MA plots of Cy5 versus Cy3 before and after LOESS normalization (2D-DIGE/MS) 28
Figure S12 MA plots of Cy5 versus Cy3 before and after LOESS normalization (2D-DIGE/MS) 29
Figure S13 MA plots of Cy5 versus Cy3 before and after LOESS normalization (2D-DIGE/MS) 30
Figure S14 Boxplots of log_2 spike-in control protein signals and variability metrics (LC-MS/MS and 2D-DIGE/MS). 31
Figure S15 ECDF plots of log_2 spike-in control protein signals (LC-MS/MS and 2D-DIGE/MS). 32
Figure S16 ECDF plots of missing protein observations across samples (LC-MS/MS and 2D-DIGE/MS, n=30) 33
Figure S17 Scatterplots of missing observations by average log_2 protein signal (LC-MS/MS and 2D-DIGE/MS) 34
Figure S18 PCA and non-metric multidimensional scaling biplots (LC-MS/MS) 35
Figure S19 PCA and non-metric multidimensional scaling biplots (2D-DIGE/MS) 36
Figure S20 Proteomics-based Hierarchical Clustering Plots (LC-MS/MS) 37
Figure S21 Proteomics-based Hierarchical Clustering Plots (2D-DIGE/MS) 38
Figure S22 Venn diagrams summarizing overlap in DA proteins/gel spots between post-vaccination days (LC-MS/MS and 2D-DIGE/MS. 39
Figure S23 Heatmap of protein log_2 fold change from pre-vaccination (LC-MS/MS, , Day 7). 40
Figure S24 Heatmap of protein log_2 fold change from pre-vaccination (LC-MS/MS, , Day 14). 41
Figure S25 Heatmap of protein log_2 fold change from pre-vaccination (2D-DIGE/MS, , Day 7). 42
Figure S26 Heatmap of protein log_2 fold change from pre-vaccination (2D-DIGE/MS, , Day 14). . . . 43
Figure S27 Venn diagrams summarizing overlap in DA proteins among 35 proteins with shared identifications for both laboratories (LC-MS/MS and 2D-DIGE/MS). 44
Figure S28 Heatmap of protein log_2 fold change for shared DA proteins (LC-MS/MS and 2D-DIGE/MS, Day 7). 45
Figure S29 Heatmap of protein log_2 fold change for shared DA proteins (LC-MS/MS and 2D-DIGE/MS, Day 14). 46
Figure S30 Pathway map - Ribosome - Homo sapiens (human) (LC-MS/MS, Day 7) 47
Figure S31 Pathway map - Ribosome - Homo sapiens (human) (LC-MS/MS, Day 14) 48
Figure S32 Pathway map - Proteasome - Homo sapiens (human) (LC-MS/MS, Day 14) 49
Figure S33 Pathway map - Protein processing in endoplasmic reticulum - Homo sapiens (human) (2D-DIGE/MS, Day 14) 50

Figure S34 Pathway map - Phagosome - Homo sapiens (human) (2D-DIGE/MS, Day 14) 51
Figure S35 Pathway map - Antigen processing and presentation - Homo sapiens (human) (2D-DIGE/MS, Day 7) 52
Figure S36 Pathway map - Antigen processing and presentation - Homo sapiens (human) (2D-DIGE/MS, Day 14) 53
Figure S37 Pathway map - Estrogen signaling pathway - Homo sapiens (human) (2D-DIGE/MS, Day 7) 54
Figure S38 Pathway map - Estrogen signaling pathway - Homo sapiens (human) (2D-DIGE/MS, Day 14) 55
Figure S39 Pathway map - Pathogenic Escherichia coli infection - Homo sapiens (human) (2D-DIGE/MS, Day 7) 56
Figure S40 Pathway map - Pathogenic Escherichia coli infection - Homo sapiens (human) (2D-DIGE/MS, Day 14) 57
Figure S41 Pathway map - Legionellosis - Homo sapiens (human) (2D-DIGE/MS, Day 14) 58
Figure S42 Pathway map - Ribosome - Homo sapiens (human) (RNA-Seq, Saint Louis University, Day 1) 59
Figure S43 Pathway map - Ribosome - Homo sapiens (human) (RNA-Seq, Saint Louis University, Day 2) 60
Figure S44 Pathway map - Ribosome - Homo sapiens (human) (RNA-Seq, Saint Louis University, Day 7) 61
Figure S45 Pathway map - Ribosome - Homo sapiens (human) (RNA-Seq, Saint Louis University, Day 14) 62
Figure S46 Pathway map - Proteasome - Homo sapiens (human) (RNA-Seq, Saint Louis University, Day 1) 63
Figure S47 Pathway map - Proteasome - Homo sapiens (human) (RNA-Seq, Saint Louis University, Day 2) 64
Figure S48 Pathway map - Proteasome - Homo sapiens (human) (RNA-Seq, Saint Louis University, Day 7) 65
Figure S49 Pathway map - Proteasome - Homo sapiens (human) (RNA-Seq, Saint Louis University, Day 14) 66
Figure S50 Pathway map - Protein processing in endoplasmic reticulum - Homo sapiens (human) (RNA-Seq, Saint Louis University, Day 1) 67
Figure S51 Pathway map - Protein processing in endoplasmic reticulum - Homo sapiens (human) (RNA-Seq, Saint Louis University, Day 2) 68
Figure S52 Pathway map - Protein processing in endoplasmic reticulum - Homo sapiens (human) (RNA-Seq, Saint Louis University, Day 7) 69
Figure S53 Pathway map - Protein processing in endoplasmic reticulum - Homo sapiens (human) (RNA-Seq, Saint Louis University, Day 14) 70
Figure S54 Pathway map - Phagosome - Homo sapiens (human) (RNA-Seq, Saint Louis University, Day 1) 71
Figure S55 Pathway map - Phagosome - Homo sapiens (human) (RNA-Seq, Saint Louis University, Day 2) 72

Figure S56 Pathway map - Phagosome - Homo sapiens (human) (RNA-Seq, Saint Louis University, Day 7) 73
Figure S57 Pathway map - Phagosome - Homo sapiens (human) (RNA-Seq, Saint Louis University, Day 14) 74
Figure S58 Pathway map - Antigen processing and presentation - Homo sapiens (human) (RNA-Seq, Saint Louis University, Day 1) 75
Figure S59 Pathway map - Antigen processing and presentation - Homo sapiens (human) (RNA-Seq, Saint Louis University, Day 2) 76
Figure S60 Pathway map - Antigen processing and presentation - Homo sapiens (human) (RNA-Seq, Saint Louis University, Day 7) 77
Figure S61 Pathway map - Antigen processing and presentation - Homo sapiens (human) (RNA-Seq, Saint Louis University, Day 14) 78
Figure S62 Pathway map - Estrogen signaling pathway - Homo sapiens (human) (RNA-Seq, Saint Louis University, Day 1) 79
Figure S63 Pathway map - Estrogen signaling pathway - Homo sapiens (human) (RNA-Seq, Saint Louis University, Day 2) 80
Figure S64 Pathway map - Estrogen signaling pathway - Homo sapiens (human) (RNA-Seq, Saint Louis University, Day 7) 81
Figure S65 Pathway map - Estrogen signaling pathway - Homo sapiens (human) (RNA-Seq, Saint Louis University, Day 14) 82
Figure S66 Pathway map - Pathogenic Escherichia coli infection - Homo sapiens (human) (RNA-Seq, Saint Louis University, Day 1) 83
Figure S67 Pathway map - Pathogenic Escherichia coli infection - Homo sapiens (human) (RNA-Seq, Saint Louis University, Day 2) 84
Figure S68 Pathway map - Pathogenic Escherichia coli infection - Homo sapiens (human) (RNA-Seq, Saint Louis University, Day 7) 85
Figure S69 Pathway map - Pathogenic Escherichia coli infection - Homo sapiens (human) (RNA-Seq, Saint Louis University, Day 14) 86
Figure S70 Pathway map - Legionellosis - Homo sapiens (human) (RNA-Seq, Saint Louis University, Day 1) 87
Figure S71 Pathway map - Legionellosis - Homo sapiens (human) (RNA-Seq, Saint Louis University, Day 2) 88
Figure S72 Pathway map - Legionellosis - Homo sapiens (human) (RNA-Seq, Saint Louis University, Day 7) 89
Figure S73 Pathway map - Legionellosis - Homo sapiens (human) (RNA-Seq, Saint Louis University, Day 14) 90
Figure S74 Venn diagrams summarizing overlap in enriched KEGG Pathways between laboratories (LC-MS/MS and 2D-DIGE/MS) 91
Figure S75 Venn diagrams summarizing overlap in enriched MSigDB Reactome Pathways between laboratories (LC-MS/MS and 2D-DIGE/MS) 92
Figure S76 Venn diagrams summarizing overlap in enriched MSigDB Immunologic Signature Sets between laboratories (LC-MS/MS and 2D-DIGE/MS) 93

Figure S77 Scatterplots to assess correlations between between laboratory log_2 fold changes (Day 0, LC-MS/MS and 2D-DIGE/MS). 94
Figure S78 Scatterplots to assess correlations between between laboratory log_2 fold changes (Day 7, LC-MS/MS and 2D-DIGE/MS). 95
Figure S79 Scatterplots to assess correlations between between laboratory log_2 fold changes (Day 14, LC-MS/MS and 2D-DIGE/MS). 96
Figure S80 Boxplots and empirical cumulative distribution function plots to summarize correlation metrics between laboratory log_2 protein signals (LC-MS/MS and 2D-DIGE/MS). 97
Figure S81 Scatterplots to assess correlations between between laboratory log_2 fold changes (Day 7, LC-MS/MS and 2D-DIGE/MS). 98
Figure S82 Scatterplots to assess correlations between between laboratory log_2 fold changes (Day 14, LC-MS/MS and 2D-DIGE/MS). 99
Figure S83 Boxplots and empirical cumulative distribution function plots to summarize correlation metrics between laboratory log_2 fold changes (LC-MS/MS and 2D-DIGE/MS). 100

List of Tables

Table S1 Overview of filtered gene sets used for the gene set enrichment analysis (LC-MS/MS and 2D-DIGE/MS). 101
Table S2 Summary statistics of proteomics sample metrics (LC-MS/MS, n=30) 101
Table S3 Summary statistics of proteomics sample metrics (2D-DIGE/MS, n=30) 101
Table S4 Identified proteins (2D-DIGE/MS) 103
Table S5 Differentially abundant proteins (LC-MS/MS, Day 7) 104
Table S6 Differentially abundant proteins (LC-MS/MS, Day 14) 106
Table S7 Differentially abundant protein gel spots (2D-DIGE/MS, Day 7) 108
Table S8 Differentially abundant protein gel spots (2D-DIGE/MS, Day 14) 110
Table S9 Enriched KEGG Pathways (LC-MS/MS, Day 7) 111
Table S10 Enriched MSigDB Reactome Pathways (LC-MS/MS, Day 7) 111
Table S11 Enriched MSigDB Immunologic Signature Sets (LC-MS/MS, Day 7) 111
Table S12 Enriched KEGG Pathways (LC-MS/MS, Day 14) 111
Table S13 Enriched MSigDB Reactome Pathways (LC-MS/MS, Day 14) 113
Table S14 Enriched MSigDB Immunologic Signature Sets (LC-MS/MS, Day 14) 115
Table S15 Enriched KEGG Pathways (2D-DIGE/MS, Day 7) 115
Table S16 Enriched MSigDB Immunologic Signature Sets (2D-DIGE/MS, Day 7) 117
Table S17 Enriched KEGG Pathways (2D-DIGE/MS, Day 14) 117
Table S18 Enriched MSigDB Reactome Pathways (2D-DIGE/MS, Day 14) 117
Table S19 Enriched MSigDB Immunologic Signature Sets (2D-DIGE/MS, Day 14) 118
Table S20 Overlapping enriched MSigDB Immunologic Signature Sets (LC-MS/MS and 2D-DIGE/MS, Day 7) 119
Table S21 Overlapping enriched MSigDB Immunologic Signature Sets (LC-MS/MS and 2D-DIGE/MS, Day 14) 119
Table S22 Impact of different normalization procedures on distributions and spike-in metrics (LC-MS/MS) 120
Table S23 Impact of different normalization procedures on distributions and spike-in metrics (2D-DIGE/MS) 120
Table S24 Overlapping differentially abundant proteins (LC-MS/MS and 2D-DIGE/MS) 121
Table S25 List of R packages and versions used for the analyses presented in this report. 122

Abbreviations

2D-DIGE	Two Dimensional Differential Gel Electrophoresis
BCA	Bicinchoninic Acid
CV	Coefficient of Variation
Cy	Cyanine
Da	Dalton
DA	Differentially Abundant
DMID	Division of Microbiology and Infectious Diseases
DTT	Dithiothreitol
DVC	DynPort Vaccine Company
ECDF	Empirical Cummulative Distribution Function
FBS	Fetal Bovine Serum
FDR	False Discovery Rate
IAA	Iodoacetimide
ID	Internal diameter
KEGG	Kyoto Encyclopedia of Genes and Genomes
LC-MS/MS	Liquid Chromatography-Tandem Mass Spectrometry
LFQ	Label-free quantification
LOESS	Local Regression
LVS	Live Vaccine Strain
MAD	Median Absolute Deviation
MS	Mass Spectrometry
MSigDB	Molecular Signatures Database
PBMC	Peripheral Blood Mononuclear Cell
PBS	Phosphate-buffered saline
PPARG	Peroxisome Proliferator-Activated Receptor Gamma
PPM	Parts Per Million
RNA	Ribonucleic Acid
TLR	Toll-Like Receptor
USAMRIID	United States Army Medical Research Institute of Infectious Diseases
VTEU	Vaccine and Treatment Evaluation Unit

Copyright Notice:

The KEGG image files and limited text-based data summary files (**collectively**, the "'KEGG Data Snapshots"') provided herein are copyright ©Kanehisa Laboratories. All rights reserved. By accepting the KEGG Data Snapshots, following terms shall be automatically accepted without limitation:

– Redistribution of the KEGG Data Snapshots is strictly prohibited.

– KEGG Data Snapshots may not be used outside of their intended purpose (i.e. as part of a summary of a data analysis). For example, the data may not be stored or assembled in order to create an internal database, even for personal use.

– A limited number of images included in the KEGG Data Snapshots may be published in printed media with appropriate attributes as is standard in academia.

– The rights granted hereunder are non-transferable.

1 Introduction

This appendix provides supporting information for the manuscript entitled "2D-DIGE and LC/MS/MS analysis of immune responses to live-attenuated tularemia vaccine".

2 Supplemental Methods

2.1 Proteomics experiments

2.1.1 Protein spike-in controls

A spike-in control protein mixture of bovine beta-lactoglobulin (Sigma L-5137), horse myoglobin (Sigma M-9267), and bovine ribonuclease A (Sigma R-6513) was prepared in 8 M urea. Stock solutions of each protein were prepared in Milli-Q grade water, quantified by UV absorbance spectrum, and combined to make 50 mL of 8 M urea solution containing 300 ng/mL of each protein. Details of protein properties and quantitation values used as listed below:

- Beta-lactoglobulin, bovine 18,367.3 g/mol; pI: 4.76; extinction coefficent: 17,210 M^{-1} cm^{-1}; 0.937 mL mg^{-1} cm^{-1}
- Myoglobin, horse 16,951.49 g/mol; pI: 7.2; extinction coefficient was not accurately calculated due to variation of heme occupancy by iron. Used weight measurement and assumed 25% of mass was water to prepare 1 mg/mL stock solution.
- RNaseA, bovine 13,700 g/mol; pI: 9.3; extinction coefficient: 8,640 M^{-1} cm^{-1}; 0.631 mL mg^{-1} cm^{-1}

2.1.2 Shared protein sequence database and protein families

The joint human subset (organism restricted to *Homo sapiens*) of the UniProtKB/Swiss-Prot and UniProtKB/TrEMBL Release 2016-03 of 16-Mar-2016 was obtained and used as a reference for proteomics searches. The *CD-HIT* software (Version 4.0 beta) was used to derive protein clusters at a 50% protein sequence identity level (henceforth, 50% CD-HIT protein clusters are referred to as protein families). Prior to proteomics searching, identical protein sequences were collapsed and the three spike-in control protein sequences were added to the protein sequence database (UniProt Accession P02754: Beta-lactoglobulin (Bovine), UniProt Accession P68082: Myoglobin (Horse), and UniProt Accession P61823: Ribonuclease pancreatic (Bovine)).

2.1.3 LC-MS/MS proteomics experiment and data processing

One mL of ice cold PBS was added to each sample and the samples were centrifuged for 10 minutes at 400g. Afterwards, 1.5 mL of supernatant was removed and discarded. An additional 500 uL of ice cold PBS was added and the samples were spun again at 1000g for 10 minutes. The supernatant was removed and the pellet was resuspended in 300 uL of urea lysis buffer (8M Urea spiked with 3 proteins), including 3 uL (100x stock) HALT protease and phosphatase inhibitor cocktail (Pierce). The entire mixture was then sonicated (Sonic Dismembrator, Fisher Scientific) 3 times for 5 s with 15 s intervals of rest at 30% amplitude to disrupt nucleic acids and was subsequently vortexed. Protein concentration was determined by the bicinchoninic acid (BCA) method, and

samples were frozen in aliquots at -80C. Protein homogenates (100 mcg) were diluted with 50 mM NH4HCO3 to a final concentration of less than 2M urea and then treated with 1 mM dithiothreitol (DTT) at 25C for 30 minutes, followed by 5 mM iodoacetimide (IAA) at 25C for 30 minutes in the dark. Protein was digested with 1:100 (w/w) lysyl endopeptidase (Wako) at 25C for 2 hours and further digested overnight with 1:50 (w/w) trypsin (Promega) at 25C. Resulting peptides were desalted with a Sep-Pak C18 column (Waters) and dried under vacuum.

Dried peptides were resuspended in 100 uL of loading buffer (0.1% formic acid, 0.03% trifluoroacetic acid, 1% acetonitrile). Peptide mixtures (2 uL) were separated on a self-packed C18 (1.9 um Dr. Maisch, Germany) fused silica column (25 cm x 75 uM internal diameter (ID); New Objective, Woburn, MA) by a Dionex Ultimate 3000 RSLCNano and monitored on a Fusion mass spectrometer (ThermoFisher Scientific, San Jose, CA). Elution was performed over a 140 minute gradient at a rate of 300nl/min with buffer B ranging from 3% to 80% (buffer A: 0.1% formic acid in water, buffer B: 0.1% formic in acetonitrile). The mass spectrometer cycle was programmed to collect at the top speed for 3 second cycles. The MS scans (400-1600 m/z range, 200,000AGC, 50 ms maximum ion time) were collected at a resolution of 120,000 at m/z 200 in profile mode and the HCD MS/MS spectra (2 m/z isolation width, 30% collision energy, 10,000 AGC target, 35 ms maximum ion time) were detected in the ion trap. Dynamic exclusion was set to exclude previous sequenced precursor ions for 20 seconds within a 10 ppm window. Precursor ions with +1, and +8 or higher charge states were excluded from sequencing.

Raw data for the samples was analyzed using MaxQuant v1.5.3.30 with Thermo Foundation 2.0 for RAW file reading capability. The search engine Andromeda, integrated into MaxQuant, was used to build and search a concatenated target-decoy human reference protein database (20157 target entries plus 245 contaminant proteins from the common repository of adventitious proteins (cRAP)built into MaxQuant). Methionine oxidation (+15.9949 Da), asparagine and glutamine deamidation (+0.9840 Da), and protein N-terminal acetylation (+42.0106 Da) were variable modifications (up to 5 allowed per peptide); cysteine was assigned a fixed carbamidomethyl modification (+57.0215 Da). Only fully tryptic peptides were considered with up to 2 miscleavages in the database search. A precursor mass tolerance of +-20 ppm was applied prior to mass accuracy calibration and +-4.5 ppm after internal MaxQuant calibration. Cofragmented peptide search was enabled to deconvolute multiplex spectra. The false discovery rate (FDR) for peptide spectral matches, proteins, and site decoy fraction were all set to 1 percent. Quantification settings were as follows: requantify with a second peak finding attempt after protein identification has completed; match MS1 peaks between runs; a 0.7 min retention time match window was used after an alignment function was found with a 20 minute RT search space. Label-free quantification (LFQ) of proteins and normalization was performed using the MaxLFQ algorithm as implemented in MaxQuant. The quantitation method only considered razor plus unique peptides for protein level quantitation.

Protein group signals were filtered to retain groups for which at least two unique peptides were identified. Zero intensity values were set to missing. The leading protein in a protein group (the protein with the highest number of identified peptides) was used as the representative protein for each group. Information about other proteins in a protein group was retained and integrated when presenting lists of differentially abundant proteins.

2.1.4 2D-DIGE/MS proteomics experiment and data processing

PBMC cell pellets were prepared by controlled thawing of cryopreserved cells in order to maximize viability, which was assessed by trypan blue stain. Cells were counted by hemocytometer and washed twice with PBS buffer

prior to freezing in aliquots of 2 million viable cells per vial. Cell pellets were lysed, proteins precipitated, and total protein quantified. Protein samples were fluorescently labeled for 2D-DIGE analysis using Cy3/Cy5/Cy2 labels. Prior to labeling, an equal protein quantity was taken from each cell lysate to create a pool for normalization of fluorescence intensity for all analytical 2D gels. This pool was labeled with Cy2 dye, and the individual samples were labeled with either Cy3 or Cy5. All labeling reactions were carried out using the same molar ratio of dye to protein to produce substoichiometric trace labeling of lysines on the protein. The samples were analyzed by 2D gel electrophoresis. Fluorescence scanning of gels to detect Cy2, Cy3, or Cy5 signal was carried out on each gel separately.

Raw data files were initially cropped and filtered for noise using ImageQuant software. The resulting image files were analyzed using DeCyder software according to the same SOP to calculate relative abundance of each detected protein spot. The set of gels were also matched to each other by DeCyder for further analysis according to desired grouping of samples.

These relative abundance data did not contain protein identifications. 2D-DIGE spots corresponding to spiked proteins were identified using mass spectrometry. Using MS-based identification, 41 2D-DIGE gel spots were linked to 68 reference sequence database entries (UniProt IDs). A listing of all gel spot IDs with protein identifications is provided in **Table S4**. Note, some gel spots were tested mutiple times.

2.2 Statistical analysis

2.2.1 Data normalization

Median normalization to account for systematic differences in protein signal distributions by aligning the medians of the $\log_2$ protein signal distributions across the 30 samples involved the following steps:

(1) for each sample, the median of the $\log_2$ protein signal distribution was determined

(2) the global median of all 30 sample medians calculated in (1) was obtained

(3) a sample specific scaling factor was then calculated as the difference ($\log_2$ scale) between the global median obtained in (2) and the sample-specific median obtained in (1)

(4) the $\log_2$ protein signal distribution for each sample was then median-normalized by adding the scaling factor ($\log_2$ scale) determined in (3)

Local regression (LOESS)-based normalization as implemented in the *affy* R package (Version 1.48.0) was used to correct systematic signal-dependent non-linear bias observed for Cy5 versus Cy3-labeled 2D-DIGE data (**Figures S11 to S13**).

2.2.2 Missing value imputation and log fold change from baseline calculation

Missing observations were imputed using the k-nearest neighbors algorithm implemented in the *impute* R package (Version 1.44.0). Only proteins/spots with at least 24/30 (80%) non-missing observations were used as input for imputation and downstream analysis. The number of neighbors to be used as part of the imputation step was set to 8. The maximum percentage of allowed missing observations for any sample was set to 80%. Subject-specific $\log_2$ protein fold changes from baseline were calculated based on normalized imputed $\log_2$ signals for

each subject and post-vaccination day (day 7, 14) by subtracting baseline (day 0) protein signals from each of the subject's post-vaccination day signals.

2.2.3 Identification of differentially abundant proteins

Proteins that significantly differed in their response from baseline were identified by using a two-sided permutation paired t-test comparing baseline (day 0) to post-vaccination (day x) protein signals ($H_0 : \mu(day_x - day_0) = 0$, $H_1 : \mu(day_x - day_0) \neq 0$; on the $\log_2$ scale). The false-discovery rate (FDR) based on the Benjamini-Hochberg procedure as implemented in the *p.adjust* R function was calculated. Proteins with an individual p-value < 0.05 and baseline fold change ≥ 1.2 were considered significantly differentially abundant (DA) proteins.

2.2.4 Pathway enrichment analysis

Pathway enrichment was carried out separately for each post-vaccination day using 5,850 known gene sets obtained from the KEGG Pathway (Version 79.0, 07/16/2016) and MSigDB (Version 5.1, 01/19/2016 including MSigDB Reactome Pathways, MSigDB Immunologic Signatures) databases. Prior to analysis, proteins in the proteomics protein database were mapped to Ensembl Gene IDs (Ensembl release 84, March 2016) using the *biomaRt* R package(Version 2.26.1) based on their UniProt protein accessions. If a UniProt protein accession mapped to multiple Ensembl Gene IDs, multiple Ensembl Gene IDs were assigned to that protein. Following the mapping step, genes in gene sets without any UniProt protein accession mappings were excluded from the gene set collections. Gene set statistics after filtering are provided in **Table S1**.

For each of the filtered gene sets, enrichment was evaluated using the *goseq* R package (Version 1.12.0) using the hypergeometric distribution to assess statistical significance. To adjust for testing multiple gene sets per category type, the Benjamini-Hochberg procedure was applied to each list. Gene sets with a FDR $\leq$ 0.1 were considered to be significantly enriched. For significantly enriched KEGG pathways, color-coded KEGG pathway maps were generated (KEGG KGML pathway layout information Version 81.0, 03/20/2017). Node background was color-coded by mean $\log_2$ fold change from pre-vaccination (red: increased from baseline, blue: decreased from baseline). For the 2D-DIGE/MS data, the largest mean $\log_2$ fold change was used for UniProt IDs with multiple gel spot IDs. If nodes in the pathway referred to multiple genes, the median $\log_2$ fold change was used to set the background color of that node (red: increased from pre-vaccination, blue: decreased from pre-vaccination). If one of the genes of a multi-gene node was significantly enriched, the node label and border was color-coded (red: increased from pre-vaccination, blue: decreased from pre-vaccination, yellow: conflict if one gene was up but another was down-regulated for the same pathway node).

2.3 Comparison of shared proteins

For individual sample comparisons, 2D-DIGE/MS data was further processed by collapsing reference database entries (UniProt IDs) with multiple gel spot IDs using the largest normalized and imputed $\log_2$ spot volume ratio among gel spot IDs (7 out of 59 uniquely identified UniProt IDs mapped to one or more gel spot ID). Collapsed ratios from pre and post-vaccination time points were then used to calculate fold changes per UniProt ID. Collapsed data was used to assess correlations between sample protein abundances and fold changes (Pearson

correlation and Spearman's rank correlation) as well as to summarize fold change responses of shared proteins in the form of heatmaps.

2.4 Statistical analysis plan deviations

Median absolute deviation (MAD) was added as a measure of variability as the coefficient of variation (CV) did not robustly capture variation for the 2D-DIGE data due to mean $\log_2$ protein signals being close to zero. As FDR-adjustment for the permutation paired t-test was too stringent to detect any DA proteins for a wide range of FDR cut offs for both laboratories, proteins with an individual p-value < 0.05 and baseline fold change ≥ 1.2 were considered DA proteins. The most recent software/R packages were installed and used except for CD-HIT for which an older version (Version 4.0 beta) was used to obtain protein families.

2.5 Software

Data was analyzed using the *R statistical programming language* (Version 3.2.5) and *R Bioconductor* (Version 3.2) packages. This report was generated using the *knitr* R package (Version 0.4-10) and *LaTeX* typesetting software (Version TeX Live 2012/Debian). The operating system used was *Ubuntu* (Version 13.04). Additional software along with version information is listed in the respective method sections and in **Table S25**.

3 Supplemental Results

3.1 Data normalization and quality control

3.1.1 Summary statistics

Summary statistics for the 30 proteomics experiments carried are provided in **Tables S2 and S3**, respectively. Corresponding boxplots are shown in **Figures S2 and S3**. Multivariate starplots that contrast LC-MS/MS experiment metrics across time points and subjects are displayed in **Figure S4**. On average, 748 gel spots were identified for each 2D-DIGE experiment and 1,639 unique protein groups were identified for the LC-MS/MS experiment (**Tables S2 and S3**). For the LC-MS/MS experiment, the median protein length ranged from 380 to 419 amino acids with a median molecular weight between 42.6 and 46.7 Da and a median isoelectric point range of 6.2 to 6.6 (**Table S2**).

3.1.2 Data normalization

Distributions of the original $\log_2$ protein signals in the form of boxplots are given in **Figures S5 and S7**. Empirical cumulative distribution function (ECDF) plots for each laboratory are displayed in **Figures S9 and S10**. The 2D-DIGE data showed a noticeable fluorescent dye effect introducing higher variability for Cy5 compared to Cy3 spot volume ratios (**Figure S7**). The dye effect was confirmed when visualizing the per-gel fold change between dyes and average signal using MA plots (**Figures S11 to S13**). These plots revealed a systematic non-linear signal-dependent dye bias with higher Cy5 spot volume ratios for low average signals versus higher Cy3 spot volume ratios for high average signals. For the LC-MS/MS data, noticeable shifts between medians across the 30 samples were observed (**Figure S5**).

Several normalization strategies were evaluated to reduce systematic bias. To assess the impact of normalization, variability in distributional properties (median and interquartile ranges) as well as spike-in control $\log_2$ protein signal across the 30 samples was numerically assessed using the median absolute deviation (MAD), a robust measure of variability (**Tables S22 and S23**). LOESS normalization successfully corrected the systematic non-linear signal-dependent dye effect observed for the 2D-DIGE data (**Figures S11 to S13**) and reduced the difference in variability observed for Cy5 and Cy3-labeled samples (**Figure S8 and Table S23**). It also reduced the MAD of $\log_2$ spot volume ratios of both spike-in proteins (**Table S23**). Median normalization of the LC-MS/MS data successfully aligned distributions with respect to their centers (**Figure S6** and **Figure S9**). It also reduced the MAD of two of the three spike-in proteins (**Table S22**). Thus, LOESS-normalized 2D-DIGE and median-normalized LC-MS/MS data were used for downstream analysis.

Distributions of the original $\log_2$ protein signals in the form of boxplots are given in **Figures S5 and S7**. Empirical cumulative distribution function (ECDF) plots for each laboratory are displayed in **Figures S9 and S10**. The 2D-DIGE data showed a noticeable fluorescent dye effect introducing higher variability for Cy5 compared to Cy3 spot volume ratios (**Figure S7**). The dye effect was confirmed when visualizing the per-gel fold change between dyes and average signal using MA plots (**Figures S11 to S13**). These plots revealed a systematic non-linear signal-dependent dye bias with higher Cy5 spot volume ratios for low average signals versus higher Cy3 spot volume ratios for high average signals. For the LC-MS/MS data, noticeable shifts between medians across the 30 samples were observed (**Figure S5**).

Several normalization strategies were evaluated to reduce systematic bias. To assess the impact of normalization, variability in distributional properties (median and interquartile ranges) as well as spike-in control $\log_2$ protein signal across the 30 samples was numerically assessed using the median absolute deviation (MAD), a robust measure of variability (**Tables S22 and S23**). LOESS normalization successfully corrected the systematic non-linear signal-dependent dye effect observed for the 2D-DIGE data (**Figures S11 to S13**) and reduced the difference in variability observed for Cy5 and Cy3-labeled samples (**Figure S8 and Table S23**). It also reduced the MAD of $\log_2$ spot volume ratios of both spike-in proteins (**Table S23**). Median normalization of the LC-MS/MS data successfully aligned distributions with respect to their centers (**Figure S6** and **Figure S9**). It also reduced the MAD of two of the three spike-in proteins (**Table S22**). Thus, LOESS-normalized 2D-DIGE and median-normalized LC-MS/MS data were used for downstream analysis.

3.1.3 Comparison of spike-in control measurements

Boxplots and ECDF plots that contrast spike-in protein (beta-lactoglobulin (bovine), myoglobin (horse), and ribonuclease pancreatic (bovine)) variability across the 30 samples within and between laboratories are displayed in **Figures S14 and S15**, respectively. Note, for the 2D-DIGE experiment, RNaseA could not confidently be assigned to a spot in the master gel. Thus, only beta-lactoglobulin and myoglobin were summarized for the 2D-DIGE data. For each laboratory, the coefficient of variation (CV) and robust MAD was calculated (see x-axis labels in **Figure S14**). To compare variability more directly between laboratories, $\log_2$ mean-centered protein signals were used. Results are summarized in the bottom panel of **Figures S14 and S15**. For both laboratories, the beta-lactoglobulin (bovine) spike-in protein showed higher variability compared to myoglobin (horse). While beta-lactoglobulin (bovine) was similar in variability (as assessed by MAD) between laboratories following normalization, differences were more pronounced for myoglobin (horse). This was also observed when contrasting

interquartile ranges for mean-centered protein signals between laboratories (**Figure S14** bottom right). Overall, the MAD was lower for the 2D-DIGE experiment compared to the LC-MS/MS experiment with an 8% reduction in MAD for bovine beta-lactoglobulin (0.58 vs. 0.63) and a 33% reduction in MAD for horse myoglobin protein (0.35 vs. 0.52) (**Figure S14**).

3.1.4 Missing observations

To assess the degree of missing observations, ECDF plots for each laboratory that summarize the percentage of proteins with increasing percentage of missing observations (from 0-100% missing observations) were generated (**Figure S16**). For both laboratories, a negative relationship between the number of missing observations and average $\log_2$ protein signal was observed (**Figure S17**, r_s=0.87 and r_s=0.39 for LC-MS/MS and 2D-DIGE data, respectively) indicating some degree of left-censoring. However, there was still considerable variation, in particular for 2D-DIGE pointing towards a mixture of random missing observations and left censoring. K-nearest neighbor imputation was applied to fill-in missing observations (see methods).

3.1.5 Global protein abundance patterns and outlying samples

Principal component, non-metric multidimensional scaling, and hierarchical clustering results for standardized imputed $\log_2$ protein signals before and after normalization are shown in **Figures S18 to S21**. No strong outliers or strong batch effects were observed.

3.2 Identification and comparison of differentially abundant proteins

DA proteins identified for the LC-MS/MS data by post-vaccination day including gene annotations, p-values, FDR-adjusted p-values, t-statistics, and $\log_2$ fold change estimates are tabulated in **Tables S5 and S6**. Gel spot IDs with differentially abundant volume ratios between pre and post-vaccination samples for the 2D-DIGE data are listed in **Tables S7 and S8**. Overlap in identified DA proteins/gel spots between post-vaccination days are summarized in **Figure S22** using Venn diagrams, both in terms of overall numbers and separately for increased/decreased DA proteins.

Using MS-based identification, we further characterized 2D-DIGE 41 gel spots linking them to 68 reference sequence database entries of which 59 were unique. A listing of all gel spot IDs with protein identifications is provided in **Table S4**. Protein identifications and associated protein annotations were added to 2D-DIGE/MS DA results where applicable (**Tables S7 and S8**). Venn diagrams that summarize the overlap in DA proteins among shared protein identifications between the LC-MS/MS and 2D-DIGE/MS experiments are shown in **Figure S27**. A list of overlapping identified proteins is given in **Table S24**.

3.3 Determination of higher order organization of differentially abundant proteins

Heatmaps that summarize baseline $\log_2$ protein fold change patterns of DA proteins by laboratory (LC-MS/MS and 2D-DIGE/MS data) and post-vaccination day are presented in **Figures S23 to S26**. To contrast fold change responses for DA proteins between laboratories, responses for shared DA proteins (DA at day 7 or 14) with reference sequence database mappings for both laboratories were identified (31 DA proteins) and visualized

(**Figures S28 and S29**).

To evaluate the functional context of DA proteins, pathway enrichment analysis was carried out for each laboratory (LC-MS/MS and 2D-DIGE/MS data) and post-vaccination day (day 7 and 14). Significantly enriched KEGG Pathways, MSigDB Reactome Pathways, and MSigDB Immunologic Signatures (**Table S1**) for the LC-MS/MS data are listed in **Tables S9 to S14**. Corresponding pathway maps summarizing LC-MS/MS signals for enriched KEGG pathways color-coded by protein fold change are shown in **Figures S30 to S32**. Tabular pathway enrichment results for the 2D-DIGE/MS data are provided in **Tables S15 to S19**. Corresponding pathways for the 2D-DIGE/MS data are provided in **Figures S33 to S41**. Venn diagrams that summarize the overlap in enriched pathways between LC-MS/MS and 2D-DIGE/MS data are shown in **Figures S74 to S76**. Lists of overlapping enriched pathways are given in **Tables S20 and S21**.

3.4 Comparison of shared proteins

Using MS-based identification, we further characterized 41 gel spots linking them to 68 reference sequence database entries (**Table S4**). Database entries with multiple spot allocations were collapsed using the largest absolute spot volume ratio per database entry. The collapsed dataset contained 59 entries of which 35 (59%) were included in the LC-MS/MS imputed analysis dataset. To assess the correlation between normalized and imputed laboratory measurements for these 35 shared proteins, scatter plots were generated for each sample (subject and time point combination) (**Figures S77 to S79**). Linear regression as well as locally weighted regression fits were added to each scatter plot and the relationship was assessed using Pearson correlation coefficient (linear increase/decrease) as well as Spearman's rank correlation coefficient (monotonic increase/decrease). Distributions of correlation metrics across all 30 samples are summarized in **Figure S80**. Corresponding figures that assess agreement between shared protein fold changes for each sample from the same subject for Days 7 and 14 are shown in **Figures S81 and S82**, respectively. Overall fold change correlation metrics are summarized in **Figure S83**.

4 References

[1] Crompton JG, et al. Lineage relationship of CD8+ T cell subsets is revealed by progressive changes in the epigenetic landscape. Cellular and Molecular Immunology. 2016, 13:502-513.
[2] Pierce EL, Poffenberger MC, Chang CH, Jones RG. Fueling immunity: insights into metabolism and lymphocyte function. Science 2013; 324:1242454
[3] http://software.broadinstitute.org/gsea/msigdb/cards/GSE2405_0H_VS_9H_A_
PHAGOCYTOPHILUM_STIM_NEUTROPHIL_DN
[4] Borjesson, Dori L., et al. "Insights into pathogen immune evasion mechanisms: Anaplasma phagocytophilum fails to induce an apoptosis differentiation program in human neutrophils." The Journal of Immunology 174.10 (2005): 6364-6372.
[5] http://software.broadinstitute.org/gsea/msigdb/cards/GSE25123_WT_VS_PPARG_KO_
MACROPHAGE_DN
[6] Szanto, Attila, et al. "STAT6 transcription factor is a facilitator of the nuclear receptor PPAR-regulated gene expression in macrophages and dendritic cells." Immunity 33.5 (2010): 699-712.

[7] http://software.broadinstitute.org/gsea/msigdb/cards/GSE37532_TREG_VS_TCONV_CD4_TCELL_FROM_LN_UP
[8] Cipolletta, Daniela, et al. "PPAR is a major driver of the accumulation and phenotype of adipose tissue T reg cells." Nature 486.7404 (2012): 549.
[9] http://software.broadinstitute.org/gsea/msigdb/cards/GSE37532_WT_VS_PPARG_KO_VISCERAL_ADIPOSE_TISSUE_TREG_UP
[10] http://software.broadinstitute.org/gsea/msigdb/cards/GSE37532_TREG_VS_TCONV_PPARG_KO_CD4_TCELL_FROM_LN_DN
[11] Lim, Hanjo, et al. "Identification of 2D-gel proteins: a comparison of MALDI/TOF peptide mass mapping to LC-ESI tandem mass spectrometry." Journal of the American Society for Mass Spectrometry 14.9 (2003): 957-970.
[12] Wu, Wells W., et al. "Comparative study of three proteomic quantitative methods, DIGE, cICAT, and iTRAQ, using 2D gel-or LC MALDI TOF/TOF." Journal of proteome research 5.3 (2006): 651-658.
[13] Cox, Juergen, and Matthias Mann. "MaxQuant enables high peptide identification rates, individualized ppb-range mass accuracies and proteome-wide protein quantification." Nature biotechnology 26.12 (2008): 1367.

Figures

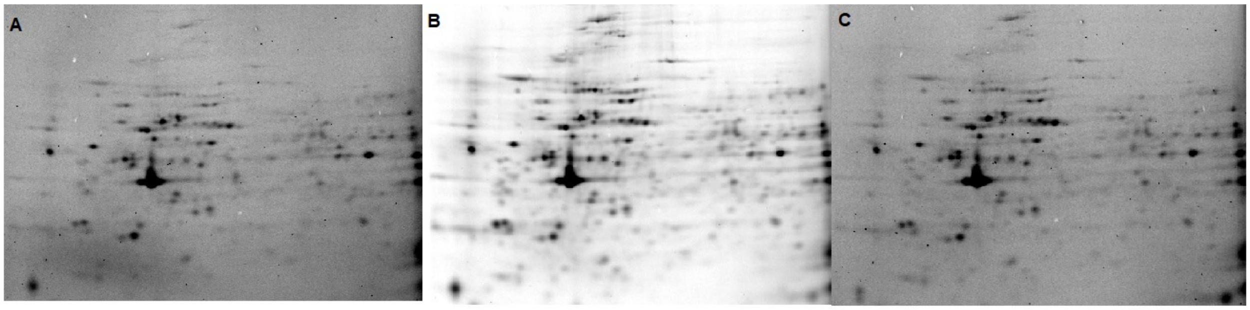

Figure S1: Representative 2D-DIGE gel images. Each sample was labeled with Cy3 (A) or Cy5 (B). Pooled samples were labeled with Cy2 (C) and used as the control.

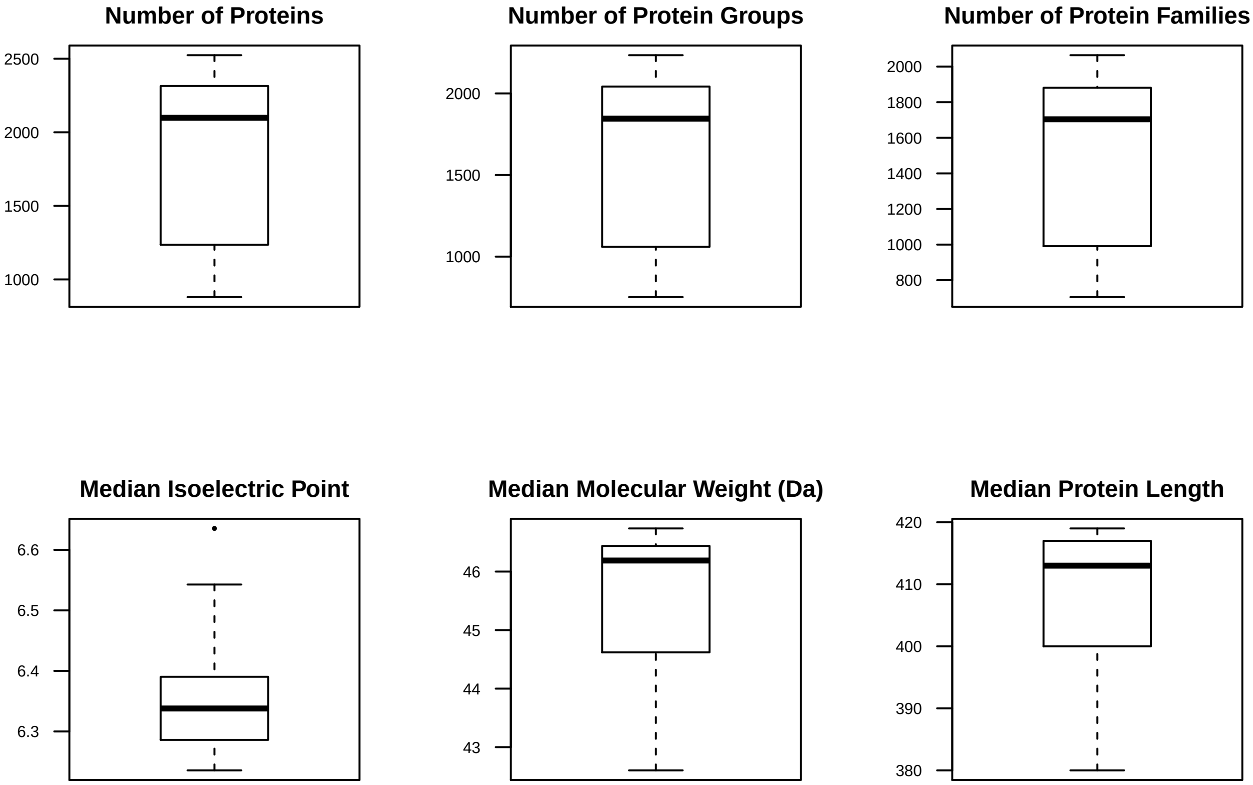

Figure S2: Boxplots of proteomics sample metrics (LC-MS/MS).

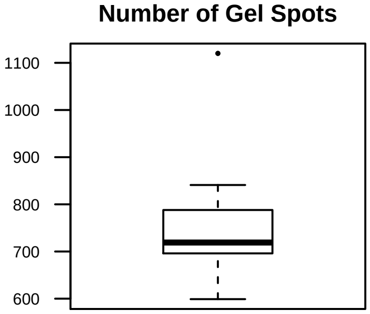

Figure S3: Boxplots of proteomics sample metrics (2D-DIGE/MS). The number of gel spots is based on the number of spots per gel that were mapped to the master gel.

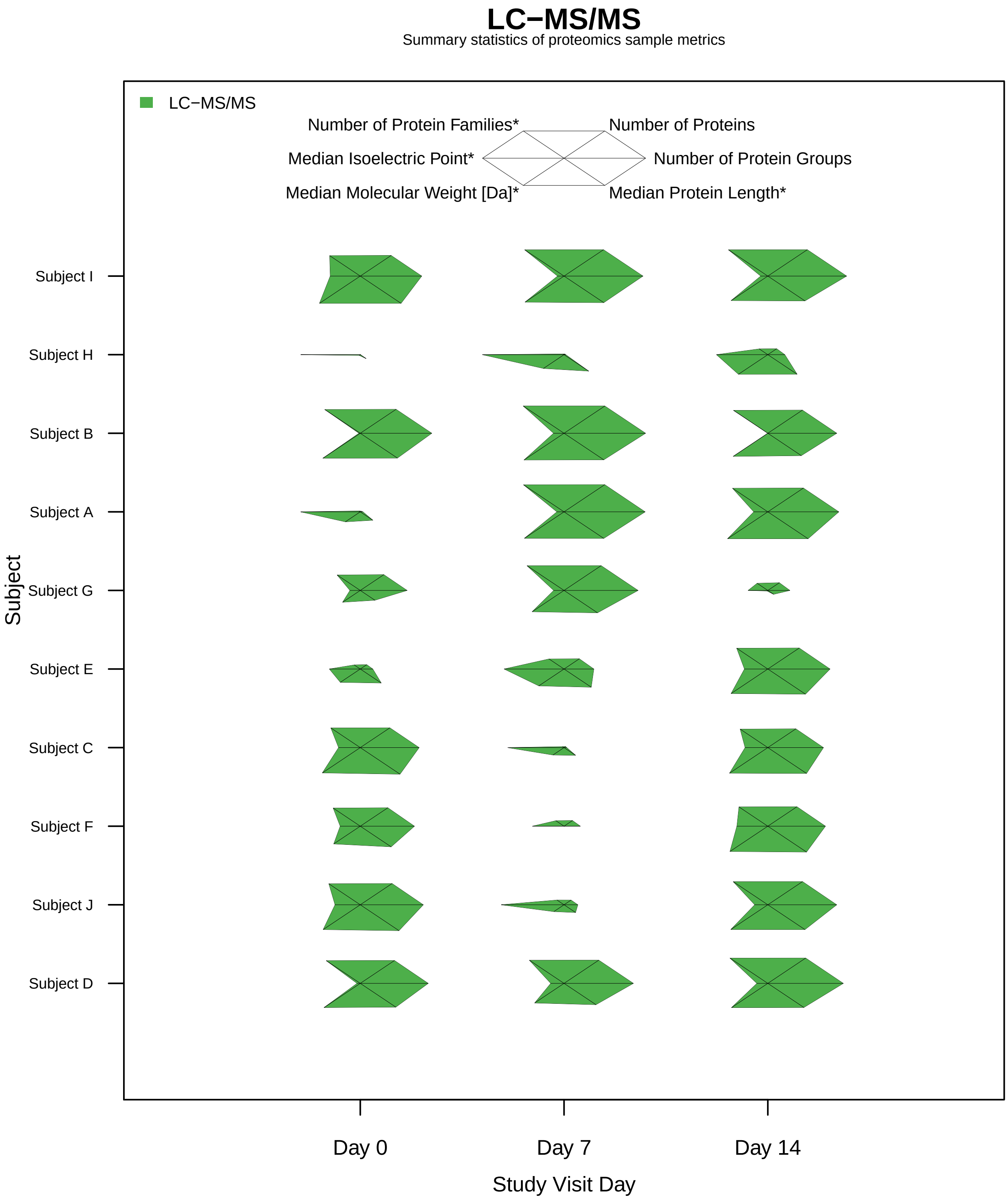

Figure S4: Starplots of proteomics sample metrics (LC-MS/MS). *: restricted to the representative protein in a protein group (leading protein). Protein families were defined at a 50% sequence identity level.

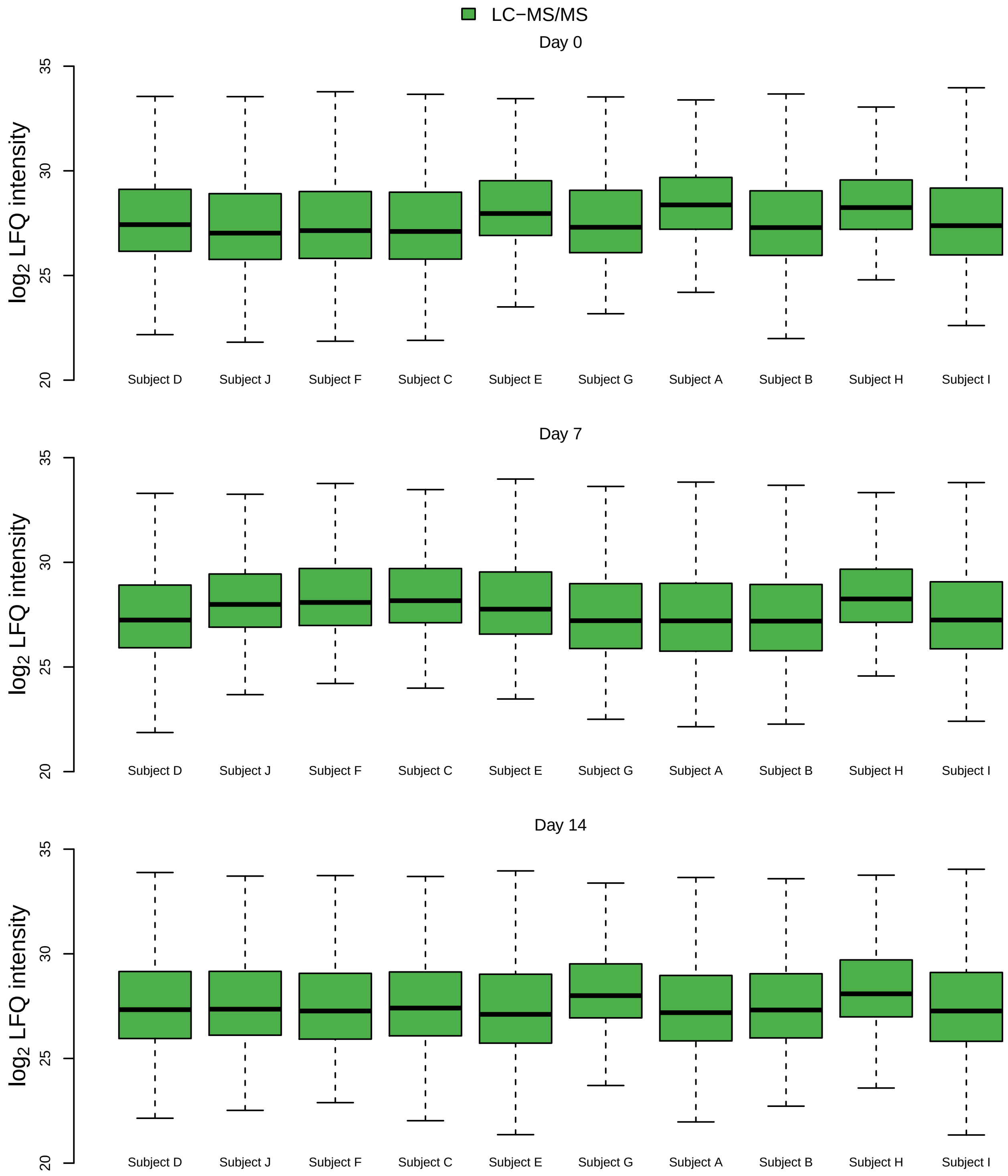

Figure S5: Boxplots of log_2 LFQ intensity before median normalization (LC-MS/MS). Outliers are not shown to highlight shifts in center and scale.

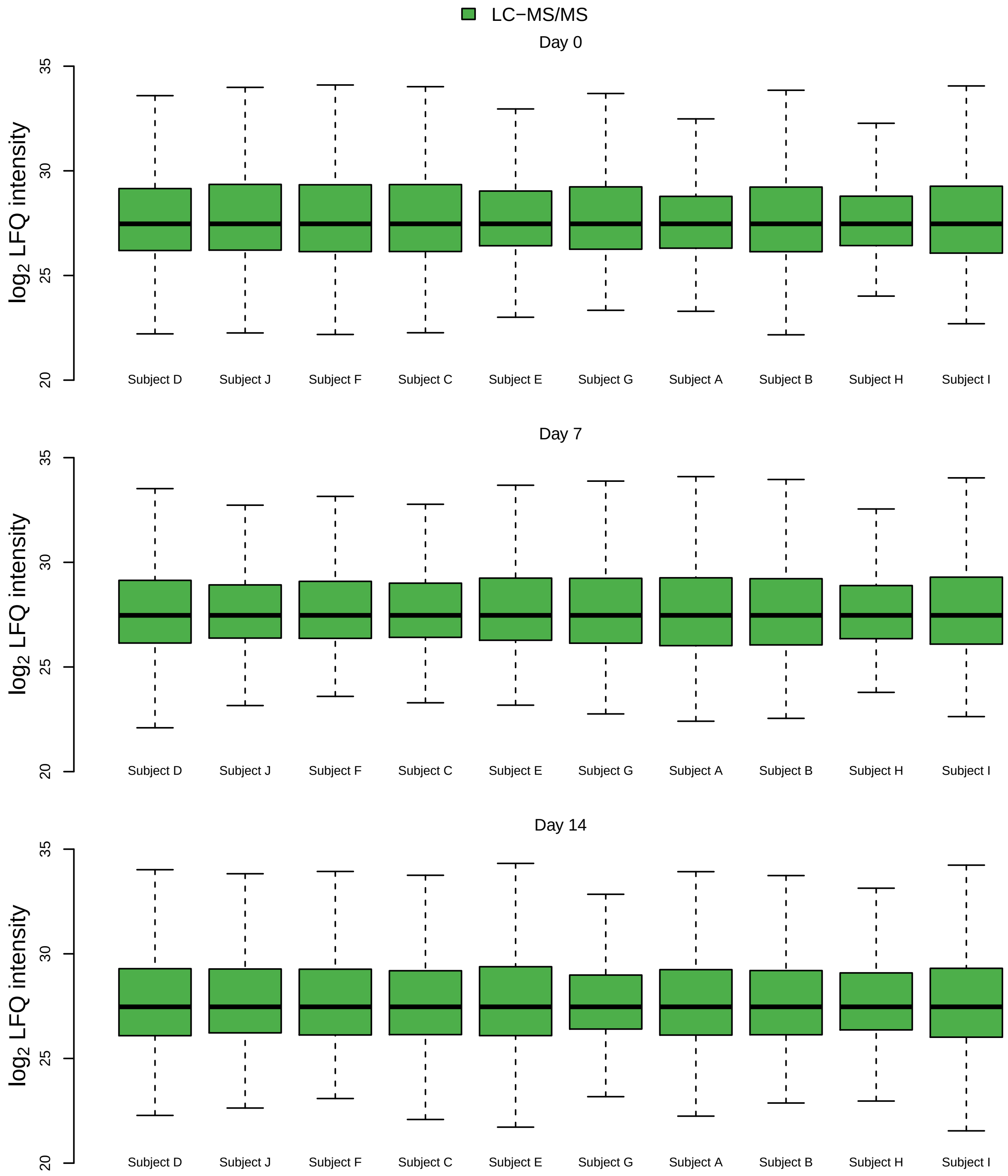

Figure S6: Boxplots of log_2 LFQ intensity after median normalization (LC-MS/MS). Outliers are not shown to highlight shifts in center and scale.

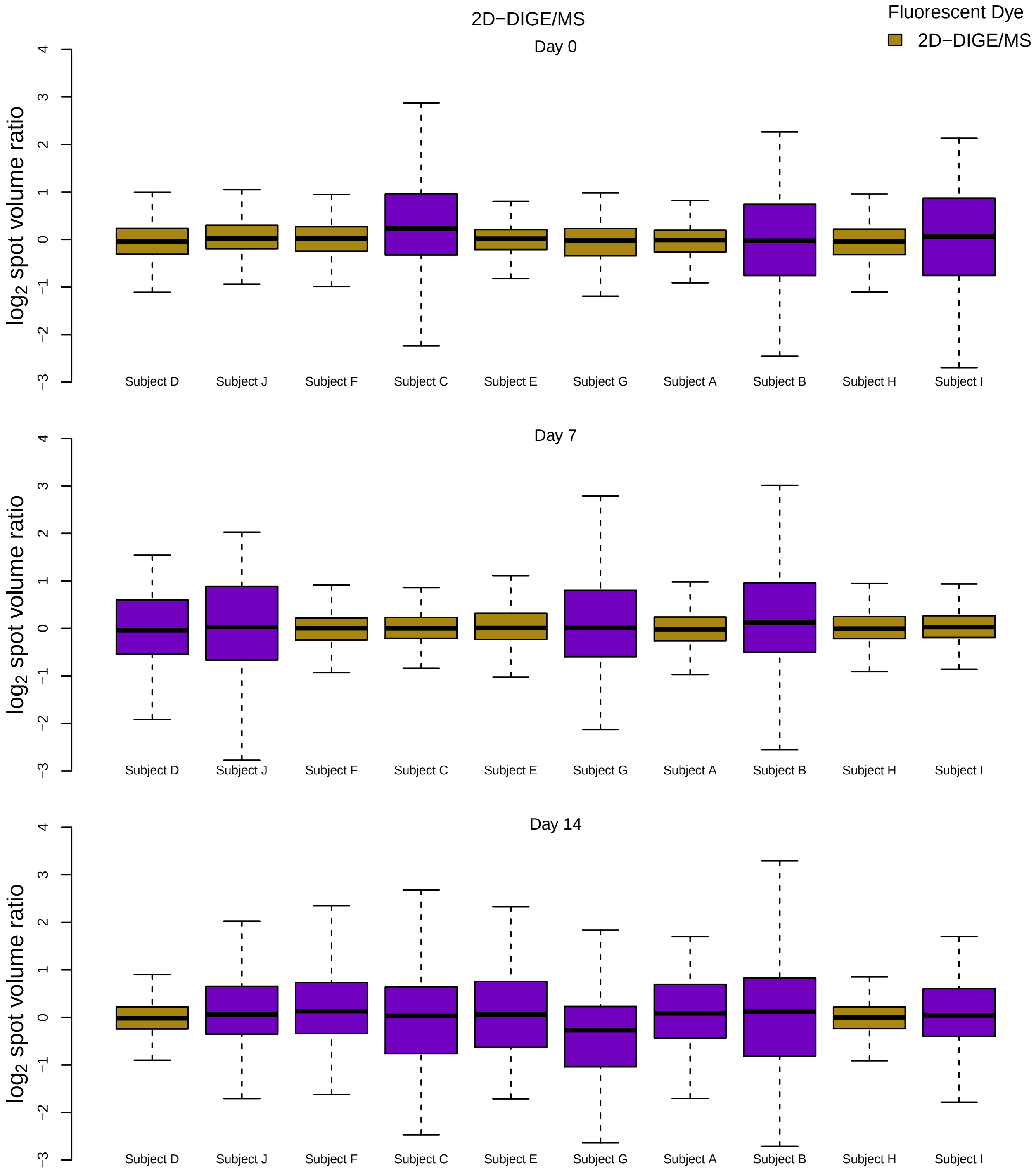

Figure S7: Boxplots of log_2 spot volume ratios before LOESS normalization (2D-DIGE/MS). Outliers are not shown to highlight shifts in center and scale.

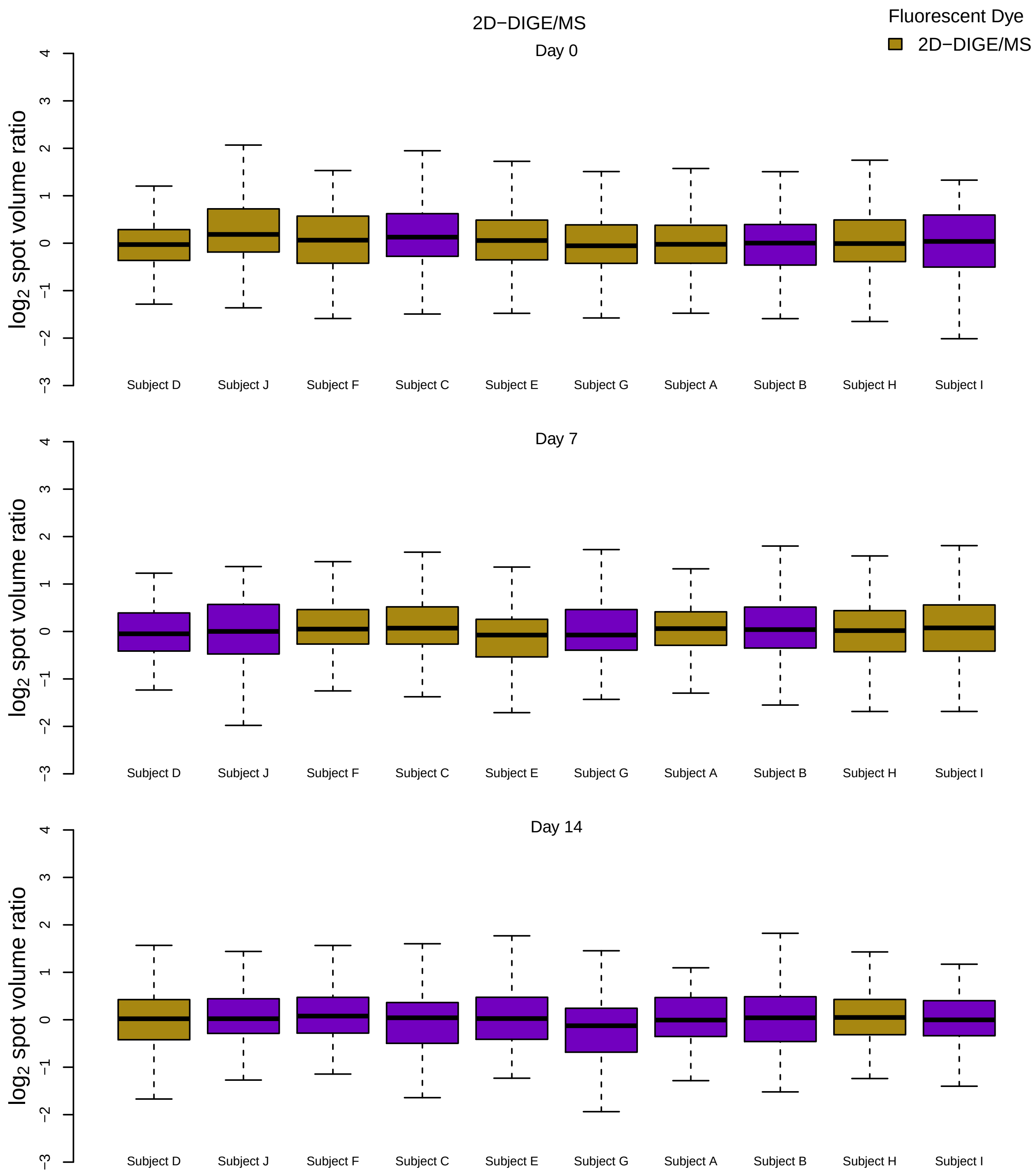

Figure S8: Boxplots of log_2 spot volume ratios after LOESS normalization (2D-DIGE/MS). Outliers are not shown to highlight shifts in center and scale.

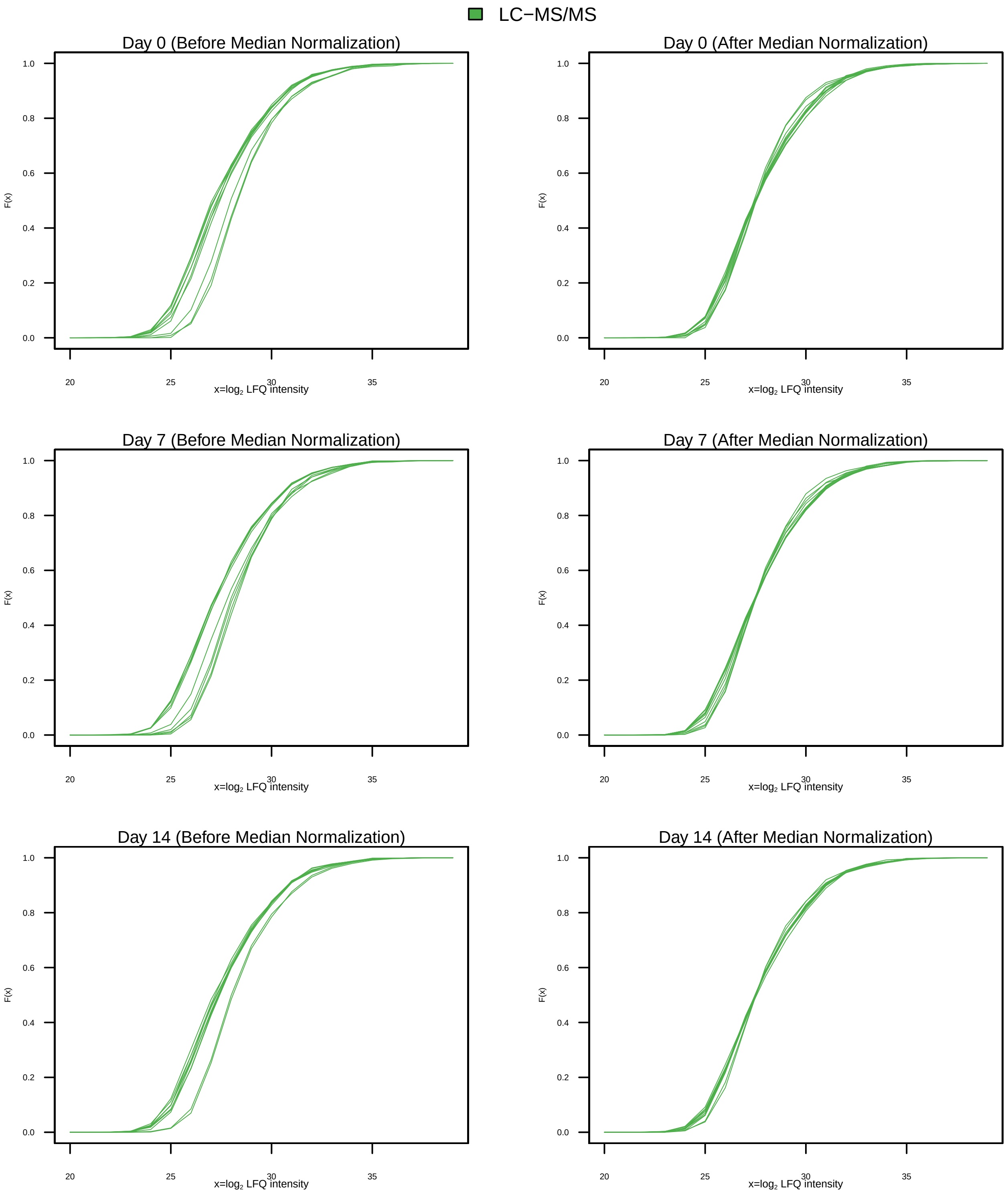

Figure S9: ECDF plots of log_2 LFQ intensity before and after median normalization (LC-MS/MS).

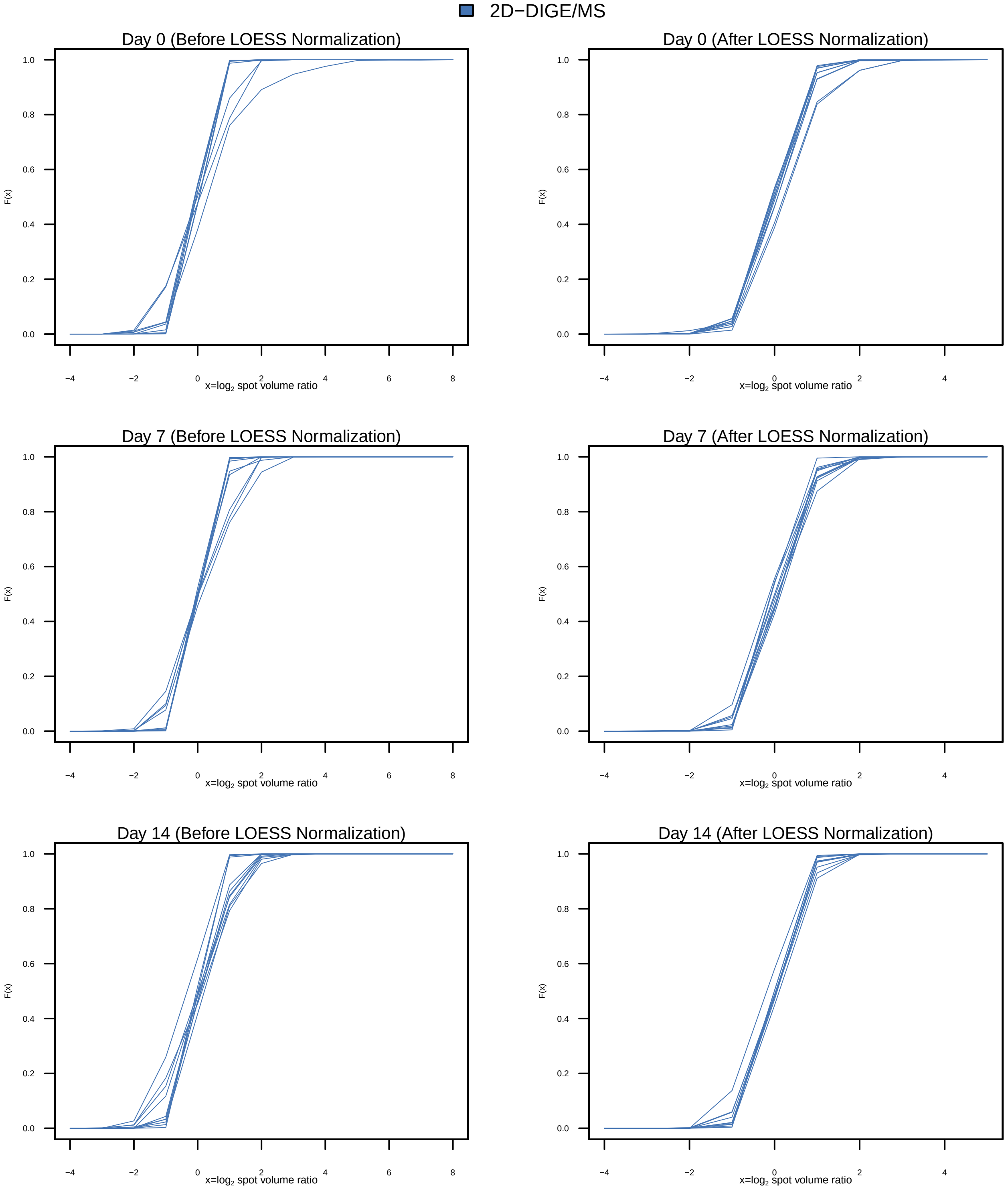

Figure S10: ECDF plots of log_2 spot volume ratios before and after LOESS normalization (2D-DIGE/MS).

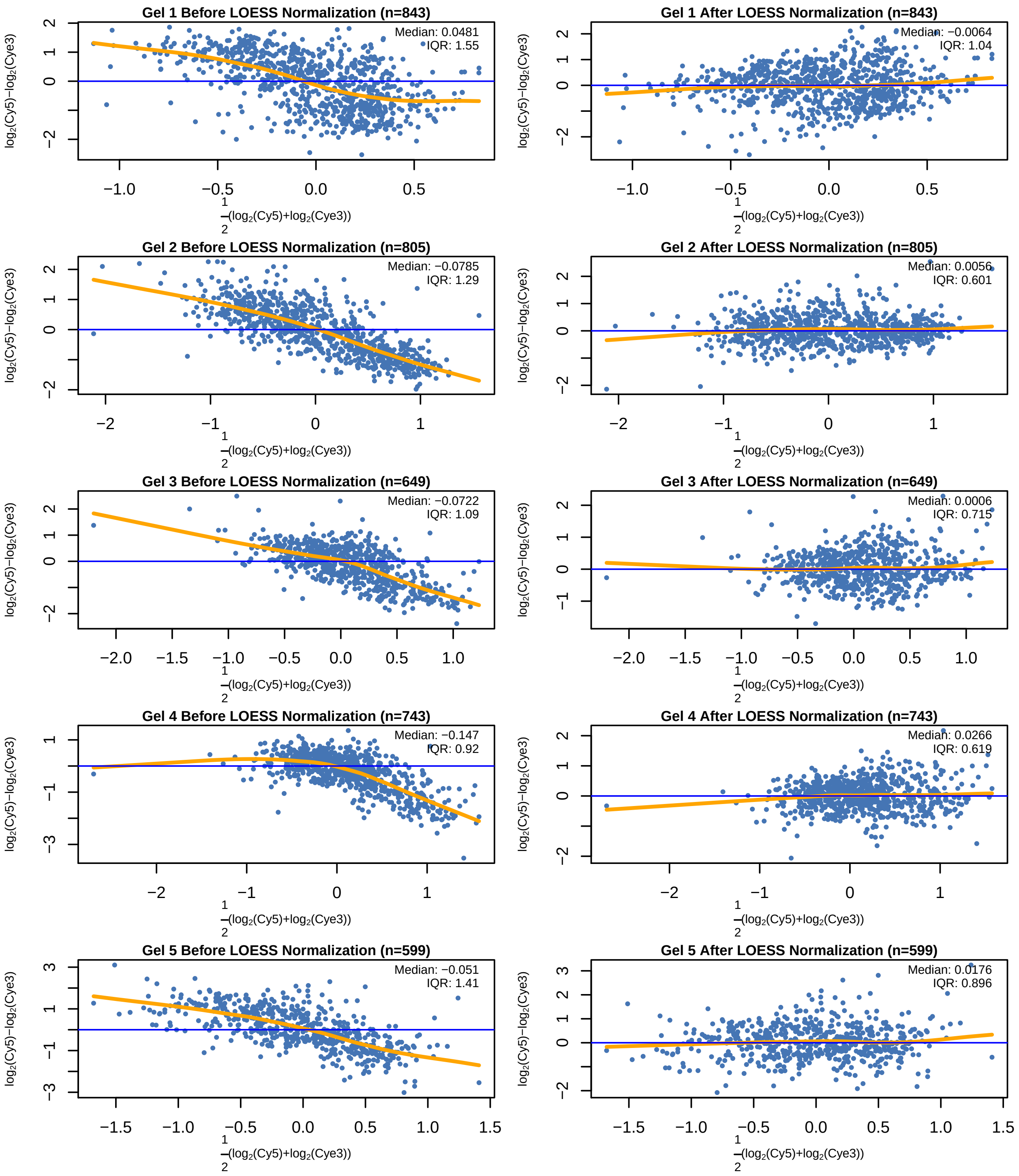

Figure S11: MA plots of Cy5 versus Cy3 before and after LOESS normalization (2D-DIGE/MS).

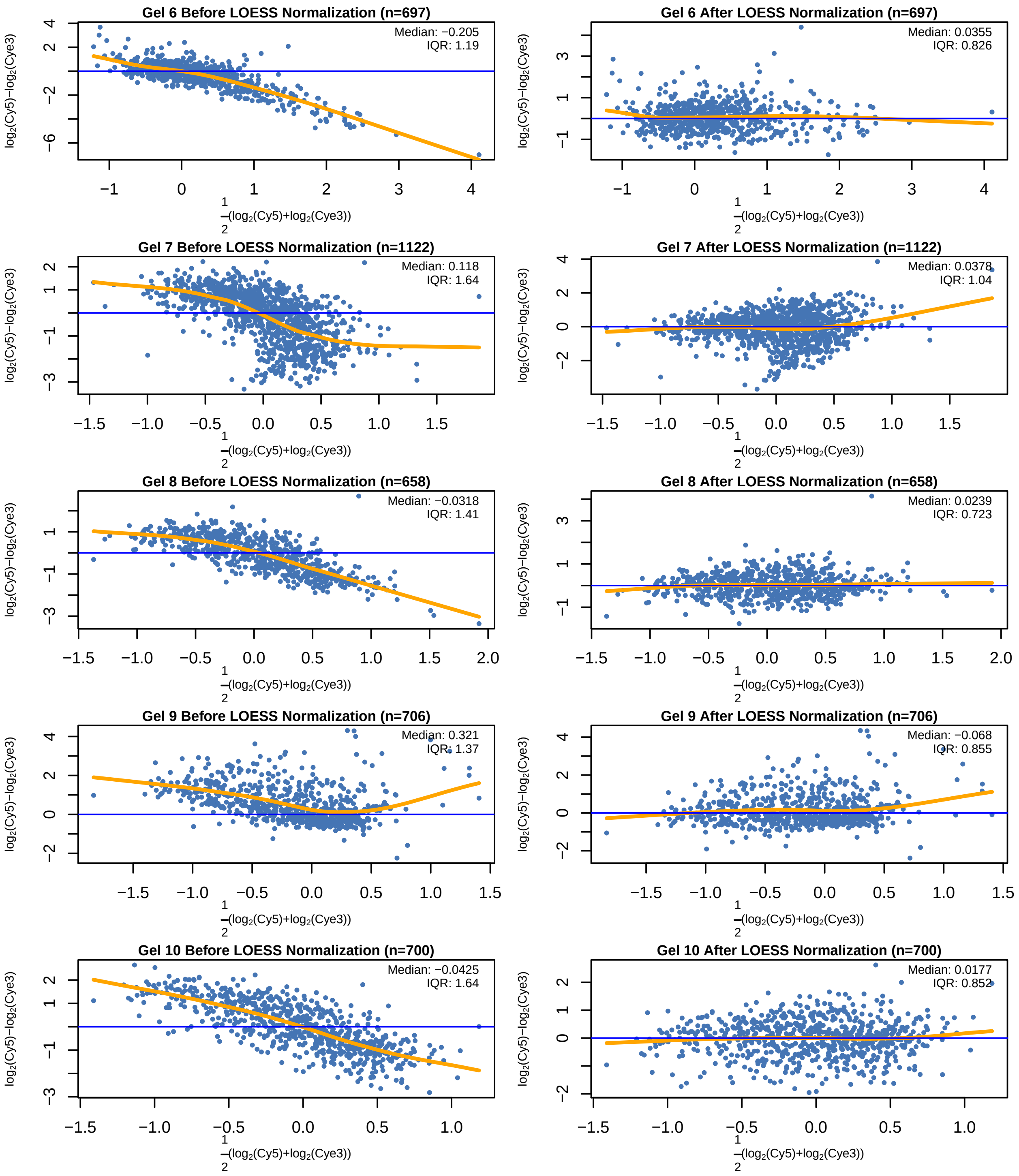

Figure S12: MA plots of Cy5 versus Cy3 before and after LOESS normalization (2D-DIGE/MS).

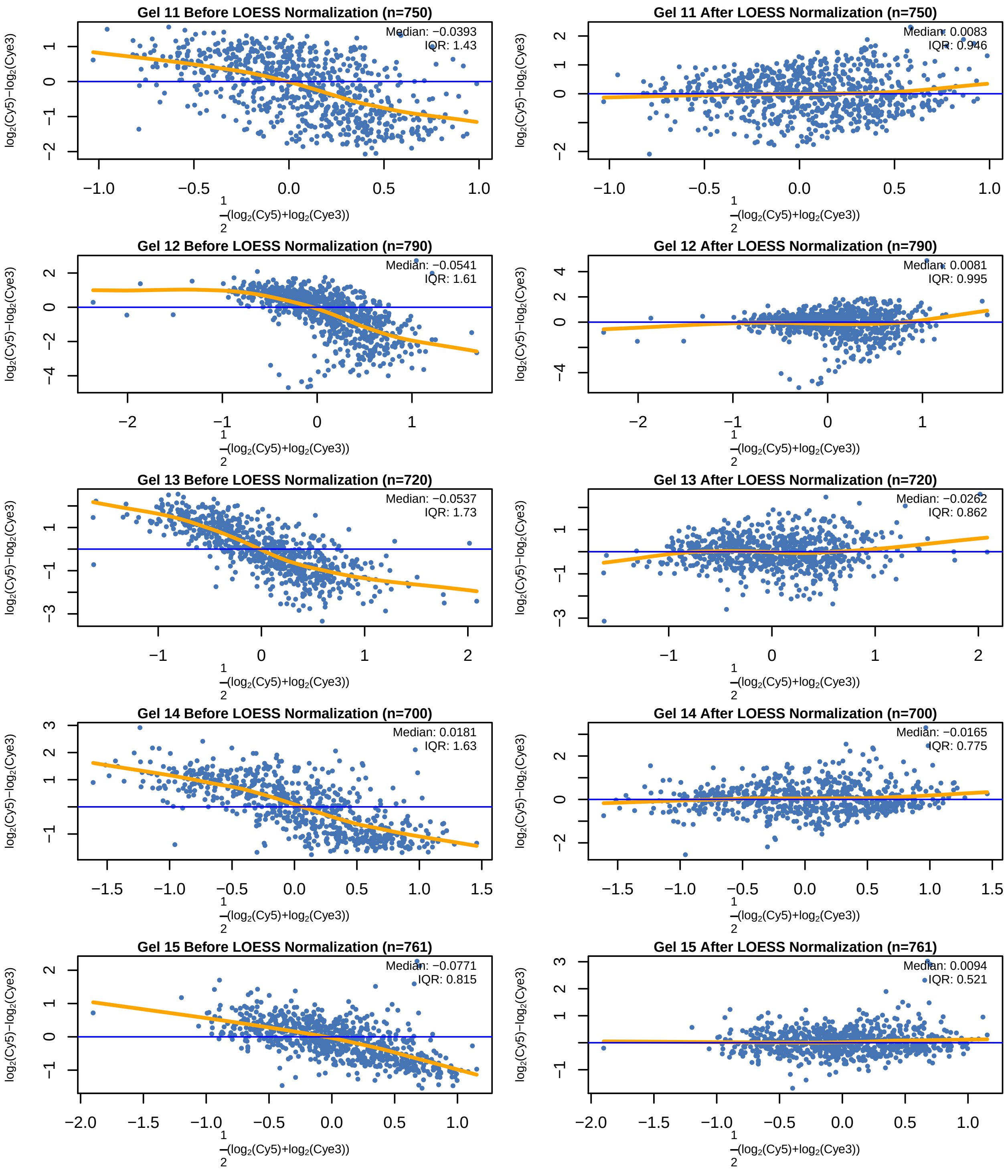

Figure S13: MA plots of Cy5 versus Cy3 before and after LOESS normalization (2D-DIGE/MS).

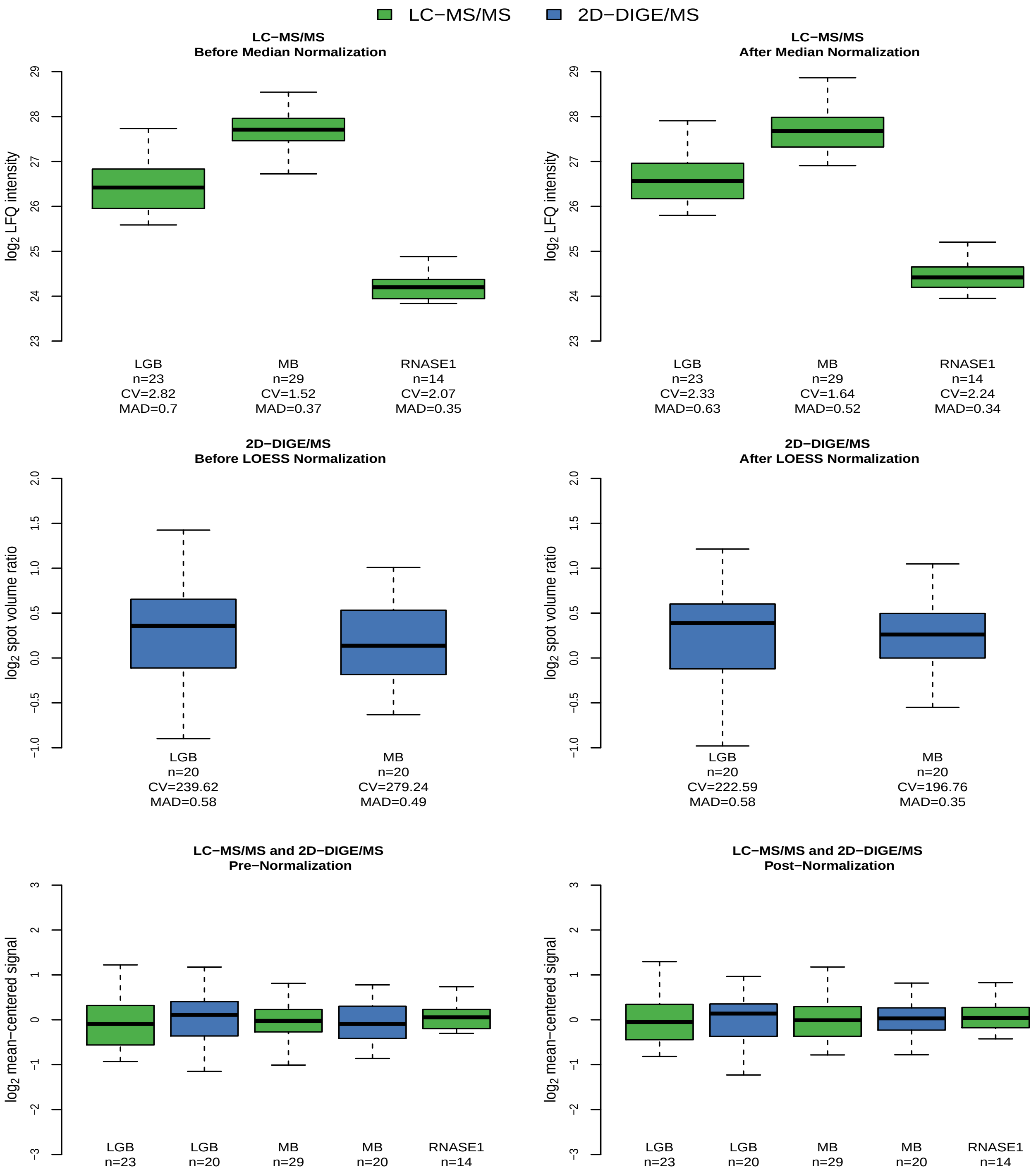

Figure S14: Boxplots of log_2 spike-in control protein signals and variability metrics (LC-MS/MS and 2D-DIGE/MS). Outliers are not shown to highlight shifts in center and scale. LGB: bovine beta-lactoglobulin protein, MB: horse myoglobin protein, and RNASE1: bovine ribonuclease pancreatic protein. n: number of non-missing observations, CV: coefficient of variation, MAD: median absolute deviation. The 2D-DIGE/MS master spot for the bovine pancreatic ribonuclease protein could not confidently be resolved.

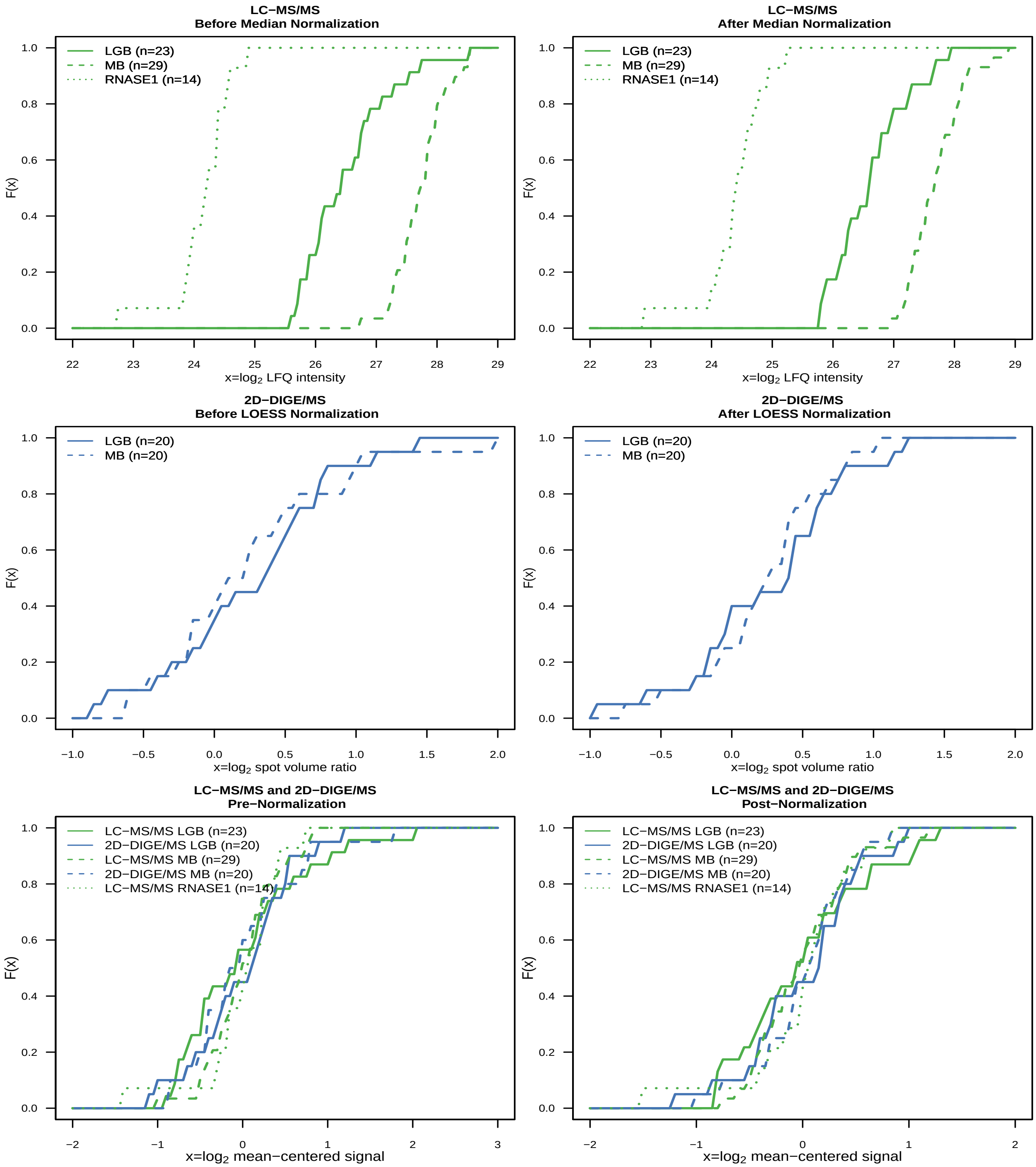

Figure S15: ECDF plots of log_2 spike-in protein signals (LC-MS/MS and 2D-DIGE/MS. LGB: bovine beta-lactoglobulin protein, MB: horse myoglobin protein, and RNASE1: bovine ribonuclease pancreatic protein. The 2D-DIGE/MS master spot for the bovine pancreatic ribonuclease protein could not confidently be resolved.

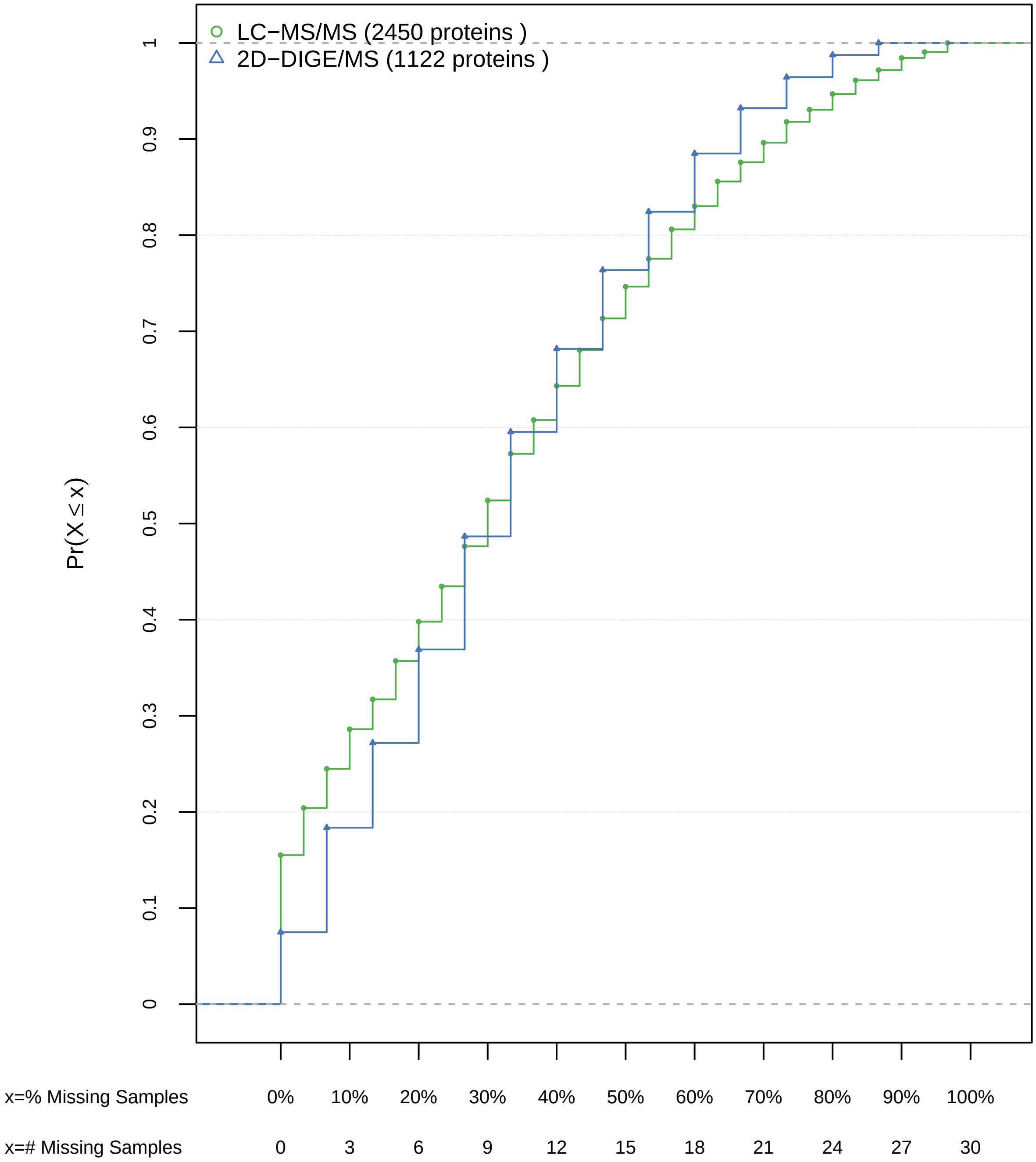

Figure S16: ECDF plots of missing protein observations across samples (LC-MS/MS and 2D-DIGE/MS, n=30).

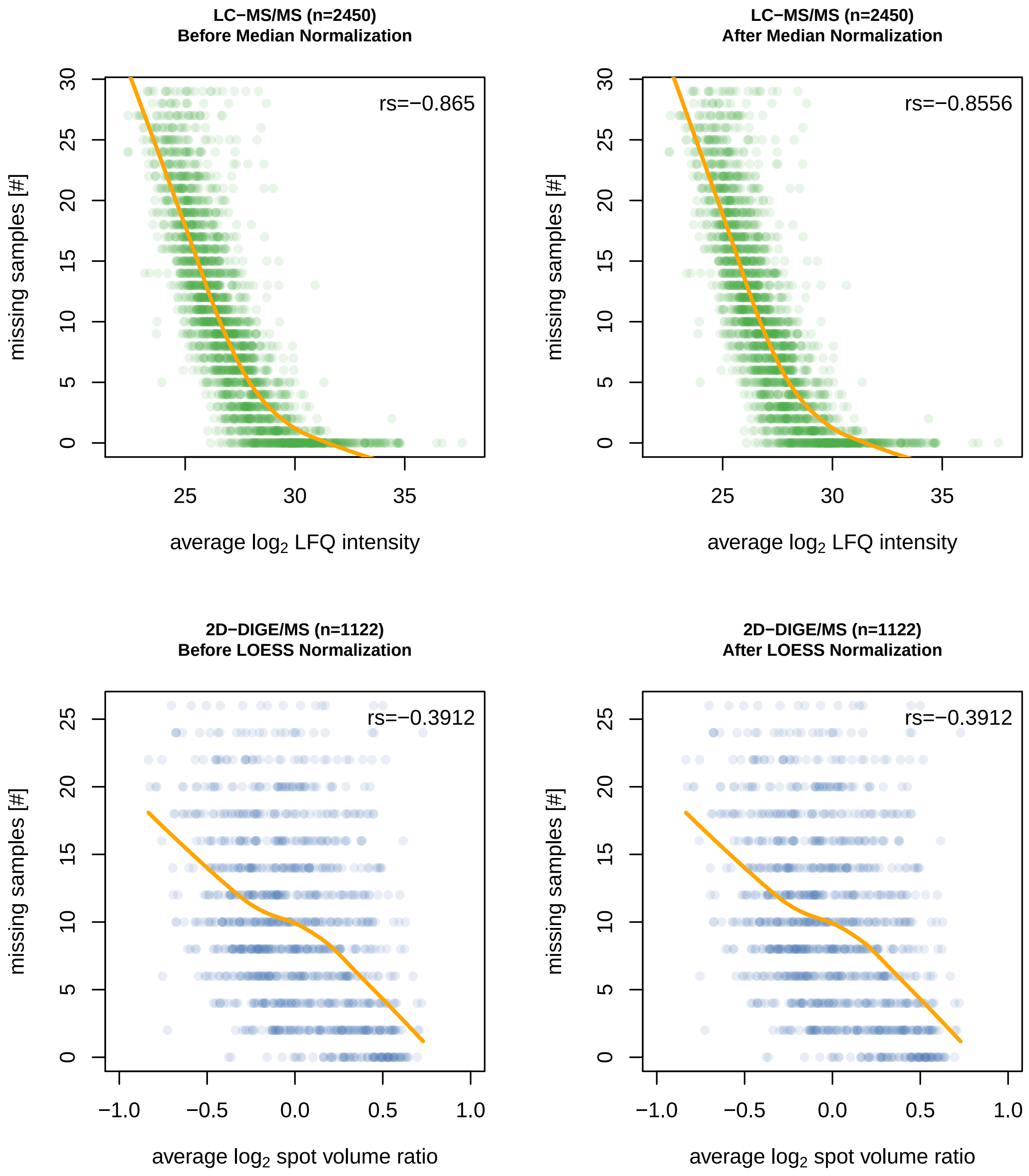

Figure S17: Scatterplots of missing observations by average log_2 protein signal (LC-MS/MS and 2D-DIGE/MS). The orange solid trend line represents a locally weighted regression fit. Spearman correlation coefficient is shown in the top right.

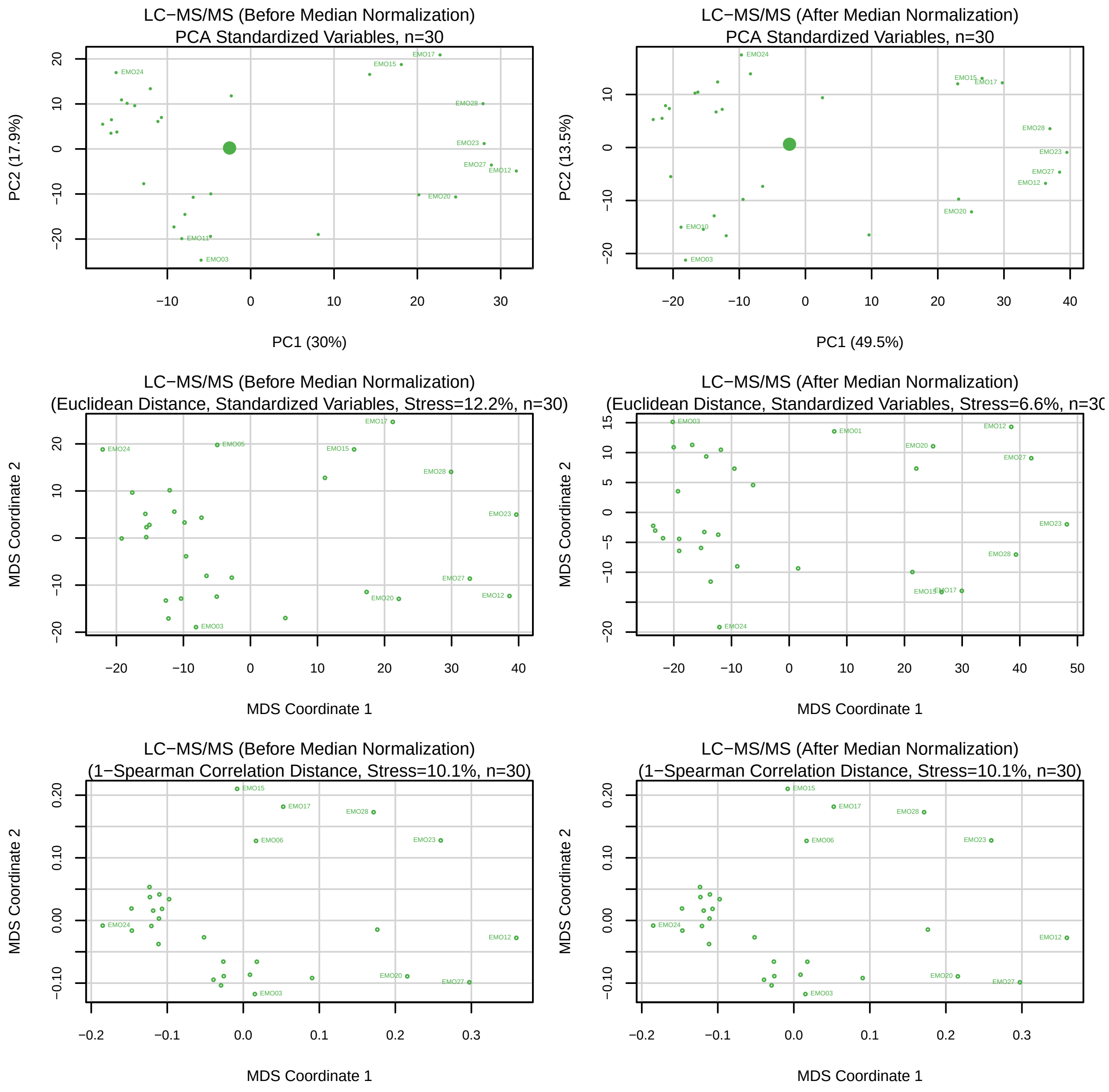

Figure S18: PCA and non-metric multidimensional scaling biplots (LC-MS/MS). PCA biplots with bivariate 95% confidence ellipses for standardized log_2 protein signals are shown at the top. Non-metric MDS results for standardized log_2 protein signals and pairwise differences based on Euclidean distance are shown in the middle. Non-metric MDS results based on 1-Spearman correlation distance between log_2 protein signals are shown at the bottom. Labels for the 10 most outlying samples based on maximum Mahalanobis distance are shown.

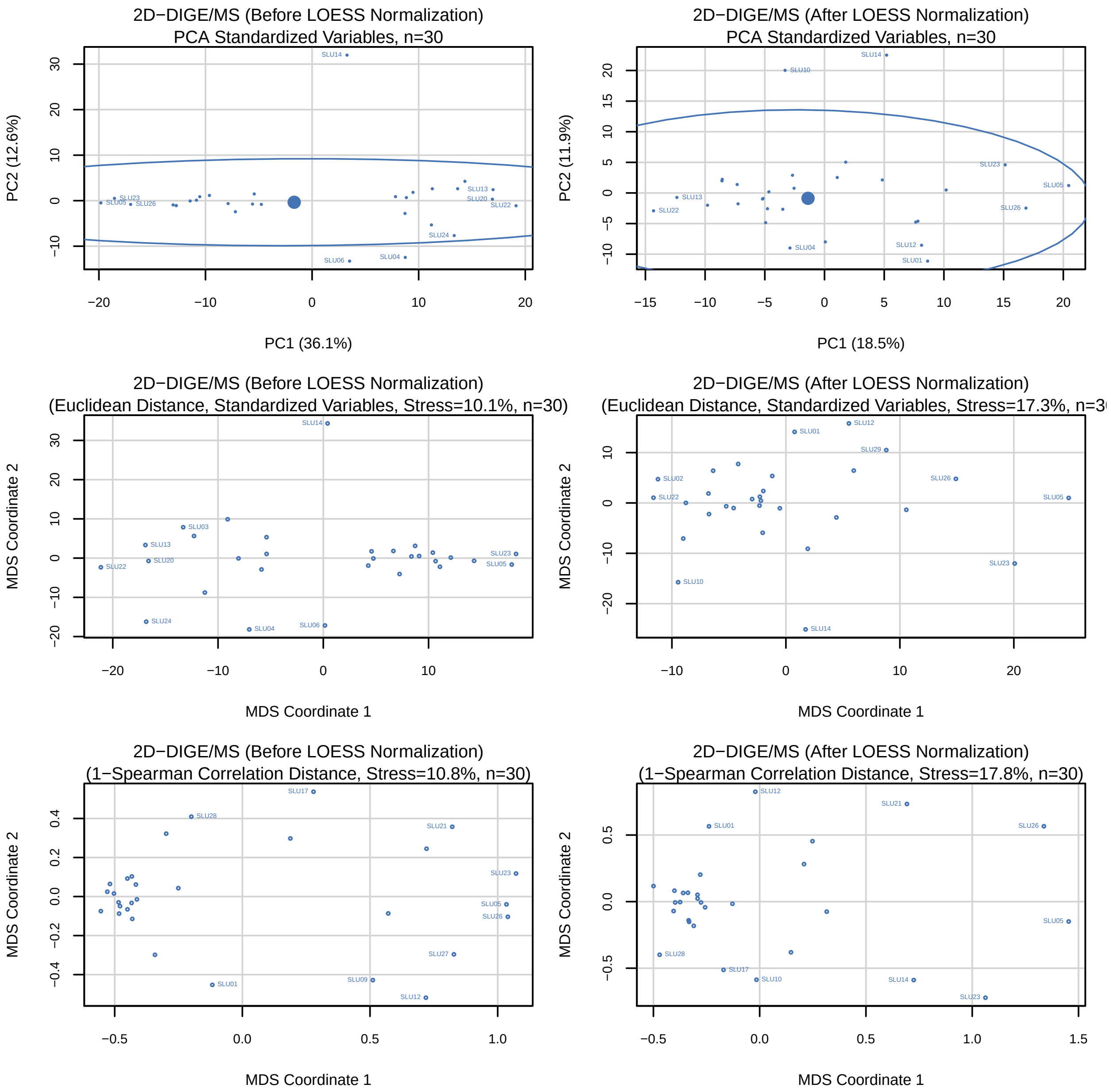

Figure S19: PCA and non-metric multidimensional scaling biplots (2D-DIGE/MS). PCA biplots with bivariate 95% confidence ellipses for standardized log_2 protein signals are shown at the top. Non-metric MDS results for standardized log_2 protein signals and pairwise differences based on Euclidean distance are shown in the middle. Non-metric MDS results based on 1-Spearman correlation distance between log_2 protein signals are shown at the bottom. Labels for the 10 most outlying samples based on maximum Mahalanobis distance are shown.

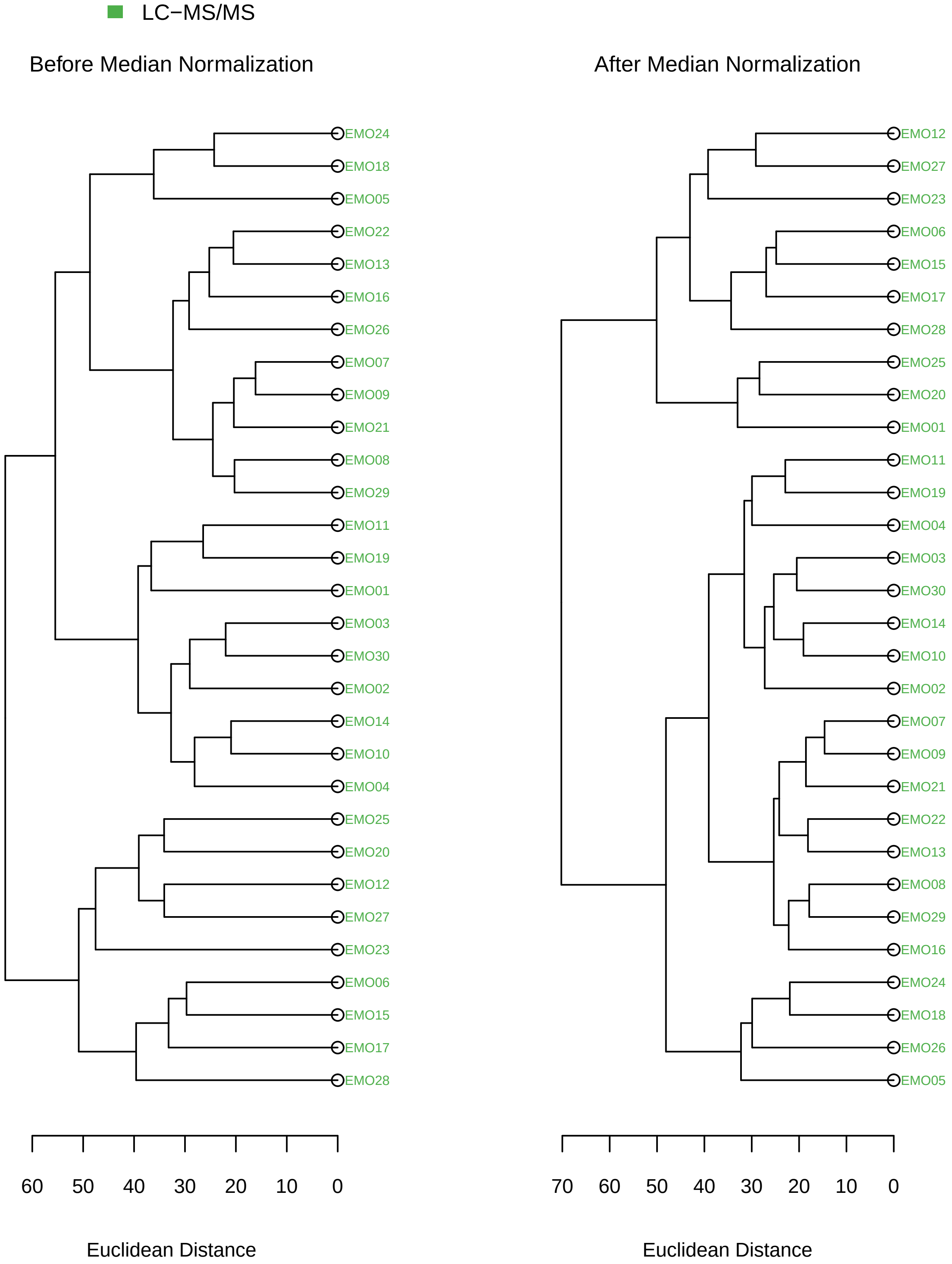

Figure S20: Proteomics-based Hierarchical Clustering Plots (LC-MS/MS). Euclidean distances between standardized log_2 protein signals hierarchically clustered using the complete linkage clustering algorithm.

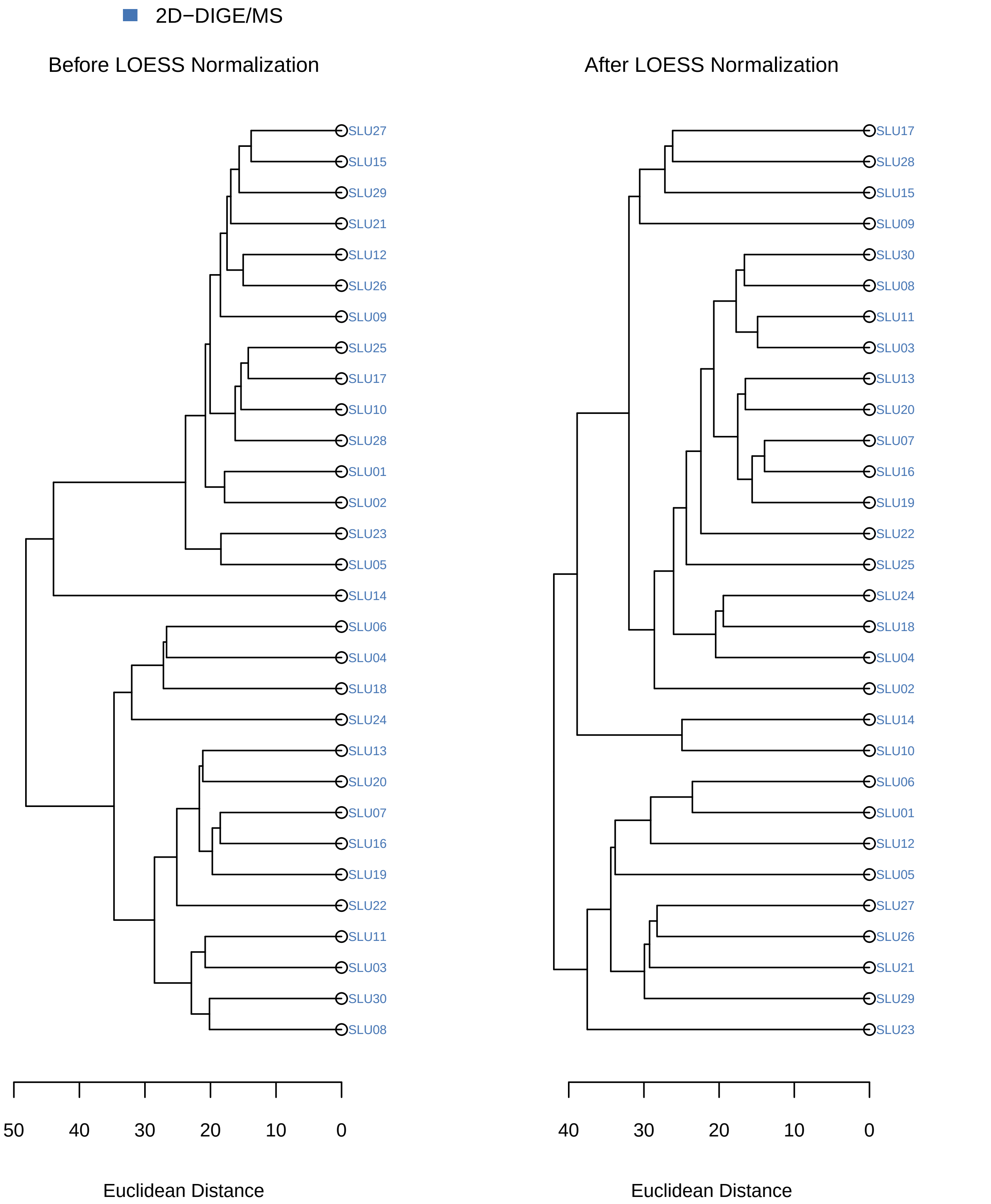

Figure S21: Proteomics-based Hierarchical Clustering Plots (2D-DIGE/MS). Euclidean distances between standardized log_2 protein signals hierarchically clustered using the complete linkage clustering algorithm.

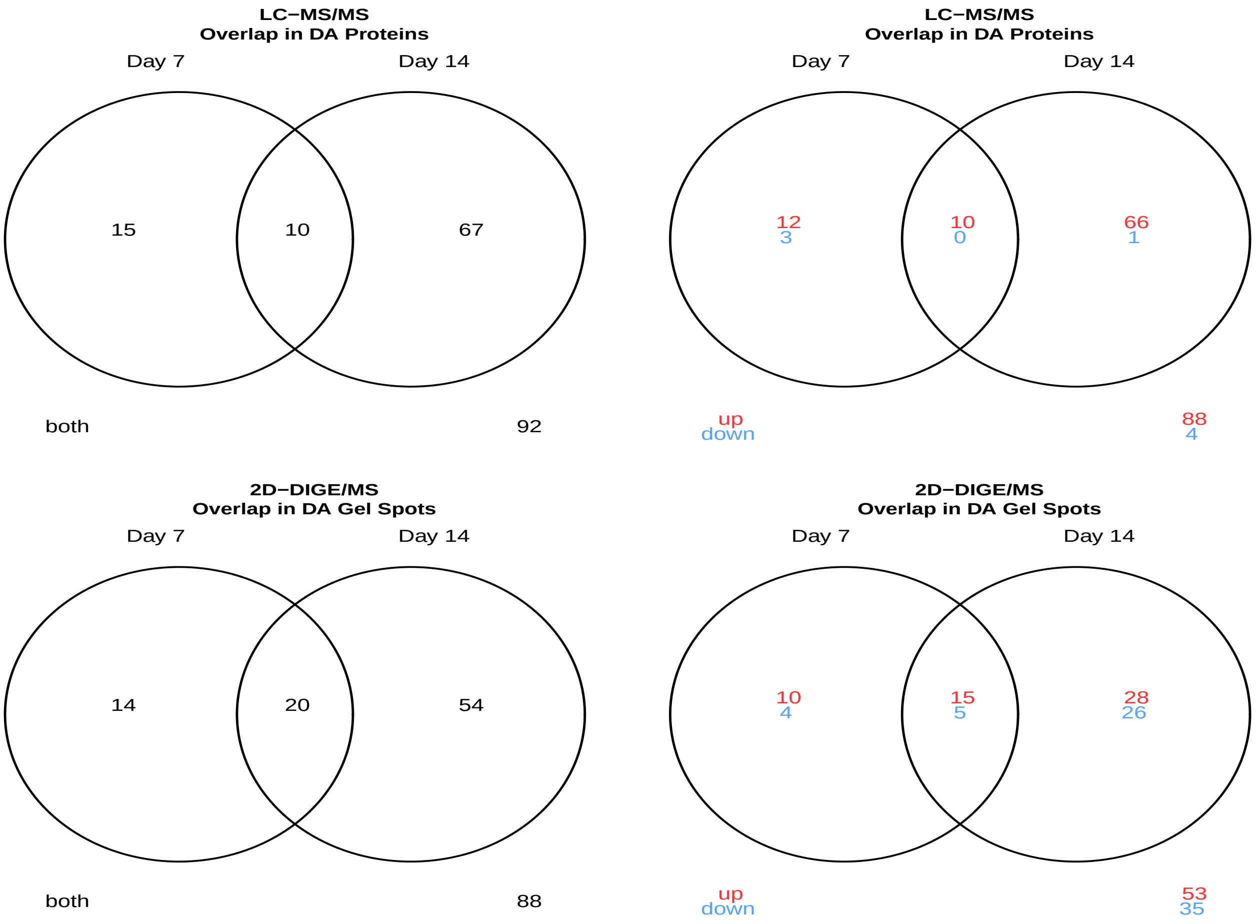

Figure S22: Venn diagrams summarizing overlap in DA proteins between post-vaccination days (LC-MS/MS and 2D-DIGE/MS). In red: increased compared to pre-vaccination, in blue: decreased compared to pre-vaccination.

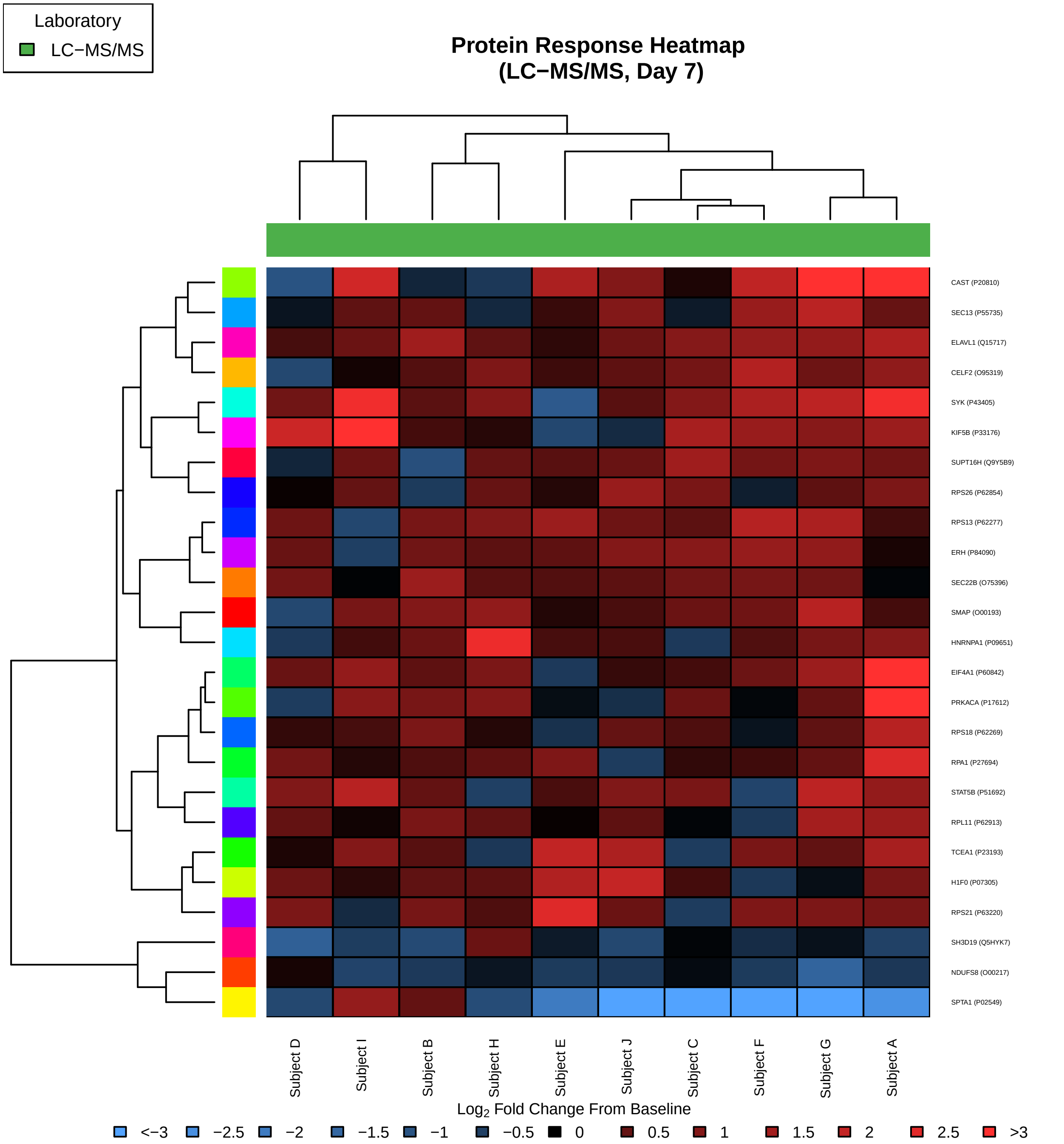

Figure S23: Heatmap of protein log_2 fold change from pre-vaccination (LC-MS/MS, , Day 7). Rows represent DA proteins. In red: proteins increased from baseline; in blue: proteins decreased from baseline. Dendrograms were obtained using complete linkage clustering of uncentered pairwise Pearson correlation distances between log_2 fold changes. Laboratory membership is highlighted below the subject dendrogram at the top. Protein family membership (50% sequence identity) is highlighted along the protein dendrogram on the left side.

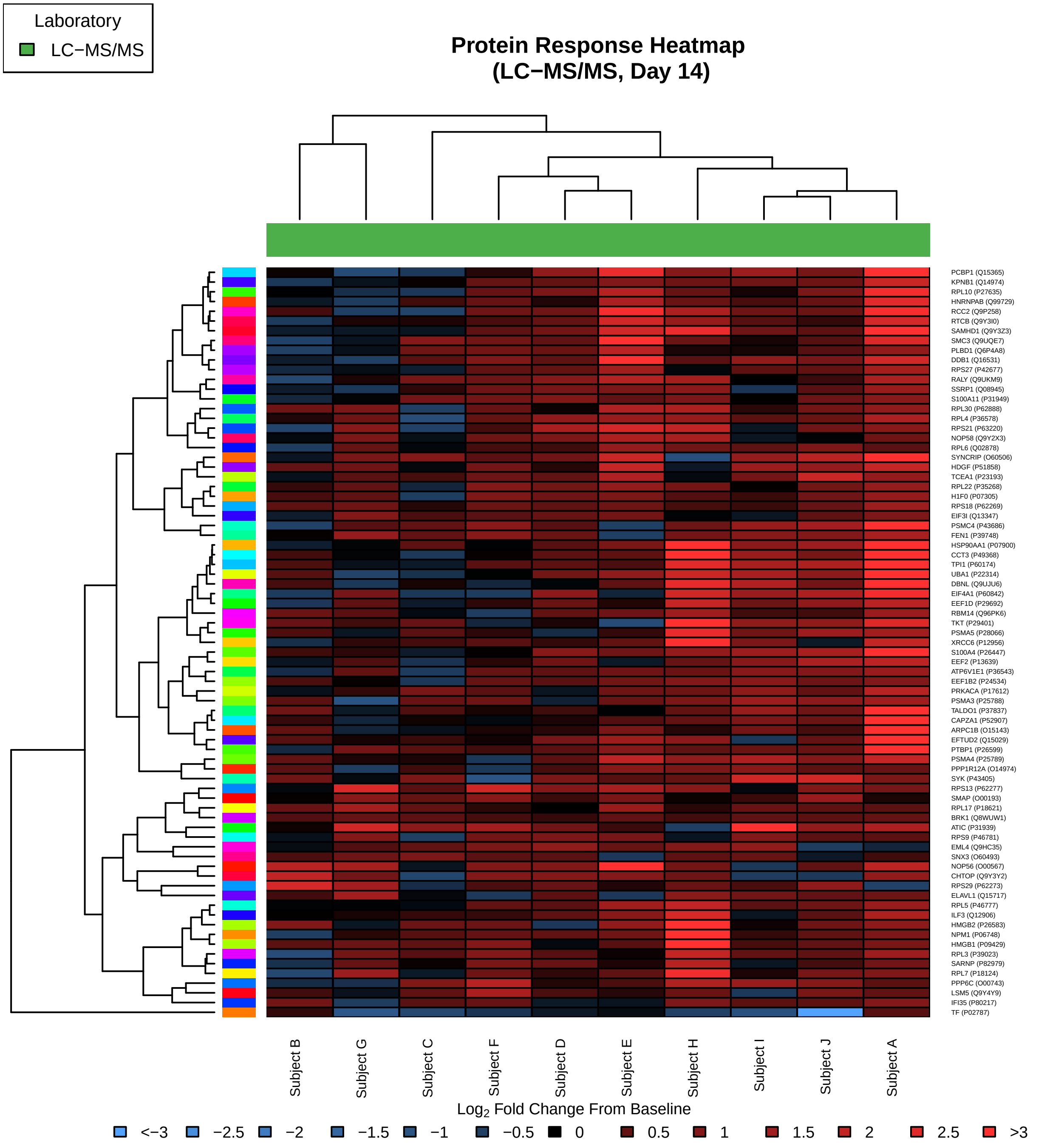

Figure S24: Heatmap of protein log_2 fold change from pre-vaccination (LC-MS/MS, , Day 14). Rows represent DA proteins. In red: proteins increased from baseline; in blue: proteins decreased from baseline. Dendrograms were obtained using complete linkage clustering of uncentered pairwise Pearson correlation distances between log_2 fold changes. Laboratory membership is highlighted below the subject dendrogram at the top. Protein family membership (50% sequence identity) is highlighted along the protein dendrogram on the left side.

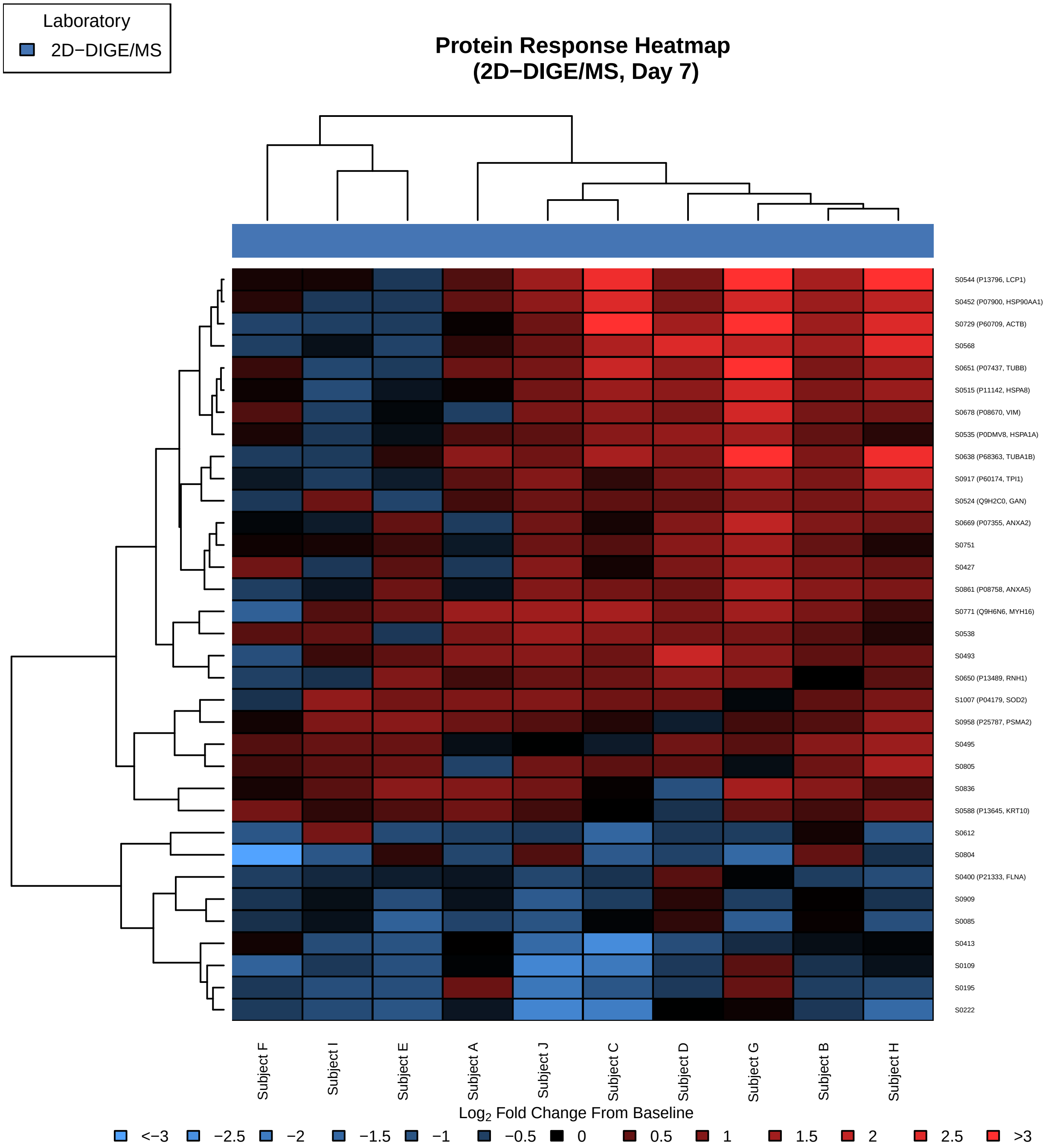

Figure S25: Heatmap of protein log_2 fold change from pre-vaccination (2D-DIGE/MS, , Day 7). Rows represent DA proteins. In red: proteins increased from baseline; in blue: proteins decreased from baseline. Dendrograms were obtained using complete linkage clustering of uncentered pairwise Pearson correlation distances between log_2 fold changes. Laboratory membership is highlighted below the subject dendrogram at the top.

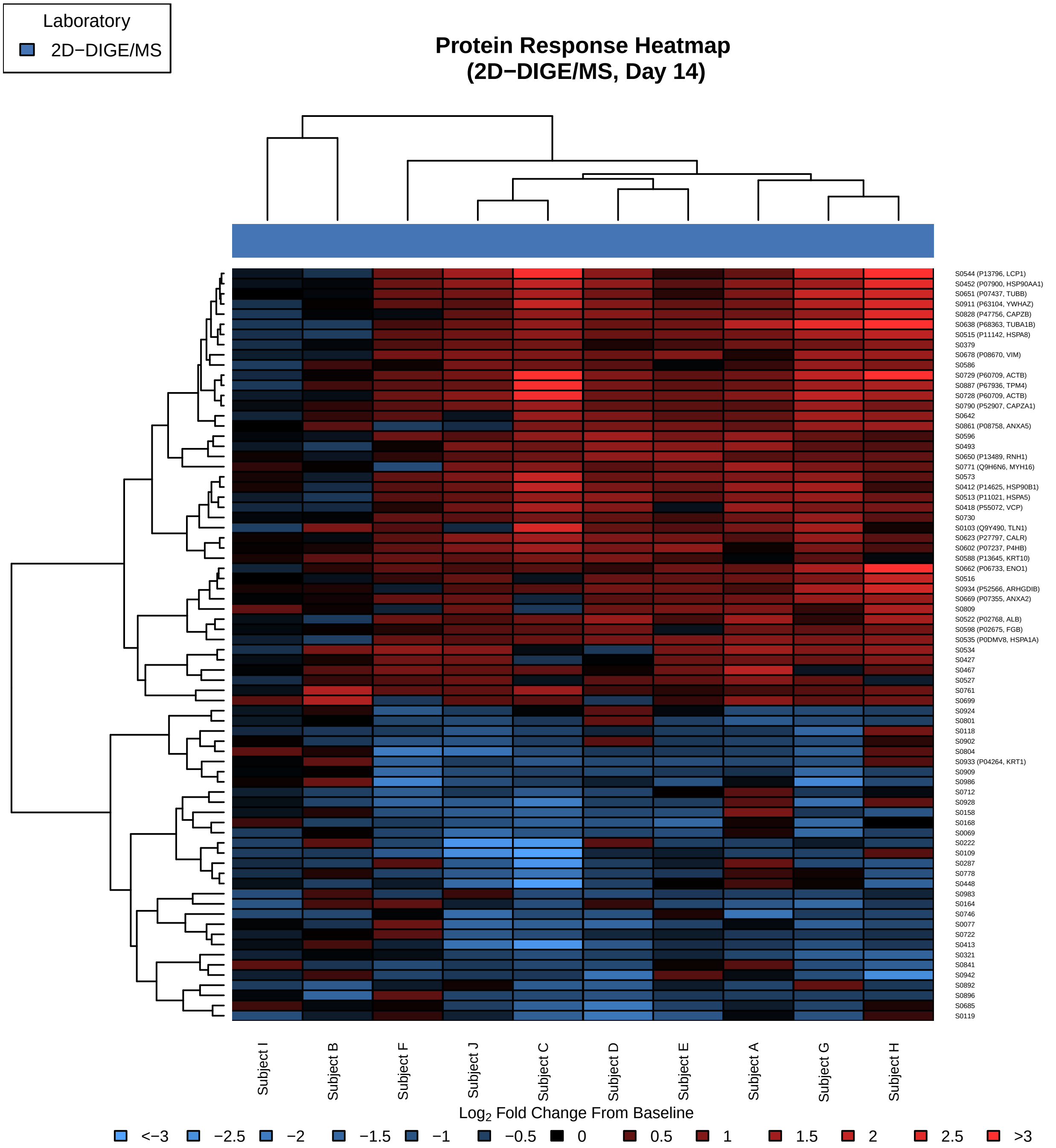

Figure S26: Heatmap of protein log_2 fold change from pre-vaccination (2D-DIGE/MS, , Day 14). Rows represent DA proteins. In red: proteins increased from baseline; in blue: proteins decreased from baseline. Dendrograms were obtained using complete linkage clustering of uncentered pairwise Pearson correlation distances between log_2 fold changes. Laboratory membership is highlighted below the subject dendrogram at the top.

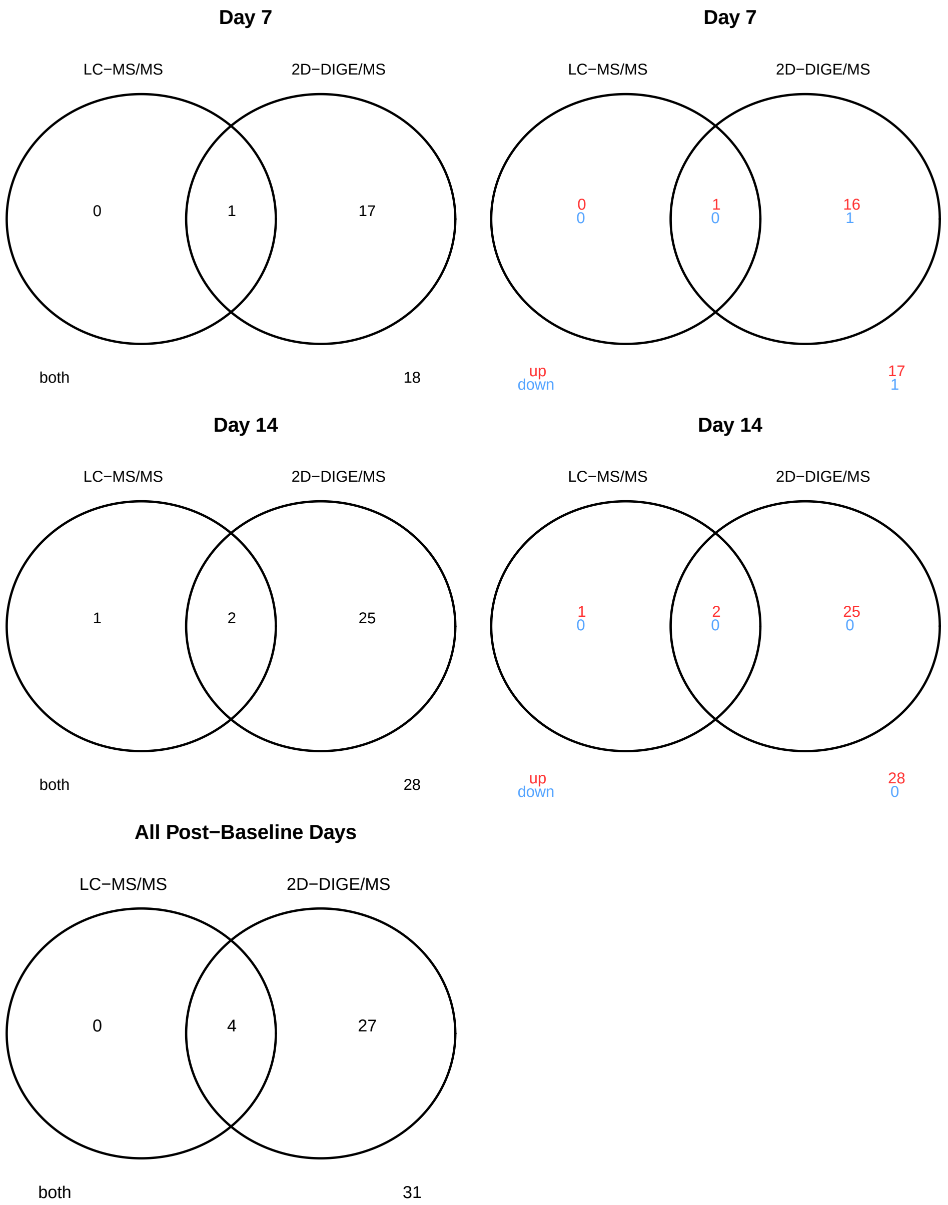

Figure S27: Venn diagrams summarizing overlap in DA proteins among 35 proteins with shared identifications for both laboratories (LC-MS/MS and 2D-DIGE/MS). In red: increased compared to pre-vaccination, in blue: decreased compared to pre-vaccination.

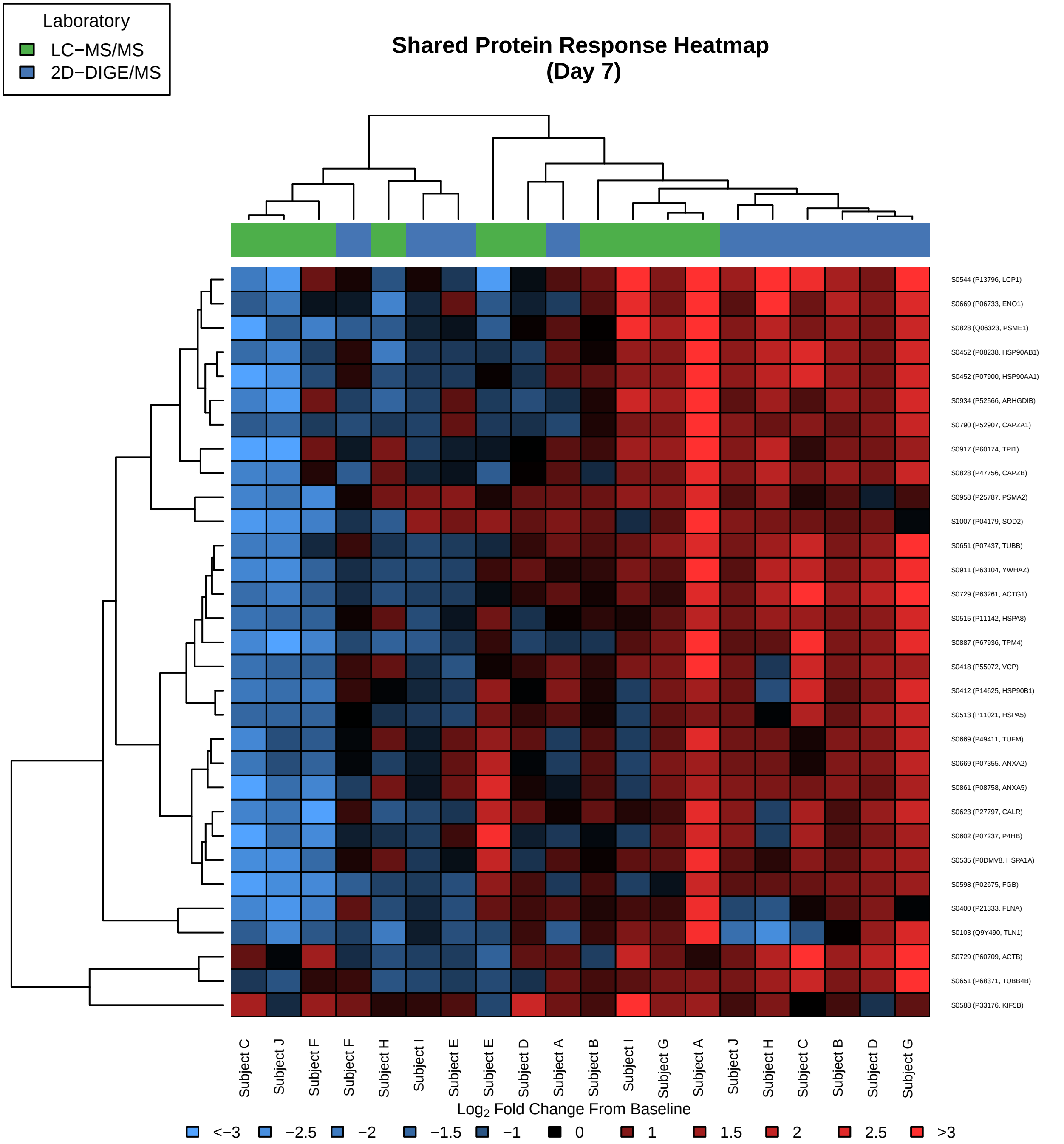

Figure S28: Heatmap of protein log_2 fold change for shared DA proteins (LC-MS/MS and 2D-DIGE/MS, Day 7). Rows represent 31 shared proteins that were DA at any post-vaccination day for any of the two laboratories. In red: proteins increased from pre-vaccination; in blue: proteins decreased from pre-vaccination. Dendrograms were obtained using complete linkage clustering of uncentered pairwise Pearson correlation distances between log_2 fold changes. Laboratory membership is highlighted below the subject dendrogram at the top.

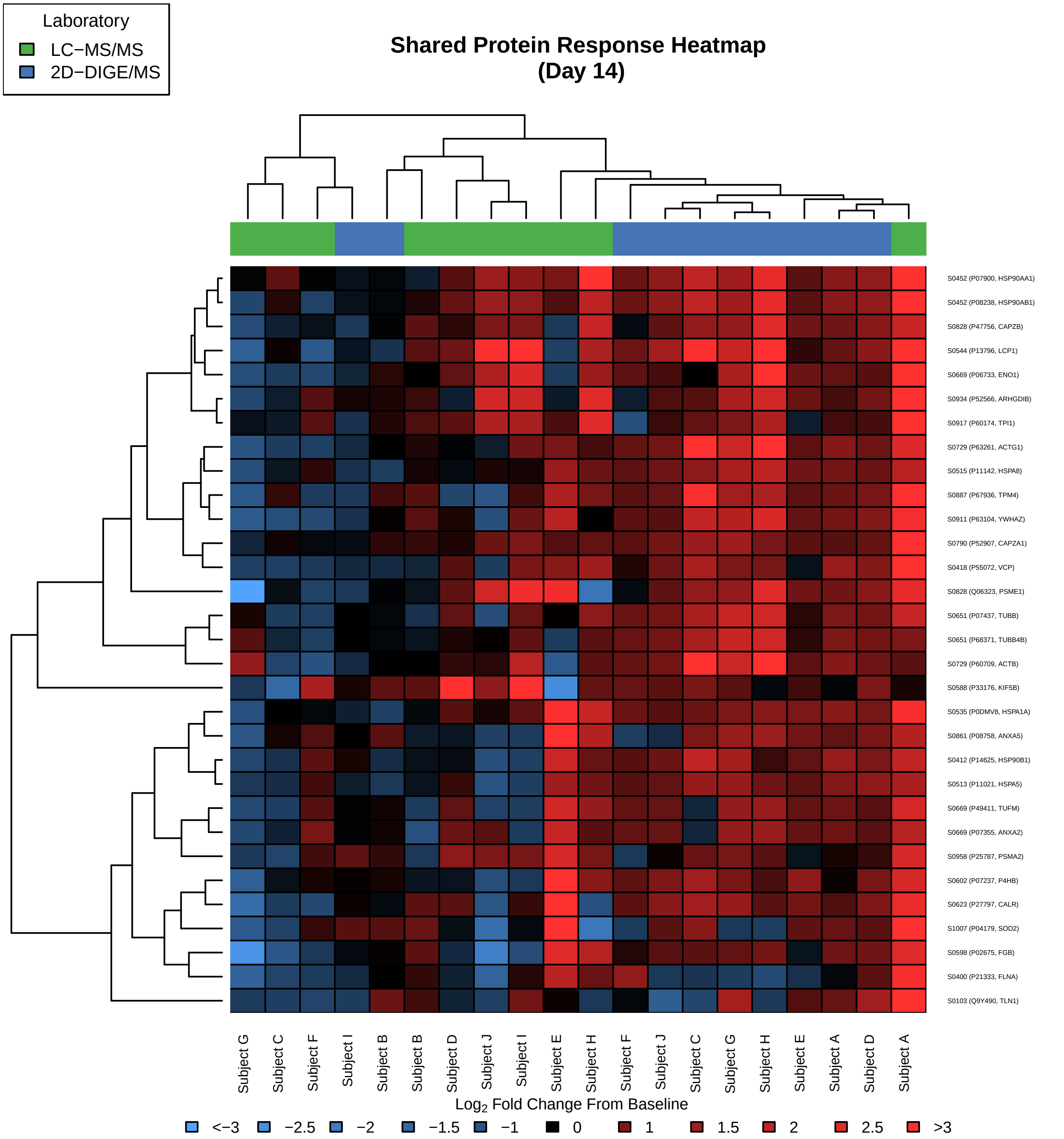

Figure S29: Heatmap of protein log_2 fold change for shared DA proteins (LC-MS/MS and 2D-DIGE/MS, Day 14). Rows represent 31 shared proteins that were DA at any post-vaccination day for any of the two laboratories. In red: proteins increased from pre-vaccination; in blue: proteins decreased from pre-vaccination. Dendrograms were obtained using complete linkage clustering of uncentered pairwise Pearson correlation distances between log_2 fold changes. Laboratory membership is highlighted below the subject dendrogram at the top.

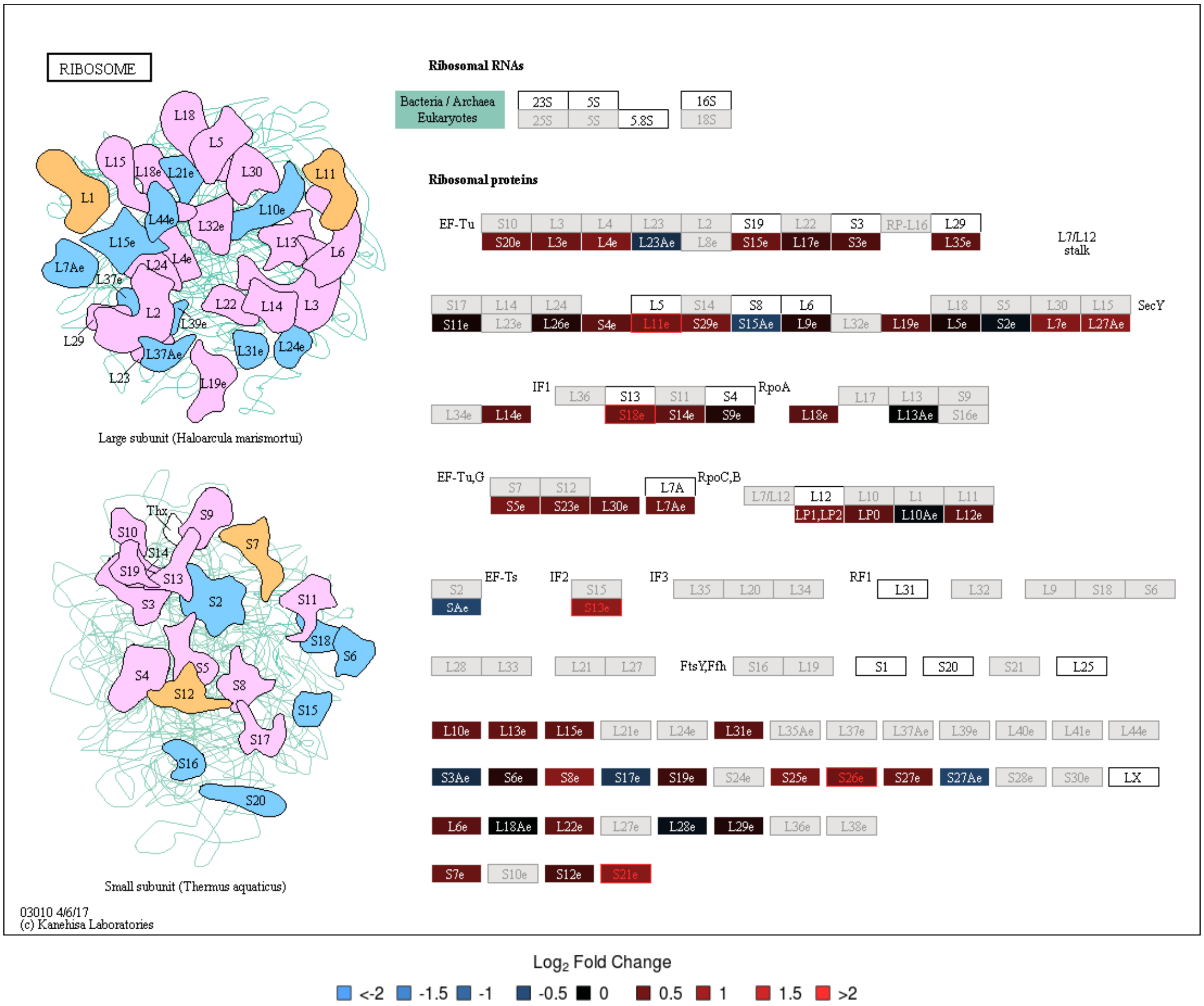

Figure S30: KEGG Pathway Map - Ribosome - Homo sapiens (human) (LC-MS/MS, Day 7). Node color gradient encodes fold change from pre-vaccination (for multi-gene nodes the median fold change of corresponding proteins is used). In red: increased compared to pre-vaccination, in blue: decreased compared to pre-vaccination, in black: fold change close to 1, in dark grey: gene encoding for protein that was not experimentally identified or had more than 20% missing observations, light grey: gene missing database mapping, white: non-human gene. Genes encoding for DA proteins are highlighted using red (significantly increased) and blue (significantly decreased) node label and border colors.

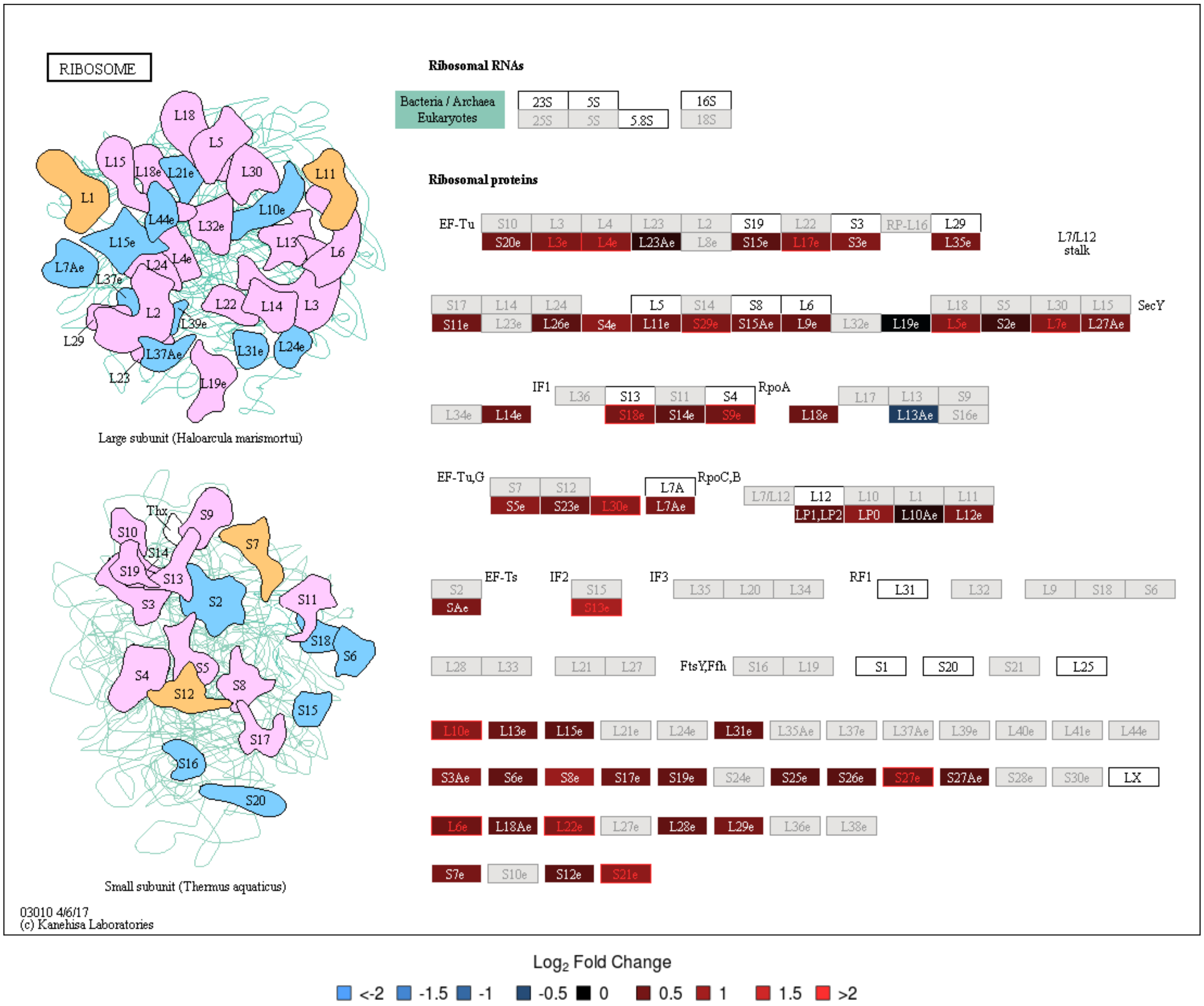

Figure S31: KEGG Pathway Map - Ribosome - Homo sapiens (human) (LC-MS/MS, Day 14). Node color gradient encodes fold change from pre-vaccination (for multi-gene nodes the median fold change of corresponding proteins is used). In red: increased compared to pre-vaccination, in blue: decreased compared to pre-vaccination, in black: fold change close to 1, in dark grey: gene encoding for protein that was not experimentally identified or had more than 20% missing observations, light grey: gene missing database mapping, white: non-human gene. Genes encoding for DA proteins are highlighted using red (significantly increased) and blue (significantly decreased) node label and border colors.

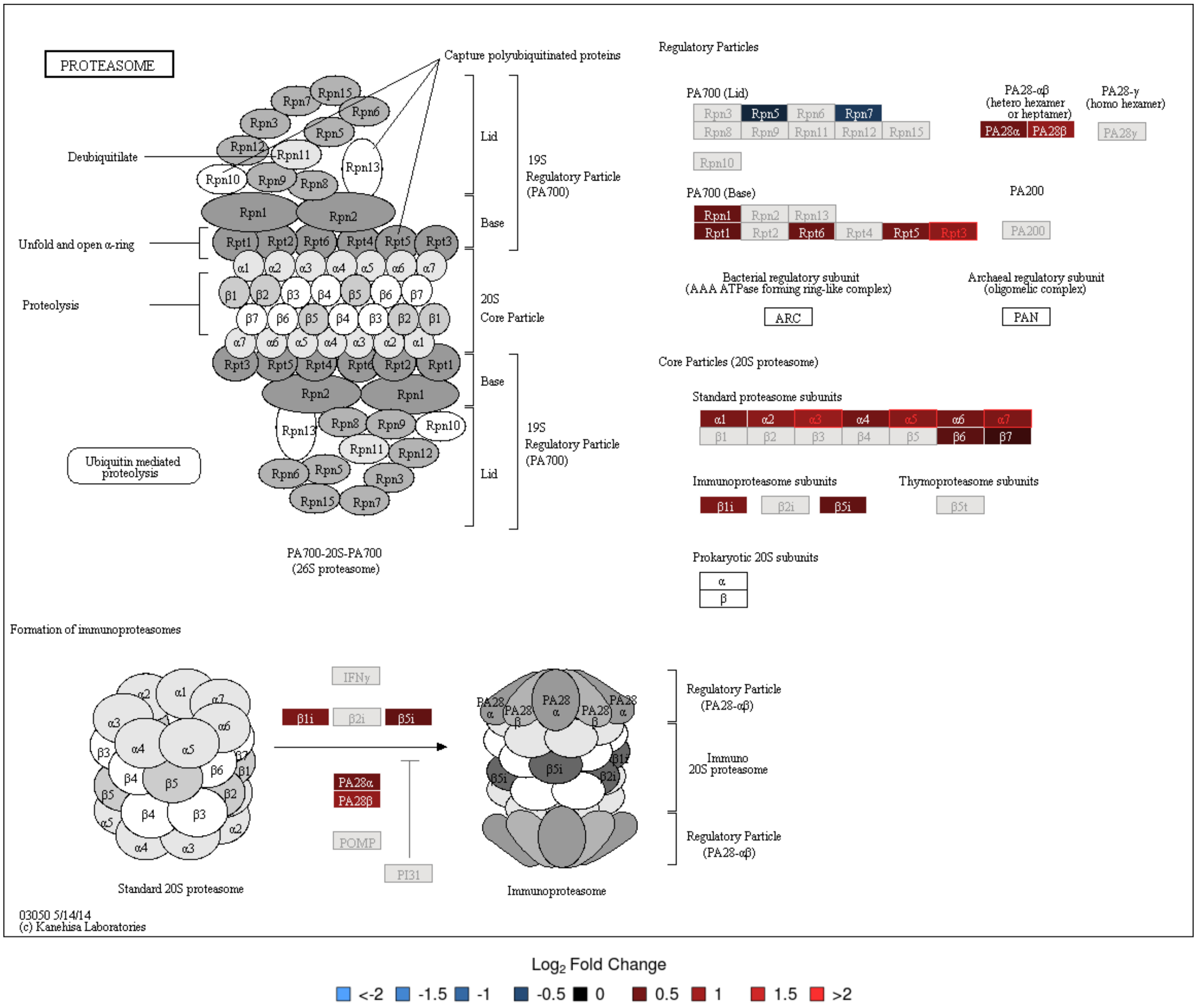

Figure S32: KEGG Pathway Map - Proteasome - Homo sapiens (human) (LC-MS/MS, Day 14). Node color gradient encodes fold change from pre-vaccination (for multi-gene nodes the median fold change of corresponding proteins is used). In red: increased compared to pre-vaccination, in blue: decreased compared to pre-vaccination, in black: fold change close to 1, in dark grey: gene encoding for protein that was not experimentally identified or had more than 20% missing observations, light grey: gene missing database mapping, white: non-human gene. Genes encoding for DA proteins are highlighted using red (significantly increased) and blue (significantly decreased) node label and border colors.

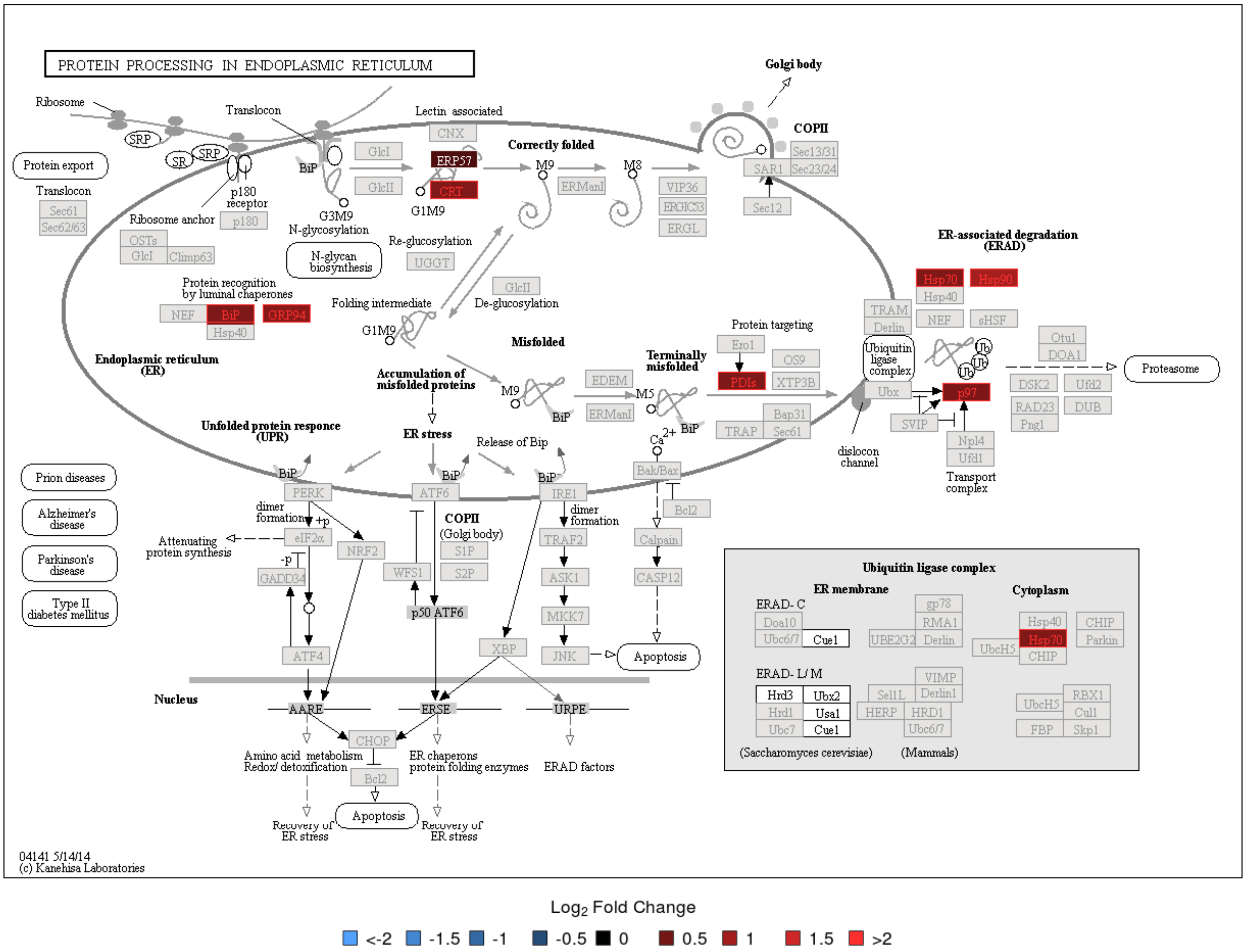

Figure S33: KEGG Pathway Map - Protein processing in endoplasmic reticulum - Homo sapiens (human) (2D-DIGE/MS, Day 14). Node color gradient encodes fold change from pre-vaccination (for multi-gene nodes the median fold change of corresponding proteins is used). In red: increased compared to pre-vaccination, in blue: decreased compared to pre-vaccination, in black: fold change close to 1, in dark grey: gene encoding for protein that was not experimentally identified or had more than 20% missing observations, light grey: gene missing database mapping, white: non-human gene. Genes encoding for DA proteins are highlighted using red (significantly increased) and blue (significantly decreased) node label and border colors.

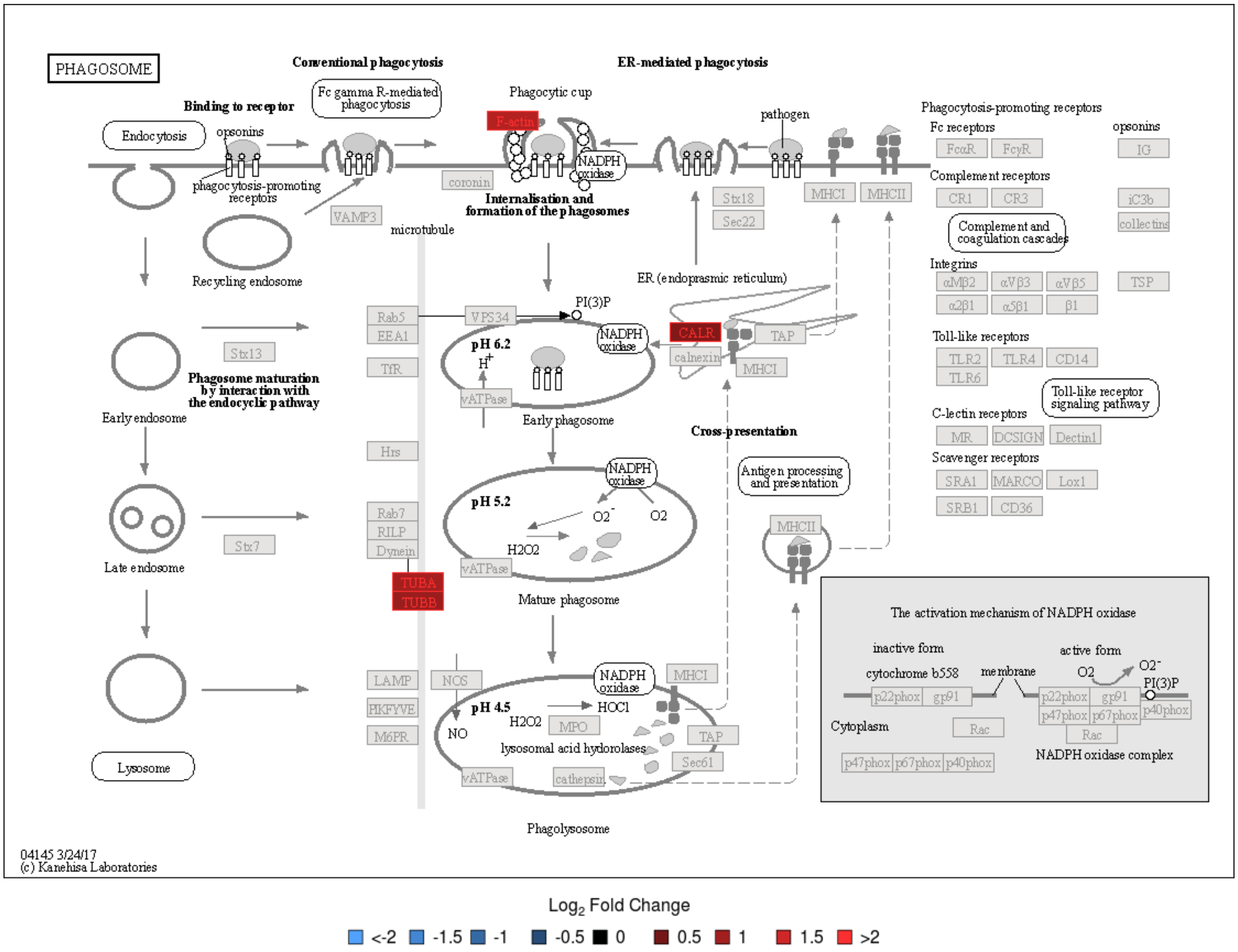

Figure S34: KEGG Pathway Map - Phagosome - Homo sapiens (human) (2D-DIGE/MS, Day 14). Node color gradient encodes fold change from pre-vaccination (for multi-gene nodes the median fold change of corresponding proteins is used). In red: increased compared to pre-vaccination, in blue: decreased compared to pre-vaccination, in black: fold change close to 1, in dark grey: gene encoding for protein that was not experimentally identified or had more than 20% missing observations, light grey: gene missing database mapping, white: non-human gene. Genes encoding for DA proteins are highlighted using red (significantly increased) and blue (significantly decreased) node label and border colors.

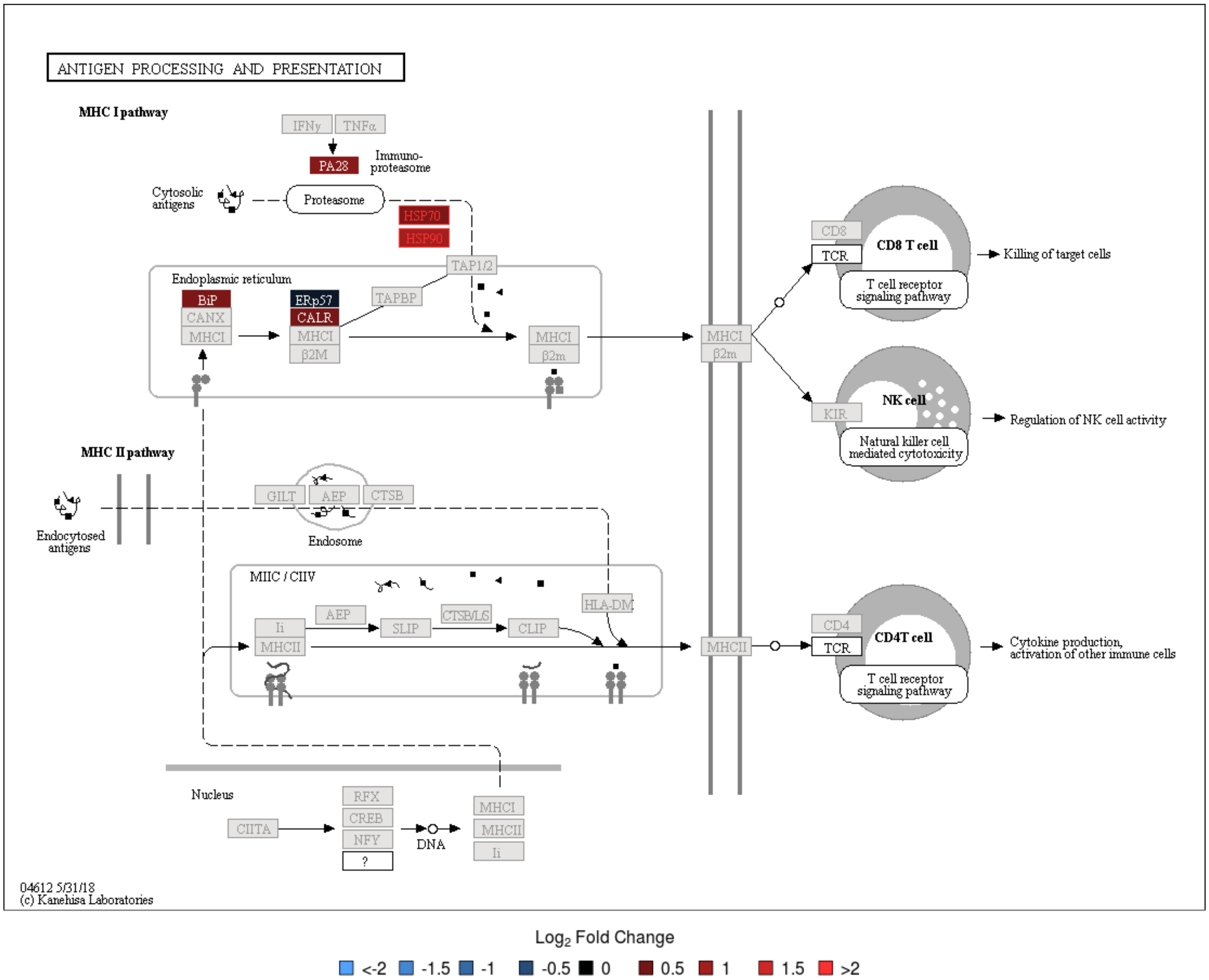

Figure S35: KEGG Pathway Map - Antigen processing and presentation - Homo sapiens (human) (2D-DIGE/MS, Day 7). Node color gradient encodes fold change from pre-vaccination (for multi-gene nodes the median fold change of corresponding proteins is used). In red: increased compared to pre-vaccination, in blue: decreased compared to pre-vaccination, in black: fold change close to 1, in dark grey: gene encoding for protein that was not experimentally identified or had more than 20% missing observations, light grey: gene missing database mapping, white: non-human gene. Genes encoding for DA proteins are highlighted using red (significantly increased) and blue (significantly decreased) node label and border colors.

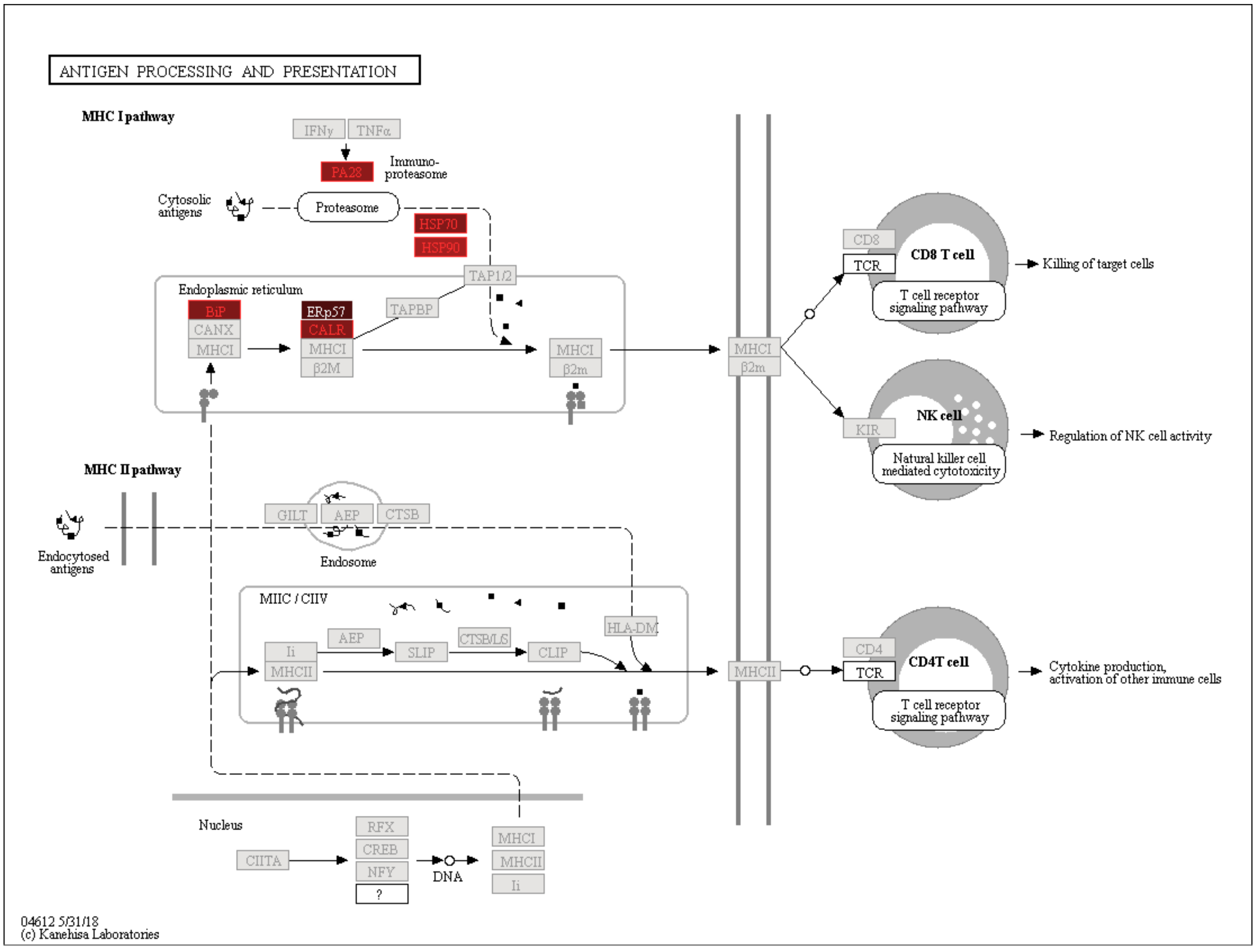

Log$_2$ Fold Change

<-2 -1.5 -1 -0.5 0 0.5 1 1.5 >2

Figure S36: KEGG Pathway Map - Antigen processing and presentation - Homo sapiens (human) (2D-DIGE/MS, Day 14). Node color gradient encodes fold change from pre-vaccination (for multi-gene nodes the median fold change of corresponding proteins is used). In red: increased compared to pre-vaccination, in blue: decreased compared to pre-vaccination, in black: fold change close to 1, in dark grey: gene encoding for protein that was not experimentally identified or had more than 20% missing observations, light grey: gene missing database mapping, white: non-human gene. Genes encoding for DA proteins are highlighted using red (significantly increased) and blue (significantly decreased) node label and border colors.

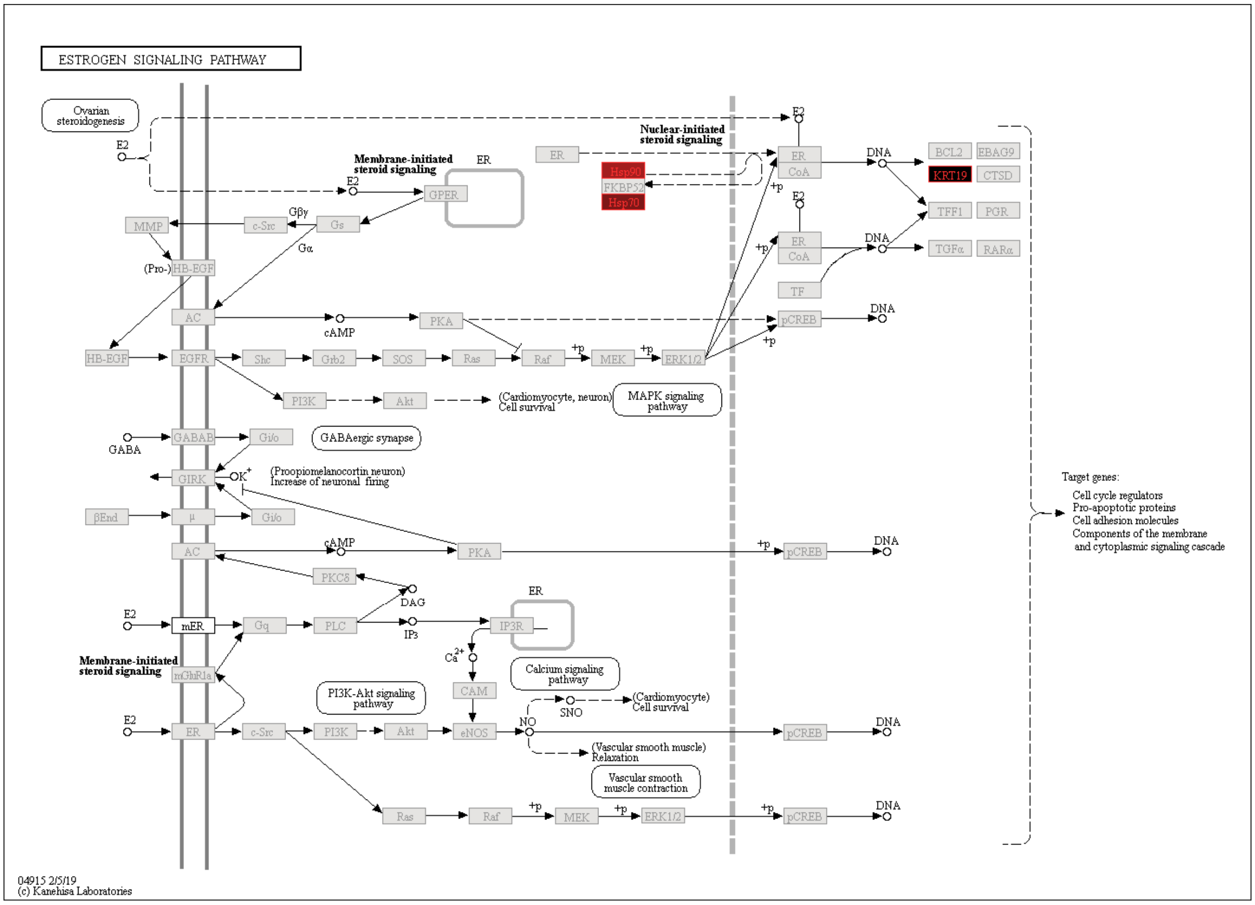

Log$_2$ Fold Change

<-2 -1.5 -1 -0.5 0 0.5 1 1.5 >2

Figure S37: KEGG Pathway Map - Estrogen signaling pathway - Homo sapiens (human) (2D-DIGE/MS, Day 7). Node color gradient encodes fold change from pre-vaccination (for multi-gene nodes the median fold change of corresponding proteins is used). In red: increased compared to pre-vaccination, in blue: decreased compared to pre-vaccination, in black: fold change close to 1, in dark grey: gene encoding for protein that was not experimentally identified or had more than 20% missing observations, light grey: gene missing database mapping, white: non-human gene. Genes encoding for DA proteins are highlighted using red (significantly increased) and blue (significantly decreased) node label and border colors.

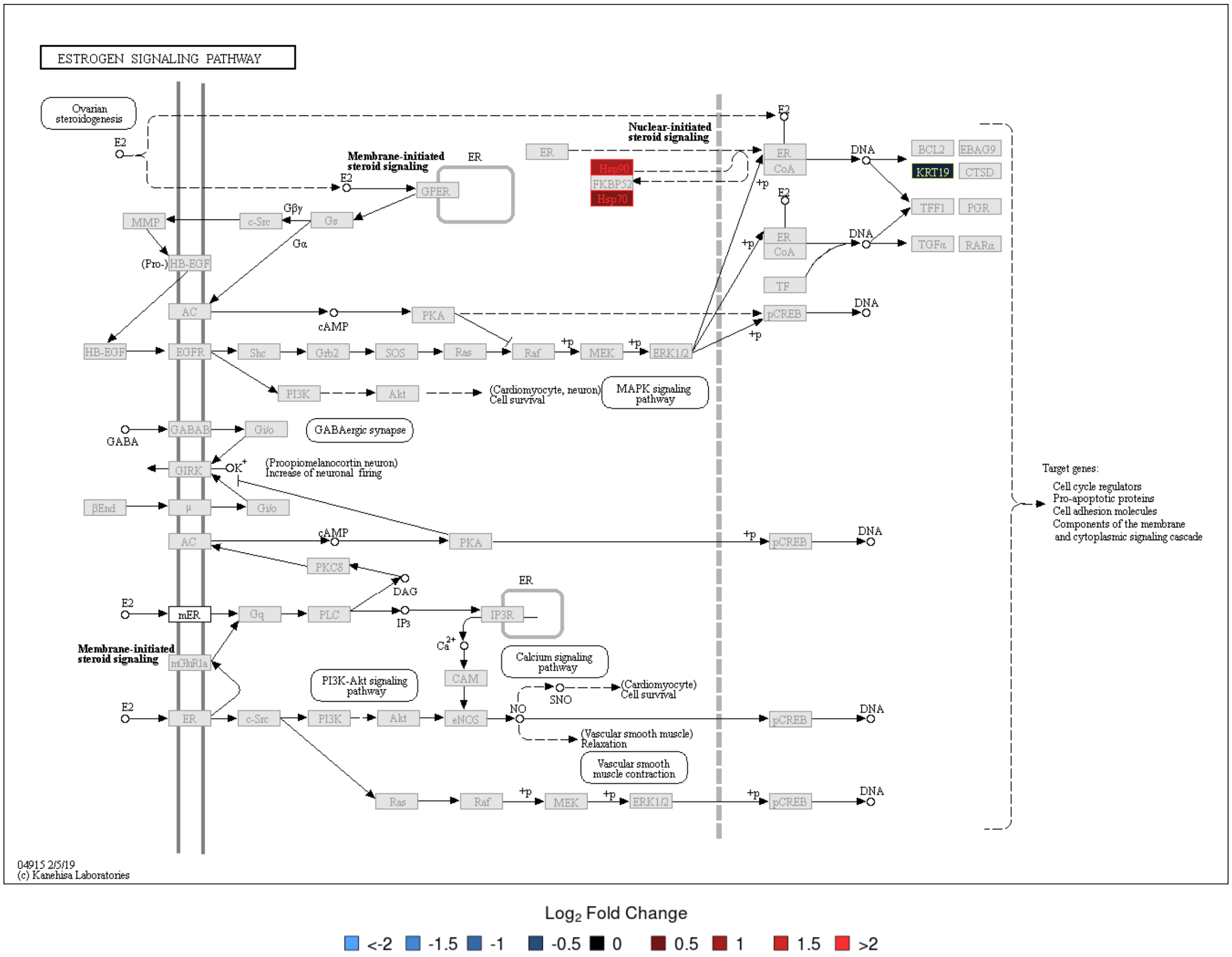

Figure S38: KEGG Pathway Map - Estrogen signaling pathway - Homo sapiens (human) (2D-DIGE/MS, Day 14). Node color gradient encodes fold change from pre-vaccination (for multi-gene nodes the median fold change of corresponding proteins is used). In red: increased compared to pre-vaccination, in blue: decreased compared to pre-vaccination, in black: fold change close to 1, in dark grey: gene encoding for protein that was not experimentally identified or had more than 20% missing observations, light grey: gene missing database mapping, white: non-human gene. Genes encoding for DA proteins are highlighted using red (significantly increased) and blue (significantly decreased) node label and border colors.

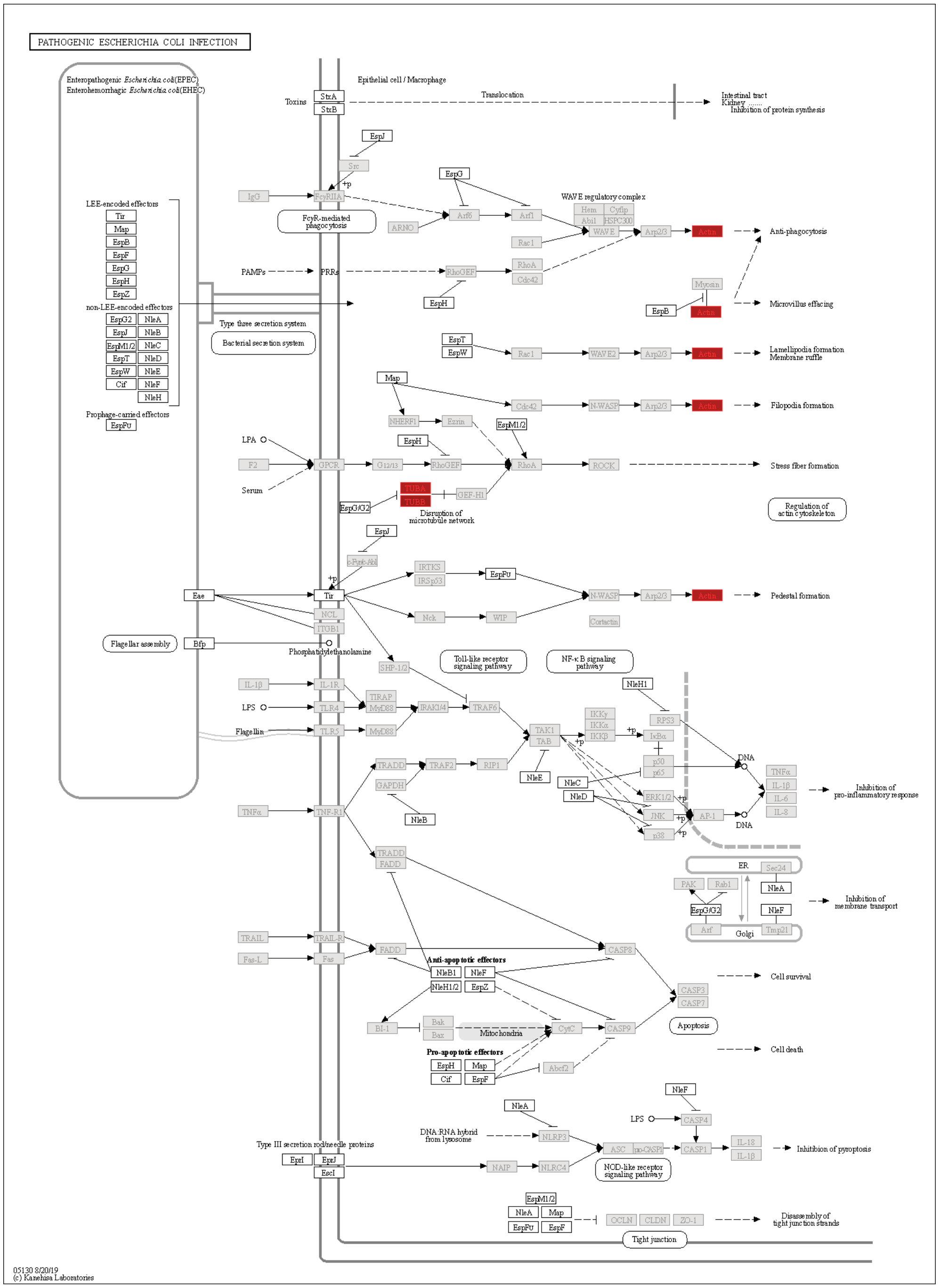

Log$_2$ Fold Change

<-2 | -1.5 | -1 | -0.5 | 0 | 0.5 | 1 | 1.5 | >2

Figure S39: KEGG Pathway Map - Pathogenic Escherichia coli infection - Homo sapiens (human) (2D-DIGE/MS, Day 7). Node color gradient encodes fold change from pre-vaccination (for multi-gene nodes the median fold change of corresponding proteins is used). In red: increased compared to pre-vaccination, in blue: decreased compared to pre-vaccination, in black: fold change close to 1, in dark grey: gene encoding for protein that was not experimentally identified or had more than 20% missing observations, light grey: gene missing database mapping, white: non-human gene. Genes encoding for DA proteins are highlighted using red (significantly increased) and blue (significantly decreased) node label and border colors.

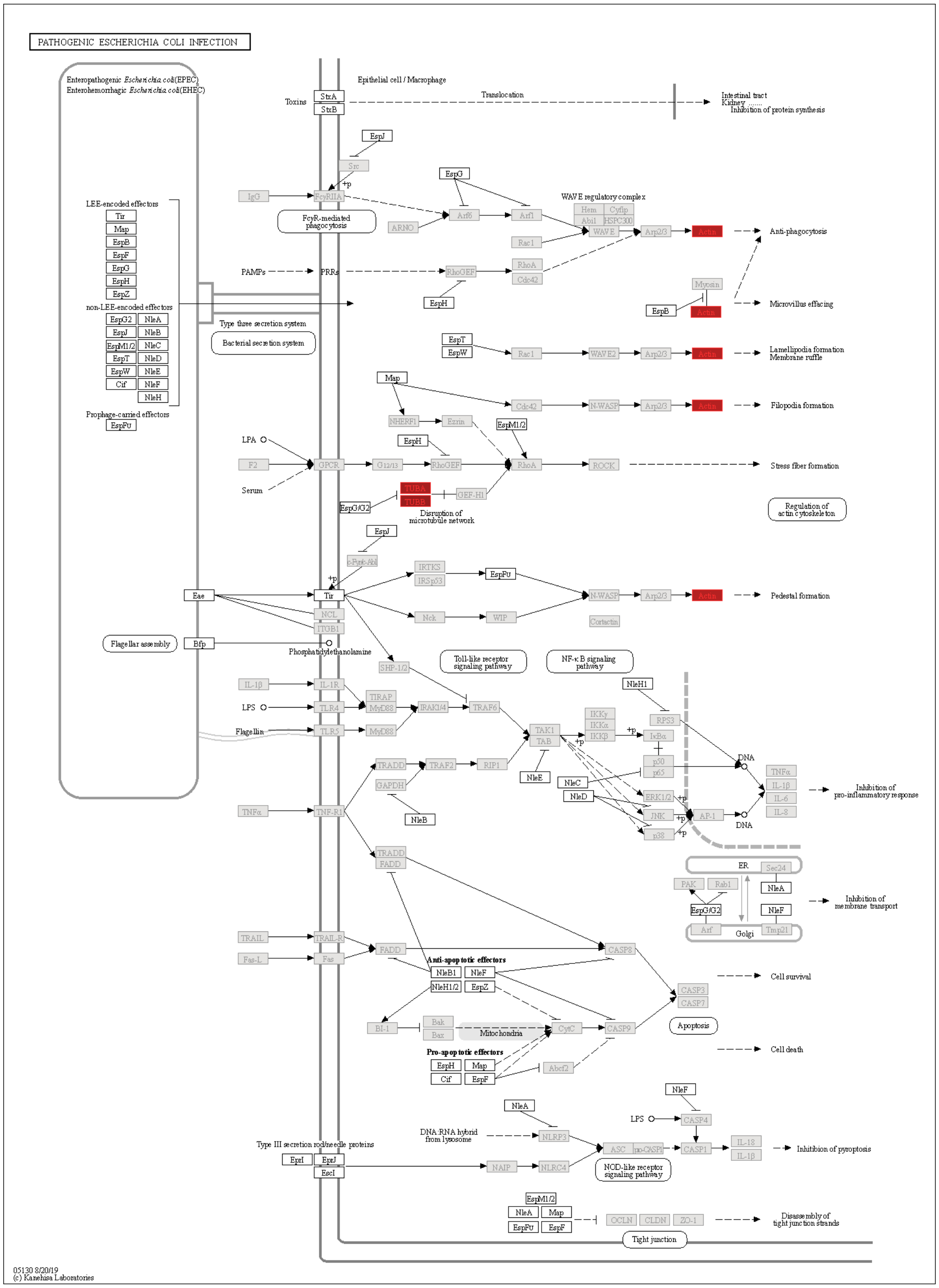

Log$_2$ Fold Change

<-2 -1.5 -1 -0.5 0 0.5 1 1.5 >2

Figure S40: KEGG Pathway Map - Pathogenic Escherichia coli infection - Homo sapiens (human) (2D-DIGE/MS, Day 14). Node color gradient encodes fold change from pre-vaccination (for multi-gene nodes the median fold change of corresponding proteins is used). In red: increased compared to pre-vaccination, in blue: decreased compared to pre-vaccination, in black: fold change close to 1, in dark grey: gene encoding for protein that was not experimentally identified or had more than 20% missing observations, light grey: gene missing database mapping, white: non-human gene. Genes encoding for DA proteins are highlighted using red (significantly increased) and blue (significantly decreased) node label and border colors.

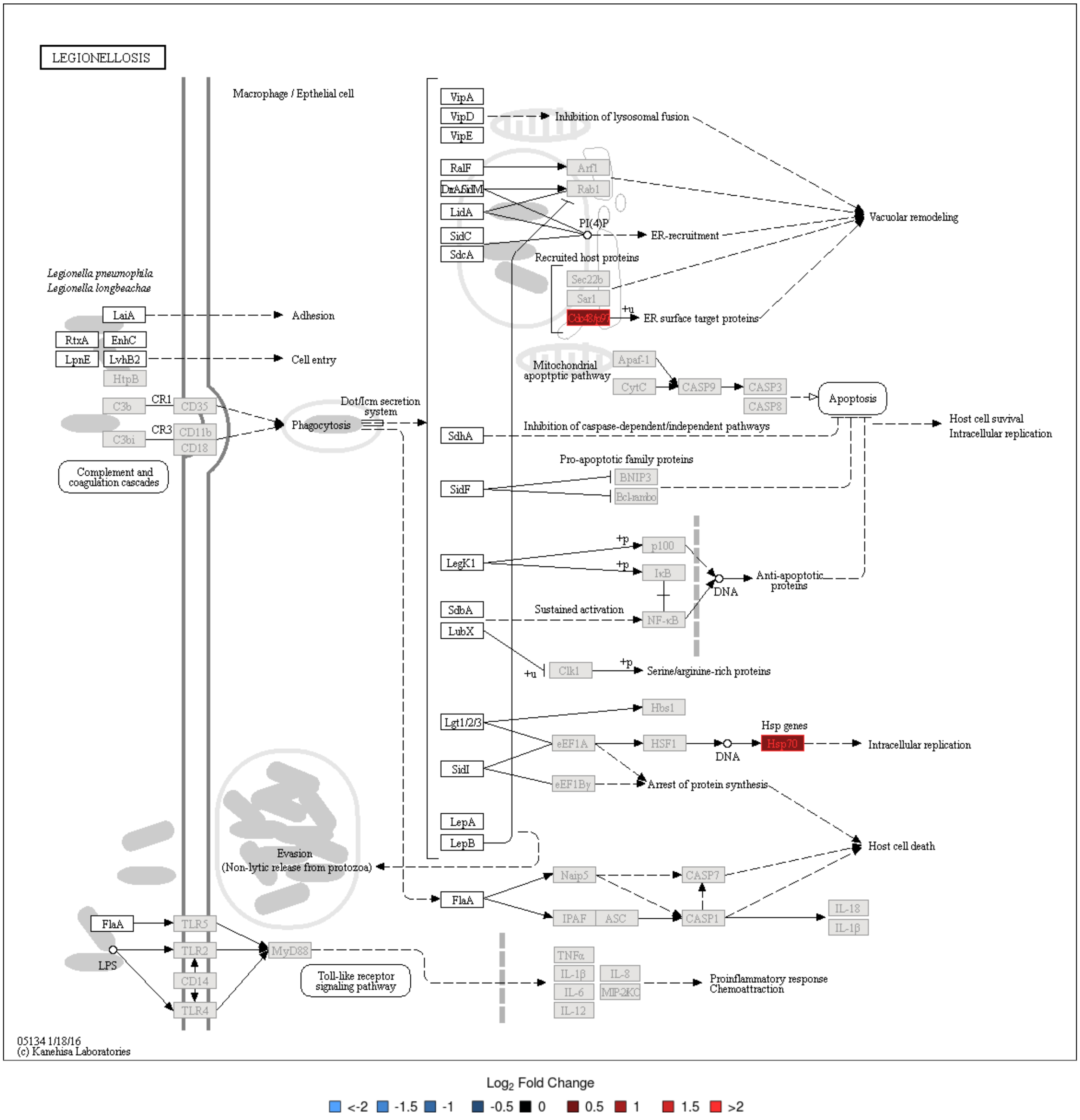

Figure S41: KEGG Pathway Map - Legionellosis - Homo sapiens (human) (2D-DIGE/MS, Day 14). Node color gradient encodes fold change from pre-vaccination (for multi-gene nodes the median fold change of corresponding proteins is used). In red: increased compared to pre-vaccination, in blue: decreased compared to pre-vaccination, in black: fold change close to 1, in dark grey: gene encoding for protein that was not experimentally identified or had more than 20% missing observations, light grey: gene missing database mapping, white: non-human gene. Genes encoding for DA proteins are highlighted using red (significantly increased) and blue (significantly decreased) node label and border colors.

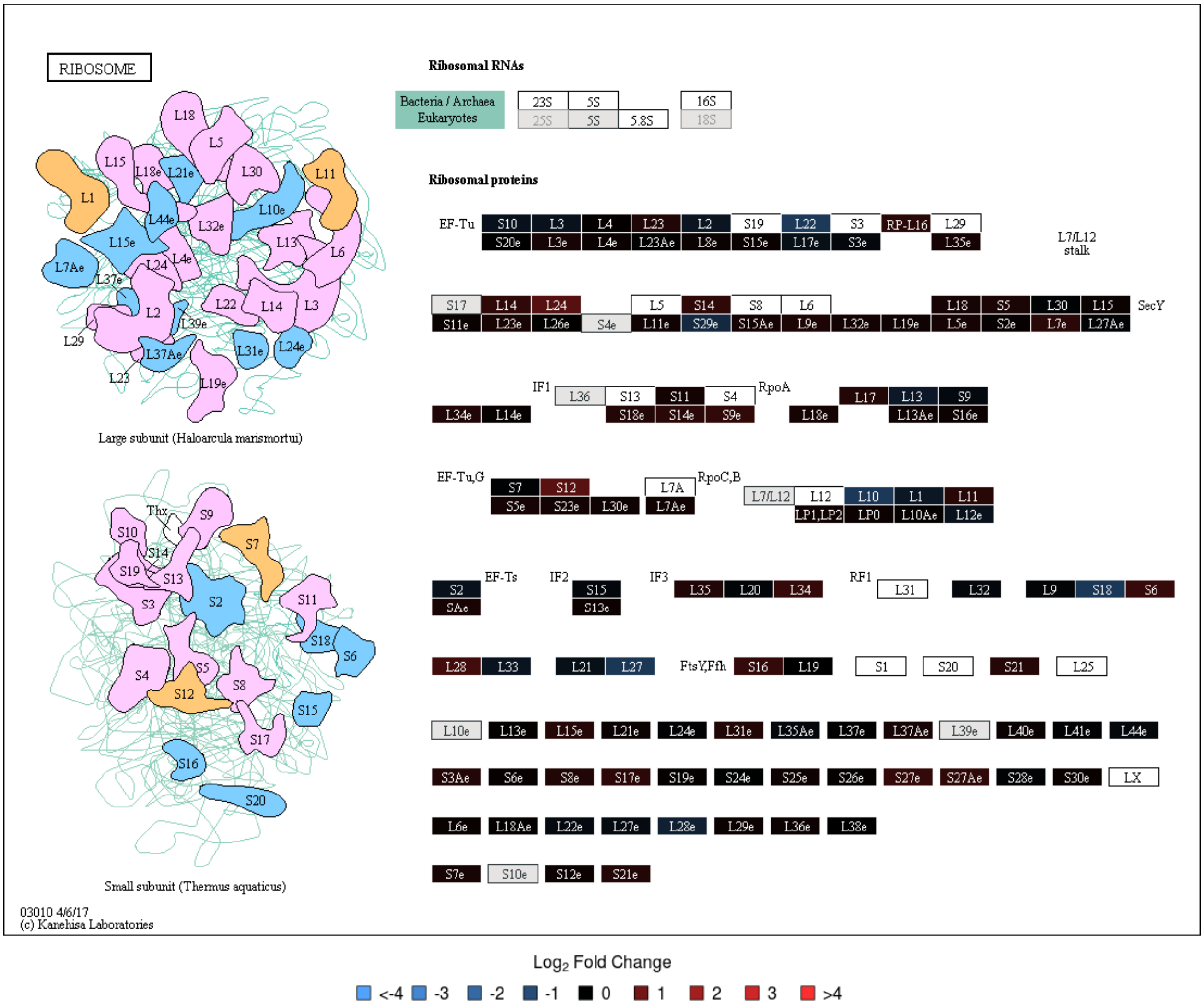

Figure S42: KEGG Pathway Map - Ribosome - Homo sapiens (human) (RNA-Seq, Saint Louis University, Day 1). Node color gradient encodes fold change from pre-vaccination (for multi-gene pathway nodes the median fold change is used). In red: up-regulated compared to pre-vaccination, in blue: down-regulated compared to pre-vaccination. In black: fold change close to 1, in dark grey: genes filtered out due to low overall expression, light grey: gene missing database mapping, white: non-human gene. DE genes are highlighted using red (significantly up-regulated) and blue (significantly down-regulated) node label and border colors.

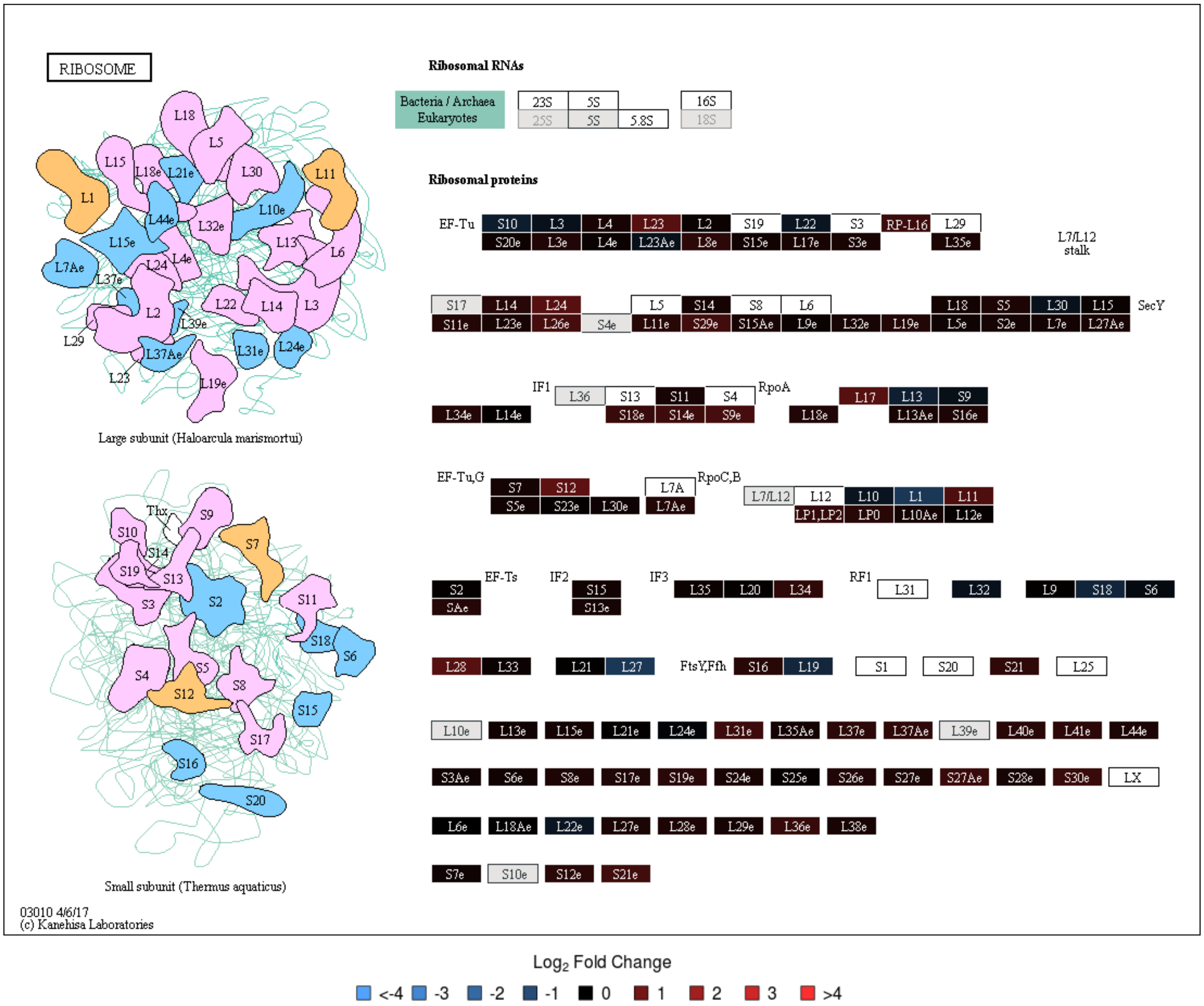

Figure S43: KEGG Pathway Map - Ribosome - Homo sapiens (human) (RNA-Seq, Saint Louis University, Day 2). Node color gradient encodes fold change from pre-vaccination (for multi-gene pathway nodes the median fold change is used). In red: up-regulated compared to pre-vaccination, in blue: down-regulated compared to pre-vaccination. In black: fold change close to 1, in dark grey: genes filtered out due to low overall expression, light grey: gene missing database mapping, white: non-human gene. DE genes are highlighted using red (significantly up-regulated) and blue (significantly down-regulated) node label and border colors.

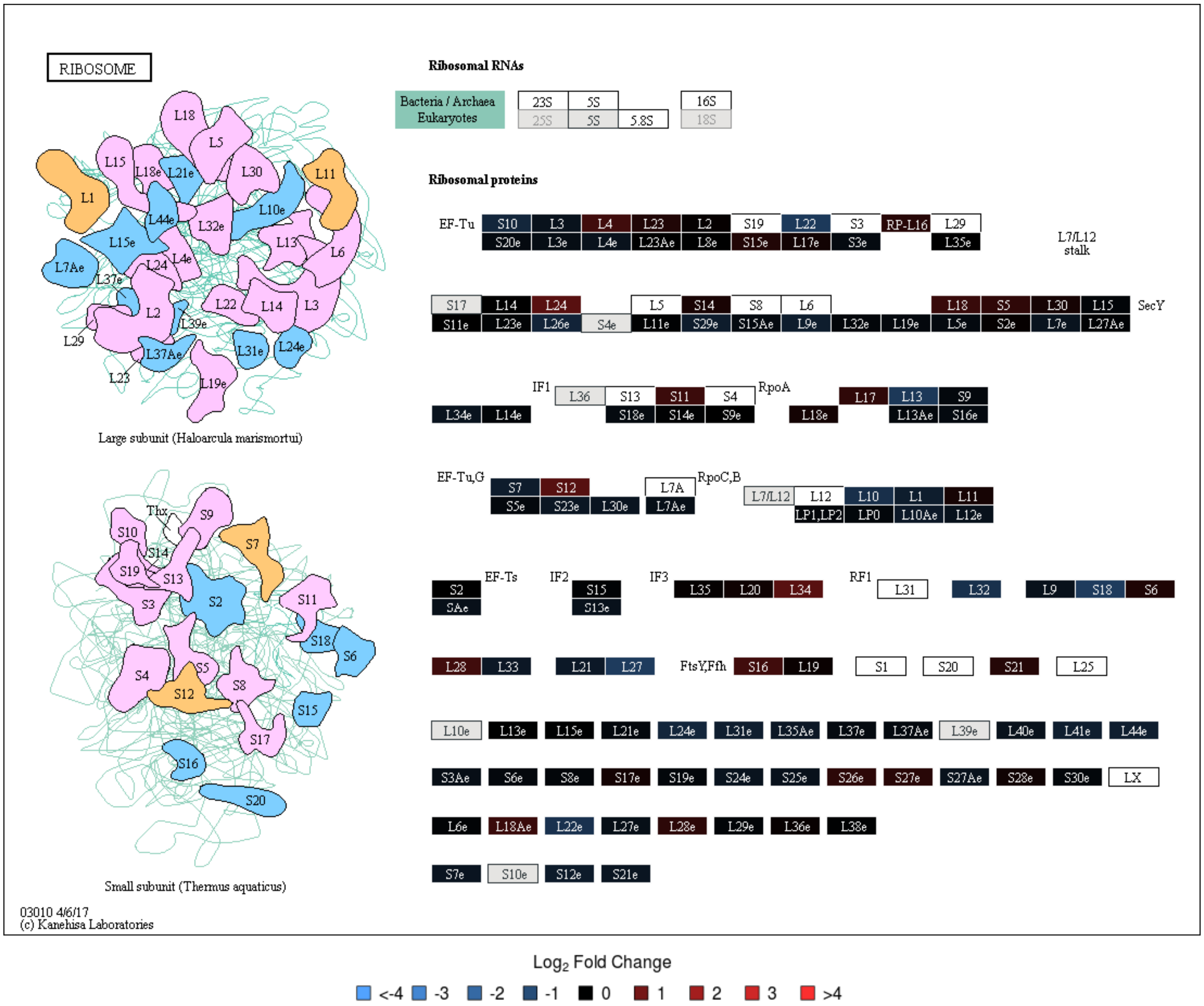

Figure S44: KEGG Pathway Map - Ribosome - Homo sapiens (human) (RNA-Seq, Saint Louis University, Day 7). Node color gradient encodes fold change from pre-vaccination (for multi-gene pathway nodes the median fold change is used). In red: up-regulated compared to pre-vaccination, in blue: down-regulated compared to pre-vaccination. In black: fold change close to 1, in dark grey: genes filtered out due to low overall expression, light grey: gene missing database mapping, white: non-human gene. DE genes are highlighted using red (significantly up-regulated) and blue (significantly down-regulated) node label and border colors.

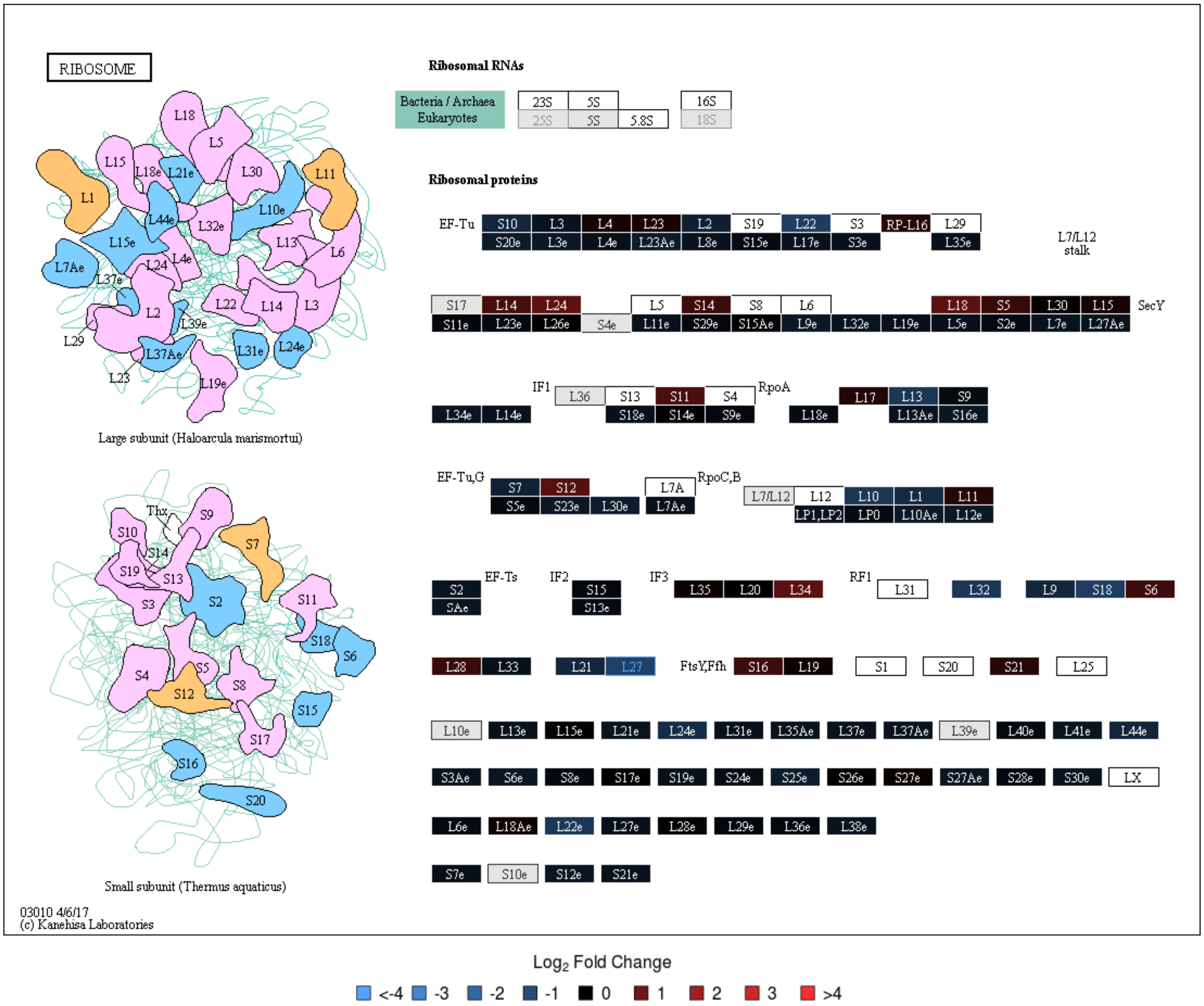

Figure S45: KEGG Pathway Map - Ribosome - Homo sapiens (human) (RNA-Seq, Saint Louis University, Day 14). Node color gradient encodes fold change from pre-vaccination (for multi-gene pathway nodes the median fold change is used). In red: up-regulated compared to pre-vaccination, in blue: down-regulated compared to pre-vaccination. In black: fold change close to 1, in dark grey: genes filtered out due to low overall expression, light grey: gene missing database mapping, white: non-human gene. DE genes are highlighted using red (significantly up-regulated) and blue (significantly down-regulated) node label and border colors.

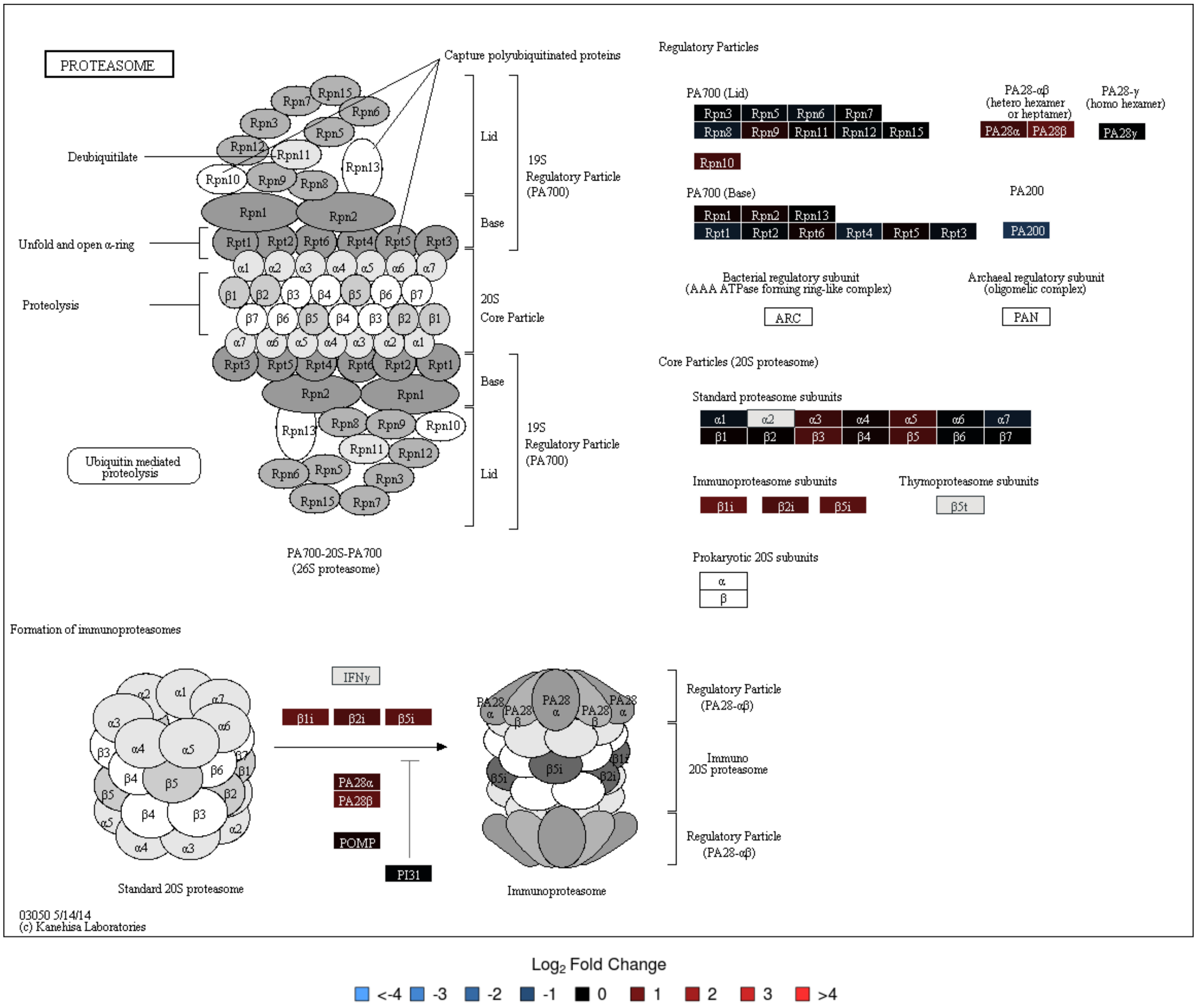

Figure S46: KEGG Pathway Map - Proteasome - Homo sapiens (human) (RNA-Seq, Saint Louis University, Day 1). Node color gradient encodes fold change from pre-vaccination (for multi-gene pathway nodes the median fold change is used). In red: up-regulated compared to pre-vaccination, in blue: down-regulated compared to pre-vaccination. In black: fold change close to 1, in dark grey: genes filtered out due to low overall expression, light grey: gene missing database mapping, white: non-human gene. DE genes are highlighted using red (significantly up-regulated) and blue (significantly down-regulated) node label and border colors.

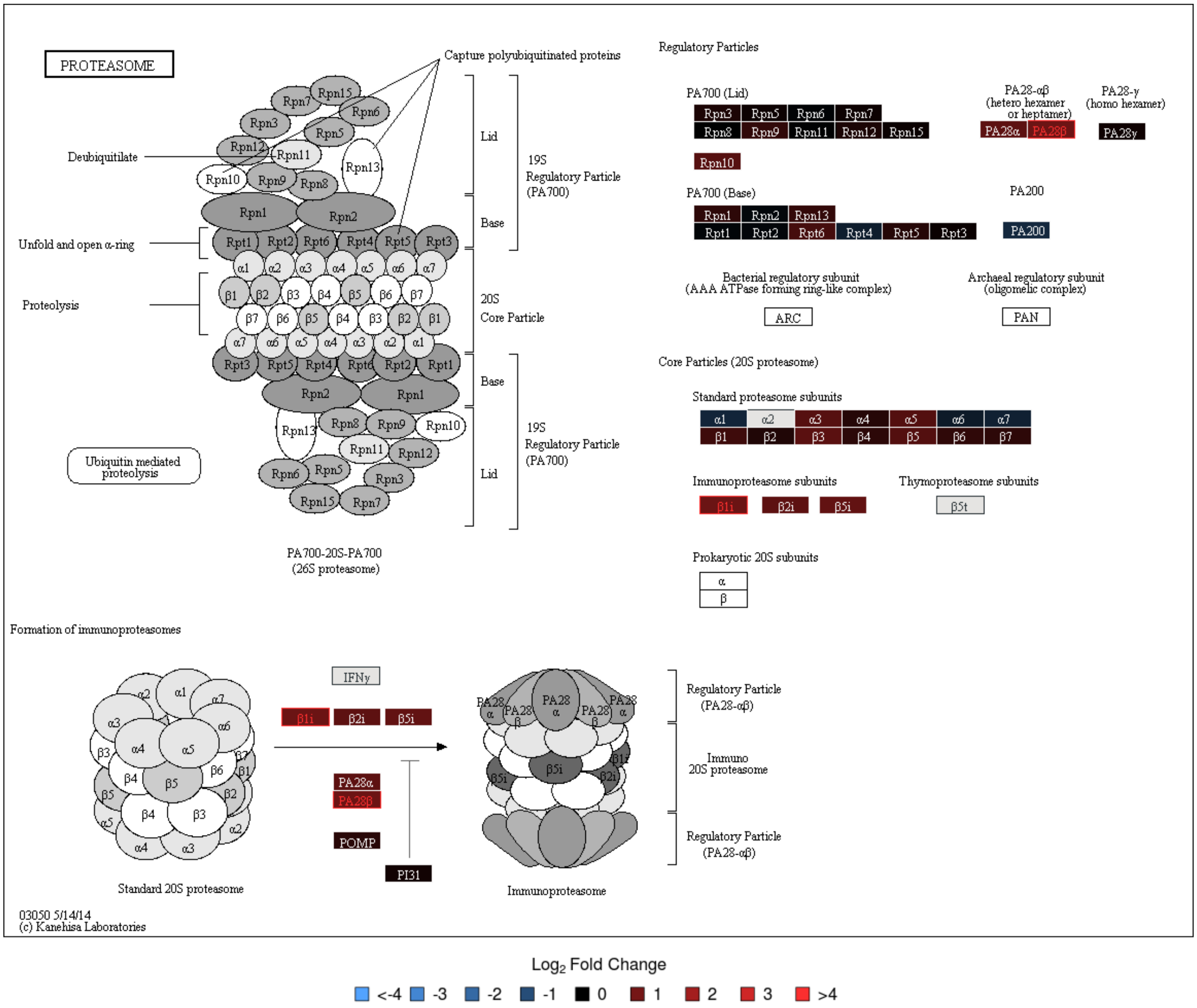

Figure S47: KEGG Pathway Map - Proteasome - Homo sapiens (human) (RNA-Seq, Saint Louis University, Day 2). Node color gradient encodes fold change from pre-vaccination (for multi-gene pathway nodes the median fold change is used). In red: up-regulated compared to pre-vaccination, in blue: down-regulated compared to pre-vaccination. In black: fold change close to 1, in dark grey: genes filtered out due to low overall expression, light grey: gene missing database mapping, white: non-human gene. DE genes are highlighted using red (significantly up-regulated) and blue (significantly down-regulated) node label and border colors.

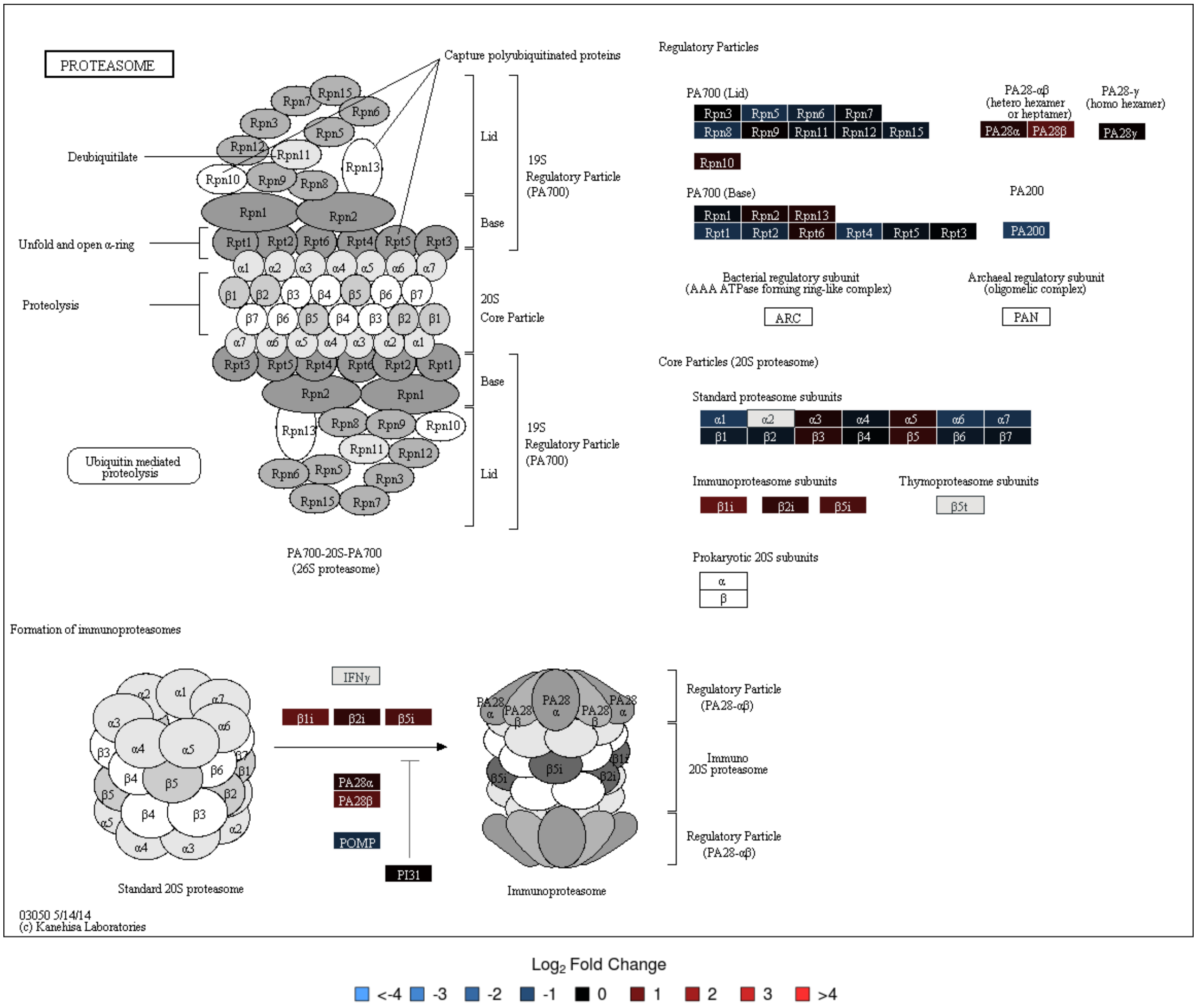

Figure S48: KEGG Pathway Map - Proteasome - Homo sapiens (human) (RNA-Seq, Saint Louis University, Day 7). Node color gradient encodes fold change from pre-vaccination (for multi-gene pathway nodes the median fold change is used). In red: up-regulated compared to pre-vaccination, in blue: down-regulated compared to pre-vaccination. In black: fold change close to 1, in dark grey: genes filtered out due to low overall expression, light grey: gene missing database mapping, white: non-human gene. DE genes are highlighted using red (significantly up-regulated) and blue (significantly down-regulated) node label and border colors.

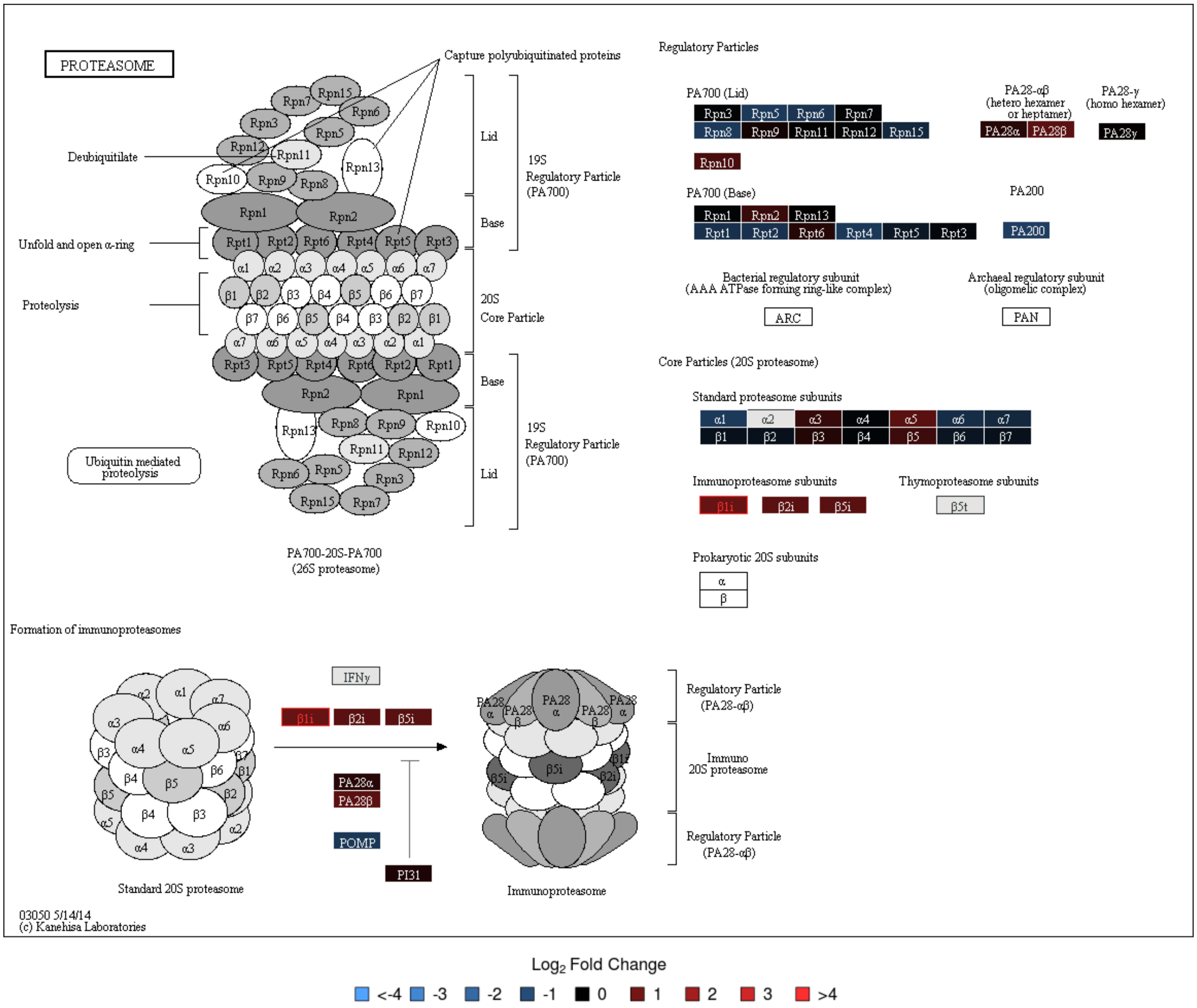

Figure S49: KEGG Pathway Map - Proteasome - Homo sapiens (human) (RNA-Seq, Saint Louis University, Day 14). Node color gradient encodes fold change from pre-vaccination (for multi-gene pathway nodes the median fold change is used). In red: up-regulated compared to pre-vaccination, in blue: down-regulated compared to pre-vaccination. In black: fold change close to 1, in dark grey: genes filtered out due to low overall expression, light grey: gene missing database mapping, white: non-human gene. DE genes are highlighted using red (significantly up-regulated) and blue (significantly down-regulated) node label and border colors.

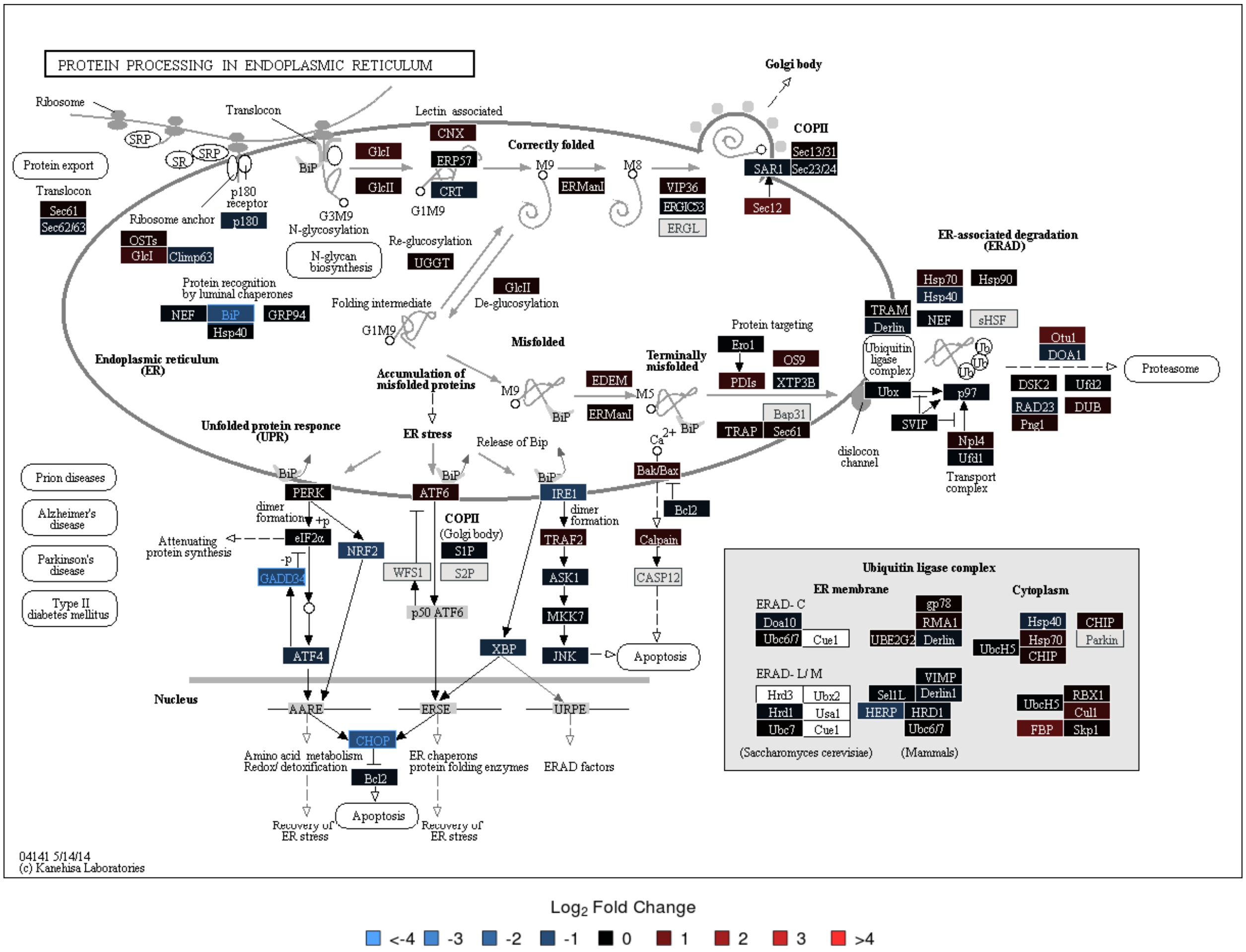

Figure S50: KEGG Pathway Map - Protein processing in endoplasmic reticulum - Homo sapiens (human) (RNA-Seq, Saint Louis University, Day 1). Node color gradient encodes fold change from pre-vaccination (for multi-gene pathway nodes the median fold change is used). In red: up-regulated compared to pre-vaccination, in blue: down-regulated compared to pre-vaccination. In black: fold change close to 1, in dark grey: genes filtered out due to low overall expression, light grey: gene missing database mapping, white: non-human gene. DE genes are highlighted using red (significantly up-regulated) and blue (significantly down-regulated) node label and border colors.

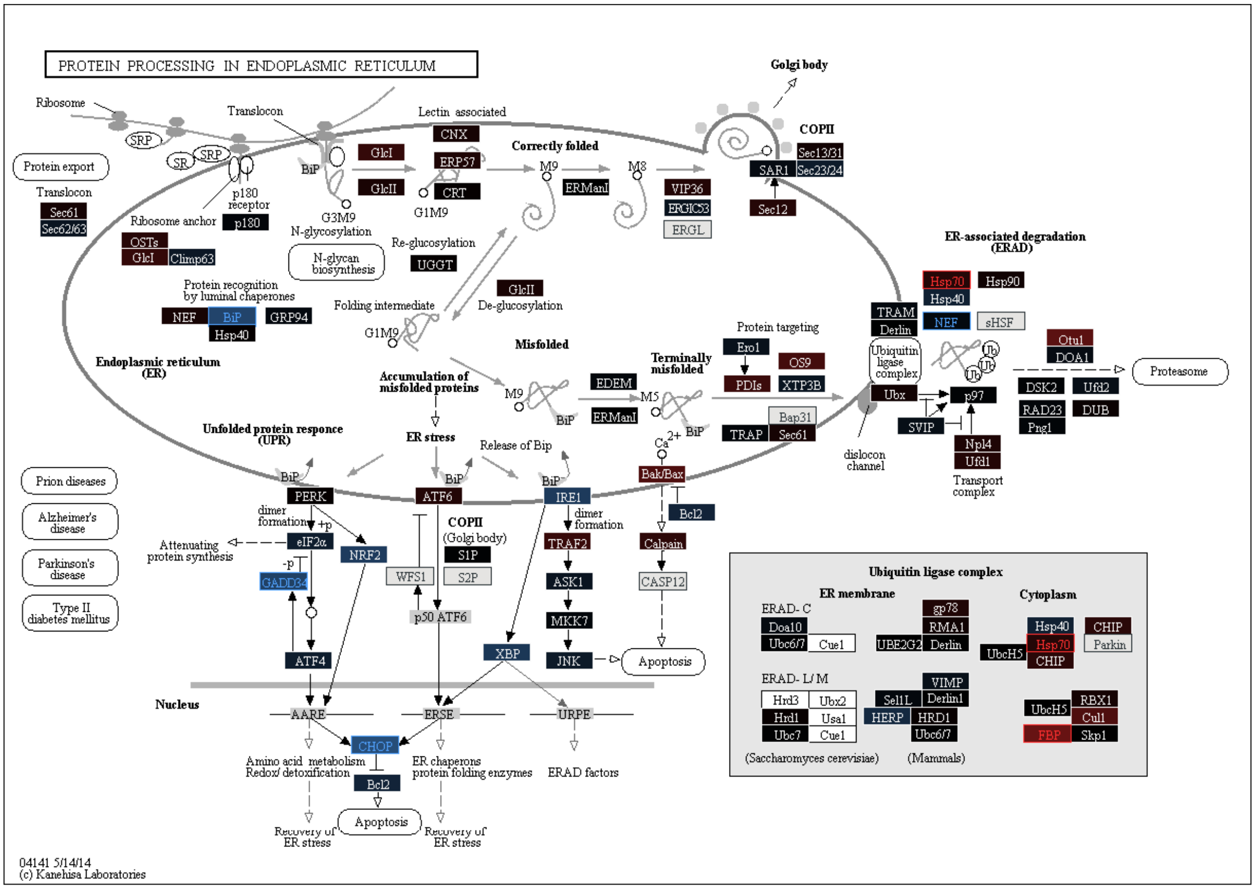

Log$_2$ Fold Change

<-4 | -3 | -2 | -1 | 0 | 1 | 2 | 3 | >4

Figure S51: KEGG Pathway Map - Protein processing in endoplasmic reticulum - Homo sapiens (human) (RNA-Seq, Saint Louis University, Day 2). Node color gradient encodes fold change from pre-vaccination (for multi-gene pathway nodes the median fold change is used). In red: up-regulated compared to pre-vaccination, in blue: down-regulated compared to pre-vaccination. In black: fold change close to 1, in dark grey: genes filtered out due to low overall expression, light grey: gene missing database mapping, white: non-human gene. DE genes are highlighted using red (significantly up-regulated) and blue (significantly down-regulated) node label and border colors.

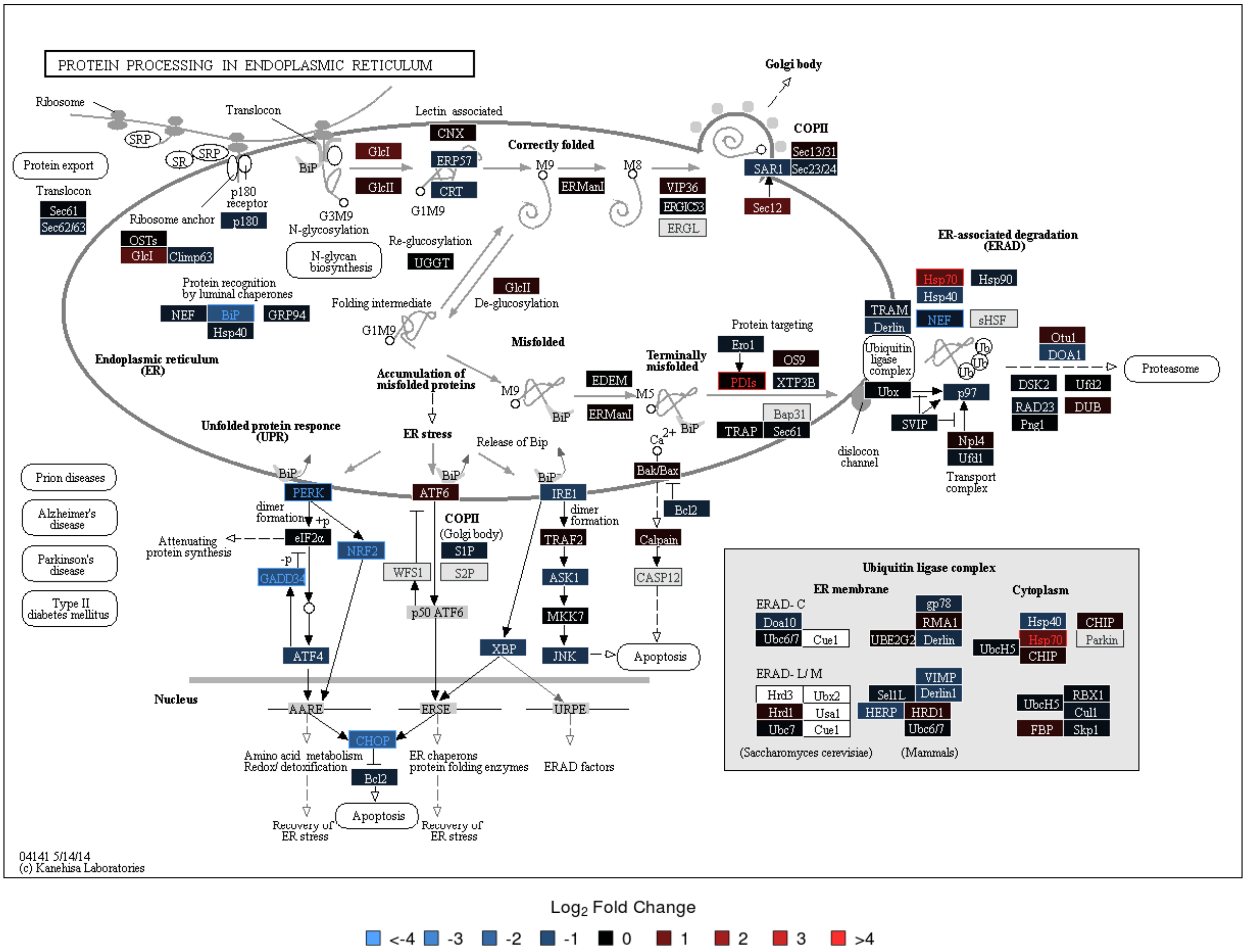

Figure S52: KEGG Pathway Map - Protein processing in endoplasmic reticulum - Homo sapiens (human) (RNA-Seq, Saint Louis University, Day 7). Node color gradient encodes fold change from pre-vaccination (for multi-gene pathway nodes the median fold change is used). In red: up-regulated compared to pre-vaccination, in blue: down-regulated compared to pre-vaccination. In black: fold change close to 1, in dark grey: genes filtered out due to low overall expression, light grey: gene missing database mapping, white: non-human gene. DE genes are highlighted using red (significantly up-regulated) and blue (significantly down-regulated) node label and border colors.

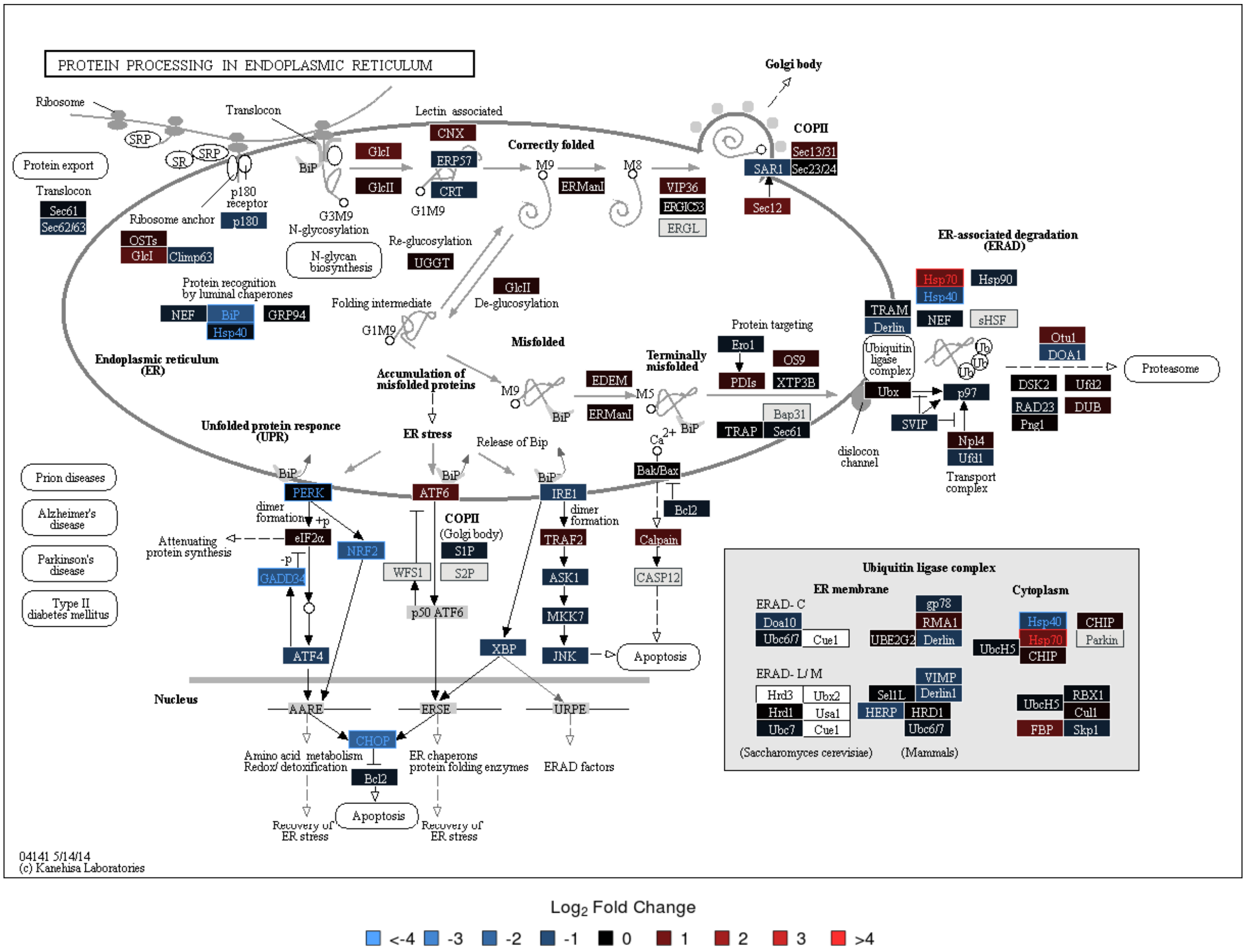

Figure S53: KEGG Pathway Map - Protein processing in endoplasmic reticulum - Homo sapiens (human) (RNA-Seq, Saint Louis University, Day 14). Node color gradient encodes fold change from pre-vaccination (for multi-gene pathway nodes the median fold change is used). In red: up-regulated compared to pre-vaccination, in blue: down-regulated compared to pre-vaccination. In black: fold change close to 1, in dark grey: genes filtered out due to low overall expression, light grey: gene missing database mapping, white: non-human gene. DE genes are highlighted using red (significantly up-regulated) and blue (significantly down-regulated) node label and border colors.

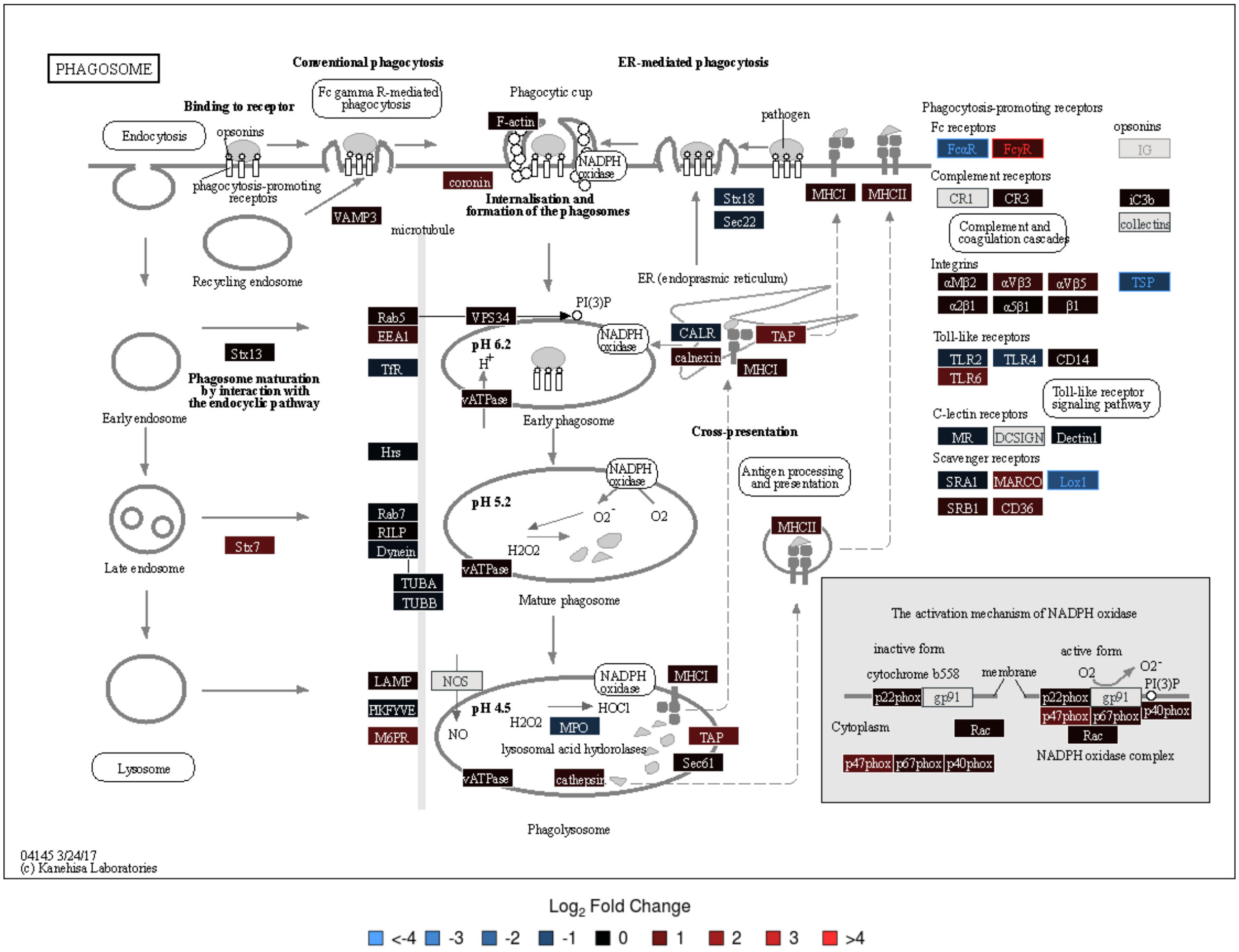

Figure S54: KEGG Pathway Map - Phagosome - Homo sapiens (human) (RNA-Seq, Saint Louis University, Day 1). Node color gradient encodes fold change from pre-vaccination (for multi-gene pathway nodes the median fold change is used). In red: up-regulated compared to pre-vaccination, in blue: down-regulated compared to pre-vaccination. In black: fold change close to 1, in dark grey: genes filtered out due to low overall expression, light grey: gene missing database mapping, white: non-human gene. DE genes are highlighted using red (significantly up-regulated) and blue (significantly down-regulated) node label and border colors.

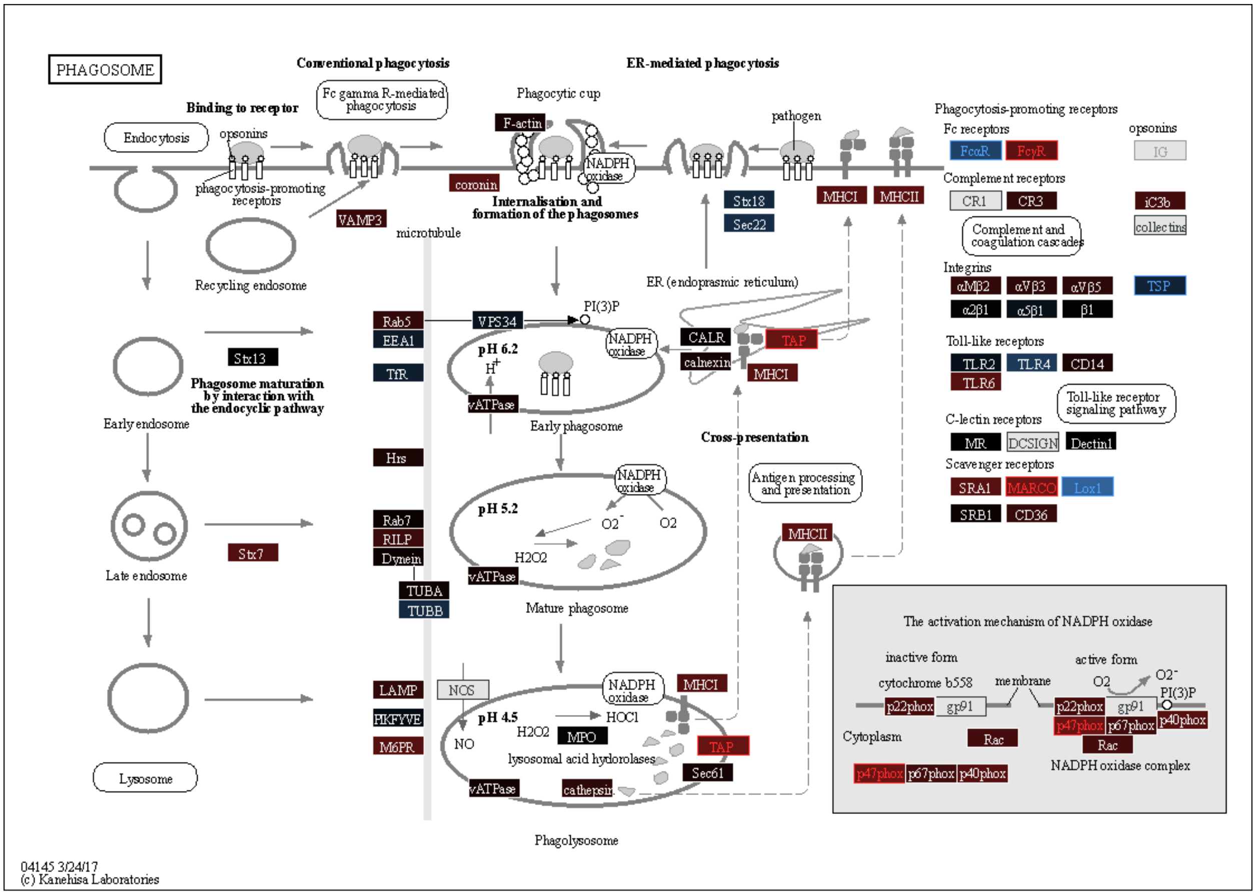

Log_2 Fold Change

<-4 -3 -2 -1 0 1 2 3 >4

Figure S55: KEGG Pathway Map - Phagosome - Homo sapiens (human) (RNA-Seq, Saint Louis University, Day 2). Node color gradient encodes fold change from pre-vaccination (for multi-gene pathway nodes the median fold change is used). In red: up-regulated compared to pre-vaccination, in blue: down-regulated compared to pre-vaccination. In black: fold change close to 1, in dark grey: genes filtered out due to low overall expression, light grey: gene missing database mapping, white: non-human gene. DE genes are highlighted using red (significantly up-regulated) and blue (significantly down-regulated) node label and border colors.

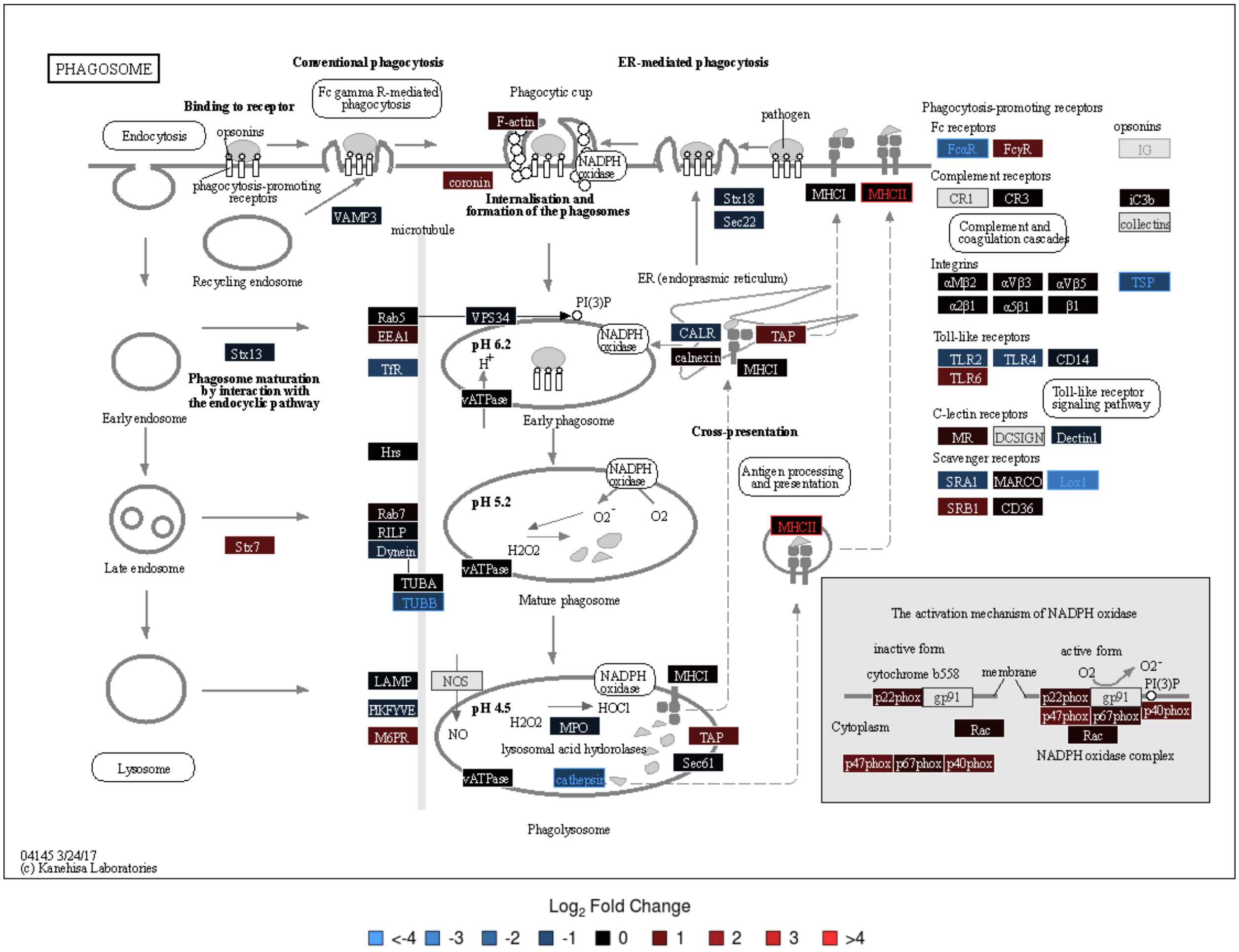

Figure S56: KEGG Pathway Map - Phagosome - Homo sapiens (human) (RNA-Seq, Saint Louis University, Day 7). Node color gradient encodes fold change from pre-vaccination (for multi-gene pathway nodes the median fold change is used). In red: up-regulated compared to pre-vaccination, in blue: down-regulated compared to pre-vaccination. In black: fold change close to 1, in dark grey: genes filtered out due to low overall expression, light grey: gene missing database mapping, white: non-human gene. DE genes are highlighted using red (significantly up-regulated) and blue (significantly down-regulated) node label and border colors.

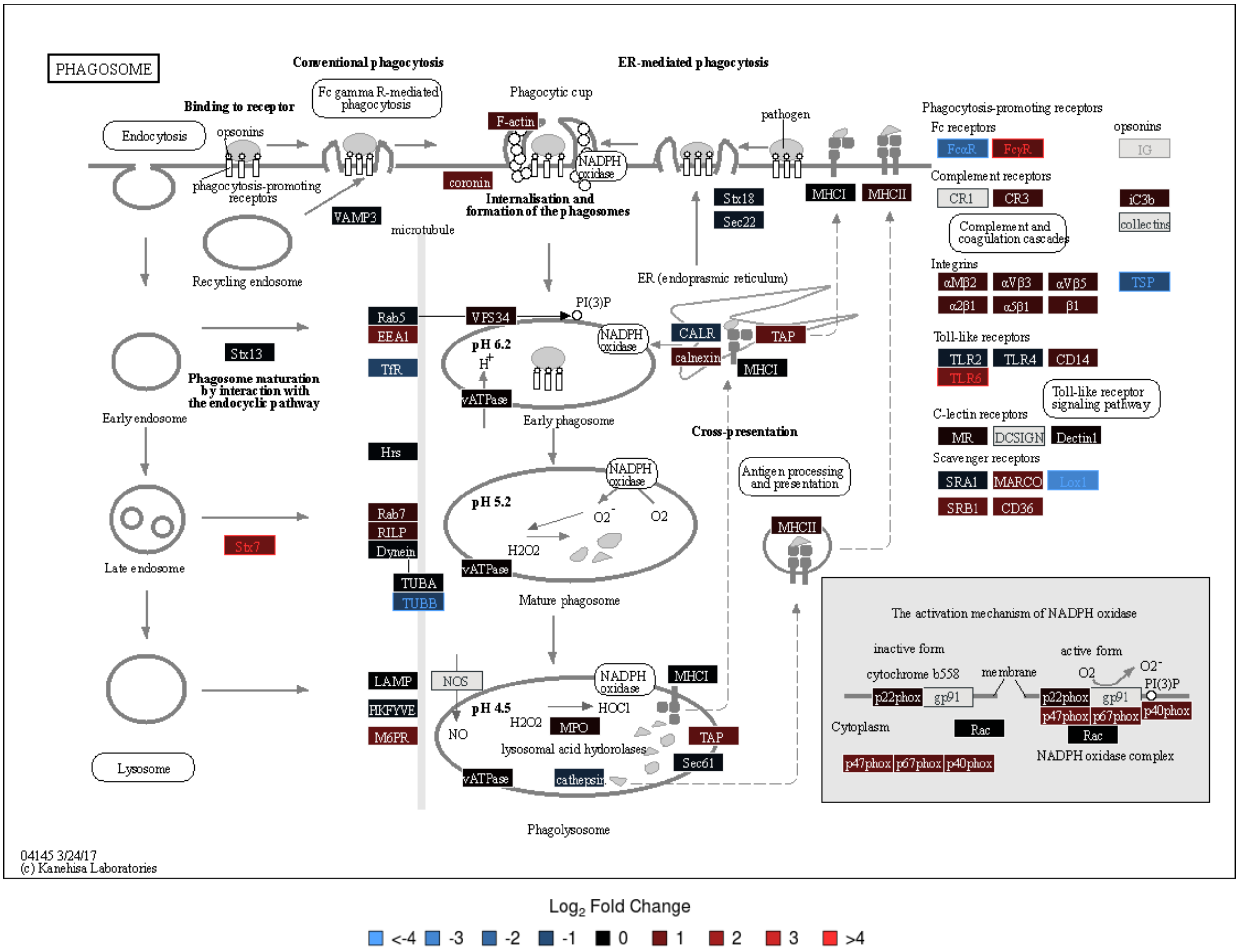

Figure S57: KEGG Pathway Map - Phagosome - Homo sapiens (human) (RNA-Seq, Saint Louis University, Day 14). Node color gradient encodes fold change from pre-vaccination (for multi-gene pathway nodes the median fold change is used). In red: up-regulated compared to pre-vaccination, in blue: down-regulated compared to pre-vaccination. In black: fold change close to 1, in dark grey: genes filtered out due to low overall expression, light grey: gene missing database mapping, white: non-human gene. DE genes are highlighted using red (significantly up-regulated) and blue (significantly down-regulated) node label and border colors.

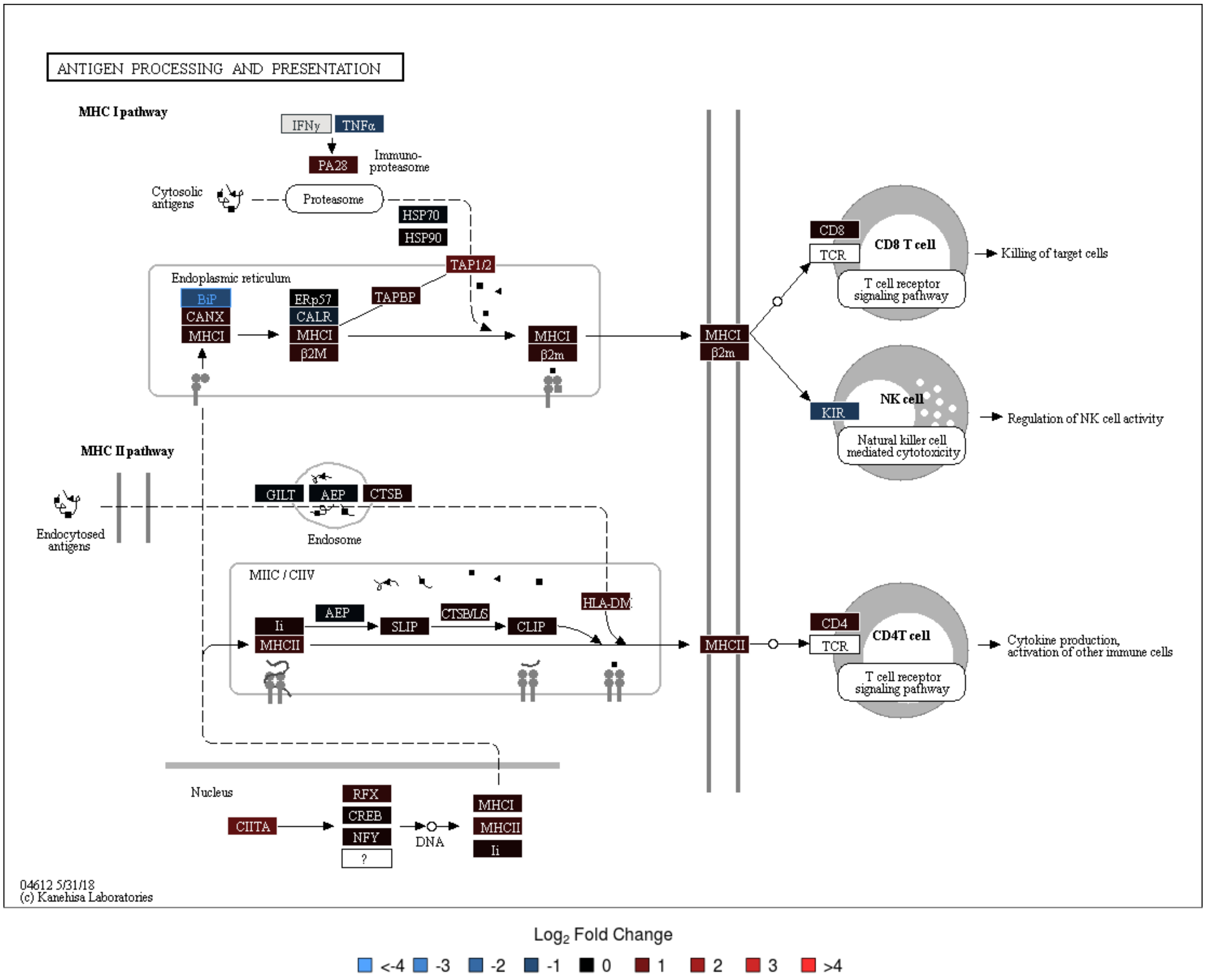

Figure S58: KEGG Pathway Map - Antigen processing and presentation - Homo sapiens (human) (RNA-Seq, Saint Louis University, Day 1). Node color gradient encodes fold change from pre-vaccination (for multi-gene pathway nodes the median fold change is used). In red: up-regulated compared to pre-vaccination, in blue: down-regulated compared to pre-vaccination. In black: fold change close to 1, in dark grey: genes filtered out due to low overall expression, light grey: gene missing database mapping, white: non-human gene. DE genes are highlighted using red (significantly up-regulated) and blue (significantly down-regulated) node label and border colors.

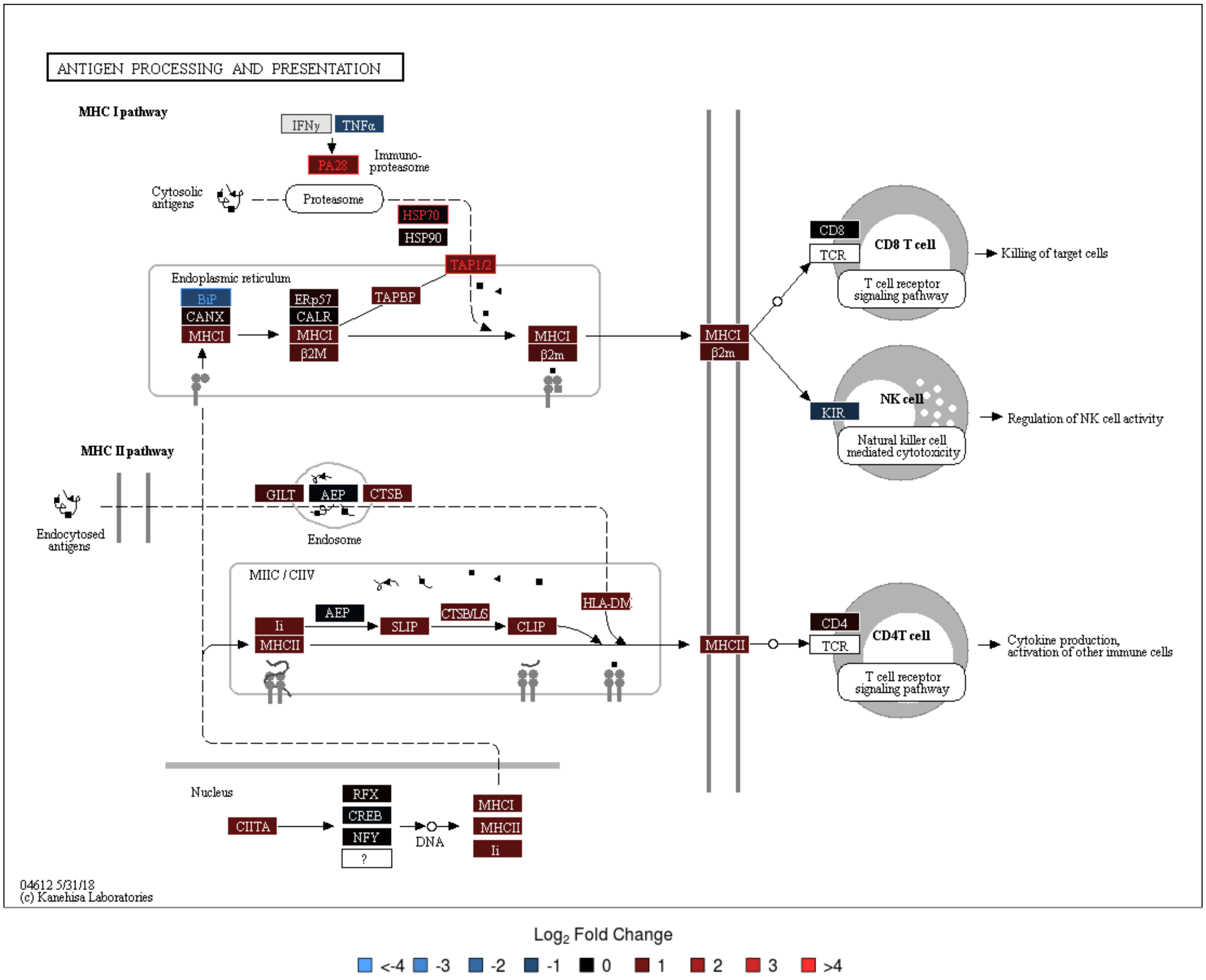

Figure S59: KEGG Pathway Map - Antigen processing and presentation - Homo sapiens (human) (RNA-Seq, Saint Louis University, Day 2). Node color gradient encodes fold change from pre-vaccination (for multi-gene pathway nodes the median fold change is used). In red: up-regulated compared to pre-vaccination, in blue: down-regulated compared to pre-vaccination. In black: fold change close to 1, in dark grey: genes filtered out due to low overall expression, light grey: gene missing database mapping, white: non-human gene. DE genes are highlighted using red (significantly up-regulated) and blue (significantly down-regulated) node label and border colors.

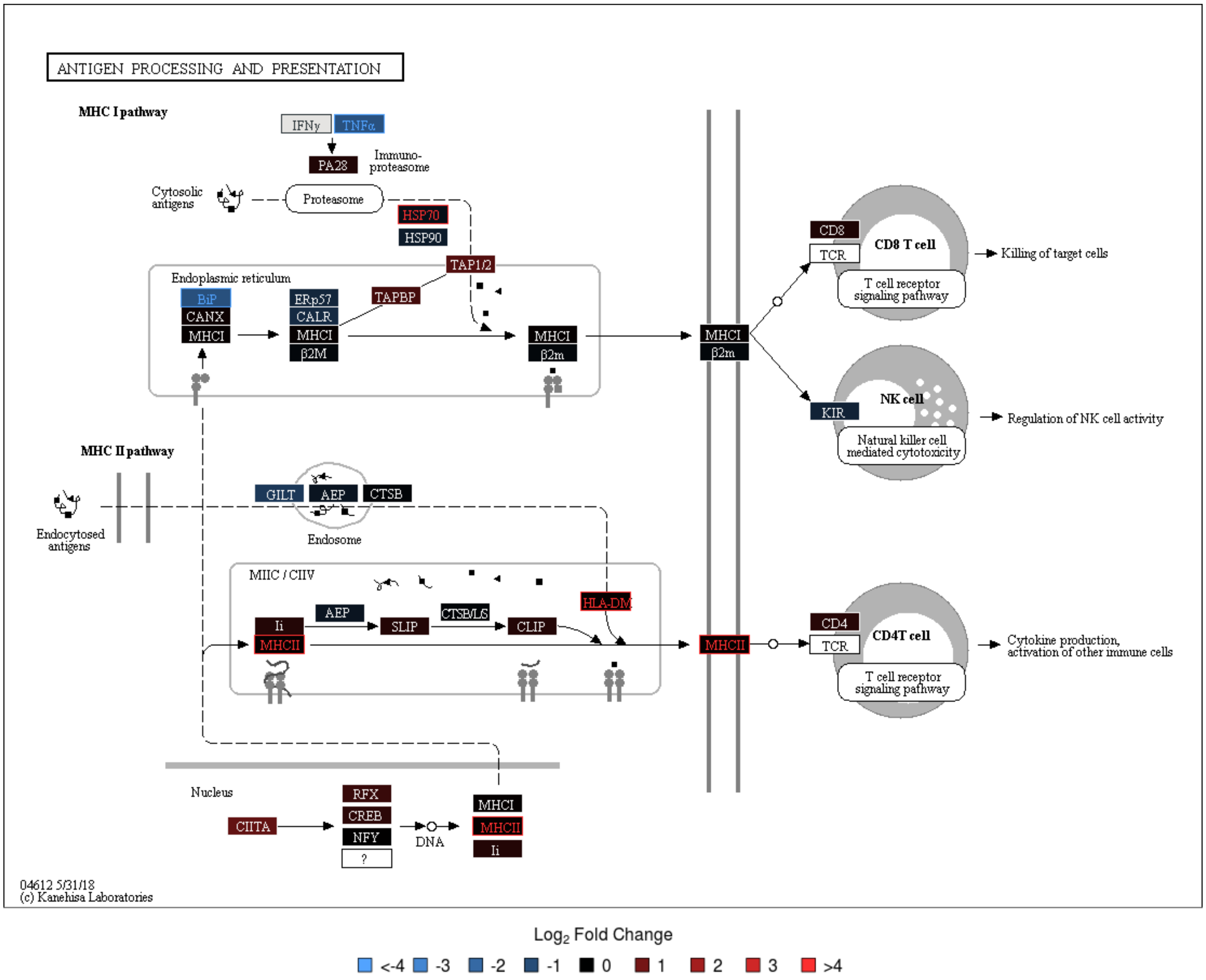

Figure S60: KEGG Pathway Map - Antigen processing and presentation - Homo sapiens (human) (RNA-Seq, Saint Louis University, Day 7). Node color gradient encodes fold change from pre-vaccination (for multi-gene pathway nodes the median fold change is used). In red: up-regulated compared to pre-vaccination, in blue: down-regulated compared to pre-vaccination. In black: fold change close to 1, in dark grey: genes filtered out due to low overall expression, light grey: gene missing database mapping, white: non-human gene. DE genes are highlighted using red (significantly up-regulated) and blue (significantly down-regulated) node label and border colors.

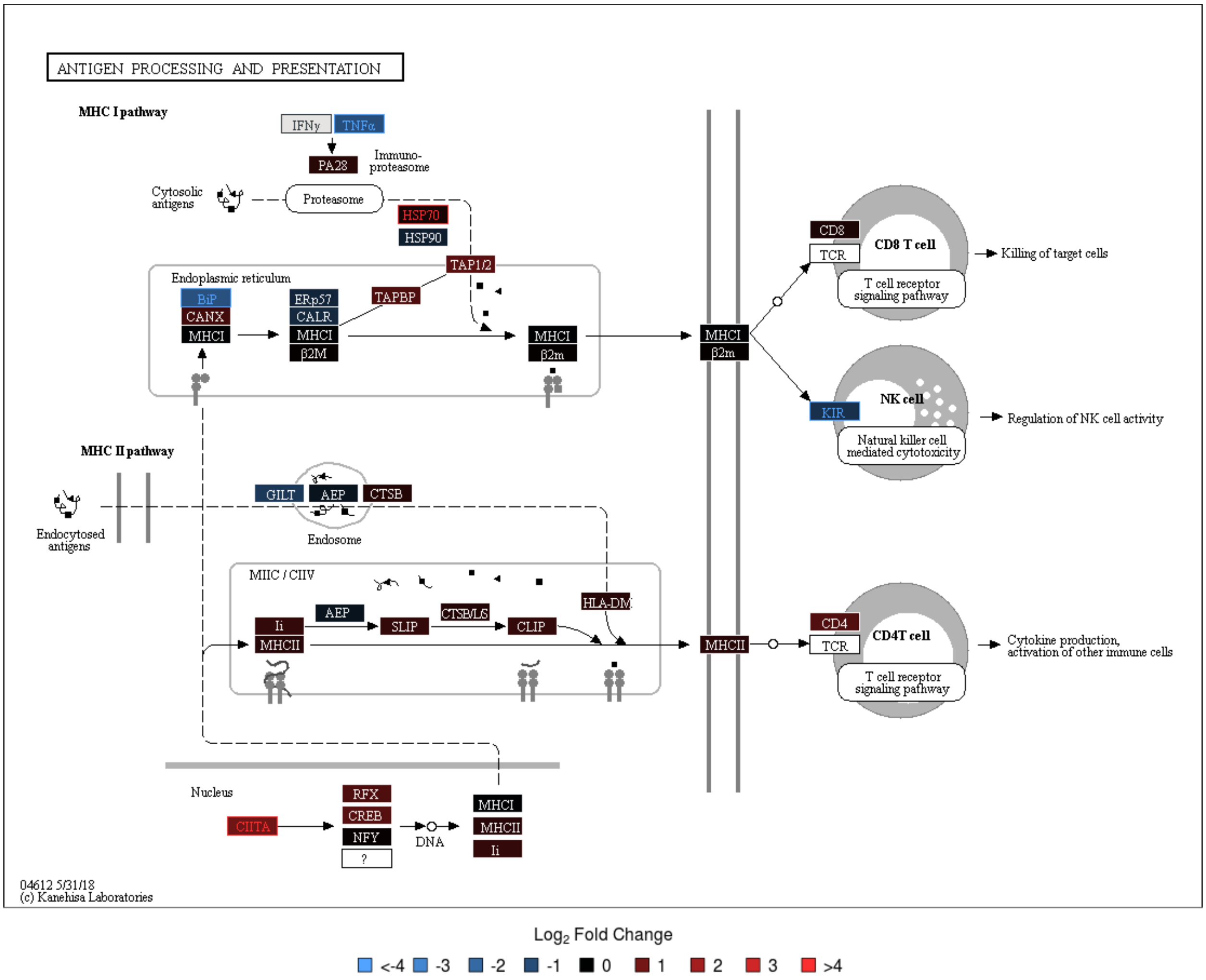

Figure S61: KEGG Pathway Map - Antigen processing and presentation - Homo sapiens (human) (RNA-Seq, Saint Louis University, Day 14). Node color gradient encodes fold change from pre-vaccination (for multi-gene pathway nodes the median fold change is used). In red: up-regulated compared to pre-vaccination, in blue: down-regulated compared to pre-vaccination. In black: fold change close to 1, in dark grey: genes filtered out due to low overall expression, light grey: gene missing database mapping, white: non-human gene. DE genes are highlighted using red (significantly up-regulated) and blue (significantly down-regulated) node label and border colors.

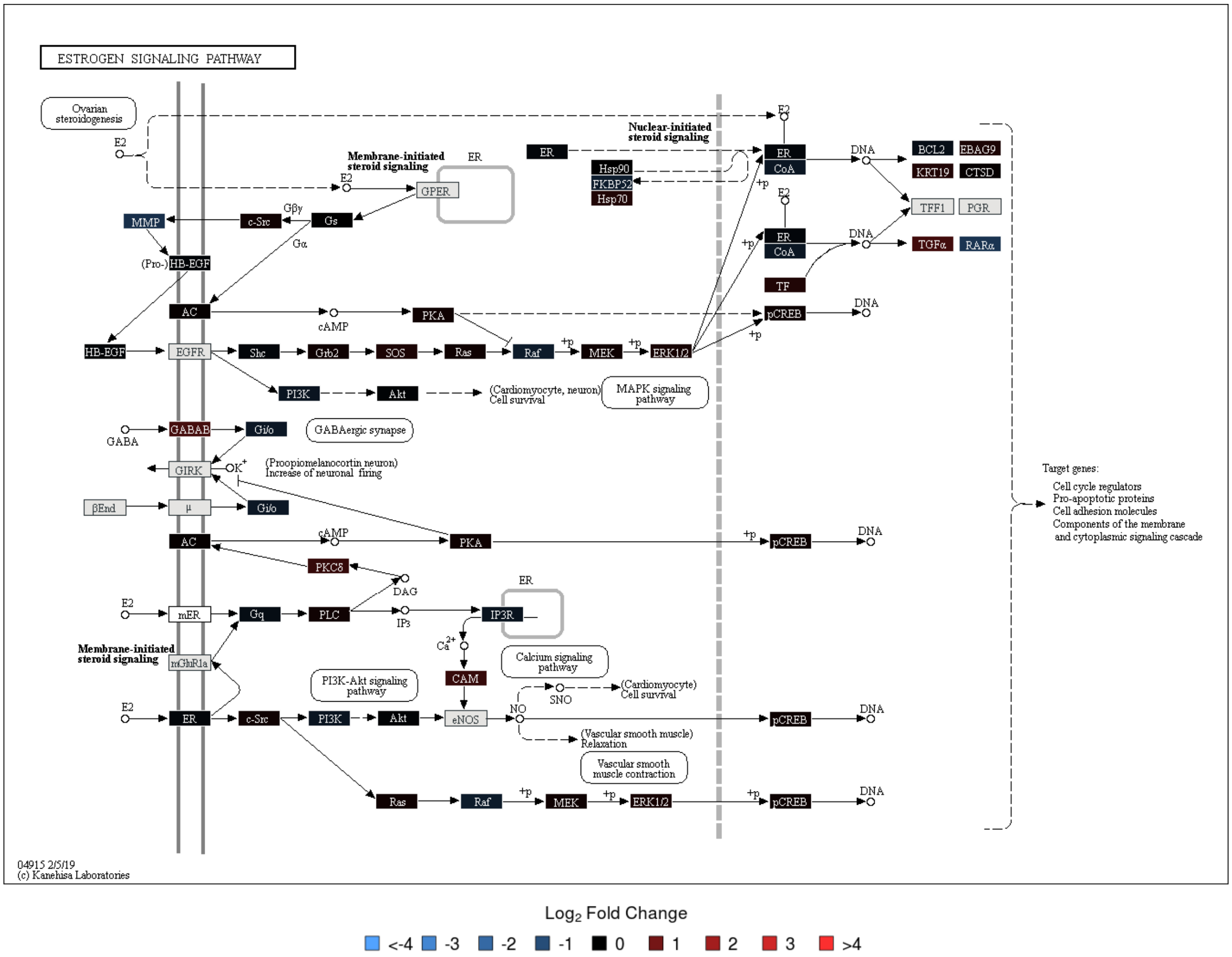

Figure S62: KEGG Pathway Map - Estrogen signaling pathway - Homo sapiens (human) (RNA-Seq, Saint Louis University, Day 1). Node color gradient encodes fold change from pre-vaccination (for multi-gene pathway nodes the median fold change is used). In red: up-regulated compared to pre-vaccination, in blue: down-regulated compared to pre-vaccination. In black: fold change close to 1, in dark grey: genes filtered out due to low overall expression, light grey: gene missing database mapping, white: non-human gene. DE genes are highlighted using red (significantly up-regulated) and blue (significantly down-regulated) node label and border colors.

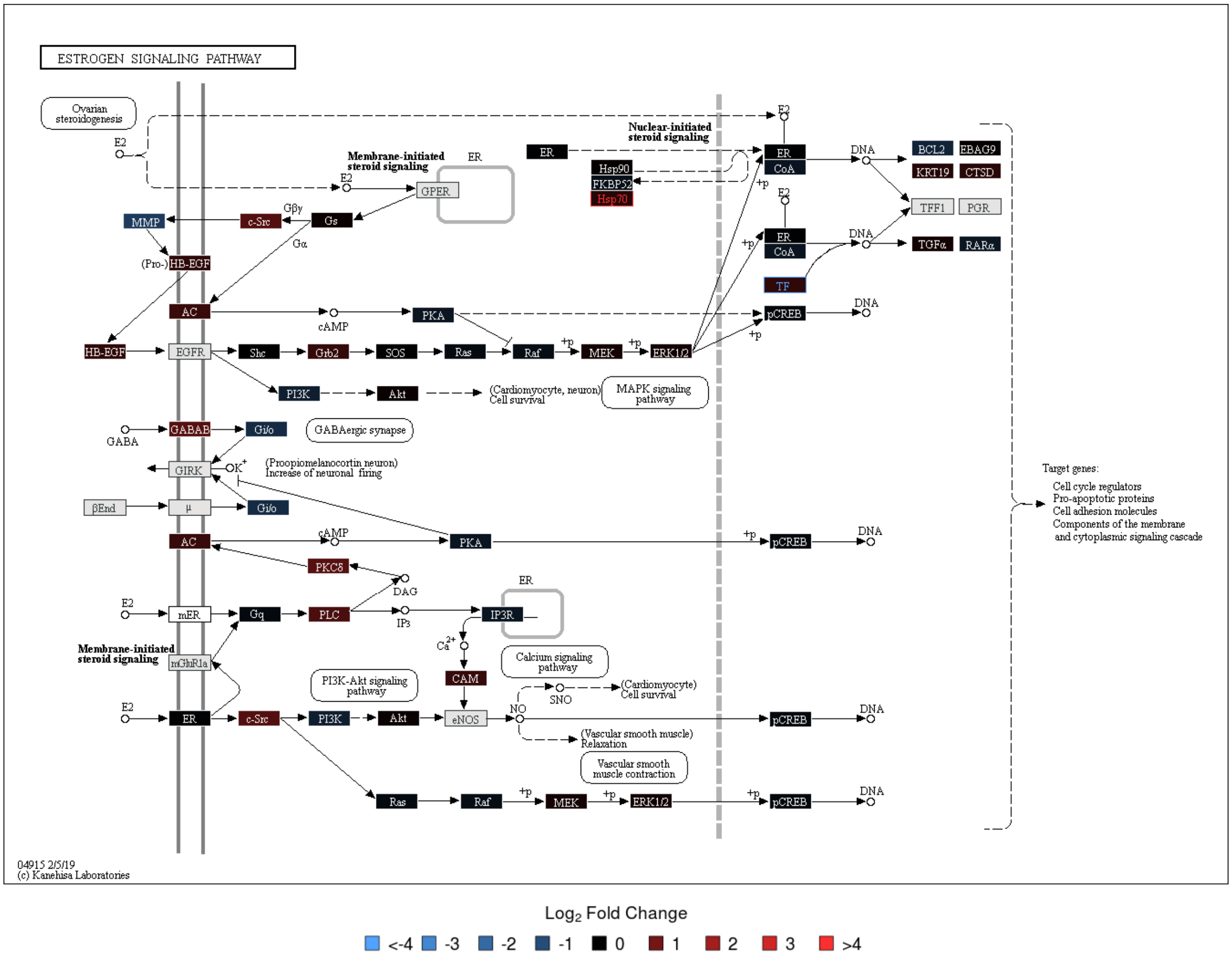

Figure S63: KEGG Pathway Map - Estrogen signaling pathway - Homo sapiens (human) (RNA-Seq, Saint Louis University, Day 2). Node color gradient encodes fold change from pre-vaccination (for multi-gene pathway nodes the median fold change is used). In red: up-regulated compared to pre-vaccination, in blue: down-regulated compared to pre-vaccination. In black: fold change close to 1, in dark grey: genes filtered out due to low overall expression, light grey: gene missing database mapping, white: non-human gene. DE genes are highlighted using red (significantly up-regulated) and blue (significantly down-regulated) node label and border colors.

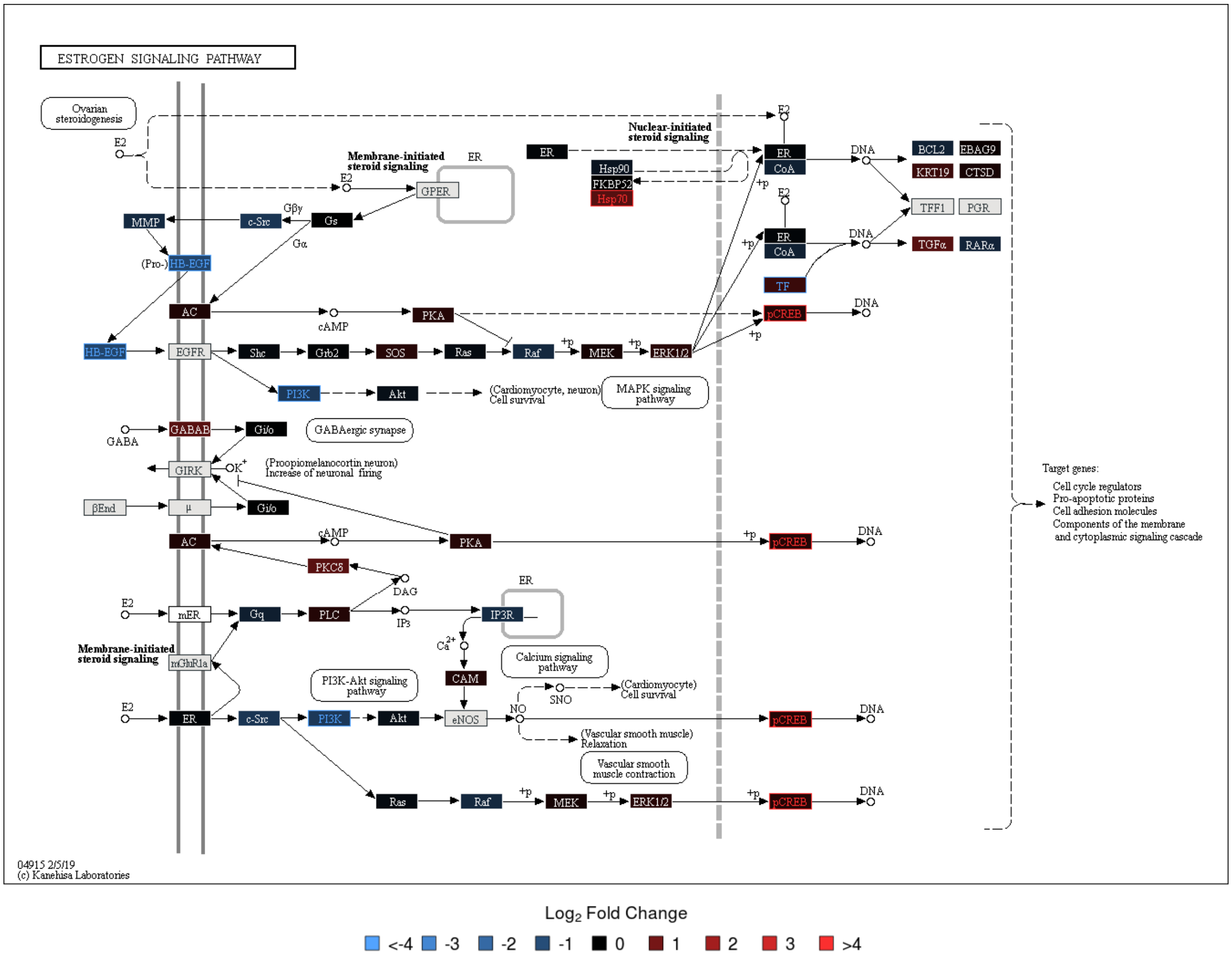

Figure S64: KEGG Pathway Map - Estrogen signaling pathway - Homo sapiens (human) (RNA-Seq, Saint Louis University, Day 7). Node color gradient encodes fold change from pre-vaccination (for multi-gene pathway nodes the median fold change is used). In red: up-regulated compared to pre-vaccination, in blue: down-regulated compared to pre-vaccination. In black: fold change close to 1, in dark grey: genes filtered out due to low overall expression, light grey: gene missing database mapping, white: non-human gene. DE genes are highlighted using red (significantly up-regulated) and blue (significantly down-regulated) node label and border colors.

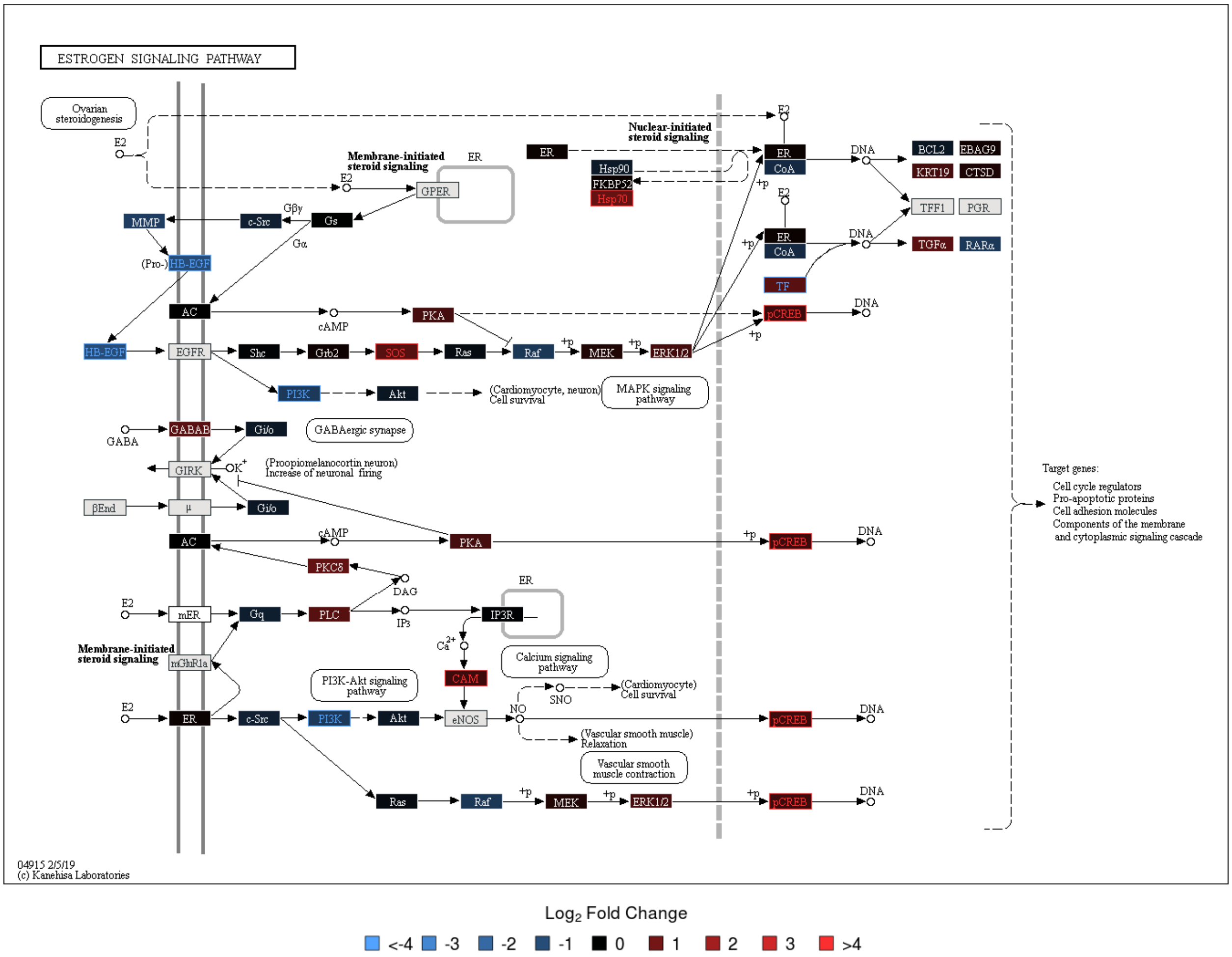

Figure S65: KEGG Pathway Map - Estrogen signaling pathway - Homo sapiens (human) (RNA-Seq, Saint Louis University, Day 14). Node color gradient encodes fold change from pre-vaccination (for multi-gene pathway nodes the median fold change is used). In red: up-regulated compared to pre-vaccination, in blue: down-regulated compared to pre-vaccination. In black: fold change close to 1, in dark grey: genes filtered out due to low overall expression, light grey: gene missing database mapping, white: non-human gene. DE genes are highlighted using red (significantly up-regulated) and blue (significantly down-regulated) node label and border colors.

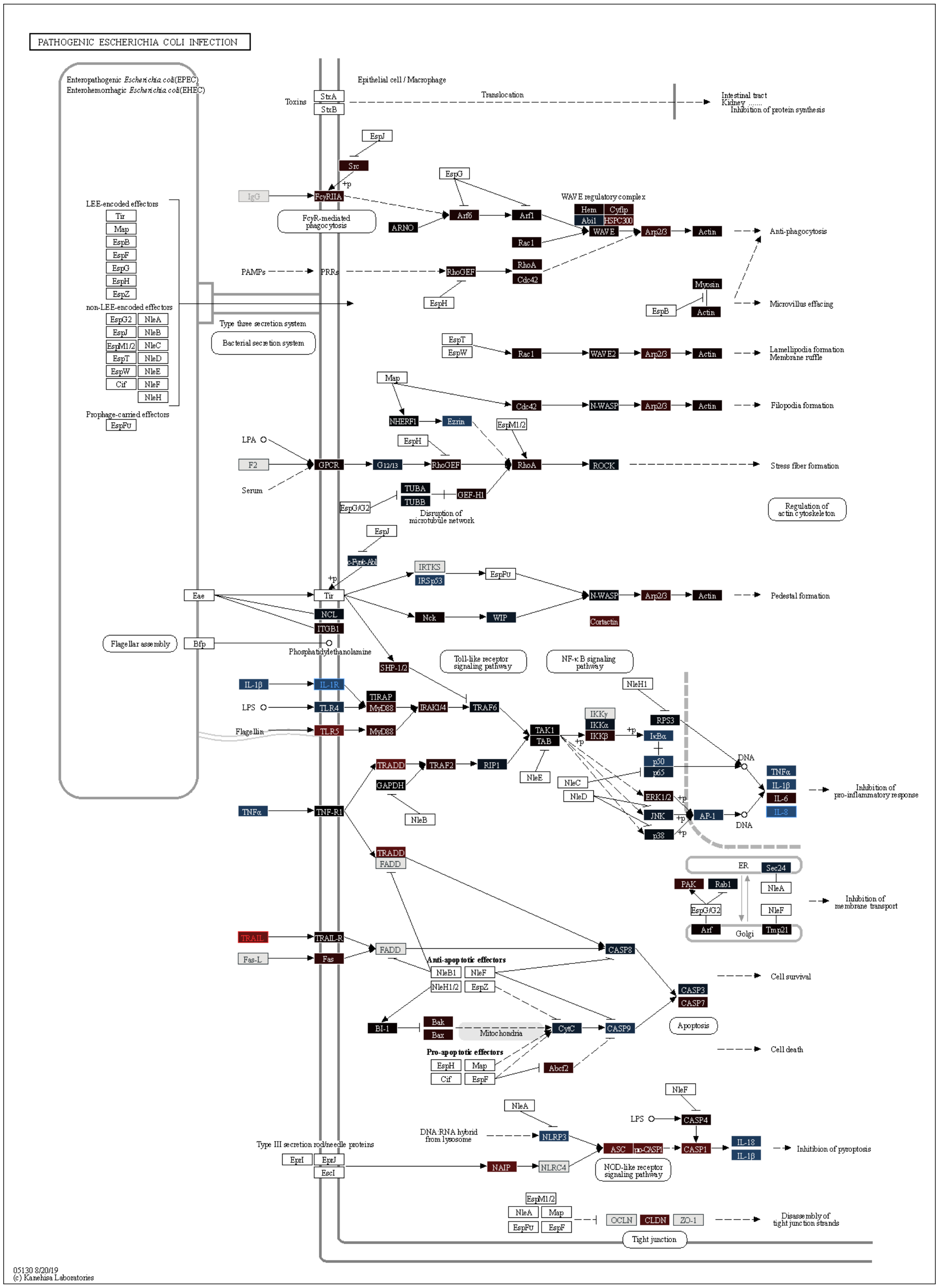

Log$_2$ Fold Change

<-4 -3 -2 -1 0 1 2 3 >4

Figure S66: KEGG Pathway Map - Pathogenic Escherichia coli infection - Homo sapiens (human) (RNA-Seq, Saint Louis University, Day 1). Node color gradient encodes fold change from pre-vaccination (for multi-gene pathway nodes the median fold change is used). In red: up-regulated compared to pre-vaccination, in blue: down-regulated compared to pre-vaccination. In black: fold change close to 1, in dark grey: genes filtered out due to low overall expression, light grey: gene missing database mapping, white: non-human gene. DE genes are highlighted using red (significantly up-regulated) and blue (significantly down-regulated) node label and border colors.

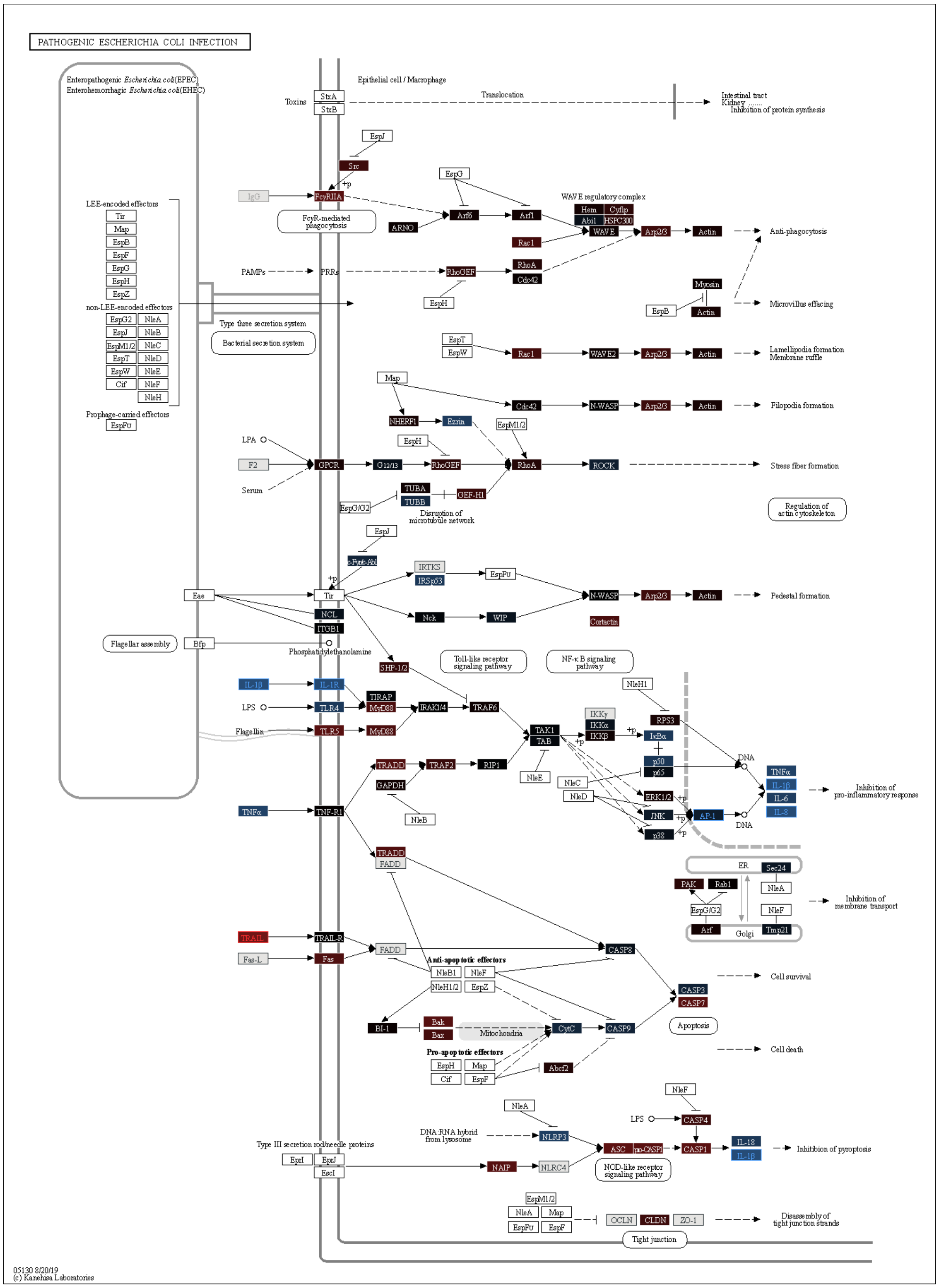

Log$_2$ Fold Change

<-4 -3 -2 -1 0 1 2 3 >4

Figure S67: KEGG Pathway Map - Pathogenic Escherichia coli infection - Homo sapiens (human) (RNA-Seq, Saint Louis University, Day 2). Node color gradient encodes fold change from pre-vaccination (for multi-gene pathway nodes the median fold change is used). In red: up-regulated compared to pre-vaccination, in blue: down-regulated compared to pre-vaccination. In black: fold change close to 1, in dark grey: genes filtered out due to low overall expression, light grey: gene missing database mapping, white: non-human gene. DE genes are highlighted using red (significantly up-regulated) and blue (significantly down-regulated) node label and border colors.

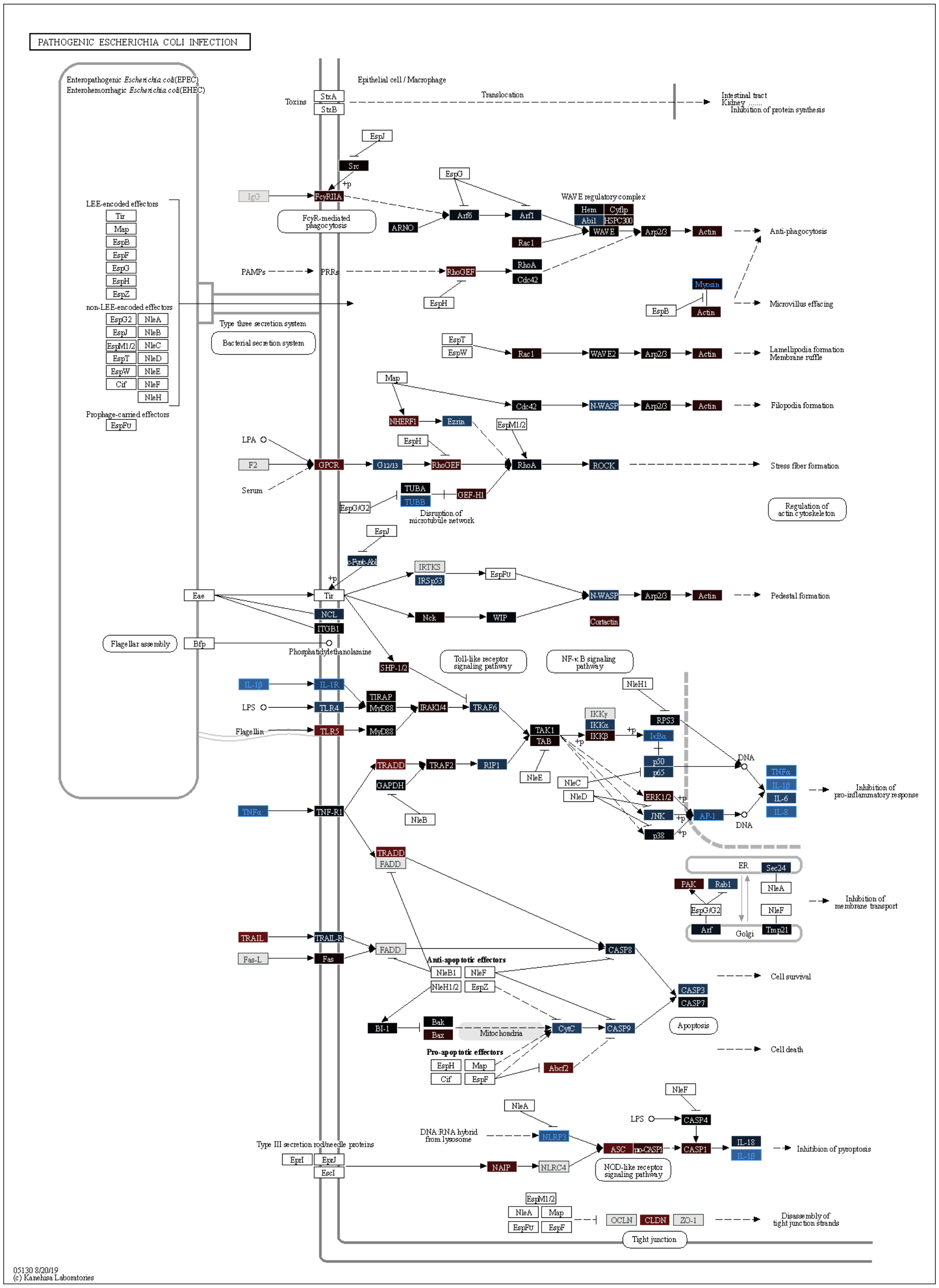

Log$_2$ Fold Change

<-4 -3 -2 -1 0 1 2 3 >4

Figure S68: KEGG Pathway Map - Pathogenic Escherichia coli infection - Homo sapiens (human) (RNA-Seq, Saint Louis University, Day 7). Node color gradient encodes fold change from pre-vaccination (for multi-gene pathway nodes the median fold change is used). In red: up-regulated compared to pre-vaccination, in blue: down-regulated compared to pre-vaccination. In black: fold change close to 1, in dark grey: genes filtered out due to low overall expression, light grey: gene missing database mapping, white: non-human gene. DE genes are highlighted using red (significantly up-regulated) and blue (significantly down-regulated) node label and border colors.

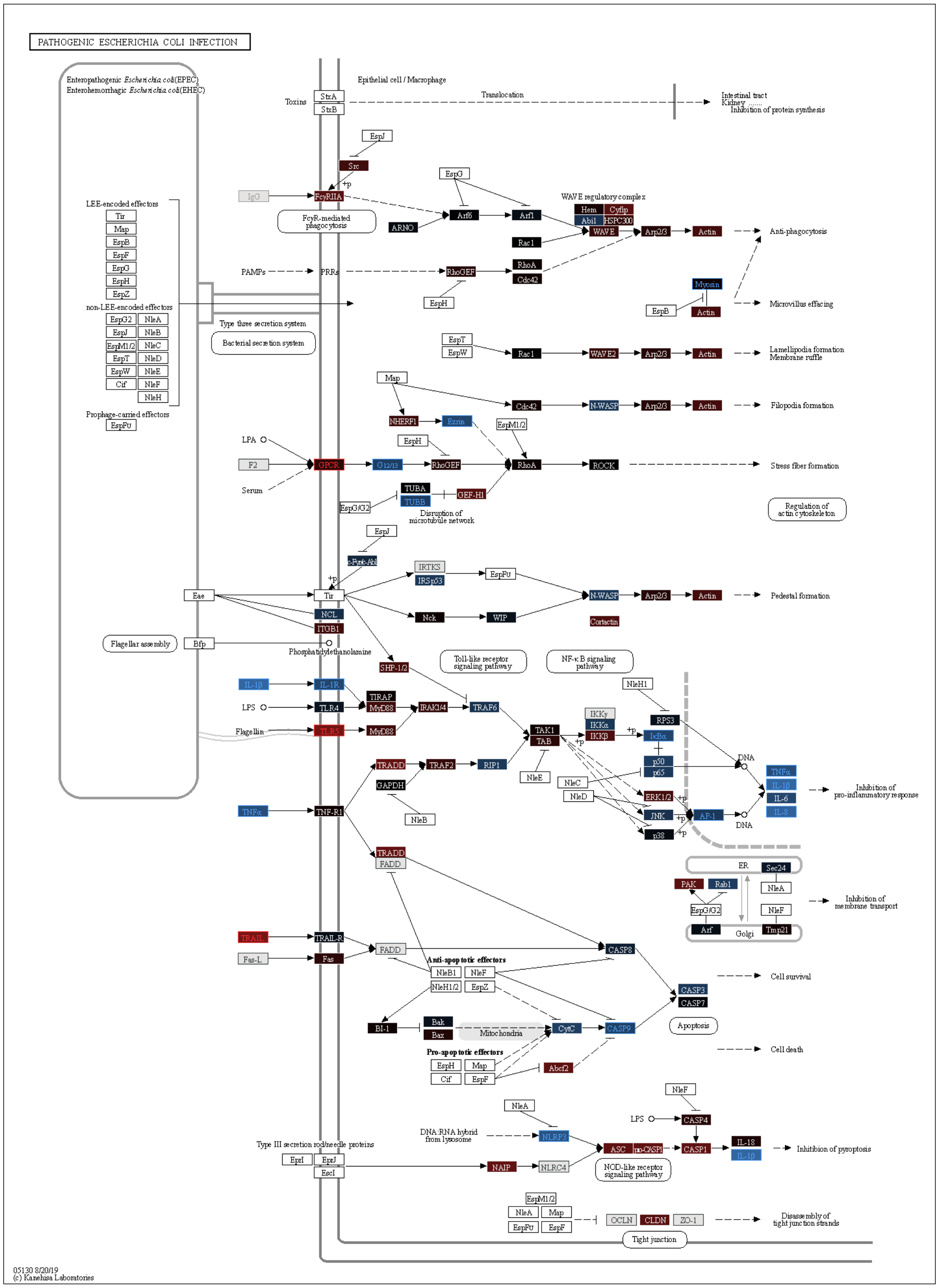

Log$_2$ Fold Change

<-4 -3 -2 -1 0 1 2 3 >4

Figure S69: KEGG Pathway Map - Pathogenic Escherichia coli infection - Homo sapiens (human) (RNA-Seq, Saint Louis University, Day 14). Node color gradient encodes fold change from pre-vaccination (for multi-gene pathway nodes the median fold change is used). In red: up-regulated compared to pre-vaccination, in blue: down-regulated compared to pre-vaccination. In black: fold change close to 1, in dark grey: genes filtered out due to low overall expression, light grey: gene missing database mapping, white: non-human gene. DE genes are highlighted using red (significantly up-regulated) and blue (significantly down-regulated) node label and border colors.

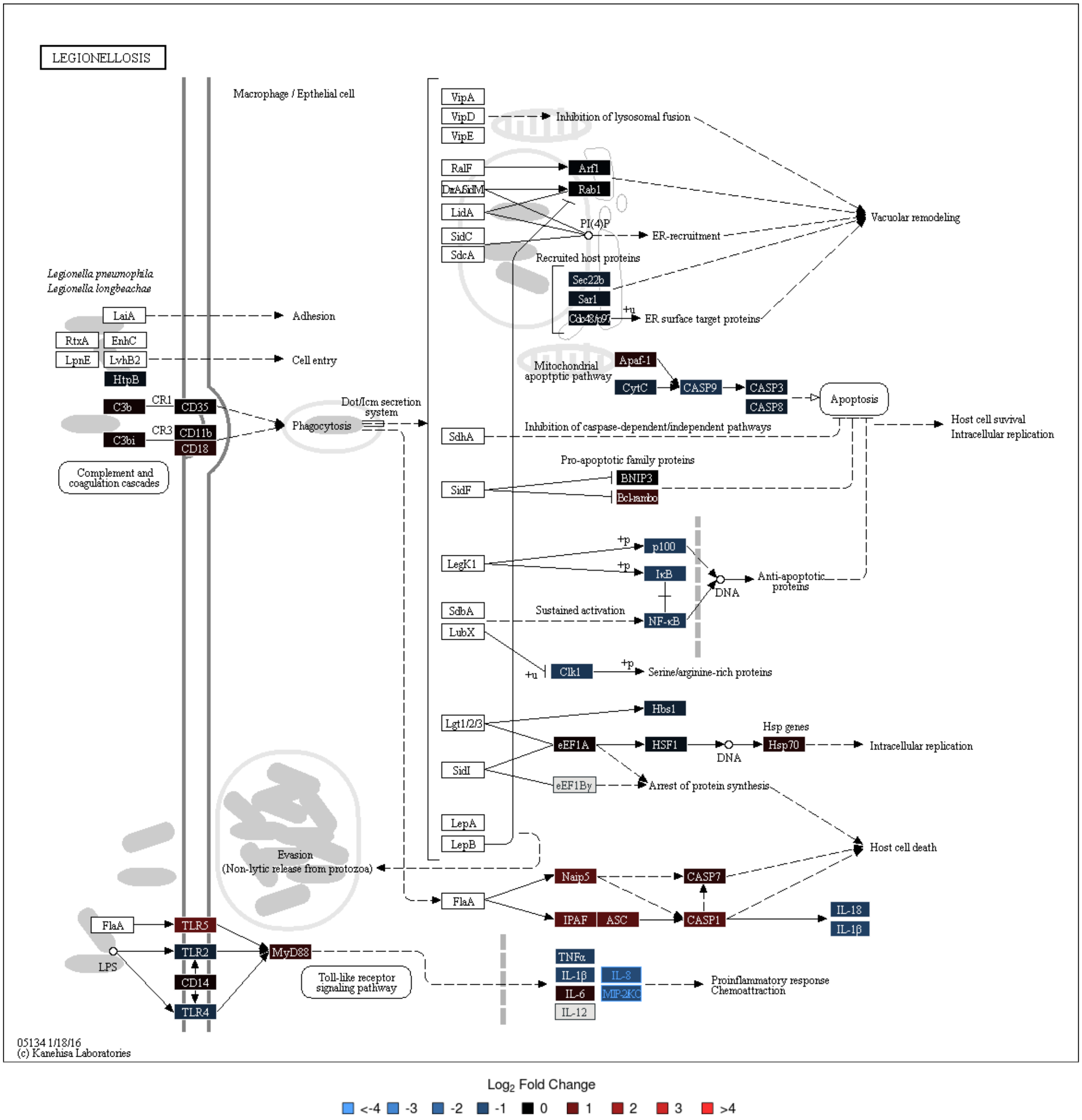

Figure S70: KEGG Pathway Map - Legionellosis - Homo sapiens (human) (RNA-Seq, Saint Louis University, Day 1). Node color gradient encodes fold change from pre-vaccination (for multi-gene pathway nodes the median fold change is used). In red: up-regulated compared to pre-vaccination, in blue: down-regulated compared to pre-vaccination. In black: fold change close to 1, in dark grey: genes filtered out due to low overall expression, light grey: gene missing database mapping, white: non-human gene. DE genes are highlighted using red (significantly up-regulated) and blue (significantly down-regulated) node label and border colors.

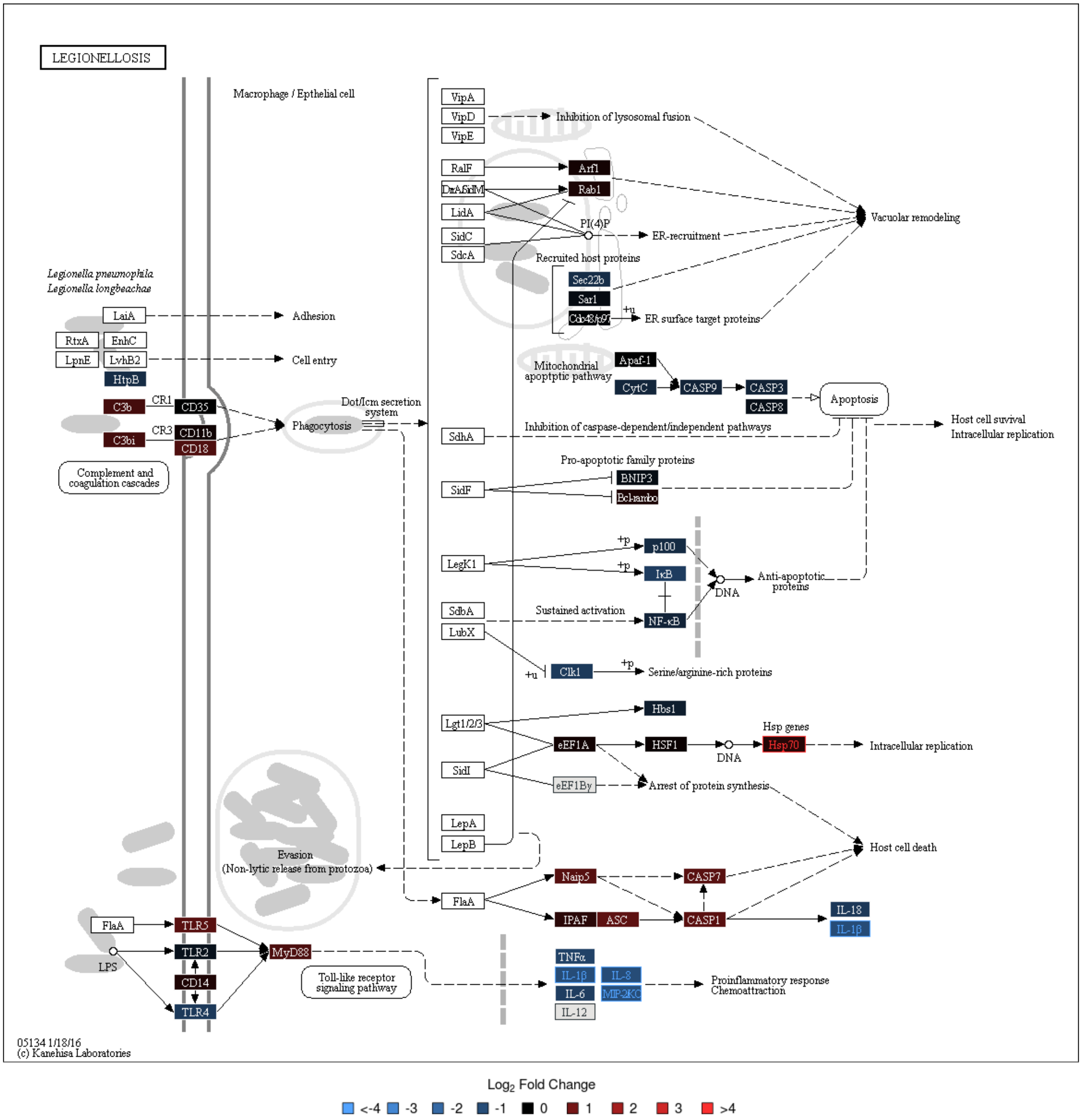

Figure S71: KEGG Pathway Map - Legionellosis - Homo sapiens (human) (RNA-Seq, Saint Louis University, Day 2). Node color gradient encodes fold change from pre-vaccination (for multi-gene pathway nodes the median fold change is used). In red: up-regulated compared to pre-vaccination, in blue: down-regulated compared to pre-vaccination. In black: fold change close to 1, in dark grey: genes filtered out due to low overall expression, light grey: gene missing database mapping, white: non-human gene. DE genes are highlighted using red (significantly up-regulated) and blue (significantly down-regulated) node label and border colors.

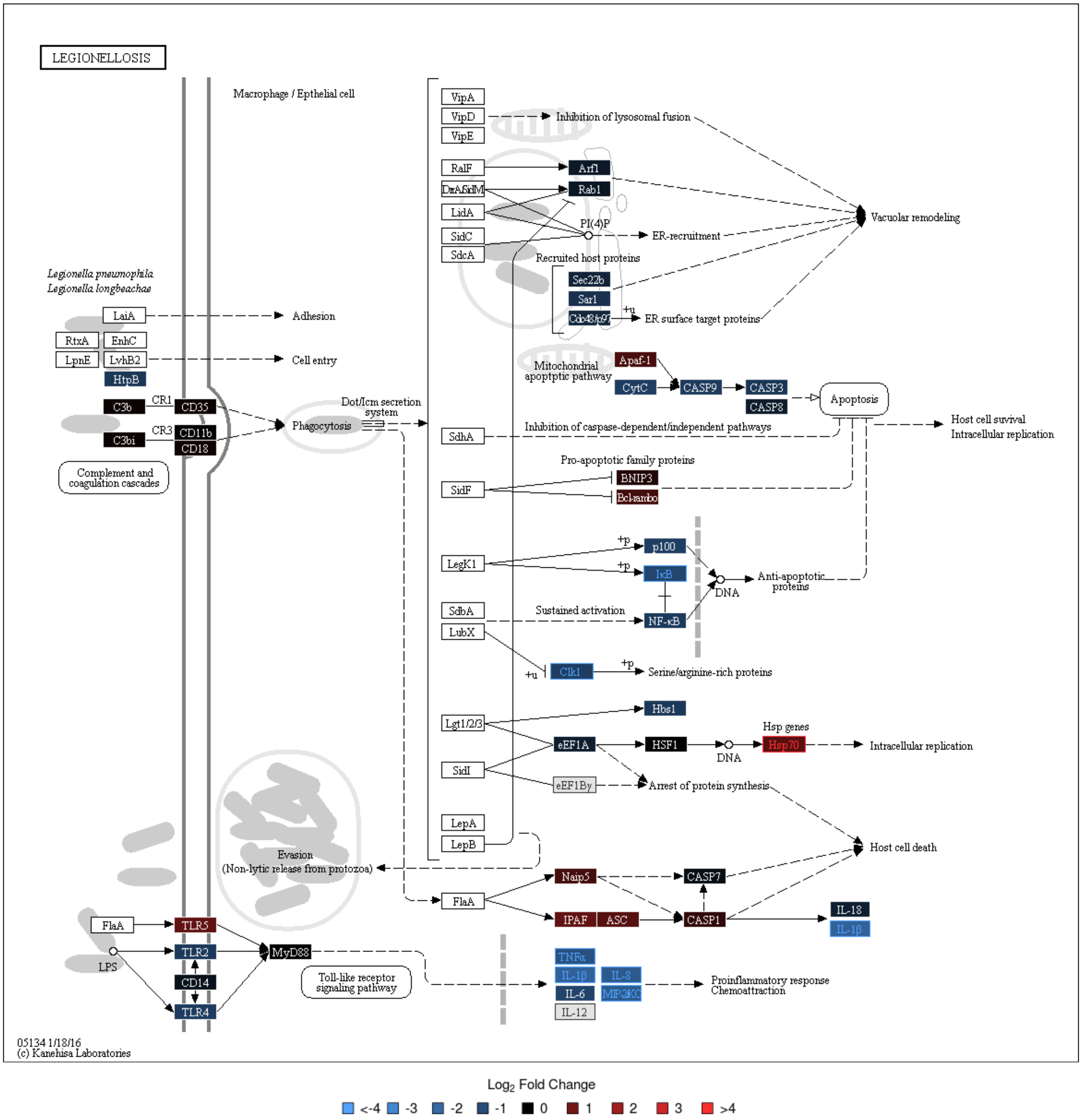

Figure S72: KEGG Pathway Map - Legionellosis - Homo sapiens (human) (RNA-Seq, Saint Louis University, Day 7). Node color gradient encodes fold change from pre-vaccination (for multi-gene pathway nodes the median fold change is used). In red: up-regulated compared to pre-vaccination, in blue: down-regulated compared to pre-vaccination. In black: fold change close to 1, in dark grey: genes filtered out due to low overall expression, light grey: gene missing database mapping, white: non-human gene. DE genes are highlighted using red (significantly up-regulated) and blue (significantly down-regulated) node label and border colors.

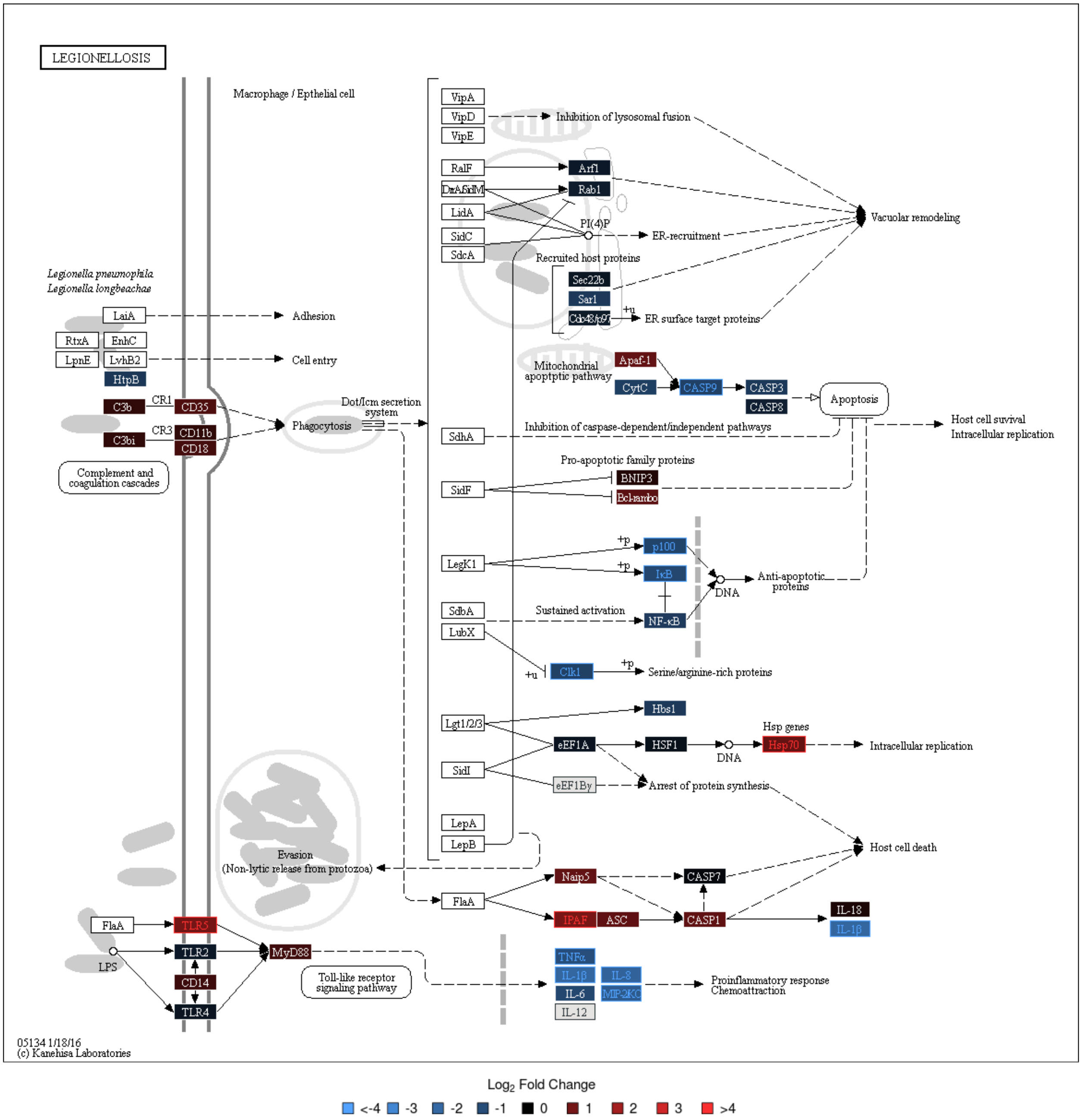

Figure S73: KEGG Pathway Map - Legionellosis - Homo sapiens (human) (RNA-Seq, Saint Louis University, Day 14). Node color gradient encodes fold change from pre-vaccination (for multi-gene pathway nodes the median fold change is used). In red: up-regulated compared to pre-vaccination, in blue: down-regulated compared to pre-vaccination. In black: fold change close to 1, in dark grey: genes filtered out due to low overall expression, light grey: gene missing database mapping, white: non-human gene. DE genes are highlighted using red (significantly up-regulated) and blue (significantly down-regulated) node label and border colors.

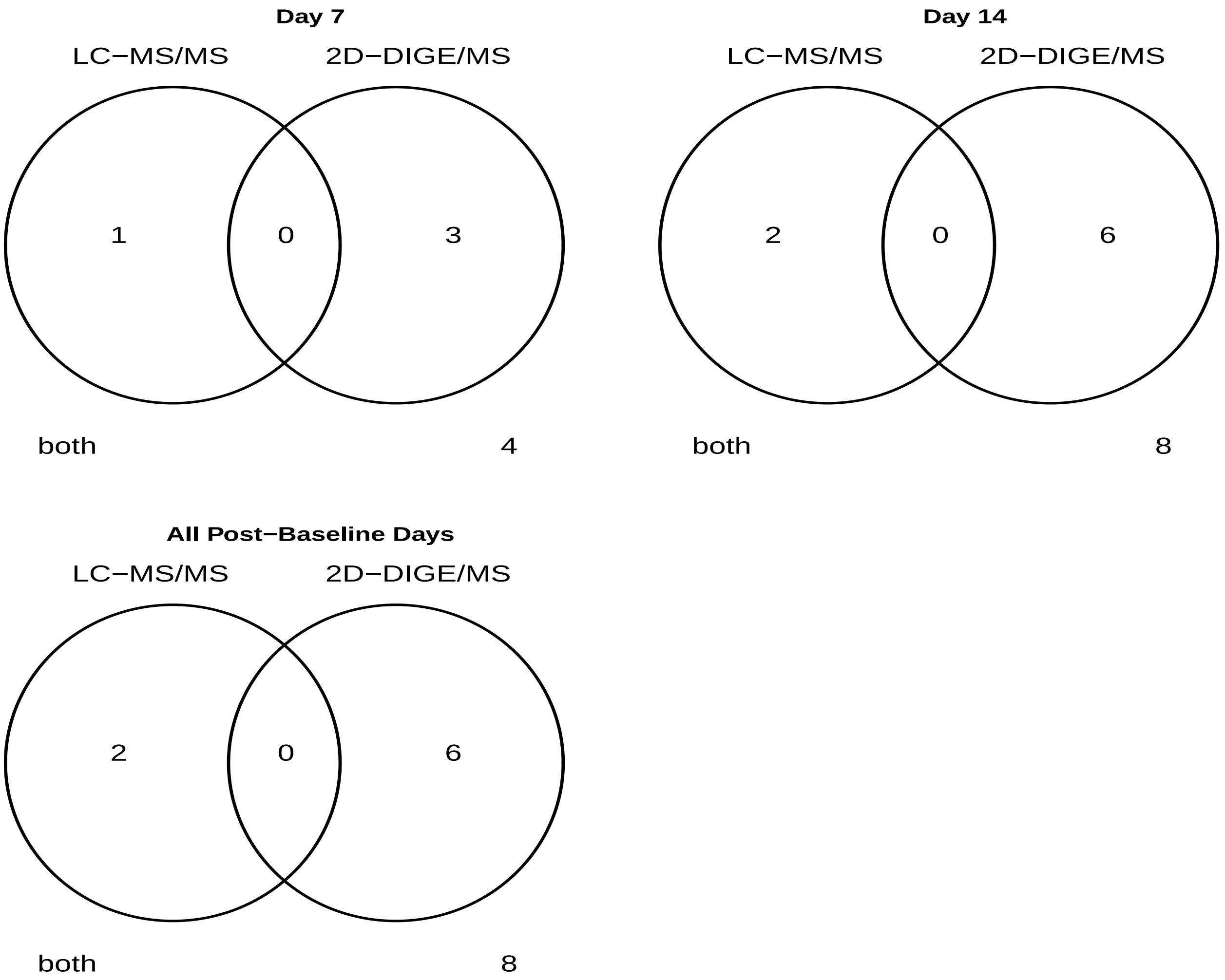

Figure S74: Venn diagrams summarizing overlap in enriched KEGG Pathways between laboratories (LC-MS/MS and 2D-DIGE/MS)

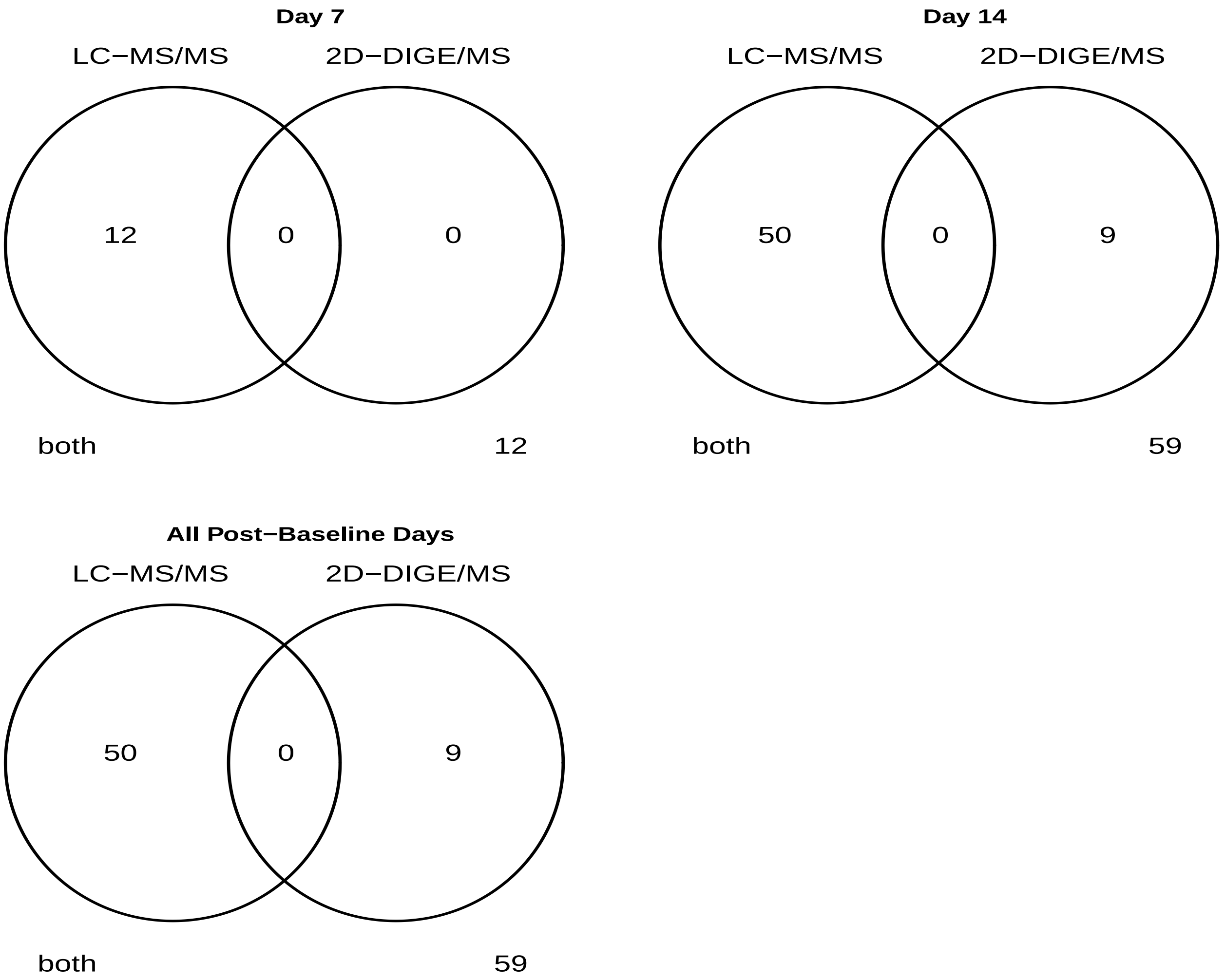

Figure S75: Venn diagrams summarizing overlap in enriched MSigDB Reactome Pathways between laboratories (LC-MS/MS and 2D-DIGE/MS)

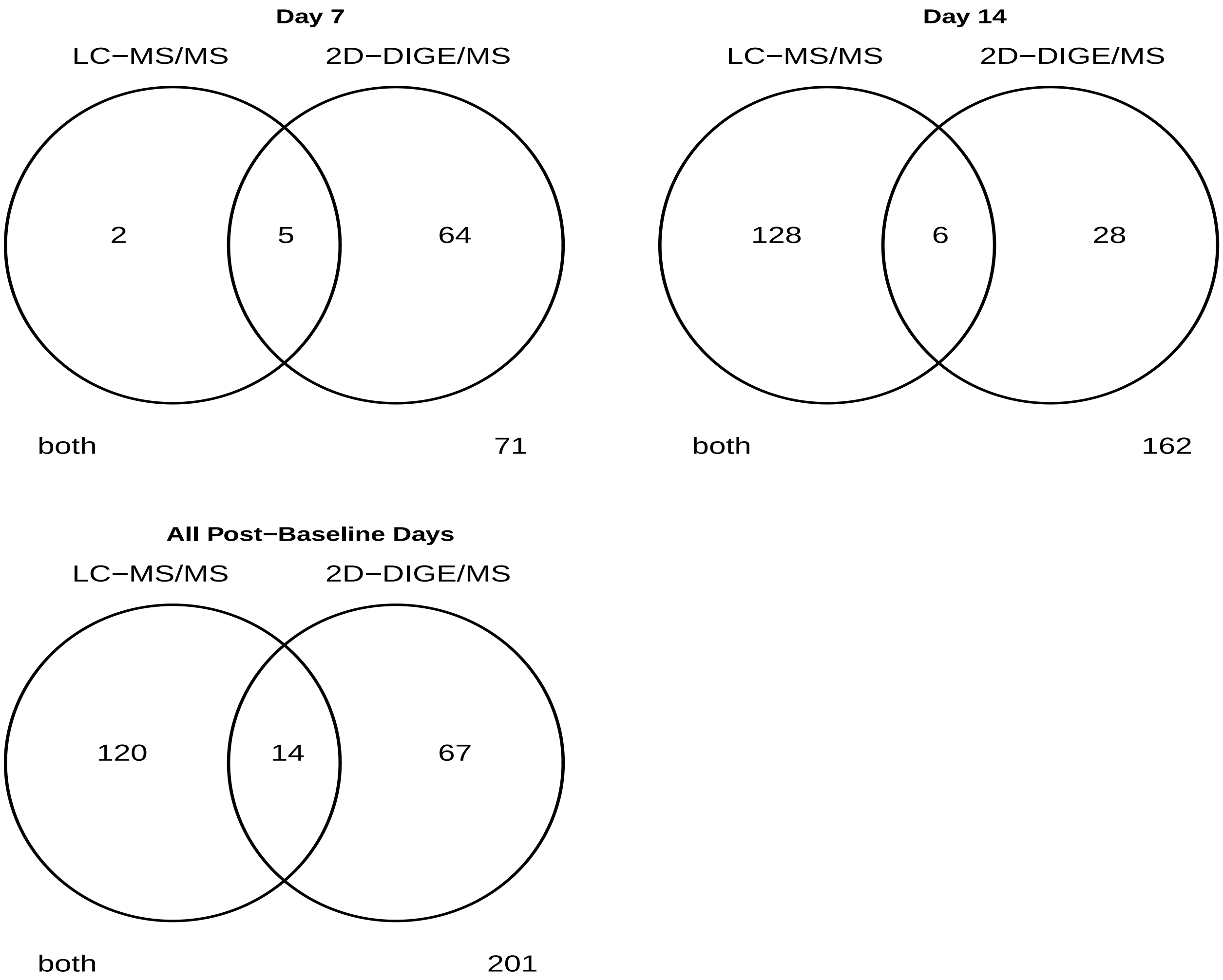

Figure S76: Venn diagrams summarizing overlap in enriched MSigDB Immunologic Signature Sets between laboratories (LC-MS/MS and 2D-DIGE/MS)

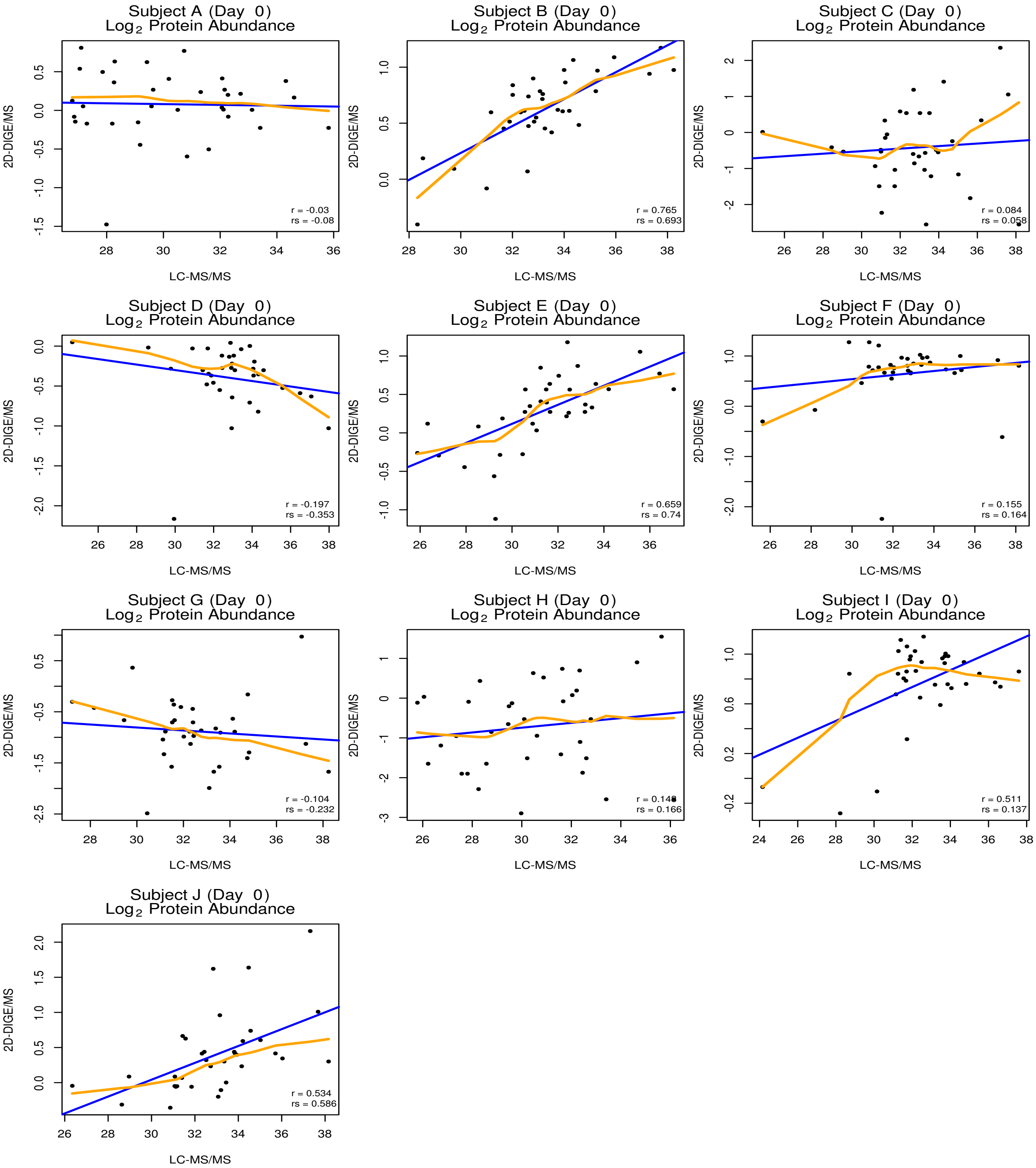

Figure S77: Scatterplots to assess correlation between laboratory log_2 protein signals (Day 0, LC-MS/MS and 2D-DIGE/MS). Each dot represents log_2 protein signal for one of 35 shared proteins; r: Pearson correlation coefficient (linear increase/decrease); rs: Spearman's rank correlation coefficient (monotonic increase/decrease); in blue: linear regression fit; in orange: locally weighted regression fit.

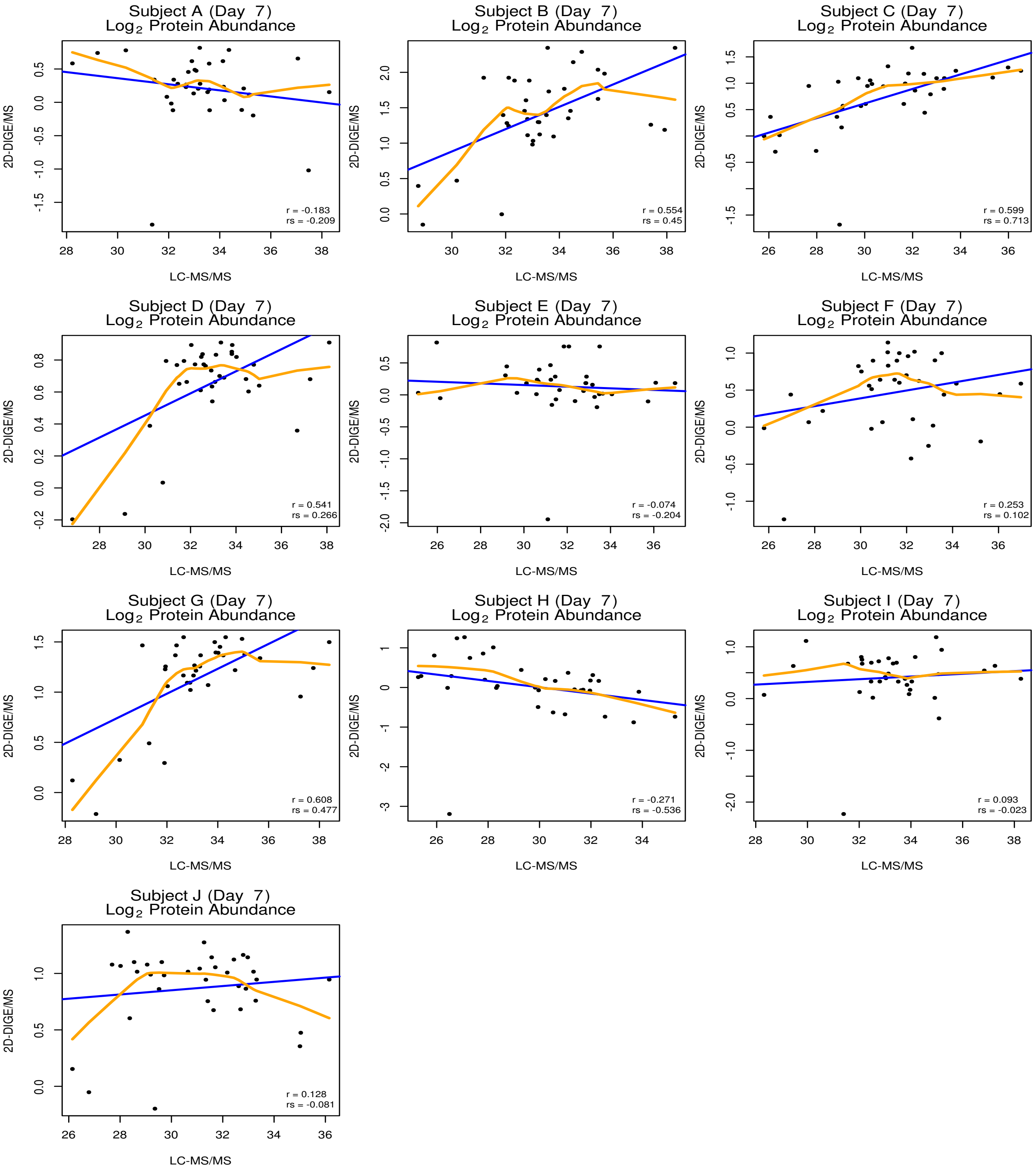

Figure S78: Scatterplots to assess correlation between laboratory log_2 protein signals (Day 7, LC-MS/MS and 2D-DIGE/MS). Each dot represents log_2 protein signal for one of 35 shared proteins; r: Pearson correlation coefficient (linear increase/decrease); rs: Spearman's rank correlation coefficient (monotonic increase/decrease); in blue: linear regression fit; in orange: locally weighted regression fit.

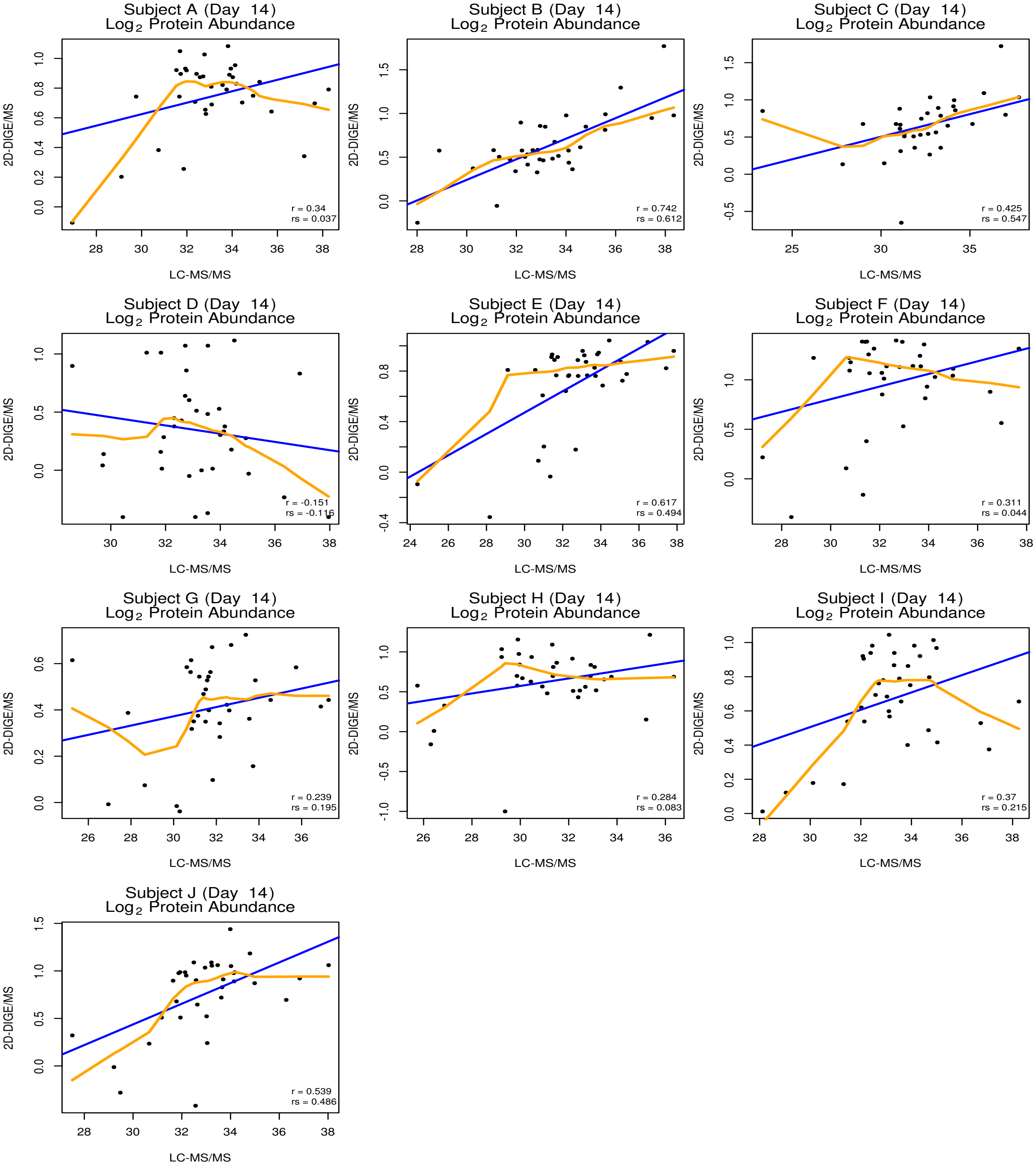

Figure S79: Scatterplots to assess correlation between laboratory log_2 protein signals (Day 14, LC-MS/MS and 2D-DIGE/MS). Each dot represents log_2 protein signal for one of 35 shared proteins; r: Pearson correlation coefficient (linear increase/decrease); rs: Spearman's rank correlation coefficient (monotonic increase/decrease); in blue: linear regression fit; in orange: locally weighted regression fit.

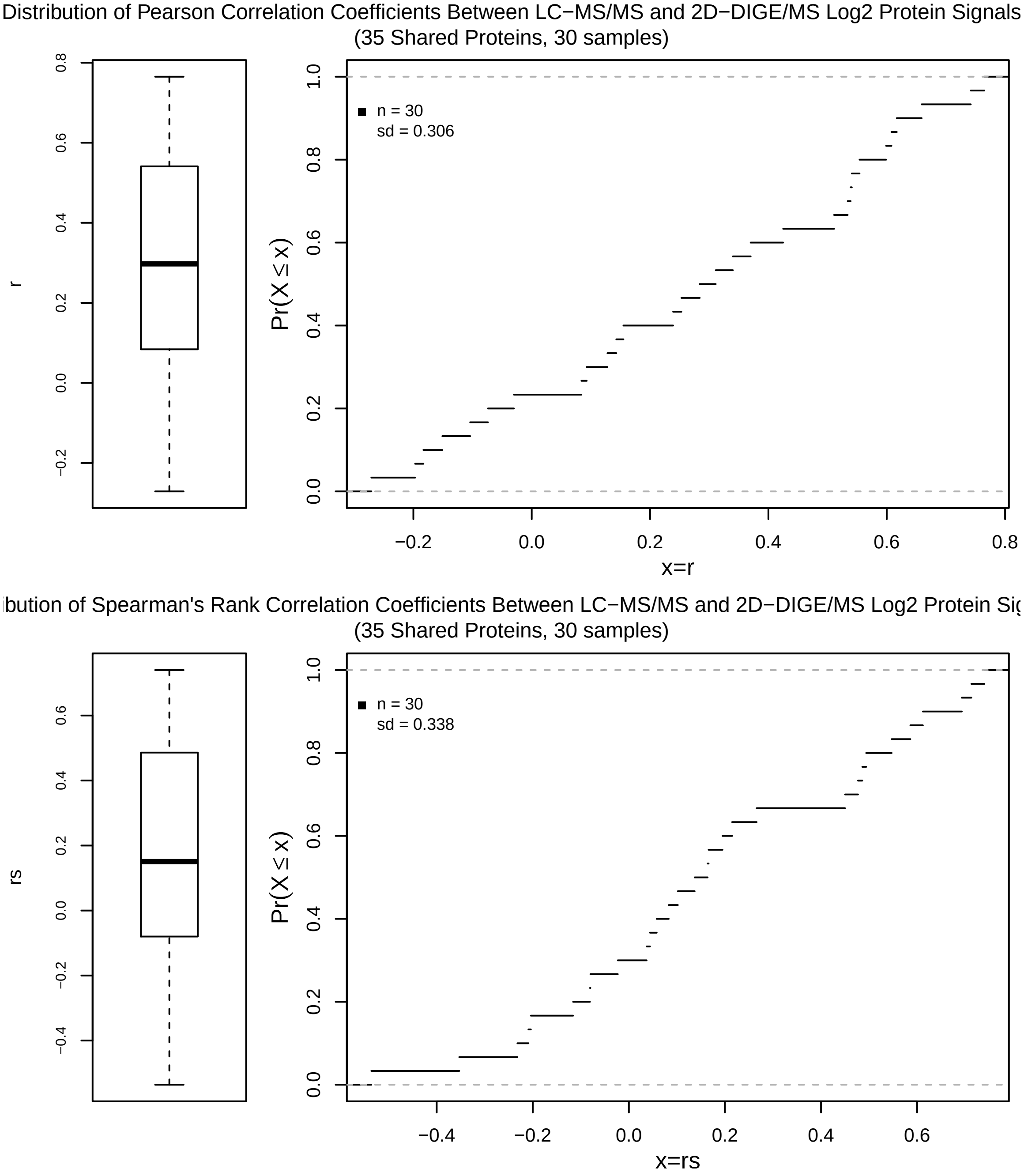

Figure S80: Boxplots and empirical cumulative distribution function plots to summarize correlation metrics between laboratory log_2 protein signals. Correlation metrics are based on 35 shared proteins collected for 30 samples. Top: Pearson correlation coefficient (linear increase/decrease); Bottom: Spearman's rank correlation coefficient (monotonic increase/decrease).

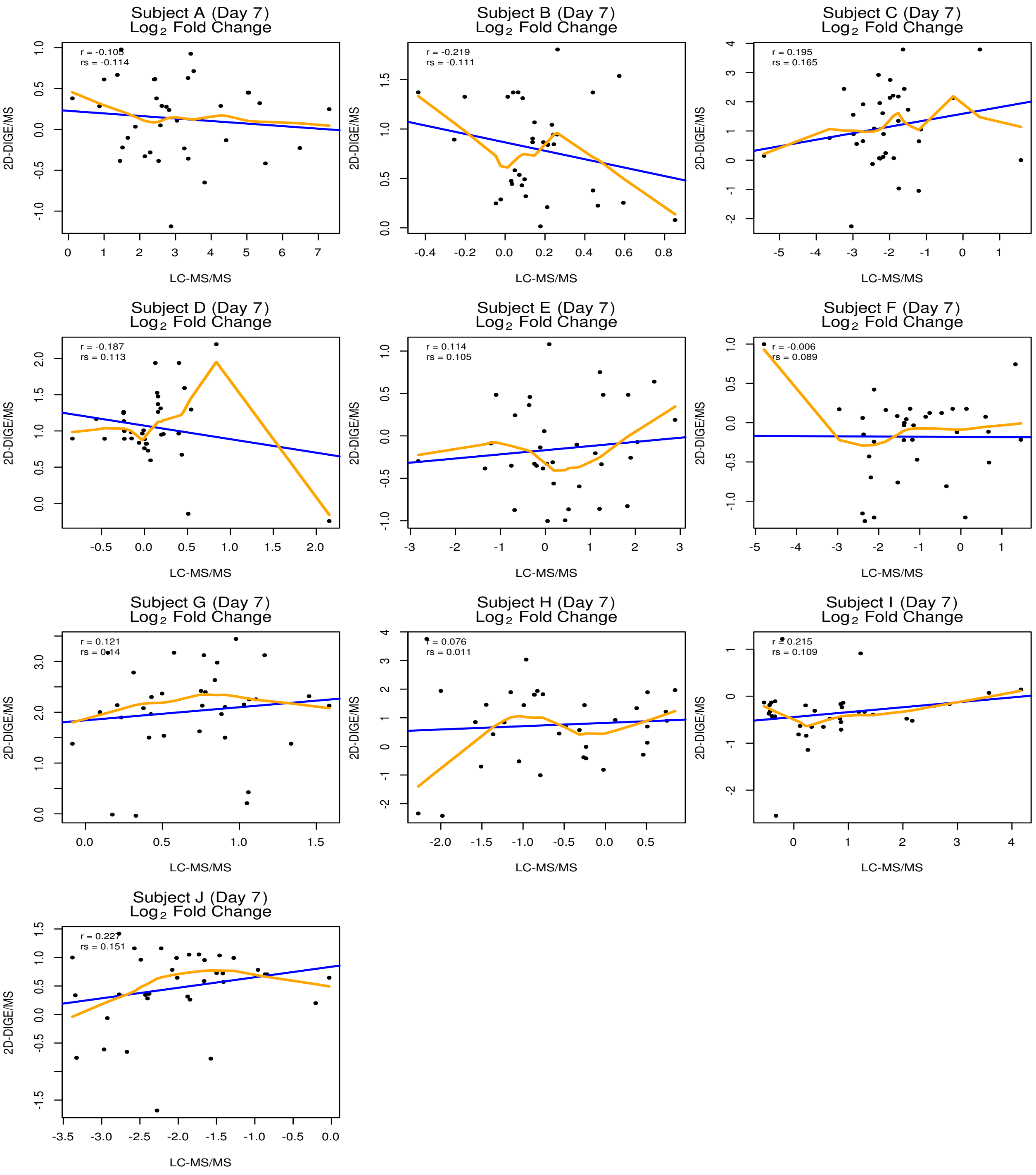

Figure S81: Scatterplots to assess correlation between laboratory log_2 fold changes (Day 7, LC-MS/MS and 2D-DIGE/MS). Each dot represents one of 35 shared proteins; r: Pearson correlation coefficient (linear increase/decrease); rs: Spearman's rank correlation coefficient (monotonic increase/decrease); in blue: linear regression fit; in orange: locally weighted regression fit.

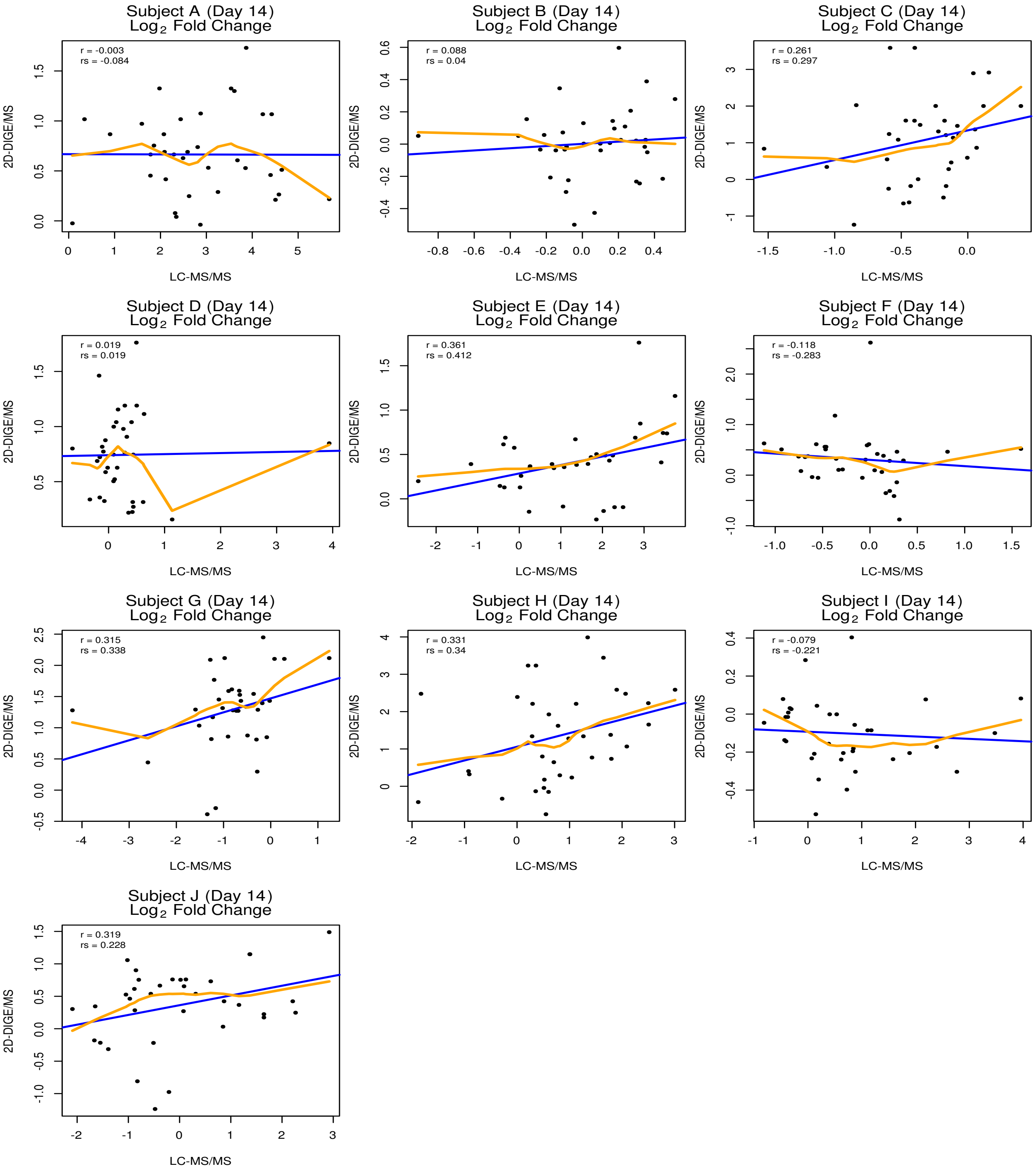

Figure S82: Scatterplots to assess correlation between laboratory log_2 fold changes (Day 14, LC-MS/MS and 2D-DIGE/MS). Each dot represents one of 35 shared proteins; r: Pearson correlation coefficient (linear increase/decrease); rs: Spearman's rank correlation coefficient (monotonic increase/decrease); in blue: linear regression fit; in orange: locally weighted regression fit.

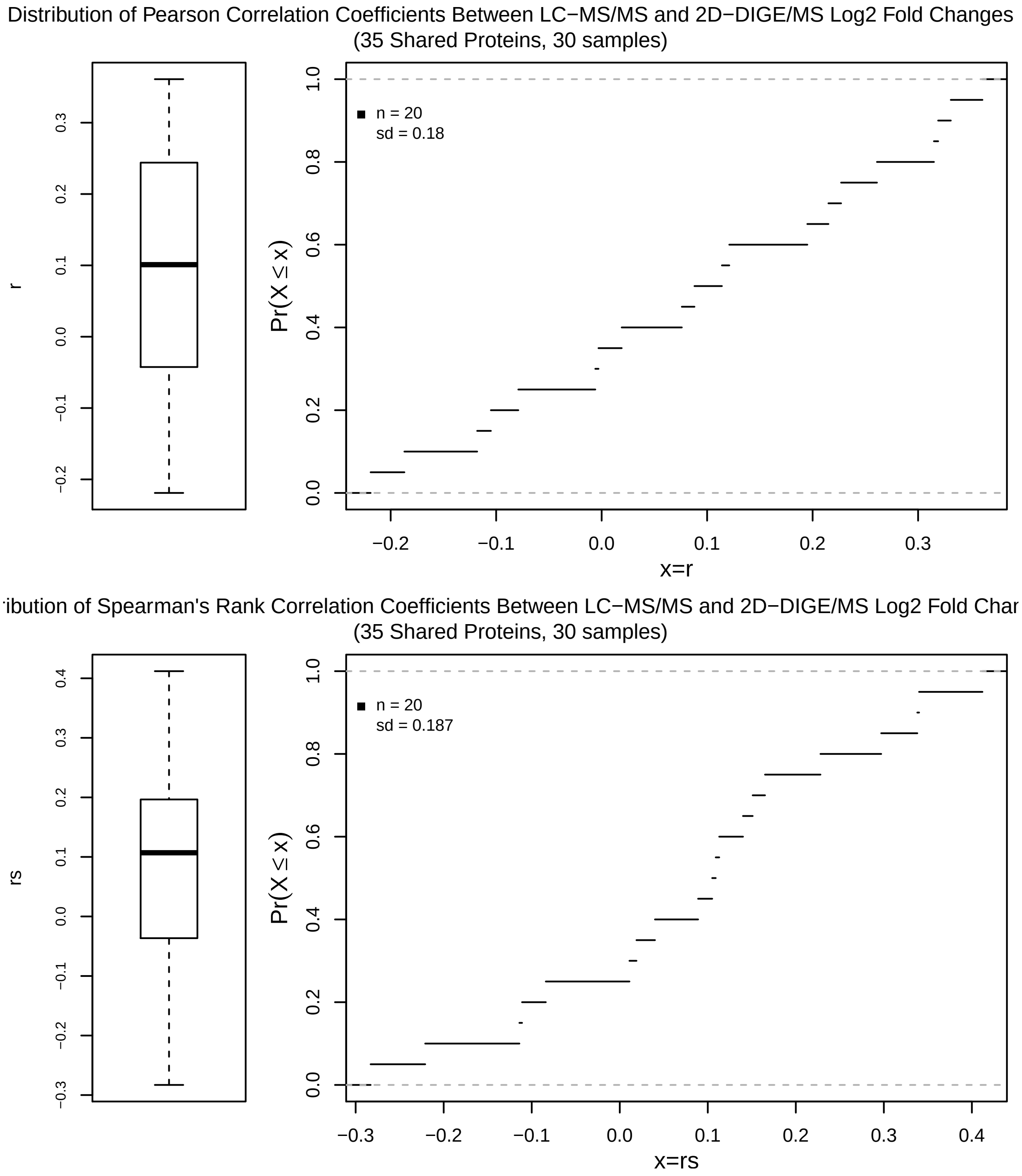

Figure S83: Boxplots and empirical cumulative distribution function plots to summarize correlation metrics between laboratory log_2 fold changes. Correlation metrics are based on 35 shared proteins collected for 30 samples. Top: Pearson correlation coefficient (linear increase/decrease); Bottom: Spearman's rank correlation coefficient (monotonic increase/decrease).

Tables

Category Type	# Gene Sets	Total #Genes	Median #Genes Per Set
KEGG Pathways	304	6493	63
MSigDB Immunologic Signature Sets	4872	16991	187
MSigDB Reactome Pathways	674	5538	26

Table S1: Overview of filtered gene sets used for the gene set enrichment analysis (LC-MS/MS and 2D-DIGE/MS). Genes sets were filtered for genes encoding for proteins present in the proteomics sequence database used by both laboratories.

	Min	Q1	Median	Mean	Q3	Max	SD	MAD
Number of Proteins	880.0	1262.8	2098.5	1871.5	2306.2	2524.0	570.7	433.7
Number of Protein Groups	752.0	1083.0	1845.5	1639.7	2034.5	2234.0	514.5	392.9
Number of Protein Families*	705.0	1008.2	1704.0	1517.0	1875.2	2064.0	470.2	364.7
Median Isoelectric Point*	6.2	6.3	6.3	6.4	6.4	6.6	0.1	0.1
Median Molecular Weight (Da)*	42.6	44.6	46.2	45.5	46.4	46.7	1.3	0.7
Median Protein Length*	380.0	400.9	413.0	407.7	417.0	419.0	11.9	7.4

Table S2: Summary statistics of proteomics sample metrics (LC-MS/MS, n=30). *: restricted to the representative protein in a protein group (leading protein). Protein families were defined at a 50% sequence identity level.

	Min	Q1	Median	Mean	Q3	Max	SD	MAD
Number of Gel Spots	599.0	696.5	719.0	748.2	780.8	1120.0	118.2	59.3

Table S3: Summary statistics of proteomics sample metrics (2D-DIGE/MS, n=30). Based on the number of spots per gel that were mapped to the master gel.

Gel Spot ID	Protein ID	Gene Name	Protein Description	50% Protein Cluster ID (Gene Name)	Mascot Search	Protein Score	Experimental MWt (kDa)	Experimental pI	Uniprot MWt (Da)	Uniprot pI
S0103	Q9Y490	TLN1	Talin-1	Q9Y4G6 (TLN2)	PMF	178	250	5.80	271766	5.77
S0103	Q9H7E2	TDRD3	Tudor domain-containing protein 3	Q9H7E2 (TDRD3)	PMF	56	250	5.80	73425	9.27
S0156	P21333	FLNA	Filamin-A	Q14315 (FLNC)	PMF	59	200	5.40	283301	5.70
S0171	Q9Y490	TLN1	Talin-1	Q9Y4G6 (TLN2)	PMF	110	200	5.70	271766	5.77
S0400	P21333	FLNA	Filamin-A	Q14315 (FLNC)	PMF	122	90	6.20	283301	5.70
S0400	Q12774	ARHGEF5	Rho guanine nucleotide exchange factor 5	Q12774 (ARHGEF5)	PMF	62	90	6.20	177888	5.37
S0400	P21333	FLNA	Filamin-A	Q14315 (FLNC)	PMF	57	90	6.20	283301	5.70
S0405	Q9Y490	TLN1	Talin-1	Q9Y4G6 (TLN2)	PMF	88	85	6.20	271766	5.77
S0412	P14625	HSP90B1	Endoplasmin	P14625 (HSP90B1)	PMF	131	80	4.40	92696	4.76
S0418	P55072	VCP	Transitional endoplasmic reticulum ATPase	P55072 (VCP)	PMF	184	85	5.00	89950	5.14
S0445	P06396	GSN	Gelsolin	P06396 (GSN)	PMF	73	80	6.00	86043	5.90
S0452	P07900	HSP90AA1	Heat shock protein HSP 90-alpha	P07900 (HSP90AA1)	PMF	72	72	4.80	85006	4.94
S0452	P08238	HSP90AB1	Heat shock protein HSP 90-beta	P07900 (HSP90AA1)	PMF	62	72	4.80	83554	4.97
S0513	P11021	HSPA5	78 kDa glucose-regulated protein	P38646 (HSPA9)	PMF	146	75	4.60	72402	5.07
S0513	P11021	HSPA5	78 kDa glucose-regulated protein	P38646 (HSPA9)	PMF	135	75	4.60	72402	5.07
S0515	P11142	HSPA8	Heat shock cognate 71 kDa protein	P11142 (HSPA8)	PMF	213	68	5.30	71082	5.37
S0522	P02768	ALB	Serum albumin	P02768 (ALB)	PMF	113	68	6.40	71317	5.92
S0522	P02768	ALB	Serum albumin	P02768 (ALB)	PMF	69	68	6.30	71317	5.92
S0524	Q9H2C0	GAN	Gigaxonin	Q9H2C0 (GAN)	PMF	59	90	8.80	68678	5.58
S0535	P0DMV8	HSPA1A	Heat shock 70 kDa protein 1A ECO:0000312|HGNC:HGNC:5232	P11142 (HSPA8)	PMF	154	70	5.60	70294	5.48
S0536	Q15833	STXBP2	Syntaxin-binding protein 2	P61764 (STXBP1)	PMF	66	65	7.30	66867	6.11
S0544	P13796	LCP1	Plastin-2	P13797 (PLS3)	PMF	165	70	5.00	70814	5.29
S0585	P30101	PDIA3	Protein disulfide-isomerase A3	P30101 (PDIA3)	PMF	86	60	5.90	57146	5.98
S0588	P13645	KRT10	Keratin, type I cytoskeletal 10	P35527 (KRT9)	PMF	62	60	4.00	59020	5.13
S0588	P33176	KIF5B	Kinesin-1 heavy chain	Q12840 (KIF5A)	PMF	61	60	4.00	110358	6.12
S0598	P02675	FGB	Fibrinogen beta chain	P02675 (FGB)	PMF	70	60	8.70	56577	8.54
S0602	P07237	P4HB	Protein disulfide-isomerase	P07237 (P4HB)	PMF	185	60	4.30	57480	4.76
S0623	P27797	CALR	Calreticulin	P27797 (CALR)	PMF	62	55	3.60	48283	4.29
S0638	P68363	TUBA1B	Tubulin alpha-1B chain	Q71U36 (TUBA1A)	PMF	107	55	4.80	50804	4.94
S0638	Q9BQE3	TUBA1C	Tubulin alpha-1C chain	Q71U36 (TUBA1A)	PMF	91	55	4.80	50548	4.96
S0638	Q71U36	TUBA1A	Tubulin alpha-1A chain	Q71U36 (TUBA1A)	PMF	76	55	4.80	50788	4.94
S0638	Q13748	TUBA3D	Tubulin alpha-3C/D chain	Q71U36 (TUBA1A)	PMF	62	55	4.80	50612	4.97
S0638	Q6PEY2	TUBA3E	Tubulin alpha-3E chain	Q71U36 (TUBA1A)	PMF	62	55	4.80	50568	5.00
S0638	Q9NY65	TUBA8	Tubulin alpha-8 chain	Q71U36 (TUBA1A)	PMF	60	55	4.80	50746	4.94
S0650	P13489	RNH1	Ribonuclease inhibitor	P13489 (RNH1)	PMF	99	50	4.20	51766	4.71
S0651	P07437	TUBB	Tubulin beta chain	Q9H4B7 (TUBB1)	PMF	182	50	4.70	50095	4.78
S0651	P68371	TUBB4B	Tubulin beta-4B chain	Q9H4B7 (TUBB1)	PMF	106	50	4.70	50255	4.79
S0651	P04350	TUBB4A	Tubulin beta-4A chain	Q9H4B7 (TUBB1)	PMF	105	50	4.70	50010	4.78
S0651	Q13885	TUBB2A	Tubulin beta-2A chain	Q9H4B7 (TUBB1)	PMF	91	50	4.70	50274	4.78
S0651	Q9BVA1	TUBB2B	Tubulin beta-2B chain	Q9H4B7 (TUBB1)	PMF	91	50	4.70	50377	4.78
S0651	Q13509	TUBB3	Tubulin beta-3 chain	Q9H4B7 (TUBB1)	PMF	84	50	4.70	50856	4.83
S0661	P61158	ACTR3	Actin-related protein 3	P61158 (ACTR3)	PMF	154	50	5.80	47797	5.61
S0662	P06733	ENO1	Alpha-enolase	P13929 (ENO3)	PMF	315	50	8.30	47481	7.01
S0669	P07355	ANXA2	Annexin A2	P04083 (ANXA1)	PMF	153	52	7.90	38808	7.57
S0669	P06733	ENO1	Alpha-enolase	P13929 (ENO3)	PMF	89	55	8.00	47481	7.01
S0669	P49411	TUFM	Elongation factor Tu, mitochondrial	P49411 (TUFM)	PMF	70	55	8.00	49852	7.26
S0678	P08670	VIM	Vimentin	P41219 (PRPH)	PMF	122	48	5.00	53676	5.06
S0728	P60709	ACTB	Actin, cytoplasmic 1	A5A3E0 (POTEF)	PMF	141	40	5.00	42052	5.29
S0728	P63261	ACTG1	Actin, cytoplasmic 2	A5A3E0 (POTEF)	PMF	141	40	5.00	42108	5.31
S0729	P60709	ACTB	Actin, cytoplasmic 1	A5A3E0 (POTEF)	PMF	74	40	5.20	42052	5.29
S0729	P63261	ACTG1	Actin, cytoplasmic 2	A5A3E0 (POTEF)	PMF	74	40	5.20	42108	5.31
S0771	Q9H6N6	MYH16	Putative uncharacterized protein MYH16 ECO:0000305	Q9H6N6 (MYH16)	PMF	69	35	4.20	128439	5.40

Gel Spot ID	Protein ID	Gene Name	Protein Description	50% Protein Cluster ID (Gene Name)	Mascot Search	Protein Score	Experimental MWt (kDa)	Experimental pI	Uniprot MWt (Da)	Uniprot pI
S0771	Q9UBV4	WNT16	Protein Wnt-16	Q9UBV4 (WNT16)	PMF	68	35	4.20	42088	8.97
S0790	P52907	CAPZA1	F-actin-capping protein subunit alpha-1	P52907 (CAPZA1)	PMF	83	35	5.60	33073	5.45
S0790	Q8N807	PDILT	Protein disulfide-isomerase-like protein of the testis	Q8N807 (PDILT)	PMF	64	35	5.60	66786	6.41
S0828	P47756	CAPZB	F-actin-capping protein subunit beta	P47756 (CAPZB)	PMF	88	30	6.00	31616	5.36
S0828	Q06323	PSME1	Proteasome activator complex subunit 1	Q06323 (PSME1)	PMF	72	30	6.00	28876	5.78
S0828	Q6ZU80	CEP128	Centrosomal protein of 128 kDa	Q6ZU80 (CEP128)	PMF	63	30	6.00	128565	6.11
S0861	P08758	ANXA5	Annexin A5	P08133 (ANXA6)	PMF	94	30	4.70	35971	4.94
S0887	P67936	TPM4	Tropomyosin alpha-4 chain	P06753 (TPM3)	PMF	87	27	4.00	28619	4.67
S0911	P63104	YWHAZ	14-3-3 protein zeta/delta	P62258 (YWHAE)	PMF	88	25	4.20	27899	4.73
S0917	P60174	TPI1	Triosephosphate isomerase	P60174 (TPI1)	PMF	92	25	8.20	31057	5.65
S0933	P04264	KRT1	Keratin, type II cytoskeletal 1	P04264 (KRT1)	PMF	114	25	6.00	66170	8.15
S0933	P35527	KRT9	Keratin, type I cytoskeletal 9	P35527 (KRT9)	PMF	64	25	6.00	62255	5.14
S0934	P52566	ARHGDIB	Rho GDP-dissociation inhibitor 2	Q99819 (ARHGDIG)	PMF	88	21	4.90	23031	5.10
S0958	P25787	PSMA2	Proteasome subunit alpha type-2	P25787 (PSMA2)	PMF	91	20	8.70	25996	6.92
S0958	O95376	ARIH2	E3 ubiquitin-protein ligase ARIH2	O95376 (ARIH2)	PMF	64	20	8.70	59378	5.40
S1007	P04179	SOD2	Superoxide dismutase [Mn], mitochondrial	P04179 (SOD2)	PMF, MS2	64	20	8.50	24878	8.35

Table S4: Identified proteins (2D-DIGE/MS). Protein annotations are based on UniProt anotations (March 16, 2016). Entries with the same Gel Spot ID were sampled multiple times.

Protein ID	Gene Name	Protein Description	50% Protein Cluster ID (Gene Name)	Log_2 Fold Change (Day 7 vs. Day 0)	t-Statistic	P-value	FDR Adjusted P-value	Protein Group Protein IDs	Protein Group Gene Ids	Protein Group Gene Names
P02549	SPTA1	Spectrin alpha chain, erythrocytic 1	P02549 (SPTA1)	-2.703	-2.930	0.0176	>0.9999			
P20810	CAST	Calpastatin	P20810 (CAST)	1.380	2.380	0.0430	>0.9999			
P43405	SYK	Tyrosine-protein kinase SYK	P43405 (SYK)	1.129	2.972	0.0195	>0.9999	P43405<br>Q8NFD2	ENSG00000165025<br>ENSG00000170209	SYK<br>ANKK1
P33176	KIF5B	Kinesin-1 heavy chain	Q12840 (KIF5A)	1.110	2.521	0.0215	>0.9999	P33176<br>O60282<br>Q12840	ENSG00000170759<br>ENSG00000168280<br>ENSG00000155980<br>ENSG00000276734	KIF5B<br>KIF5C<br>KIF5A
Q15717	ELAVL1	ELAV-like protein 1	Q15717 (ELAVL1)	0.864	5.047	0.0020	>0.9999			
P60842	EIF4A1	Eukaryotic initiation factor 4A-I	P38919 (EIF4A3)	0.852	2.589	0.0078	>0.9999			
P62277	RPS13	40S ribosomal protein S13	P62277 (RPS13)	0.771	3.334	0.0137	>0.9999			
P51692	STAT5B	Signal transducer and activator of transcription 5B	P42229 (STAT5A)	0.733	2.713	0.0293	>0.9999			
P23193	TCEA1	Transcription elongation factor A protein 1	P23193 (TCEA1)	0.716	2.724	0.0273	>0.9999	P23193<br>Q15560	ENSG00000171703<br>ENSG00000187735	TCEA1<br>TCEA2
P63220	RPS21	40S ribosomal protein S21	P63220 (RPS21)	0.692	2.796	0.0137	>0.9999			
P17612	PRKACA	cAMP-dependent protein kinase catalytic subunit alpha	P22694 (PRKACB)	0.628	1.964	0.0488	>0.9999			
P84090	ERH	Enhancer of rudimentary homolog	P84090 (ERH)	0.613	3.485	0.0137	>0.9999			
P07305	H1F0	Histone H1.0	P07305 (H1F0)	0.594	2.503	0.0195	>0.9999			
O00193	SMAP	Small acidic protein	O00193 (SMAP)	0.593	2.739	0.0215	>0.9999			
O95319	CELF2	CUGBP Elav-like family member 2	O95319 (CELF2)	0.540	2.539	0.0312	>0.9999	O95319<br>Q92879	ENSG00000149187<br>ENSG00000048740	CELF2<br>CELF1
P09651	HNRNPA1	Heterogeneous nuclear ribonucleoprotein A1	P51991 (HNRNPA3)	0.539	1.950	0.0449	>0.9999	P09651<br>Q32P51	ENSG00000135486<br>ENSG00000139675	HNRNPA1<br>HN-RNPA1L2
P55735	SEC13	Protein SEC13 homolog	P55735 (SEC13)	0.537	2.488	0.0254	>0.9999			
P27694	RPA1	Replication protein A 70 kDa DNA-binding subunit	P27694 (RPA1)	0.522	2.208	0.0195	>0.9999			
O75396	SEC22B	Vesicle-trafficking protein SEC22b	O75396 (SEC22B)	0.510	3.813	0.0078	>0.9999			
P62913	RPL11	60S ribosomal protein L11	P62913 (RPL11)	0.474	2.497	0.0195	>0.9999			
Q9Y5B9	SUPT16H	FACT complex subunit SPT16	Q9Y5B9 (SUPT16H)	0.465	2.333	0.0488	>0.9999			
P62854	RPS26	40S ribosomal protein S26	P62854 (RPS26)	0.410	2.513	0.0312	>0.9999			
P62269	RPS18	40S ribosomal protein S18	P62269 (RPS18)	0.399	2.163	0.0254	>0.9999			
O00217	NDUFS8	NADH dehydrogenase [ubiquinone] iron-sulfur protein 8, mitochondrial	O00217 (NDUFS8)	-0.370	-2.850	0.0059	>0.9999			
Q5HYK7	SH3D19	SH3 domain-containing protein 19	Q5HYK7 (SH3D19)	-0.355	-2.211	0.0488	>0.9999			

Table S5: Differentially abundant proteins (LC-MS/MS, Day 7). Sorted by descending absolute log_2 fold change. Proteins are represented by the leading protein in a protein group. Protein description are based on UniProt anotations (March 16, 2016). Protein to gene mappings and gene names were obtained from Ensembl (Ensembl Release 84; March 2016).

Protein ID	Gene Name	Protein Description	50% Protein Cluster ID (Gene Name)	Log_2 Fold Change (Day 14 vs. Day 0)	t-Statistic	P-value	FDR Adjusted P-value	Protein Group Protein IDs	Protein Group Gene Ids	Protein Group Gene Names
P31939	ATIC	Bifunctional purine biosynthesis protein PURH	P31939 (ATIC)	1.152	3.247	0.0098	0.4756			
P22314	UBA1	Ubiquitin-like modifier-activating enzyme 1	P22314 (UBA1)	1.141	2.159	0.0273	0.5222			
P07900	HSP90AA1	Heat shock protein HSP 90-alpha	P07900 (HSP90AA1)	1.126	2.386	0.0156	0.5188	P07900<br>Q14568<br>Q58FG0	ENSG00000080824	HSP90AA1<br>HSP90AA2P<br>HSP90AA5P
P60174	TPI1	Triosephosphate isomerase	P60174 (TPI1)	1.117	2.389	0.0078	0.4756			

Protein ID	Gene Name	Protein Description	50% Protein Cluster ID (Gene Name)	Log_2 Fold Change (Day 14 vs. Day 0)	t-Statistic	P-value	FDR Adjusted P-value	Protein Group Protein IDs	Protein Group Gene Ids	Protein Group Gene Names
O00567	NOP56	Nucleolar protein 56	O00567 (NOP56)	1.117	3.390	0.0078	0.4756			
O60506	SYNCRIP	Heterogeneous nuclear ribonucleoprotein Q	O43390 (HNRNPR)	1.095	2.597	0.0234	0.5188			
Q9Y3Z3	SAMHD1	Deoxynucleoside triphosphate triphosphohydrolase SAMHD1	Q9Y3Z3 (SAMHD1)	1.083	2.397	0.0156	0.5188			
P62277	RPS13	40S ribosomal protein S13	P62277 (RPS13)	1.030	3.824	0.0078	0.4756			
P06748	NPM1	Nucleophosmin	P06748 (NPM1)	1.014	1.482	0.0176	0.5188			
P49368	CCT3	T-complex protein 1 subunit gamma	P49368 (CCT3)	1.013	2.123	0.0195	0.5188			
Q15365	PCBP1	Poly(rC)-binding protein 1	P57721 (PCBP3)	1.013	2.213	0.0371	0.5648			
P26447	S100A4	Protein S100-A4	P26447 (S100A4)	0.934	2.932	0.0059	0.4756			
Q9P258	RCC2	Protein RCC2	Q9P258 (RCC2)	0.916	2.417	0.0293	0.5248			
Q16531	DDB1	DNA damage-binding protein 1	Q16531 (DDB1)	0.893	2.502	0.0195	0.5188			
P60842	EIF4A1	Eukaryotic initiation factor 4A-I	P38919 (EIF4A3)	0.878	2.403	0.0430	0.5895			
P25789	PSMA4	Proteasome subunit alpha type-4	P25789 (PSMA4)	0.859	3.211	0.0098	0.4756			
Q9UQE7	SMC3	Structural maintenance of chromosomes protein 3	Q9UQE7 (SMC3)	0.851	2.141	0.0312	0.5248			
P51858	HDGF	Hepatoma-derived growth factor	Q5TGJ6 (HDGFL1)	0.849	3.363	0.0098	0.4756			
P29401	TKT	Transketolase	Q9H0I9 (TKTL2)	0.835	2.211	0.0488	0.5959			
Q9UJU6	DBNL	Drebrin-like protein	Q9UJU6 (DBNL)	0.828	2.196	0.0488	0.5959			
P26583	HMGB2	High mobility group protein B2	P23497 (SP100)	0.813	2.542	0.0137	0.5122			
P09429	HMGB1	High mobility group protein B1	P23497 (SP100)	0.807	2.232	0.0039	0.4756	P09429<br>B2RPK0	ENSG00000189403	HMGB1<br>HMGB1P1
Q15029	EFTUD2	116 kDa U5 small nuclear ribonucleoprotein component	Q15029 (EFTUD2)	0.799	2.073	0.0195	0.5188			
P63220	RPS21	40S ribosomal protein S21	P63220 (RPS21)	0.767	2.371	0.0469	0.5959			
P43686	PSMC4	26S protease regulatory subunit 6B	P43686 (PSMC4)	0.757	2.280	0.0371	0.5648			
P43405	SYK	Tyrosine-protein kinase SYK	P43405 (SYK)	0.747	2.432	0.0391	0.5707	P43405<br>Q8NFD2	ENSG00000165025<br>ENSG00000170209	SYK<br>ANKK1
P23193	TCEA1	Transcription elongation factor A protein 1	P23193 (TCEA1)	0.732	3.118	0.0078	0.4756	P23193<br>Q15560	ENSG00000171703<br>ENSG00000187735	TCEA1<br>TCEA2
P39748	FEN1	Flap endonuclease 1 ECO:0000255|HAMAP-Rule:MF03140	P39748 (FEN1)	0.729	3.782	0.0098	0.4756			
O00743	PPP6C	Serine/threonine-protein phosphatase 6 catalytic subunit	P62714 (PPP2CB)	0.712	2.936	0.0215	0.5188			
P62888	RPL30	60S ribosomal protein L30	P62888 (RPL30)	0.712	3.206	0.0098	0.4756			
P26599	PTBP1	Polypyrimidine tract-binding protein 1	P26599 (PTBP1)	0.709	2.498	0.0039	0.4756			
Q9UKM9	RALY	RNA-binding protein Raly	Q9UKM9 (RALY)	0.697	2.701	0.0234	0.5188			
P27635	RPL10	60S ribosomal protein L10	P27635 (RPL10)	0.691	2.288	0.0254	0.5222	P27635<br>Q96L21	ENSG00000165496	RPL10<br>RPL10L
Q9Y3I0	RTCB	tRNA-splicing ligase RtcB homolog ECO:0000255|HAMAP-Rule:MF03144	Q9Y3I0 (RTCB)	0.688	2.303	0.0254	0.5222			
P12956	XRCC6	X-ray repair cross-complementing protein 6	P12956 (XRCC6)	0.684	2.104	0.0234	0.5188			
P36578	RPL4	60S ribosomal protein L4	P36578 (RPL4)	0.680	2.973	0.0215	0.5188			
P28066	PSMA5	Proteasome subunit alpha type-5	P28066 (PSMA5)	0.674	2.382	0.0195	0.5188			
P37837	TALDO1	Transaldolase	P37837 (TALDO1)	0.672	2.200	0.0117	0.5122			
P29692	EEF1D	Elongation factor 1-delta	P29692 (EEF1D)	0.658	2.581	0.0215	0.5188			
P02787	TF	Serotransferrin	P02788 (LTF)	-0.652	-1.995	0.0312	0.5248			
P46777	RPL5	60S ribosomal protein L5	P46777 (RPL5)	0.641	2.867	0.0156	0.5188			
P18124	RPL7	60S ribosomal protein L7	P18124 (RPL7)	0.637	2.126	0.0469	0.5959			
Q9Y3Y2	CHTOP	Chromatin target of PRMT1 protein	Q9Y3Y2 (CHTOP)	0.622	2.360	0.0488	0.5959			
Q12906	ILF3	Interleukin enhancer-binding factor 3	Q12906 (ILF3)	0.614	2.369	0.0098	0.4756	Q12906<br>Q96SI9	ENSG00000129351<br>ENSG00000165209	ILF3<br>STRBP
P35268	RPL22	60S ribosomal protein L22	P35268 (RPL22)	0.610	3.905	0.0078	0.4756			
Q9Y2X3	NOP58	Nucleolar protein 58	Q9Y2X3 (NOP58)	0.609	2.858	0.0312	0.5248			
P17612	PRKACA	cAMP-dependent protein kinase catalytic subunit alpha	P22694 (PRKACB)	0.590	3.167	0.0098	0.4756			

Protein ID	Gene Name	Protein Description	50% Protein Cluster ID (Gene Name)	Log_2 Fold Change (Day 14 vs. Day 0)	t-Statistic	P-value	FDR Adjusted P-value	Protein Group Protein IDs	Protein Group Gene Ids	Protein Group Gene Names
P62273	RPS29	40S ribosomal protein S29	P62273 (RPS29)	0.588	2.182	0.0488	0.5959			
Q99729	HNRNPAB	Heterogeneous nuclear ribonucleoprotein A/B	O14979 (HNRNPDL)	0.585	2.128	0.0312	0.5248			
Q14974	KPNB1	Importin subunit beta-1	Q14974 (KPNB1)	0.582	2.718	0.0137	0.5122			
P25788	PSMA3	Proteasome subunit alpha type-3	P25788 (PSMA3)	0.581	2.524	0.0410	0.5707			
P39023	RPL3	60S ribosomal protein L3	Q92901 (RPL3L)	0.580	2.390	0.0449	0.5959	P39023<br>Q92901	ENSG00000100316<br>ENSG00000140986	RPL3<br>RPL3L
P13639	EEF2	Elongation factor 2	P13639 (EEF2)	0.568	2.394	0.0352	0.5648			
O15143	ARPC1B	Actin-related protein 2/3 complex subunit 1B	O15143 (ARPC1B)	0.565	1.742	0.0273	0.5222			
P52907	CAPZA1	F-actin-capping protein subunit alpha-1	P52907 (CAPZA1)	0.555	1.757	0.0254	0.5222			
P36543	ATP6V1E1	V-type proton ATPase subunit E 1	P36543 (ATP6V1E1)	0.547	3.268	0.0137	0.5122			
O00193	SMAP	Small acidic protein	O00193 (SMAP)	0.538	3.368	0.0020	0.4756			
P18621	RPL17	60S ribosomal protein L17	P18621 (RPL17)	0.535	3.369	0.0020	0.4756			
P31949	S100A11	Protein S100-A11	P31949 (S100A11)	0.528	3.714	0.0137	0.5122			
P24534	EEF1B2	Elongation factor 1-beta	P24534 (EEF1B2)	0.519	3.697	0.0098	0.4756			
P62269	RPS18	40S ribosomal protein S18	P62269 (RPS18)	0.514	4.414	0.0020	0.4756			
P07305	H1F0	Histone H1.0	P07305 (H1F0)	0.500	3.323	0.0117	0.5122			
Q6P4A8	PLBD1	Phospholipase B-like 1	Q6P4A8 (PLBD1)	0.499	2.292	0.0371	0.5648			
Q9HC35	EML4	Echinoderm microtubule-associated protein-like 4	Q9HC35 (EML4)	0.484	2.677	0.0312	0.5248			
Q96PK6	RBM14	RNA-binding protein 14	Q96PK6 (RBM14)	0.479	2.620	0.0234	0.5188			
Q08945	SSRP1	FACT complex subunit SSRP1	Q08945 (SSRP1)	0.451	2.434	0.0410	0.5707			
P82979	SARNP	SAP domain-containing ribonucleoprotein	P82979 (SARNP)	0.446	2.368	0.0332	0.5481			
Q15717	ELAVL1	ELAV-like protein 1	Q15717 (ELAVL1)	0.437	2.360	0.0391	0.5707			
P42677	RPS27	40S ribosomal protein S27	Q71UM5 (RPS27L)	0.432	2.211	0.0371	0.5648			
P46781	RPS9	40S ribosomal protein S9	P46781 (RPS9)	0.431	2.670	0.0312	0.5248			
Q02878	RPL6	60S ribosomal protein L6	Q02878 (RPL6)	0.408	2.789	0.0215	0.5188			
Q9Y4Y9	LSM5	U6 snRNA-associated Sm-like protein LSm5	Q9Y4Y9 (LSM5)	0.400	2.349	0.0273	0.5222			
O14974	PPP1R12A	Protein phosphatase 1 regulatory subunit 12A	O14974 (PPP1R12A)	0.396	2.430	0.0410	0.5707			
Q13347	EIF3I	Eukaryotic translation initiation factor 3 subunit I ECO:0000255|HAMAP-Rule:MF03008	Q13347 (EIF3I)	0.389	3.043	0.0195	0.5188			
P80217	IFI35	Interferon-induced 35 kDa protein	P80217 (IFI35)	0.362	2.488	0.0410	0.5707			
Q8WUW1	BRK1	Protein BRICK1	Q8WUW1 (BRK1)	0.346	8.413	0.0020	0.4756			
O60493	SNX3	Sorting nexin-3	Q9UMY4 (SNX12)	0.318	2.936	0.0195	0.5188			

Table S6: Differentially abundant proteins (LC-MS/MS, Day 14). Sorted by descending absolute log_2 fold change. Proteins are represented by the leading protein in a protein group. Protein description are based on UniProt anotations (March 16, 2016). Protein to gene mappings and gene names were obtained from Ensembl (Ensembl Release 84; March 2016).

Gel Spot ID	Protein ID	Gene Name	Protein Description	50% Protein Cluster ID (Gene Name)	Log_2 Fold Change (Day 7 vs. Day 0)	t-Statistic	P-value	FDR Adjusted P-value	Protein Group Protein IDs	Protein Group Gene Ids	Protein Group Gene Names
S0544	P13796	LCP1	Plastin-2	P13797 (PLS3)	1.311	3.048	0.0117	0.5256	P13796	ENSG00000136167	LCP1
S0729	P60709	ACTB	Actin, cytoplasmic 1	A5A3E0 (POTEF)	1.131	2.305	0.0488	0.5532	P60709<br>P63261	ENSG00000075624<br>ENSG00000184009	ACTB<br>ACTG1

Gel Spot ID	Protein ID	Gene Name	Protein Description	50% Protein Cluster ID (Gene Name)	Log_2 Fold Change (Day 7 vs. Day 0)	t-Statistic	P-value	FDR Adjusted P-value	Protein Group Protein IDs	Protein Group Gene Ids	Protein Group Gene Names
S0638	P68363	TUBA1B	Tubulin alpha-1B chain	Q71U36 (TUBA1A)	1.118	2.715	0.0156	0.5256	P68363<br>Q9BQE3<br>Q71U36<br>Q13748<br>Q6PEY2<br>Q9NY65	ENSG00000152086<br>ENSG00000123416<br>ENSG00000198033<br>ENSG00000075886<br>ENSG00000167552<br>ENSG00000183785<br>ENSG00000167553	TUBA1B<br>TUBA1C<br>TUBA1A<br>TUBA3D<br>TUBA3E<br>TUBA8
S0452	P07900	HSP90AA1	Heat shock protein HSP 90-alpha	P07900 (HSP90AA1)	0.997	3.099	0.0176	0.5256	P07900<br>P08238	ENSG00000096384<br>ENSG00000080824	HSP90AA1<br>HSP90AB1
S0568					0.972	2.590	0.0371	0.5391			
S0651	P07437	TUBB	Tubulin beta chain	Q9H4B7 (TUBB1)	0.937	2.624	0.0254	0.5256	P07437<br>P68371<br>P04350<br>Q13885<br>Q9BVA1<br>Q13509	ENSG00000137285<br>ENSG00000229684<br>ENSG00000104833<br>ENSG00000232575<br>ENSG00000258947<br>ENSG00000232421<br>ENSG00000196230<br>ENSG00000224156<br>ENSG00000227739<br>ENSG00000235067<br>ENSG00000183311<br>ENSG00000137267<br>ENSG00000188229	TUBB<br>TUBB4B<br>TUBB4A<br>TUBB2A<br>TUBB2B<br>TUBB3
S0222					-0.837	-3.097	0.0059	0.5256			
S0804					-0.772	-2.141	0.0391	0.5391			
S0771	Q9H6N6	MYH16	Putative uncharacterized protein MYH16 ECO:0000305	Q9H6N6 (MYH16)	0.727	2.623	0.0332	0.5391	Q9H6N6<br>Q9UBV4	ENSG00000002745	MYH16<br>WNT16
S0109					-0.716	-2.594	0.0176	0.5256			
S0515	P11142	HSPA8	Heat shock cognate 71 kDa protein	P11142 (HSPA8)	0.691	2.405	0.0430	0.5391	P11142	ENSG00000109971	HSPA8
S0413					-0.679	-2.661	0.0117	0.5256			
S0493					0.666	2.740	0.0234	0.5256			
S1007	P04179	SOD2	Superoxide dismutase [Mn], mitochondrial	P04179 (SOD2)	0.611	4.239	0.0078	0.5256	P04179		SOD2
S0678	P08670	VIM	Vimentin	P41219 (PRPH)	0.593	2.276	0.0430	0.5391	P08670	ENSG00000026025	VIM
S0861	P08758	ANXA5	Annexin A5	P08133 (ANXA6)	0.587	2.959	0.0195	0.5256	P08758	ENSG00000164111	ANXA5
S0917	P60174	TPI1	Triosephosphate isomerase	P60174 (TPI1)	0.580	2.449	0.0391	0.5391	P60174	ENSG00000111669	TPI1
S0538					0.577	3.721	0.0059	0.5256			
S0195					-0.529	-2.302	0.0449	0.5470			
S0427					0.528	2.937	0.0176	0.5256			
S0669	P07355	ANXA2	Annexin A2	P04083 (ANXA1)	0.527	2.415	0.0332	0.5391	P07355<br>P06733<br>P49411	ENSG00000182718<br>ENSG00000074800	ANXA2<br>ENO1<br>TUFM
S0085					-0.516	-2.945	0.0215	0.5256			
S0836					0.509	2.285	0.0410	0.5391			
S0612					-0.494	-2.475	0.0430	0.5391			
S0535	P0DMV8	HSPA1A	Heat shock 70 kDa protein 1A ECO:0000312|HGNC:HGNC:5232	P11142 (HSPA8)	0.471	2.466	0.0293	0.5391	P0DMV8	ENSG00000237724<br>ENSG00000235941<br>ENSG00000215328<br>ENSG00000234475<br>ENSG00000204389	HSPA1A
S0958	P25787	PSMA2	Proteasome subunit alpha type-2	P25787 (PSMA2)	0.457	3.093	0.0078	0.5256	P25787<br>O95376	ENSG00000177479<br>ENSG00000106588	PSMA2<br>ARIH2

Gel Spot ID	Protein ID	Gene Name	Protein Description	50% Protein Cluster ID (Gene Name)	Log_2 Fold Change (Day 7 vs. Day 0)	t-Statistic	P-value	FDR Adjusted P-value	Protein Group Protein IDs	Protein Group Gene Ids	Protein Group Gene Names
S0495					0.452	2.899	0.0156	0.5256			
S0524	Q9H2C0	GAN	Gigaxonin	Q9H2C0 (GAN)	0.432	2.512	0.0410	0.5391	Q9H2C0	ENSG00000261609	GAN
S0751					0.418	2.592	0.0098	0.5256			
S0805					0.418	2.401	0.0352	0.5391			
S0650	P13489	RNH1	Ribonuclease inhibitor	P13489 (RNH1)	0.389	2.325	0.0469	0.5532	P13489	ENSG00000023191<br>ENSG00000276230	RNH1
S0909					-0.340	-2.701	0.0156	0.5256			
S0588	P13645	KRT10	Keratin, type I cytoskeletal 10	P35527 (KRT9)	0.330	2.916	0.0215	0.5256	P13645<br>P33176	ENSG00000170759<br>ENSG00000186395	KRT10<br>KIF5B
S0400	P21333	FL	Filamin-A	Q14315 (FLNC)	-0.270	-2.709	0.0254	0.5256	P21333<br>Q12774<br>P21333	ENSG00000196924<br>ENSG00000050327	FL<br>ARHGEF5

Table S7: Differentially abundant protein gel spots (2D-DIGE/MS, Day 7). Sorted by descending absolute log_2 fold change. Protein annotations are based on UniProt anotations (March 16, 2016)

Gel Spot ID	Protein ID	Gene Name	Protein Description	50% Protein Cluster ID (Gene Name)	Log_2 Fold Change (Day 14 vs. Day 0)	t-Statistic	P-value	FDR Adjusted P-value	Protein Group Protein IDs	Protein Group Gene Ids	Protein Group Gene Names
S0544	P13796	LCP1	Plastin-2	P13797 (PLS3)	1.197	2.985	0.0117	0.1733	P13796	ENSG00000136167	LCP1
S0729	P60709	ACTB	Actin, cytoplasmic 1	A5A3E0 (POTEF)	1.184	2.863	0.0059	0.1348	P60709<br>P63261	ENSG00000075624<br>ENSG00000184009	ACTB<br>ACTG1
S0638	P68363	TUBA1B	Tubulin alpha-1B chain	Q71U36 (TUBA1A)	1.033	2.658	0.0195	0.1972	P68363<br>Q9BQE3<br>Q71U36<br>Q13748<br>Q6PEY2<br>Q9NY65	ENSG00000152086<br>ENSG00000123416<br>ENSG00000198033<br>ENSG00000075886<br>ENSG00000167552<br>ENSG00000183785<br>ENSG00000167553	TUBA1B<br>TUBA1C<br>TUBA1A<br>TUBA3D<br>TUBA3E<br>TUBA8
S0452	P07900	HSP90AA1	Heat shock protein HSP 90-alpha	P07900 (HSP90AA1)	1.026	3.789	0.0078	0.1348	P07900<br>P08238	ENSG00000096384<br>ENSG00000080824	HSP90AA1<br>HSP90AB1
S0728	P60709	ACTB	Actin, cytoplasmic 1	A5A3E0 (POTEF)	1.003	3.497	0.0078	0.1348	P60709<br>P63261	ENSG00000075624<br>ENSG00000184009	ACTB<br>ACTG1
S0651	P07437	TUBB	Tubulin beta chain	Q9H4B7 (TUBB1)	0.893	3.434	0.0078	0.1348	P07437<br>P68371<br>P04350<br>Q13885<br>Q9BVA1<br>Q13509	ENSG00000137285<br>ENSG00000229684<br>ENSG00000104833<br>ENSG00000232575<br>ENSG00000258947<br>ENSG00000232421<br>ENSG00000196230<br>ENSG00000224156<br>ENSG00000227739<br>ENSG00000235067<br>ENSG00000183311<br>ENSG00000137267<br>ENSG00000188229	TUBB<br>TUBB4B<br>TUBB4A<br>TUBB2A<br>TUBB2B<br>TUBB3
S0911	P63104	YWHAZ	14-3-3 protein zeta/delta	P62258 (YWHAE)	0.886	3.140	0.0059	0.1348	P63104	ENSG00000164924	YWHAZ
S0887	P67936	TPM4	Tropomyosin alpha-4 chain	P06753 (TPM3)	0.850	2.904	0.0078	0.1348	P67936	ENSG00000167460	TPM4

Gel Spot ID	Protein ID	Gene Name	Protein Description	50% Protein Cluster ID (Gene Name)	Log_2 Fold Change (Day 14 vs. Day 0)	t-Statistic	P-value	FDR Ad-justed P-value	Protein Group Protein IDs	Protein Group Gene Ids	Protein Group Gene Names
S0109					-0.839	-2.516	0.0078	0.1348			
S0662	P06733	ENO1	Alpha-enolase	P13929 (ENO3)	0.770	1.984	0.0078	0.1348	P06733	ENSG00000074800	ENO1
S0746					-0.744	-3.868	0.0059	0.1348			
S0828	P47756	CAPZB	F-actin-capping protein subunit beta	P47756 (CAPZB)	0.743	2.875	0.0156	0.1797	P47756<br>Q06323<br>Q6ZU80	ENSG00000077549<br>ENSG00000092010<br>ENSG00000100629	CAPZB<br>PSME1<br>CEP128
S0222					-0.737	-2.147	0.0312	0.2270			
S0573					0.723	3.678	0.0059	0.1348			
S0413					-0.722	-2.546	0.0078	0.1348			
S0928					-0.722	-2.681	0.0273	0.2166			
S0412	P14625	HSP90B1	Endoplasmin	P14625 (HSP90B1)	0.710	3.153	0.0078	0.1348	P14625	ENSG00000166598	HSP90B1
S0515	P11142	HSPA8	Heat shock cognate 71 kDa protein	P11142 (HSPA8)	0.704	3.067	0.0156	0.1797	P11142	ENSG00000109971	HSPA8
S0986					-0.699	-2.372	0.0332	0.2370			
S0069					-0.692	-3.828	0.0078	0.1348			
S0534					0.689	3.315	0.0156	0.1797			
S0168					-0.684	-3.289	0.0137	0.1797			
S0448					-0.672	-2.181	0.0371	0.2439			
S0678	P08670	VIM	Vimentin	P41219 (PRPH)	0.665	3.734	0.0137	0.1797	P08670	ENSG00000026025	VIM
S0077					-0.643	-2.834	0.0254	0.2166			
S0623	P27797	CALR	Calreticulin	P27797 (CALR)	0.640	3.791	0.0059	0.1348	P27797	ENSG00000179218	CALR
S0596					0.622	3.544	0.0078	0.1348			
S0804					-0.616	-2.350	0.0449	0.2619			
S0909					-0.610	-3.482	0.0039	0.1348			
S0513	P11021	HSPA5	78 kDa glucose-regulated protein	P38646 (HSPA9)	0.606	3.355	0.0137	0.1797	P11021<br>P11021	ENSG00000044574	HSPA5
S0119					-0.605	-2.751	0.0293	0.2166			
S0103	Q9Y490	TLN1	Talin-1	Q9Y4G6 (TLN2)	0.597	2.254	0.0371	0.2439	Q9Y490<br>Q9H7E2	ENSG00000083544<br>ENSG00000137076	TLN1<br>TDRD3
S0942					-0.596	-2.150	0.0371	0.2439			
S0642					0.594	3.100	0.0117	0.1733			
S0602	P07237	P4HB	Protein disulfide-isomerase	P07237 (P4HB)	0.590	3.687	0.0020	0.1348	P07237	ENSG00000185624	P4HB
S0934	P52566	ARHGDIB	Rho GDP-dissociation inhibitor 2	Q99819 (ARHGDIG)	0.589	2.477	0.0078	0.1348	P52566	ENSG00000111348	ARHGDIB
S0287					-0.587	-2.067	0.0449	0.2619			
S0790	P52907	CAPZA1	F-actin-capping protein subunit alpha-1	P52907 (CAPZA1)	0.584	3.776	0.0039	0.1348	P52907<br>Q8N807	ENSG00000169340<br>ENSG00000116489	CAPZA1<br>PDILT
S0418	P55072	VCP	Transitional endoplasmic reticulum ATPase	P55072 (VCP)	0.583	2.844	0.0273	0.2166	P55072	ENSG00000165280	VCP
S0522	P02768	ALB	Serum albumin	P02768 (ALB)	0.575	2.738	0.0234	0.2156	P02768<br>P02768	ENSG00000163631	ALB
S0321					-0.553	-3.615	0.0020	0.1348			
S0535	P0DMV8	HSPA1A	Heat shock 70 kDa protein 1A ECO:0000312|HGNC:HGNC:5232	P11142 (HSPA8)	0.540	3.274	0.0156	0.1797	P0DMV8	ENSG00000237724<br>ENSG00000235941<br>ENSG00000215328<br>ENSG00000234475<br>ENSG00000204389	HSPA1A
S0158					-0.538	-2.452	0.0449	0.2619			
S0933	P04264	KRT1	Keratin, type II cytoskeletal 1	P04264 (KRT1)	-0.537	-2.871	0.0254	0.2166	P04264<br>P35527	ENSG00000167768<br>ENSG00000171403	KRT1<br>KRT9
S0861	P08758	ANXA5	Annexin A5	P08133 (ANXA6)	0.529	2.747	0.0254	0.2166	P08758	ENSG00000164111	ANXA5
S0761					0.525	2.879	0.0039	0.1348			
S0493					0.513	2.743	0.0293	0.2166			
S0778					-0.505	-2.512	0.0234	0.2156			

Gel Spot ID	Protein ID	Gene Name	Protein Description	50% Protein Cluster ID (Gene Name)	Log_2 Fold Change (Day 14 vs. Day 0)	t-Statistic	P-value	FDR Adjusted P-value	Protein Group Protein IDs	Protein Group Gene Ids	Protein Group Gene Names
S0516					0.500	2.484	0.0176	0.1819			
S0771	Q9H6N6	MYH16	Putative uncharacterized protein MYH16 ECO:0000305	Q9H6N6 (MYH16)	0.498	2.543	0.0293	0.2166	Q9H6N6<br>Q9UBV4	ENSG00000002745	MYH16<br>WNT16
S0669	P07355	ANXA2	Annexin A2	P04083 (ANXA1)	0.493	3.090	0.0117	0.1733	P07355<br>P06733<br>P49411	ENSG00000182718<br>ENSG00000074800	ANXA2<br>ENO1<br>TUFM
S0896					-0.485	-3.062	0.0156	0.1797			
S0164					-0.481	-2.365	0.0469	0.2695			
S0809					0.474	2.585	0.0273	0.2166			
S0467					0.470	2.626	0.0117	0.1733			
S0685					-0.465	-2.180	0.0449	0.2619			
S0650	P13489	RNH1	Ribonuclease inhibitor	P13489 (RNH1)	0.461	3.235	0.0059	0.1348	P13489	ENSG00000023191<br>ENSG00000276230	RNH1
S0892					-0.457	-2.513	0.0352	0.2426			
S0699					0.455	2.360	0.0293	0.2166			
S0730					0.438	3.542	0.0078	0.1348			
S0801					-0.417	-2.828	0.0215	0.2118			
S0841					-0.413	-2.485	0.0391	0.2450			
S0118					-0.407	-2.222	0.0488	0.2732			
S0427					0.404	3.022	0.0234	0.2156			
S0379					0.402	3.096	0.0176	0.1819			
S0586					0.401	2.488	0.0410	0.2534			
S0902					-0.399	-2.683	0.0352	0.2426			
S0712					-0.389	-2.511	0.0273	0.2166			
S0983					-0.386	-3.212	0.0176	0.1819			
S0588	P13645	KRT10	Keratin, type I cytoskeletal 10	P35527 (KRT9)	0.347	3.453	0.0078	0.1348	P13645<br>P33176	ENSG00000170759<br>ENSG00000186395	KRT10<br>KIF5B
S0924					-0.316	-2.225	0.0488	0.2732			
S0598	P02675	FGB	Fibrinogen beta chain	P02675 (FGB)	0.314	3.224	0.0176	0.1819	P02675	ENSG00000171564	FGB
S0722					-0.283	-2.223	0.0391	0.2450			
S0527					0.279	2.331	0.0391	0.2450			

Table S8: Differentially abundant protein gel spots (2D-DIGE/MS, Day 14). Sorted by descending absolute log_2 fold change. Protein annotations are based on UniProt anotations (March 16, 2016)

Category Name	Category Genes #	Sig. Genes #(%) [Protein Fam. #]	Genes Up #(%)	Genes Down #(%)	Jaccard similarity coefficient	P	FDR
Ribosome	127	5 (3.9) [5]	5 (3.9)	0 (0)	0.0362	<0.0001	0.003

Table S9: Enriched KEGG Pathways (LC-MS/MS, Day 7). Results are sorted by false discovery rate and Jaccard similarity coefficient.

Category Name	Category Genes #	Sig. Genes #(%) [Protein Fam. #]	Genes Up #(%)	Genes Down #(%)	Jaccard similarity coefficient	P	FDR
ACTIVATION OF THE MRNA UPON BINDING OF THE CAP BINDING COMPLEX AND EIFS AND SUBSEQUENT BINDING TO 43S	54	5 (9.3) [5]	5 (9.3)	0 (0)	0.0725	<0.0001	0.0004
3 UTR MEDIATED TRANSLATIONAL REGULATION	102	6 (5.9) [6]	6 (5.9)	0 (0)	0.0517	<0.0001	0.0004
METABOLISM OF MRNA	204	7 (3.4) [7]	7 (3.4)	0 (0)	0.0323	<0.0001	0.001
PEPTIDE CHAIN ELONGATION	82	5 (6.1) [5]	5 (6.1)	0 (0)	0.0515	<0.0001	0.0011
TRANSLATION	142	6 (4.2) [6]	6 (4.2)	0 (0)	0.0385	<0.0001	0.0011
FORMATION OF THE TERNARY COMPLEX AND SUBSEQUENTLY THE 43S COMPLEX	46	4 (8.7) [4]	4 (8.7)	0 (0)	0.0645	<0.0001	0.0016
INFLUENZA VIRAL RNA TRANSCRIPTION AND REPLICATION	97	5 (5.2) [5]	5 (5.2)	0 (0)	0.0446	<0.0001	0.0016
METABOLISM OF RNA	248	7 (2.8) [7]	7 (2.8)	0 (0)	0.0268	<0.0001	0.0016
NONSENSE MEDIATED DECAY ENHANCED BY THE EXON JUNCTION COMPLEX	103	5 (4.9) [5]	5 (4.9)	0 (0)	0.0424	<0.0001	0.0019
SRP DEPENDENT COTRANSLATIONAL PROTEIN TARGETING TO MEMBRANE	105	5 (4.8) [5]	5 (4.8)	0 (0)	0.0417	<0.0001	0.0019
INFLUENZA LIFE CYCLE	131	5 (3.8) [5]	5 (3.8)	0 (0)	0.0342	<0.0001	0.0049
METABOLISM OF PROTEINS	408	7 (1.7) [7]	7 (1.7)	0 (0)	0.0166	0.0004	0.021

Table S10: Enriched MSigDB Reactome Pathways (LC-MS/MS, Day 7). Results are sorted by false discovery rate and Jaccard similarity coefficient.

Category Name	Category Genes #	Sig. Genes #(%) [Protein Fam. #]	Genes Up #(%)	Genes Down #(%)	Jaccard similarity coefficient	P	FDR
GSE41978 KLRG1 HIGH VS LOW EFFECTOR CD8 TCELL DN	186	5 (2.7) [5]	5 (2.7)	0 (0)	0.0244	<0.0001	0.0101
GSE41978 ID2 KO VS ID2 KO AND BIM KO KLRG1 LOW EFFECTOR CD8 TCELL DN	190	5 (2.6) [5]	5 (2.6)	0 (0)	0.0239	<0.0001	0.0101
GSE2405 0H VS 9H A PHAGOCYTOPHILUM STIM NEUTROPHIL DN	192	5 (2.6) [5]	5 (2.6)	0 (0)	0.0237	<0.0001	0.0101
GSE22886 NAIVE TCELL VS DC UP	183	4 (2.2) [4]	4 (2.2)	0 (0)	0.0197	0.0001	0.0999
GSE22886 NAIVE BCELL VS NEUTROPHIL UP	184	4 (2.2) [4]	4 (2.2)	0 (0)	0.0196	0.0001	0.0999
GSE3982 EOSINOPHIL VS MAST CELL DN	186	4 (2.2) [4]	4 (2.2)	0 (0)	0.0194	0.0001	0.0999
GSE2405 0H VS 24H A PHAGOCYTOPHILUM STIM NEUTROPHIL UP	193	4 (2.1) [4]	4 (2.1)	0 (0)	0.0188	0.0001	0.0999

Table S11: Enriched MSigDB Immunologic Signature Sets (LC-MS/MS, Day 7). Results are sorted by false discovery rate and Jaccard similarity coefficient.

Category Name	Category Genes #	Sig. Genes #(%) [Protein Fam. #]	Genes Up #(%)	Genes Down #(%)	Jaccard similarity coefficient	P	FDR
Ribosome	127	13 (10.2) [14]	13 (10.2)	0 (0)	0.0798	<0.0001	<0.0001
Proteasome	44	4 (9.1) [4]	4 (9.1)	0 (0)	0.0449	0.0003	0.0473

Table S12: Enriched KEGG Pathways (LC-MS/MS, Day 14). Results are sorted by false discovery rate and Jaccard similarity coefficient.

Category Name	Category Genes #	Sig. Genes #(%) [Protein Fam. #]	Genes Up #(%)	Genes Down #(%)	Jaccard similarity coefficient	P	FDR
TRANSLATION	142	18 (12.7) [18]	18 (12.7)	0 (0)	0.1000	<0.0001	<0.0001
METABOLISM OF MRNA	204	20 (9.8) [20]	20 (9.8)	0 (0)	0.0833	<0.0001	<0.0001
PEPTIDE CHAIN ELONGATION	82	14 (17.1) [14]	14 (17.1)	0 (0)	0.1129	<0.0001	<0.0001
3 UTR MEDIATED TRANSLATIONAL REGULATION	102	15 (14.7) [15]	15 (14.7)	0 (0)	0.1049	<0.0001	<0.0001
METABOLISM OF RNA	248	20 (8.1) [20]	20 (8.1)	0 (0)	0.0704	<0.0001	<0.0001
INFLUENZA VIRAL RNA TRANSCRIPTION AND REPLICATION	97	14 (14.4) [14]	14 (14.4)	0 (0)	0.1007	<0.0001	<0.0001
INFLUENZA LIFE CYCLE	131	15 (11.5) [15]	15 (11.5)	0 (0)	0.0872	<0.0001	<0.0001
NONSENSE MEDIATED DECAY ENHANCED BY THE EXON JUNCTION COMPLEX	103	13 (12.6) [13]	13 (12.6)	0 (0)	0.0890	<0.0001	<0.0001
SRP DEPENDENT COTRANSLATIONAL PROTEIN TARGETING TO MEMBRANE	105	13 (12.4) [13]	13 (12.4)	0 (0)	0.0878	<0.0001	<0.0001
METABOLISM OF PROTEINS	408	20 (4.9) [20]	20 (4.9)	0 (0)	0.0450	<0.0001	<0.0001
ACTIVATION OF THE MRNA UPON BINDING OF THE CAP BINDING COMPLEX AND EIFS AND SUB-SEQUENT BINDING TO 43S	54	7 (13) [7]	7 (13)	0 (0)	0.0680	<0.0001	<0.0001
FORMATION OF THE TERNARY COMPLEX AND SUBSEQUENTLY THE 43S COMPLEX	46	6 (13) [6]	6 (13)	0 (0)	0.0625	<0.0001	0.0003
APOPTOSIS INDUCED DNA FRAGMENTATION	13	4 (30.8) [3]	4 (30.8)	0 (0)	0.0615	<0.0001	0.0003
APOPTOSIS	142	9 (6.3) [8]	9 (6.3)	0 (0)	0.0476	<0.0001	0.0005
HIV INFECTION	188	10 (5.3) [10]	10 (5.3)	0 (0)	0.0427	<0.0001	0.0007
APOPTOTIC EXECUTION PHASE	51	5 (9.8) [4]	5 (9.8)	0 (0)	0.0490	0.0001	0.0061
DNA REPLICATION	185	8 (4.3) [8]	8 (4.3)	0 (0)	0.0343	0.0005	0.019
CELL CYCLE MITOTIC	299	10 (3.3) [10]	10 (3.3)	0 (0)	0.0290	0.0007	0.0263
CDK MEDIATED PHOSPHORYLATION AND REMOVAL OF CDC6	46	4 (8.7) [4]	4 (8.7)	0 (0)	0.0408	0.0011	0.0335
CROSS PRESENTATION OF SOLUBLE EXOGENOUS ANTIGENS ENDOSOMES	46	4 (8.7) [4]	4 (8.7)	0 (0)	0.0408	0.0011	0.0335
AUTODEGRADATION OF THE E3 UBIQUITIN LIGASE COP1	47	4 (8.5) [4]	4 (8.5)	0 (0)	0.0404	0.0012	0.0335
REGULATION OF ORNITHINE DECARBOXYLASE ODC	47	4 (8.5) [4]	4 (8.5)	0 (0)	0.0404	0.0012	0.0335
REGULATION OF MRNA STABILITY BY PROTEINS THAT BIND AU RICH ELEMENTS	78	5 (6.4) [5]	5 (6.4)	0 (0)	0.0388	0.0011	0.0335
HOST INTERACTIONS OF HIV FACTORS	118	6 (5.1) [6]	6 (5.1)	0 (0)	0.0357	0.0011	0.0335
MITOTIC M M G1 PHASES	165	7 (4.2) [7]	7 (4.2)	0 (0)	0.0327	0.0012	0.0335
P53 INDEPENDENT G1 S DNA DAMAGE CHECKPOINT	48	4 (8.3) [4]	4 (8.3)	0 (0)	0.0400	0.0013	0.0339
SCF BETA TRCP MEDIATED DEGRADATION OF EMI1	49	4 (8.2) [4]	4 (8.2)	0 (0)	0.0396	0.0014	0.0339
VIF MEDIATED DEGRADATION OF APOBEC3G	49	4 (8.2) [4]	4 (8.2)	0 (0)	0.0396	0.0014	0.0339
CELL CYCLE	388	11 (2.8) [11]	11 (2.8)	0 (0)	0.0254	0.0015	0.0339
DESTABILIZATION OF MRNA BY AUF1 HNRNP D0	50	4 (8) [4]	4 (8)	0 (0)	0.0392	0.0015	0.0344
P53 DEPENDENT G1 DNA DAMAGE RESPONSE	52	4 (7.7) [4]	4 (7.7)	0 (0)	0.0385	0.0018	0.0385
SCFSKP2 MEDIATED DEGRADATION OF P27 P21	53	4 (7.5) [4]	4 (7.5)	0 (0)	0.0381	0.0019	0.04
CDT1 ASSOCIATION WITH THE CDC6 ORC ORIGIN COMPLEX	54	4 (7.4) [4]	4 (7.4)	0 (0)	0.0377	0.002	0.0404
SYNTHESIS OF DNA	90	5 (5.6) [5]	5 (5.6)	0 (0)	0.0355	0.002	0.0404
AUTODEGRADATION OF CDH1 BY CDH1 APC C	56	4 (7.1) [4]	4 (7.1)	0 (0)	0.0370	0.0023	0.0437
REGULATION OF APOPTOSIS	56	4 (7.1) [4]	4 (7.1)	0 (0)	0.0370	0.0023	0.0437
ER PHAGOSOME PATHWAY	58	4 (6.9) [4]	4 (6.9)	0 (0)	0.0364	0.0027	0.0483
ACTIVATION OF NF KAPPAB IN B CELLS	61	4 (6.6) [4]	4 (6.6)	0 (0)	0.0354	0.0032	0.0566
CYCLIN E ASSOCIATED EVENTS DURING G1 S TRANSITION	62	4 (6.5) [4]	4 (6.5)	0 (0)	0.0351	0.0034	0.0576
APC C CDH1 MEDIATED DEGRADATION OF CDC20 AND OTHER APC C CDH1 TARGETED PROTEINS IN LATE MITOSIS EARLY G1	63	4 (6.3) [4]	4 (6.3)	0 (0)	0.0348	0.0036	0.0576
ASSEMBLY OF THE PRE REPLICATIVE COMPLEX	63	4 (6.3) [4]	4 (6.3)	0 (0)	0.0348	0.0036	0.0576
SIGNALING BY WNT	63	4 (6.3) [4]	4 (6.3)	0 (0)	0.0348	0.0036	0.0576
APC C CDC20 MEDIATED DEGRADATION OF MITOTIC PROTEINS	65	4 (6.2) [4]	4 (6.2)	0 (0)	0.0342	0.004	0.0616
ORC1 REMOVAL FROM CHROMATIN	65	4 (6.2) [4]	4 (6.2)	0 (0)	0.0342	0.004	0.0616
S PHASE	106	5 (4.7) [5]	5 (4.7)	0 (0)	0.0318	0.0041	0.0616
ANTIGEN PROCESSING CROSS PRESENTATION	71	4 (5.6) [4]	4 (5.6)	0 (0)	0.0325	0.0055	0.0808
ADVANCED GLYCOSYLATION ENDPRODUCT RECEPTOR SIGNALING	12	2 (16.7) [2]	2 (16.7)	0 (0)	0.0303	0.0062	0.0891
SIGNALING BY THE B CELL RECEPTOR BCR	119	5 (4.2) [5]	5 (4.2)	0 (0)	0.0294	0.0067	0.0943
REGULATION OF MITOTIC CELL CYCLE	76	4 (5.3) [4]	4 (5.3)	0 (0)	0.0312	0.007	0.0965
EARLY PHASE OF HIV LIFE CYCLE	13	2 (15.4) [2]	2 (15.4)	0 (0)	0.0299	0.0073	0.0983

Category Name	Category Genes #	Sig. Genes #(%) [Protein Fam. #]	Genes Up #(%)	Genes Down #(%)	Jaccard similarity coefficient	P	FDR

Table S13: Enriched MSigDB Reactome Pathways (LC-MS/MS, Day 14). Results are sorted by false discovery rate and Jaccard similarity coefficient.

Category Name	Category Genes #	Sig. Genes #(%) [Protein Fam. #]	Genes Up #(%)	Genes Down #(%)	Jaccard similarity coefficient	P	FDR
GSE2405 0H VS 9H A PHAGOCYTOPHILUM STIM NEUTROPHIL DN	192	20 (10.4) [20]	20 (10.4)	0 (0)	0.0813	<0.0001	<0.0001
GSE2405 0H VS 24H A PHAGOCYTOPHILUM STIM NEUTROPHIL UP	193	17 (8.8) [17]	17 (8.8)	0 (0)	0.0680	<0.0001	<0.0001
GSE42088 UNINF VS LEISHMANIA INF DC 2H DN	188	13 (6.9) [13]	13 (6.9)	0 (0)	0.0522	<0.0001	<0.0001
GSE41978 KLRG1 HIGH VS LOW EFFECTOR CD8 TCELL DN	186	12 (6.5) [12]	12 (6.5)	0 (0)	0.0484	<0.0001	<0.0001
GSE41978 ID2 KO VS ID2 KO AND BIM KO KLRG1 LOW EFFECTOR CD8 TCELL DN	190	12 (6.3) [12]	12 (6.3)	0 (0)	0.0476	<0.0001	<0.0001
GSE22886 NAIVE TCELL VS DC UP	183	11 (6) [12]	11 (6)	0 (0)	0.0447	<0.0001	<0.0001
GSE13485 CTRL VS DAY7 YF17D VACCINE PBMC UP	160	9 (5.6) [10]	9 (5.6)	0 (0)	0.0400	<0.0001	<0.0001
GSE21927 SPLEEN C57BL6 VS 4T1 TUMOR BALBC MONOCYTES UP	183	9 (4.9) [9]	9 (4.9)	0 (0)	0.0363	<0.0001	<0.0001
GSE25123 WT VS PPARG KO MACROPHAGE DN	190	9 (4.7) [9]	9 (4.7)	0 (0)	0.0353	<0.0001	<0.0001
GSE6269 FLU VS E COLI INF PBMC DN	147	8 (5.4) [9]	7 (4.8)	1 (0.7)	0.0376	<0.0001	0.0001
GSE3720 UNSTIM VS LPS STIM VD2 GAMMADELTA TCELL UP	156	8 (5.1) [8]	8 (5.1)	0 (0)	0.0360	<0.0001	0.0002
GSE7509 UNSTIM VS FCGRIIB STIM MONOCYTE UP	159	8 (5) [8]	8 (5)	0 (0)	0.0356	<0.0001	0.0002
GSE3565 DUSP1 VS WT SPLENOCYTES DN	165	8 (4.8) [8]	8 (4.8)	0 (0)	0.0346	<0.0001	0.0002
GSE14000 TRANSLATED RNA VS MRNA DC DN	178	8 (4.5) [9]	8 (4.5)	0 (0)	0.0328	<0.0001	0.0004
GSE42724 NAIVE VS B1 BCELL DN	184	8 (4.3) [8]	8 (4.3)	0 (0)	0.0320	<0.0001	0.0004
GSE22886 DAY0 VS DAY1 MONOCYTE IN CULTURE UP	185	8 (4.3) [8]	8 (4.3)	0 (0)	0.0319	<0.0001	0.0004
GSE42088 UNINF VS LEISHMANIA INF DC 4H DN	188	8 (4.3) [8]	8 (4.3)	0 (0)	0.0315	<0.0001	0.0004
GSE411 100MIN VS 400MIN IL6 STIM MACROPHAGE DN	190	8 (4.2) [8]	8 (4.2)	0 (0)	0.0312	<0.0001	0.0005
GSE26669 CTRL VS COSTIM BLOCK MLR CD8 TCELL UP	194	8 (4.1) [8]	8 (4.1)	0 (0)	0.0308	<0.0001	0.0005
GSE3720 UNSTIM VS PMA STIM VD1 GAMMADELTA TCELL UP	163	7 (4.3) [7]	7 (4.3)	0 (0)	0.0304	<0.0001	0.0017
GSE3720 UNSTIM VS PMA STIM VD2 GAMMADELTA TCELL UP	165	7 (4.2) [7]	7 (4.2)	0 (0)	0.0302	<0.0001	0.0017
GSE21670 UNTREATED VS TGFB IL6 TREATED CD4 TCELL DN	168	7 (4.2) [7]	7 (4.2)	0 (0)	0.0298	<0.0001	0.0019
GSE34205 HEALTHY VS FLU INF INFANT PBMC UP	178	7 (3.9) [7]	7 (3.9)	0 (0)	0.0286	<0.0001	0.0026
GSE29618 PRE VS DAY7 FLU VACCINE PDC DN	183	7 (3.8) [7]	7 (3.8)	0 (0)	0.0280	<0.0001	0.0028
GSE21546 ELK1 KO VS SAP1A KO AND ELK1 KO DP THYMOCYTES DN	184	7 (3.8) [7]	7 (3.8)	0 (0)	0.0279	<0.0001	0.0028
GSE22886 NAIVE BCELL VS NEUTROPHIL UP	184	7 (3.8) [7]	7 (3.8)	0 (0)	0.0279	<0.0001	0.0028
GSE17721 LPS VS PAM3CSK4 6H BMDM DN	186	7 (3.8) [7]	7 (3.8)	0 (0)	0.0277	<0.0001	0.0028
GSE37532 TREG VS TCONV CD4 TCELL FROM LN UP	187	7 (3.7) [7]	7 (3.7)	0 (0)	0.0276	<0.0001	0.0028
GSE17721 0.5H VS 12H GARDIQUIMOD BMDM UP	189	7 (3.7) [7]	7 (3.7)	0 (0)	0.0273	<0.0001	0.0028
GSE360 LOW DOSE B MALAYI VS M TUBERCULOSIS DC UP	189	7 (3.7) [7]	7 (3.7)	0 (0)	0.0273	<0.0001	0.0028
GSE39864 WT VS GATA3 KO TREG DN	189	7 (3.7) [7]	7 (3.7)	0 (0)	0.0273	<0.0001	0.0028
GSE339 CD4POS VS CD8POS DC IN CULTURE UP	194	7 (3.6) [7]	7 (3.6)	0 (0)	0.0268	<0.0001	0.0033
GSE24574 BCL6 HIGH VS LOW TFH CD4 TCELL DN	196	7 (3.6) [7]	7 (3.6)	0 (0)	0.0266	<0.0001	0.0034
GSE6269 HEALTHY VS FLU INF PBMC UP	160	6 (3.8) [6]	6 (3.8)	0 (0)	0.0263	<0.0001	0.0099
GSE34156 NOD2 LIGAND VS NOD2 AND TLR1 TLR2 LIGAND 6H TREATED MONOCYTE UP	172	6 (3.5) [7]	6 (3.5)	0 (0)	0.0250	0.0001	0.0144
GSE2770 IL12 ACT VS ACT CD4 TCELL 6H UP	177	6 (3.4) [6]	6 (3.4)	0 (0)	0.0245	0.0001	0.0159
GSE5099 CLASSICAL M1 VS ALTERNATIVE M2 MACROPHAGE UP	177	6 (3.4) [6]	6 (3.4)	0 (0)	0.0245	0.0001	0.0159
GSE1925 3H VS 24H IFNG STIM MACROPHAGE DN	182	6 (3.3) [6]	6 (3.3)	0 (0)	0.0240	0.0001	0.0181
GSE22886 NAIVE TCELL VS DC DN	186	6 (3.2) [6]	6 (3.2)	0 (0)	0.0236	0.0002	0.0181
GSE28408 LY6G POS VS NEG DC DN	186	6 (3.2) [6]	6 (3.2)	0 (0)	0.0236	0.0002	0.0181
GSE7509 DC VS MONOCYTE UP	186	6 (3.2) [6]	6 (3.2)	0 (0)	0.0236	0.0002	0.0181
GSE17721 CPG VS GARDIQUIMOD 8H BMDM DN	187	6 (3.2) [6]	5 (2.7)	1 (0.5)	0.0235	0.0002	0.0181
GSE22886 CTRL VS LPS 24H DC UP	187	6 (3.2) [6]	6 (3.2)	0 (0)	0.0235	0.0002	0.0181
GSE2770 UNTREATED VS ACT CD4 TCELL 2H DN	187	6 (3.2) [6]	6 (3.2)	0 (0)	0.0235	0.0002	0.0181
GSE42088 2H VS 24H LEISHMANIA INF DC UP	189	6 (3.2) [6]	6 (3.2)	0 (0)	0.0233	0.0002	0.0182

Category Name	Category Genes #	Sig. Genes #(%) [Protein Fam. #]	Genes Up #(%)	Genes Down #(%)	Jaccard similarity coefficient	P	FDR
GSE17721 PAM3CSK4 VS GADIQUIMOD 24H BMDM UP	190	6 (3.2) [6]	6 (3.2)	0 (0)	0.0233	0.0002	0.0182
GSE22886 NEUTROPHIL VS MONOCYTE DN	190	6 (3.2) [6]	6 (3.2)	0 (0)	0.0233	0.0002	0.0182
GSE17721 12H VS 24H GARDIQUIMOD BMDM UP	191	6 (3.1) [6]	6 (3.1)	0 (0)	0.0232	0.0002	0.0182
GSE9988 LOW LPS VS VEHICLE TREATED MONOCYTE DN	191	6 (3.1) [6]	6 (3.1)	0 (0)	0.0232	0.0002	0.0182
GSE22886 NAIVE BCELL VS MONOCYTE DN	192	6 (3.1) [6]	6 (3.1)	0 (0)	0.0231	0.0002	0.0184
GSE17721 POLYIC VS CPG 4H BMDM UP	193	6 (3.1) [6]	6 (3.1)	0 (0)	0.0230	0.0002	0.0185
GSE22601 IMMATURE CD4 SINGLE POSITIVE VS CD4 SINGLE POSITIVE THYMOCYTE UP	195	6 (3.1) [6]	6 (3.1)	0 (0)	0.0228	0.0002	0.0192
GSE22886 NAIVE CD4 TCELL VS 48H ACT TH1 DN	197	6 (3) [6]	6 (3)	0 (0)	0.0226	0.0002	0.0195
GSE9006 TYPE 1 VS TYPE 2 DIABETES PBMC AT DX UP	197	6 (3) [6]	6 (3)	0 (0)	0.0226	0.0002	0.0195
GSE36476 CTRL VS TSST ACT 16H MEMORY CD4 TCELL OLD DN	199	6 (3) [6]	6 (3)	0 (0)	0.0225	0.0002	0.0203
GSE40274 CTRL VS XBP1 TRANSDUCED ACTIVATED CD4 TCELL DN	155	5 (3.2) [5]	5 (3.2)	0 (0)	0.0223	0.0006	0.05
GSE17974 IL4 AND ANTI IL12 VS UNTREATED 0.5H ACT CD4 TCELL UP	158	5 (3.2) [6]	5 (3.2)	0 (0)	0.0220	0.0006	0.0536
GSE3565 CTRL VS LPS INJECTED DUSP1 KO SPLENOCYTES DN	161	5 (3.1) [5]	5 (3.1)	0 (0)	0.0217	0.0007	0.0574
GSE9601 UNTREATED VS PI3K INHIBITOR TREATED HCMV INF MONOCYTE UP	167	5 (3) [5]	5 (3)	0 (0)	0.0212	0.0008	0.0598
GSE25088 CTRL VS IL4 AND ROSIGLITAZONE STIM STAT6 KO MACROPHAGE DN	169	5 (3) [5]	5 (3)	0 (0)	0.0210	0.0008	0.0598
GSE2770 TGFB AND IL4 ACT VS ACT CD4 TCELL 2H UP	178	5 (2.8) [6]	5 (2.8)	0 (0)	0.0202	0.0011	0.0598
GSE11864 CSF1 VS CSF1 IFNG PAM3CYS IN MAC DN	179	5 (2.8) [5]	5 (2.8)	0 (0)	0.0202	0.0011	0.0598
GSE18804 BRAIN VS COLON TUMORAL MACROPHAGE DN	179	5 (2.8) [5]	5 (2.8)	0 (0)	0.0202	0.0011	0.0598
GSE32986 UNSTIM VS CURDLAN HIGHDOSE STIM DC DN	181	5 (2.8) [5]	5 (2.8)	0 (0)	0.0200	0.0012	0.0598
GSE2770 IL12 AND TGFB ACT VS ACT CD4 TCELL 2H UP	182	5 (2.7) [5]	5 (2.7)	0 (0)	0.0199	0.0012	0.0598
GSE13484 12H VS 3H YF17D VACCINE STIM PBMC DN	183	5 (2.7) [5]	5 (2.7)	0 (0)	0.0198	0.0012	0.0598
GSE37532 WT VS PPARG KO VISCERAL ADIPOSE TISSUE TREG UP	183	5 (2.7) [5]	5 (2.7)	0 (0)	0.0198	0.0012	0.0598
GSE3982 NEUTROPHIL VS TH2 DN	183	5 (2.7) [5]	5 (2.7)	0 (0)	0.0198	0.0012	0.0598
GSE3982 EFF MEMORY CD4 TCELL VS NKCELL DN	184	5 (2.7) [6]	5 (2.7)	0 (0)	0.0198	0.0012	0.0598
GSE24814 STAT5 KO VS WT PRE BCELL DN	184	5 (2.7) [5]	5 (2.7)	0 (0)	0.0198	0.0012	0.0598
GSE360 DC VS MAC B MALAYI HIGH DOSE DN	185	5 (2.7) [5]	5 (2.7)	0 (0)	0.0197	0.0013	0.0598
GSE1460 DP THYMOCYTE VS NAIVE CD4 TCELL ADULT BLOOD DN	186	5 (2.7) [6]	5 (2.7)	0 (0)	0.0196	0.0013	0.0598
GSE17721 LPS VS PAM3CSK4 8H BMDM DN	186	5 (2.7) [6]	5 (2.7)	0 (0)	0.0196	0.0013	0.0598
GSE35685 CD34POS CD10NEG CD62LPOS VS CD34POS CD10POS BONE MARROW UP	186	5 (2.7) [6]	5 (2.7)	0 (0)	0.0196	0.0013	0.0598
GSE10239 NAIVE VS DAY4.5 EFF CD8 TCELL UP	186	5 (2.7) [5]	5 (2.7)	0 (0)	0.0196	0.0013	0.0598
GSE10239 NAIVE VS KLRG1HIGH EFF CD8 TCELL UP	186	5 (2.7) [5]	5 (2.7)	0 (0)	0.0196	0.0013	0.0598
GSE22886 NAIVE CD4 TCELL VS 48H ACT TH2 UP	186	5 (2.7) [5]	5 (2.7)	0 (0)	0.0196	0.0013	0.0598
GSE25088 ROSIGLITAZONE VS IL4 AND ROSIGLITAZONE STIM STAT6 KO MACROPHAGE DAY10 DN	186	5 (2.7) [5]	5 (2.7)	0 (0)	0.0196	0.0013	0.0598
GSE360 CTRL VS L MAJOR DC UP	186	5 (2.7) [5]	5 (2.7)	0 (0)	0.0196	0.0013	0.0598
GSE360 L DONOVANI VS B MALAYI LOW DOSE DC DN	186	5 (2.7) [5]	5 (2.7)	0 (0)	0.0196	0.0013	0.0598
GSE3982 EOSINOPHIL VS MAST CELL DN	186	5 (2.7) [5]	5 (2.7)	0 (0)	0.0196	0.0013	0.0598
GSE45837 WT VS GFI1 KO PDC DN	186	5 (2.7) [5]	5 (2.7)	0 (0)	0.0196	0.0013	0.0598
GSE14000 4H VS 16H LPS DC UP	187	5 (2.7) [5]	5 (2.7)	0 (0)	0.0195	0.0013	0.0598
GSE1460 INTRATHYMIC T PROGENITOR VS NAIVE CD4 TCELL ADULT BLOOD UP	187	5 (2.7) [5]	5 (2.7)	0 (0)	0.0195	0.0013	0.0598
GSE14908 RESTING VS HDM STIM CD4 TCELL ATOPIC PATIENT UP	187	5 (2.7) [5]	5 (2.7)	0 (0)	0.0195	0.0013	0.0598
GSE17721 POLYIC VS GARDIQUIMOD 12H BMDM DN	187	5 (2.7) [5]	5 (2.7)	0 (0)	0.0195	0.0013	0.0598
GSE2124 CTRL VS LYMPHOTOXIN BETA TREATED MLN UP	187	5 (2.7) [5]	5 (2.7)	0 (0)	0.0195	0.0013	0.0598
GSE22432 MULTIPOTENT PROGENITOR VS PDC DN	187	5 (2.7) [5]	5 (2.7)	0 (0)	0.0195	0.0013	0.0598
GSE22886 NAIVE CD8 TCELL VS DC DN	187	5 (2.7) [5]	5 (2.7)	0 (0)	0.0195	0.0013	0.0598
GSE2770 UNTREATED VS IL4 TREATED ACT CD4 TCELL 2H UP	187	5 (2.7) [5]	5 (2.7)	0 (0)	0.0195	0.0013	0.0598
GSE3039 NKT CELL VS B1 BCELL DN	187	5 (2.7) [5]	5 (2.7)	0 (0)	0.0195	0.0013	0.0598
GSE3982 MAC VS NEUTROPHIL LPS STIM UP	187	5 (2.7) [5]	5 (2.7)	0 (0)	0.0195	0.0013	0.0598
GSE17721 0.5H VS 24H PAM3CSK4 BMDM UP	188	5 (2.7) [5]	4 (2.1)	1 (0.5)	0.0195	0.0014	0.0598
GSE34205 HEALTHY VS FLU INF INFANT PBMC DN	188	5 (2.7) [5]	5 (2.7)	0 (0)	0.0195	0.0014	0.0598
GSE3982 CENT MEMORY CD4 TCELL VS NKCELL UP	188	5 (2.7) [5]	5 (2.7)	0 (0)	0.0195	0.0014	0.0598
GSE15330 LYMPHOID MULTIPOTENT VS GRANULOCYTE MONOCYTE PROGENITOR IKAROS KO UP	189	5 (2.6) [5]	5 (2.6)	0 (0)	0.0194	0.0014	0.0598
GSE22886 NAIVE CD4 TCELL VS 12H ACT TH1 DN	189	5 (2.6) [5]	5 (2.6)	0 (0)	0.0194	0.0014	0.0598

Category Name	Category Genes #	Sig. Genes #(%) [Protein Fam. #]	Genes Up #(%)	Genes Down #(%)	Jaccard similarity coefficient	P	FDR
GSE22886 NAIVE CD4 TCELL VS MONOCYTE DN	189	5 (2.6) [5]	5 (2.6)	0 (0)	0.0194	0.0014	0.0598
GSE27859 DC VS CD11C INT F480 INT DC UP	189	5 (2.6) [5]	5 (2.6)	0 (0)	0.0194	0.0014	0.0598
GSE35685 CD34POS CD38NEG VS CD34POS CD10NEG CD62LPOS BONE MARROW UP	189	5 (2.6) [5]	5 (2.6)	0 (0)	0.0194	0.0014	0.0598
GSE43955 10H VS 60H ACT CD4 TCELL WITH TGFB IL6 UP	189	5 (2.6) [5]	5 (2.6)	0 (0)	0.0194	0.0014	0.0598
GSE17721 CTRL VS GARDIQUIMOD 12H BMDM UP	190	5 (2.6) [5]	4 (2.1)	1 (0.5)	0.0193	0.0014	0.0598
GSE17721 LPS VS CPG 24H BMDM DN	190	5 (2.6) [5]	5 (2.6)	0 (0)	0.0193	0.0014	0.0598
GSE18893 CTRL VS TNF TREATED TCONV 2H UP	190	5 (2.6) [5]	5 (2.6)	0 (0)	0.0193	0.0014	0.0598
GSE20152 SPHK1 KO VS WT HTNFA OVERXPRESS ANKLE DN	190	5 (2.6) [5]	5 (2.6)	0 (0)	0.0193	0.0014	0.0598
GSE17721 0.5H VS 8H GARDIQUIMOD BMDM UP	191	5 (2.6) [5]	5 (2.6)	0 (0)	0.0192	0.0015	0.0598
GSE21360 SECONDARY VS TERTIARY MEMORY CD8 TCELL UP	191	5 (2.6) [5]	5 (2.6)	0 (0)	0.0192	0.0015	0.0598
GSE29164 CD8 TCELL VS CD8 TCELL AND IL12 TREATED MELANOMA DAY3 UP	191	5 (2.6) [5]	5 (2.6)	0 (0)	0.0192	0.0015	0.0598
GSE29618 BCELL VS MDC DAY7 FLU VACCINE DN	191	5 (2.6) [5]	5 (2.6)	0 (0)	0.0192	0.0015	0.0598
GSE3982 EFF MEMORY CD4 TCELL VS TH2 DN	191	5 (2.6) [5]	5 (2.6)	0 (0)	0.0192	0.0015	0.0598
GSE16450 CTRL VS IFNA 6H STIM MATURE NEURON CELL LINE DN	192	5 (2.6) [5]	5 (2.6)	0 (0)	0.0192	0.0015	0.0598
GSE2405 HEAT KILLED LYSATE VS LIVE A PHAGOCYTOPHILUM STIM NEUTROPHIL 9H UP	192	5 (2.6) [5]	4 (2.1)	1 (0.5)	0.0192	0.0015	0.0598
GSE31082 CD4 VS CD8 SP THYMOCYTE DN	192	5 (2.6) [5]	5 (2.6)	0 (0)	0.0192	0.0015	0.0598
GSE360 CTRL VS L DONOVANI MAC UP	192	5 (2.6) [5]	5 (2.6)	0 (0)	0.0192	0.0015	0.0598
GSE360 DC VS MAC L DONOVANI DN	192	5 (2.6) [5]	5 (2.6)	0 (0)	0.0192	0.0015	0.0598
GSE5099 MONOCYTE VS CLASSICAL M1 MACROPHAGE DN	192	5 (2.6) [5]	5 (2.6)	0 (0)	0.0192	0.0015	0.0598
GSE7764 IL15 TREATED VS CTRL NK CELL 24H UP	192	5 (2.6) [5]	5 (2.6)	0 (0)	0.0192	0.0015	0.0598
GSE12845 IGD NEG BLOOD VS NAIVE TONSIL BCELL UP	193	5 (2.6) [5]	5 (2.6)	0 (0)	0.0191	0.0015	0.0598
GSE13411 NAIVE VS IGM MEMORY BCELL DN	193	5 (2.6) [5]	5 (2.6)	0 (0)	0.0191	0.0015	0.0598
GSE22196 HEALTHY VS OBESE MOUSE SKIN GAMMADELTA TCELL DN	193	5 (2.6) [5]	5 (2.6)	0 (0)	0.0191	0.0015	0.0598
GSE22886 UNSTIM VS STIM MEMORY TCELL DN	193	5 (2.6) [5]	5 (2.6)	0 (0)	0.0191	0.0015	0.0598
GSE26030 TH1 VS TH17 RESTIMULATED DAY5 POST POLARIZATION UP	193	5 (2.6) [5]	5 (2.6)	0 (0)	0.0191	0.0015	0.0598
GSE26669 CTRL VS COSTIM BLOCK MLR CD4 TCELL UP	193	5 (2.6) [5]	5 (2.6)	0 (0)	0.0191	0.0015	0.0598
GSE26727 WT VS KLF2 KO LPS STIM MACROPHAGE DN	193	5 (2.6) [5]	5 (2.6)	0 (0)	0.0191	0.0015	0.0598
GSE42021 TCONV PLN VS CD24INT TCONV THYMUS UP	193	5 (2.6) [5]	5 (2.6)	0 (0)	0.0191	0.0015	0.0598
GSE23114 PERITONEAL CAVITY B1A BCELL VS SPLEEN BCELL DN	194	5 (2.6) [5]	5 (2.6)	0 (0)	0.0190	0.0016	0.0601
GSE17721 CTRL VS LPS 12H BMDM DN	195	5 (2.6) [5]	5 (2.6)	0 (0)	0.0189	0.0016	0.0601
GSE3982 MEMORY CD4 TCELL VS TH2 DN	195	5 (2.6) [5]	5 (2.6)	0 (0)	0.0189	0.0016	0.0601
GSE24634 NAIVE CD4 TCELL VS DAY5 IL4 CONV TREG DN	196	5 (2.6) [5]	5 (2.6)	0 (0)	0.0189	0.0016	0.0601
GSE28726 ACT CD4 TCELL VS ACT NKTCELL UP	196	5 (2.6) [5]	5 (2.6)	0 (0)	0.0189	0.0016	0.0601
GSE41867 NAIVE VS DAY8 LCMV EFFECTOR CD8 TCELL UP	196	5 (2.6) [5]	5 (2.6)	0 (0)	0.0189	0.0016	0.0601
GSE9006 HEALTHY VS TYPE 2 DIABETES PBMC AT DX UP	196	5 (2.6) [5]	5 (2.6)	0 (0)	0.0189	0.0016	0.0601
KAECH NAIVE VS DAY15 EFF CD8 TCELL DN	196	5 (2.6) [5]	5 (2.6)	0 (0)	0.0189	0.0016	0.0601
GSE16266 CTRL VS HEATSHOCK AND LPS STIM MEF UP	197	5 (2.5) [5]	5 (2.5)	0 (0)	0.0188	0.0017	0.061

Table S14: Enriched MSigDB Immunologic Signature Sets (LC-MS/MS, Day 14). Results are sorted by false discovery rate and Jaccard similarity coefficient.

Category Name	Category Genes #	Sig. Genes #(%) [Protein Fam. #]	Genes Up #(%)	Genes Down #(%)	Jaccard similarity coefficient	P	FDR
Pathogenic Escherichia coli infection	54	3 (5.6) [3]	3 (5.6)	0 (0)	0.2273	0.0001	0.0287
Antigen processing and presentation	64	3 (4.7) [2]	3 (4.7)	0 (0)	0.0460	0.0002	0.0287
Estrogen signaling pathway	97	3 (3.1) [2]	3 (3.1)	0 (0)	0.0333	0.0006	0.0653

Table S15: Enriched KEGG Pathways (2D-DIGE/MS, Day 7). Results are sorted by false discovery rate and Jaccard similarity coefficient.

Category Name	Category Genes #	Sig. Genes #(%) [Protein Fam. #]	Genes Up #(%)	Genes Down #(%)	Jaccard similarity coefficient	P	FDR
GSE22886 NAIVE VS IGG IGA MEMORY BCELL DN	186	6 (3.2) [6]	6 (3.2)	0 (0)	0.0429	<0.0001	<0.0001
GSE29618 BCELL VS MDC DN	193	6 (3.1) [6]	6 (3.1)	0 (0)	0.0415	<0.0001	<0.0001
GSE29618 BCELL VS MDC DAY7 FLU VACCINE DN	191	6 (3.1) [6]	6 (3.1)	0 (0)	0.0370	<0.0001	<0.0001
GSE41978 ID2 KO VS ID2 KO AND BIM KO KLRG1 LOW EFFECTOR CD8 TCELL DN	190	5 (2.6) [5]	5 (2.6)	0 (0)	0.0324	<0.0001	0.0006
GSE2405 0H VS 9H A PHAGOCYTOPHILUM STIM NEUTROPHIL DN	192	5 (2.6) [5]	5 (2.6)	0 (0)	0.0321	<0.0001	0.0006
GSE22886 NAIVE CD4 TCELL VS DC DN	190	5 (2.6) [5]	5 (2.6)	0 (0)	0.0276	<0.0001	0.0006
GSE22886 UNSTIM VS IL2 STIM NKCELL DN	192	4 (2.1) [4]	4 (2.1)	0 (0)	0.0369	<0.0001	0.0072
GSE29618 PDC VS MDC DAY7 FLU VACCINE DN	190	4 (2.1) [4]	3 (1.6)	1 (0.5)	0.0324	<0.0001	0.0072
GSE360 LOW DOSE B MALAYI VS M TUBERCULOSIS DC DN	191	4 (2.1) [3]	4 (2.1)	0 (0)	0.0323	<0.0001	0.0072
GSE22886 NAIVE CD8 TCELL VS DC DN	187	4 (2.1) [4]	4 (2.1)	0 (0)	0.0280	<0.0001	0.0072
GSE29949 CD8 NEG DC SPLEEN VS MONOCYTE BONE MARROW UP	189	4 (2.1) [4]	3 (1.6)	1 (0.5)	0.0278	<0.0001	0.0072
GSE36826 NORMAL VS STAPH AUREUS INF IL1R KO SKIN DN	187	4 (2.1) [4]	3 (1.6)	1 (0.5)	0.0233	<0.0001	0.0072
GSE22886 NAIVE BCELL VS MONOCYTE DN	192	4 (2.1) [4]	4 (2.1)	0 (0)	0.0227	<0.0001	0.0072
GSE43955 10H VS 30H ACT CD4 TCELL UP	192	4 (2.1) [4]	4 (2.1)	0 (0)	0.0227	<0.0001	0.0072
GSE28726 ACT CD4 TCELL VS ACT NKTCELL UP	196	4 (2) [4]	4 (2)	0 (0)	0.0223	<0.0001	0.0072
GSE29618 BCELL VS MONOCYTE DN	190	4 (2.1) [4]	4 (2.1)	0 (0)	0.0183	<0.0001	0.0072
GSE36826 WT VS IL1R KO SKIN DN	190	4 (2.1) [4]	3 (1.6)	1 (0.5)	0.0183	<0.0001	0.0072
GSE27786 LSK VS CD8 TCELL DN	193	4 (2.1) [4]	3 (1.6)	1 (0.5)	0.0180	<0.0001	0.0072
GSE26669 CTRL VS COSTIM BLOCK MLR CD8 TCELL UP	194	4 (2.1) [4]	4 (2.1)	0 (0)	0.0179	<0.0001	0.0072
GSE22886 NAIVE VS IGM MEMORY BCELL DN	188	3 (1.6) [3]	3 (1.6)	0 (0)	0.0279	0.0007	0.0543
GSE36476 CTRL VS TSST ACT 72H MEMORY CD4 TCELL OLD DN	192	3 (1.6) [3]	3 (1.6)	0 (0)	0.0274	0.0007	0.0543
GSE29618 PDC VS MDC DN	186	3 (1.6) [3]	3 (1.6)	0 (0)	0.0234	0.0007	0.0543
GSE41978 KLRG1 HIGH VS LOW EFFECTOR CD8 TCELL DN	186	3 (1.6) [3]	3 (1.6)	0 (0)	0.0234	0.0007	0.0543
GSE2405 0H VS 24H A PHAGOCYTOPHILUM STIM NEUTROPHIL UP	193	3 (1.6) [3]	3 (1.6)	0 (0)	0.0226	0.0007	0.0543
GSE36476 CTRL VS TSST ACT 40H MEMORY CD4 TCELL YOUNG DN	193	3 (1.6) [3]	3 (1.6)	0 (0)	0.0226	0.0007	0.0543
GSE22886 UNSTIM VS IL15 STIM NKCELL DN	194	3 (1.5) [3]	3 (1.5)	0 (0)	0.0225	0.0007	0.0543
GSE36476 CTRL VS TSST ACT 16H MEMORY CD4 TCELL YOUNG DN	195	3 (1.5) [3]	3 (1.5)	0 (0)	0.0224	0.0007	0.0543
GSE36476 CTRL VS TSST ACT 72H MEMORY CD4 TCELL YOUNG DN	196	3 (1.5) [3]	3 (1.5)	0 (0)	0.0223	0.0008	0.0543
GSE26488 CTRL VS PEPTIDE INJECTION OT2 THYMOCYTE DN	171	3 (1.8) [3]	2 (1.2)	1 (0.6)	0.0200	0.0005	0.0543
GSE17974 0.5H VS 72H IL4 AND ANTI IL12 ACT CD4 TCELL DN	186	3 (1.6) [3]	3 (1.6)	0 (0)	0.0186	0.0007	0.0543
GSE39022 LN VS SPLEEN DC UP	186	3 (1.6) [3]	3 (1.6)	0 (0)	0.0186	0.0007	0.0543
GSE3982 EOSINOPHIL VS MAST CELL DN	186	3 (1.6) [3]	3 (1.6)	0 (0)	0.0186	0.0007	0.0543
GSE3982 MAC VS CENT MEMORY CD4 TCELL UP	189	3 (1.6) [3]	3 (1.6)	0 (0)	0.0183	0.0007	0.0543
KAECH DAY15 EFF VS MEMORY CD8 TCELL DN	190	3 (1.6) [3]	3 (1.6)	0 (0)	0.0183	0.0007	0.0543
GSE1460 CD4 THYMOCYTE VS NAIVE CD4 TCELL CORD BLOOD UP	191	3 (1.6) [3]	3 (1.6)	0 (0)	0.0182	0.0007	0.0543
GSE1432 1H VS 24H IFNG MICROGLIA UP	192	3 (1.6) [3]	2 (1)	1 (0.5)	0.0181	0.0007	0.0543
GSE26156 DOUBLE POSITIVE VS CD4 SINGLE POSITIVE THYMOCYTE DN	192	3 (1.6) [3]	3 (1.6)	0 (0)	0.0181	0.0007	0.0543
GSE28726 NAIVE CD4 TCELL VS NAIVE VA24NEG NKTCELL UP	192	3 (1.6) [3]	3 (1.6)	0 (0)	0.0181	0.0007	0.0543
GSE3982 EOSINOPHIL VS TH1 DN	192	3 (1.6) [3]	3 (1.6)	0 (0)	0.0181	0.0007	0.0543
GSE13306 RA VS UNTREATED MEM CD4 TCELL DN	193	3 (1.6) [3]	3 (1.6)	0 (0)	0.0180	0.0007	0.0543
GSE339 EX VIVO VS IN CULTURE CD4CD8DN DC DN	193	3 (1.6) [3]	3 (1.6)	0 (0)	0.0180	0.0007	0.0543
GSE42088 UNINF VS LEISHMANIA INF DC 8H UP	193	3 (1.6) [3]	3 (1.6)	0 (0)	0.0180	0.0007	0.0543
GSE7852 LN VS THYMUS TREG DN	193	3 (1.6) [3]	3 (1.6)	0 (0)	0.0180	0.0007	0.0543
GSE23114 PERITONEAL CAVITY B1A BCELL VS SPLEEN BCELL DN	194	3 (1.5) [3]	3 (1.5)	0 (0)	0.0179	0.0007	0.0543
GSE24634 TEFF VS TCONV DAY3 IN CULTURE UP	196	3 (1.5) [3]	3 (1.5)	0 (0)	0.0178	0.0008	0.0543
GSE43955 1H VS 10H ACT CD4 TCELL UP	196	3 (1.5) [3]	3 (1.5)	0 (0)	0.0178	0.0008	0.0543
GSE43955 1H VS 60H ACT CD4 TCELL UP	196	3 (1.5) [3]	3 (1.5)	0 (0)	0.0178	0.0008	0.0543
GSE21033 CTRL VS POLYIC STIM DC 12H UP	148	3 (2) [3]	2 (1.4)	1 (0.7)	0.0169	0.0003	0.0543
GSE11961 MEMORY BCELL DAY7 VS MEMORY BCELL DAY40 UP	182	3 (1.6) [3]	2 (1.1)	1 (0.5)	0.0142	0.0006	0.0543
GSE43863 NAIVE VS TFH CD4 EFF TCELL D6 LCMV UP	182	3 (1.6) [3]	2 (1.1)	1 (0.5)	0.0142	0.0006	0.0543
GSE26030 UNSTIM VS RESTIM TH17 DAY5 POST POLARIZATION DN	184	3 (1.6) [3]	3 (1.6)	0 (0)	0.0140	0.0006	0.0543
GSE3982 EOSINOPHIL VS TH2 DN	185	3 (1.6) [3]	3 (1.6)	0 (0)	0.0140	0.0006	0.0543

Category Name	Category Genes #	Sig. Genes #(%) [Protein Fam. #]	Genes Up #(%)	Genes Down #(%)	Jaccard similarity coefficient	P	FDR
GSE22886 NAIVE TCELL VS DC DN	186	3 (1.6) [3]	3 (1.6)	0 (0)	0.0139	0.0007	0.0543
GSE29617 CTRL VS DAY3 TIV FLU VACCINE PBMC 2008 DN	186	3 (1.6) [3]	3 (1.6)	0 (0)	0.0139	0.0007	0.0543
GSE11864 CSF1 IFNG VS CSF1 PAM3CYS IN MAC UP	189	3 (1.6) [3]	3 (1.6)	0 (0)	0.0137	0.0007	0.0543
GSE11961 MARGINAL ZONE BCELL VS MEMORY BCELL DAY7 UP	189	3 (1.6) [3]	2 (1.1)	1 (0.5)	0.0137	0.0007	0.0543
GSE20715 0H VS 48H OZONE TLR4 KO LUNG DN	190	3 (1.6) [3]	3 (1.6)	0 (0)	0.0136	0.0007	0.0543
GSE21670 IL6 VS TGFB AND IL6 TREATED STAT3 KO CD4 TCELL UP	190	3 (1.6) [3]	2 (1.1)	1 (0.5)	0.0136	0.0007	0.0543
GSE2770 UNTREATED VS IL12 TREATED ACT CD4 TCELL 48H DN	190	3 (1.6) [3]	3 (1.6)	0 (0)	0.0136	0.0007	0.0543
GSE5960 TH1 VS ANERGIC TH1 DN	190	3 (1.6) [3]	3 (1.6)	0 (0)	0.0136	0.0007	0.0543
GSE3982 EFF MEMORY CD4 TCELL VS TH2 DN	191	3 (1.6) [3]	3 (1.6)	0 (0)	0.0136	0.0007	0.0543
GSE18893 TCONV VS TREG 24H TNF STIM UP	193	3 (1.6) [3]	3 (1.6)	0 (0)	0.0135	0.0007	0.0543
GSE3982 CENT MEMORY CD4 TCELL VS TH2 DN	193	3 (1.6) [3]	3 (1.6)	0 (0)	0.0135	0.0007	0.0543
GSE43863 TH1 VS LY6C INT CXCR5POS EFFECTOR CD4 TCELL UP	193	3 (1.6) [3]	3 (1.6)	0 (0)	0.0135	0.0007	0.0543
GSE24142 EARLY THYMIC PROGENITOR VS DN3 THYMOCYTE FETAL UP	194	3 (1.5) [3]	2 (1)	1 (0.5)	0.0134	0.0007	0.0543
GSE25088 CTRL VS ROSIGLITAZONE STIM STAT6 KO MACROPHAGE DN	194	3 (1.5) [3]	3 (1.5)	0 (0)	0.0134	0.0007	0.0543
GSE24142 DN2 VS DN3 THYMOCYTE UP	195	3 (1.5) [3]	2 (1)	1 (0.5)	0.0133	0.0007	0.0543
GSE4748 CTRL VS CYANOBACTERIUM LPSLIKE STIM DC 3H DN	195	3 (1.5) [3]	3 (1.5)	0 (0)	0.0133	0.0007	0.0543
GSE36476 CTRL VS TSST ACT 16H MEMORY CD4 TCELL OLD DN	199	3 (1.5) [3]	3 (1.5)	0 (0)	0.0220	0.0008	0.0559

Table S16: Enriched MSigDB Immunologic Signature Sets (2D-DIGE/MS, Day 7). Results are sorted by false discovery rate and Jaccard similarity coefficient.

Category Name	Category Genes #	Sig. Genes #(%) [Protein Fam. #]	Genes Up #(%)	Genes Down #(%)	Jaccard similarity coefficient	P	FDR
Protein processing in endoplasmic reticulum	159	8 (5) [7]	8 (5)	0 (0)	0.0481	<0.0001	<0.0001
Antigen processing and presentation	64	5 (7.8) [4]	5 (7.8)	0 (0)	0.0745	<0.0001	0.0002
Pathogenic Escherichia coli infection	54	4 (7.4) [4]	4 (7.4)	0 (0)	0.2133	<0.0001	0.0023
Estrogen signaling pathway	97	4 (4.1) [3]	4 (4.1)	0 (0)	0.0388	0.0002	0.0175
Legionellosis	48	3 (6.2) [2]	3 (6.2)	0 (0)	0.0366	0.0005	0.0279
Phagosome	142	4 (2.8) [4]	4 (2.8)	0 (0)	0.0982	0.001	0.0498

Table S17: Enriched KEGG Pathways (2D-DIGE/MS, Day 14). Results are sorted by false discovery rate and Jaccard similarity coefficient.

Category Name	Category Genes #	Sig. Genes #(%) [Protein Fam. #]	Genes Up #(%)	Genes Down #(%)	Jaccard similarity coefficient	P	FDR
ACTIVATION OF CHAPERONE GENES BY ATF6 ALPHA	9	3 (33.3) [3]	3 (33.3)	0 (0)	0.0811	<0.0001	0.0016
ACTIVATION OF CHAPERONES BY ATF6 ALPHA	11	3 (27.3) [3]	3 (27.3)	0 (0)	0.0769	<0.0001	0.0016
RESPONSE TO ELEVATED PLATELET CYTOSOLIC CA2	76	4 (5.3) [4]	4 (5.3)	0 (0)	0.0388	<0.0001	0.0146
UNFOLDED PROTEIN RESPONSE	76	4 (5.3) [4]	4 (5.3)	0 (0)	0.0388	<0.0001	0.0146
PLATELET ACTIVATION SIGNALING AND AGGREGATION	187	5 (2.7) [5]	5 (2.7)	0 (0)	0.0235	0.0002	0.0336
MUSCLE CONTRACTION	44	3 (6.8) [3]	3 (6.8)	0 (0)	0.0417	0.0004	0.0395
DIABETES PATHWAYS	125	4 (3.2) [4]	4 (3.2)	0 (0)	0.0331	0.0006	0.057
GRB2 SOS PROVIDES LINKAGE TO MAPK SIGNALING FOR INTERGRINS	15	2 (13.3) [2]	2 (13.3)	0 (0)	0.0455	0.001	0.0765
P130CAS LINKAGE TO MAPK SIGNALING FOR INTEGRINS	15	2 (13.3) [2]	2 (13.3)	0 (0)	0.0455	0.001	0.0765

Table S18: Enriched MSigDB Reactome Pathways (2D-DIGE/MS, Day 14). Results are sorted by false discovery rate and Jaccard similarity coefficient.

Category Name	Category Genes #	Sig. Genes #(%) [Protein Fam. #]	Genes Up #(%)	Genes Down #(%)	Jaccard similarity coefficient	P	FDR
GSE2405 0H VS 9H A PHAGOCYTOPHILUM STIM NEUTROPHIL DN	192	8 (4.2) [8]	8 (4.2)	0 (0)	0.0485	<0.0001	<0.0001
GSE22886 UNSTIM VS IL2 STIM NKCELL DN	192	7 (3.6) [7]	7 (3.6)	0 (0)	0.0485	<0.0001	<0.0001
GSE29618 BCELL VS MDC DN	193	6 (3.1) [6]	6 (3.1)	0 (0)	0.0391	<0.0001	0.0006
GSE22886 NAIVE VS IGG IGA MEMORY BCELL DN	186	5 (2.7) [5]	5 (2.7)	0 (0)	0.0357	<0.0001	0.0045
GSE360 LOW DOSE B MALAYI VS M TUBERCULOSIS DC DN	191	5 (2.6) [4]	5 (2.6)	0 (0)	0.0349	<0.0001	0.0045
GSE41978 ID2 KO VS ID2 KO AND BIM KO KLRG1 LOW EFFECTOR CD8 TCELL DN	190	5 (2.6) [5]	5 (2.6)	0 (0)	0.0306	<0.0001	0.0045
GSE29618 BCELL VS MDC DAY7 FLU VACCINE DN	191	5 (2.6) [5]	5 (2.6)	0 (0)	0.0304	<0.0001	0.0045
GSE36826 NORMAL VS STAPH AUREUS INF IL1R KO SKIN DN	187	5 (2.7) [5]	5 (2.7)	0 (0)	0.0264	<0.0001	0.0045
GSE22886 NAIVE CD4 TCELL VS DC DN	190	5 (2.6) [5]	5 (2.6)	0 (0)	0.0261	<0.0001	0.0045
GSE1460 CD4 THYMOCYTE VS NAIVE CD4 TCELL CORD BLOOD UP	191	5 (2.6) [5]	5 (2.6)	0 (0)	0.0260	<0.0001	0.0045
GSE43260 BTLA POS VS NEG INTRATUMORAL CD8 TCELL UP	195	5 (2.6) [5]	5 (2.6)	0 (0)	0.0255	<0.0001	0.0045
GSE41176 WT VS TAK1 KO ANTI IGM STIM BCELL 24H UP	186	4 (2.2) [4]	4 (2.2)	0 (0)	0.0404	0.0002	0.029
GSE22140 HEALTHY VS ARTHRITIC GERMFREE MOUSE CD4 TCELL DN	189	4 (2.1) [4]	4 (2.1)	0 (0)	0.0307	0.0002	0.029
GSE2405 0H VS 24H A PHAGOCYTOPHILUM STIM NEUTROPHIL UP	193	4 (2.1) [4]	4 (2.1)	0 (0)	0.0302	0.0002	0.029
GSE12845 IGD POS VS NEG BLOOD BCELL DN	178	4 (2.2) [4]	4 (2.2)	0 (0)	0.0275	0.0001	0.029
GSE29618 PDC VS MDC DN	186	4 (2.2) [4]	4 (2.2)	0 (0)	0.0265	0.0002	0.029
GSE22886 NAIVE CD8 TCELL VS DC DN	187	4 (2.1) [4]	4 (2.1)	0 (0)	0.0264	0.0002	0.029
GSE25087 FETAL VS ADULT TREG UP	187	4 (2.1) [4]	4 (2.1)	0 (0)	0.0264	0.0002	0.029
GSE29949 CD8 NEG DC SPLEEN VS MONOCYTE BONE MARROW UP	189	4 (2.1) [4]	4 (2.1)	0 (0)	0.0262	0.0002	0.029
GSE22886 UNSTIM VS IL15 STIM NKCELL DN	194	4 (2.1) [4]	4 (2.1)	0 (0)	0.0256	0.0002	0.029
GSE37532 TREG VS TCONV PPARG KO CD4 TCELL FROM LN DN	184	4 (2.2) [4]	4 (2.2)	0 (0)	0.0222	0.0002	0.029
GSE24634 NAIVE CD4 TCELL VS DAY10 IL4 CONV TREG DN	191	4 (2.1) [4]	4 (2.1)	0 (0)	0.0216	0.0002	0.029
GSE9006 TYPE 1 DIABETES AT DX VS 1MONTH POST DX PBMC UP	191	4 (2.1) [4]	4 (2.1)	0 (0)	0.0216	0.0002	0.029
GSE26156 DOUBLE POSITIVE VS CD4 SINGLE POSITIVE THYMOCYTE DN	192	4 (2.1) [4]	4 (2.1)	0 (0)	0.0215	0.0002	0.029
GSE3982 EOSINOPHIL VS TH1 DN	192	4 (2.1) [4]	4 (2.1)	0 (0)	0.0215	0.0002	0.029
GSE43955 10H VS 30H ACT CD4 TCELL UP	192	4 (2.1) [4]	4 (2.1)	0 (0)	0.0215	0.0002	0.029
GSE23114 PERITONEAL CAVITY B1A BCELL VS SPLEEN BCELL DN	194	4 (2.1) [4]	4 (2.1)	0 (0)	0.0213	0.0002	0.029
GSE9006 HEALTHY VS TYPE 1 DIABETES PBMC 1MONTH POST DX UP	194	4 (2.1) [4]	4 (2.1)	0 (0)	0.0213	0.0002	0.029
GSE34156 UNTREATED VS 24H NOD2 AND TLR1 TLR2 LIGAND TREATED MONOCYTE UP	167	4 (2.4) [4]	4 (2.4)	0 (0)	0.0191	0.0001	0.029
GSE34156 TLR1 TLR2 LIGAND VS NOD2 AND TLR1 TLR2 LIGAND 24H TREATED MONOCYTE UP	181	4 (2.2) [4]	4 (2.2)	0 (0)	0.0179	0.0002	0.029
GSE43863 NAIVE VS TFH CD4 EFF TCELL D6 LCMV UP	182	4 (2.2) [4]	4 (2.2)	0 (0)	0.0179	0.0002	0.029
GSE37301 HEMATOPOIETIC STEM CELL VS COMMON LYMPHOID PROGENITOR UP	187	4 (2.1) [4]	4 (2.1)	0 (0)	0.0175	0.0002	0.029
GSE20715 0H VS 48H OZONE TLR4 KO LUNG DN	190	4 (2.1) [4]	4 (2.1)	0 (0)	0.0172	0.0002	0.029
GSE36826 WT VS IL1R KO SKIN DN	190	4 (2.1) [4]	4 (2.1)	0 (0)	0.0172	0.0002	0.029

Table S19: Enriched MSigDB Immunologic Signature Sets (2D-DIGE/MS, Day 14). Results are sorted by false discovery rate and Jaccard similarity coefficient.

Category Name	Category Genes #	LC-MS/MS			2D-DIGE/MS		
		Sig. Genes #(%) [Protein Fam. #]	Jaccard similarity coefficient	FDR	Sig. Genes #(%) [Protein Fam. #]	Jaccard similarity coefficient	FDR
GSE41978 KLRG1 HIGH VS LOW EFFECTOR CD8 TCELL DN	186	5 (2.7) [5]	0.0244	0.0101	3 (1.6) [3]	0.0234	0.0543
GSE41978 ID2 KO VS ID2 KO AND BIM KO KLRG1 LOW EFFECTOR CD8 TCELL DN	190	5 (2.6) [5]	0.0239	0.0101	5 (2.6) [5]	0.0324	0.0006
GSE2405 0H VS 9H A PHAGOCYTOPHILUM STIM NEUTROPHIL DN	192	5 (2.6) [5]	0.0237	0.0101	5 (2.6) [5]	0.0321	0.0006
GSE3982 EOSINOPHIL VS MAST CELL DN	186	4 (2.2) [4]	0.0194	0.0999	3 (1.6) [3]	0.0186	0.0543
GSE2405 0H VS 24H A PHAGOCYTOPHILUM STIM NEUTROPHIL UP	193	4 (2.1) [4]	0.0188	0.0999	3 (1.6) [3]	0.0226	0.0543

Table S20: Overlapping enriched MSigDB Immunologic Signature Sets (LC-MS/MS and 2D-DIGE/MS, Day 7).

Category Name	Category Genes #	LC-MS/MS			2D-DIGE/MS		
		Sig. Genes #(%) [Protein Fam. #]	Jaccard similarity coefficient	FDR	Sig. Genes #(%) [Protein Fam. #]	Jaccard similarity coefficient	FDR
GSE2405 0H VS 9H A PHAGOCYTOPHILUM STIM NEUTROPHIL DN	192	20 (10.4) [20]	0.0813	<0.0001	8 (4.2) [8]	0.0485	<0.0001
GSE2405 0H VS 24H A PHAGOCYTOPHILUM STIM NEUTROPHIL UP	193	17 (8.8) [17]	0.0680	<0.0001	4 (2.1) [4]	0.0302	0.029
GSE41978 ID2 KO VS ID2 KO AND BIM KO KLRG1 LOW EFFECTOR CD8 TCELL DN	190	12 (6.3) [12]	0.0476	<0.0001	5 (2.6) [5]	0.0306	0.0045
GSE22886 NAIVE CD8 TCELL VS DC DN	187	5 (2.7) [5]	0.0195	0.0598	4 (2.1) [4]	0.0264	0.029
GSE29618 BCELL VS MDC DAY7 FLU VACCINE DN	191	5 (2.6) [5]	0.0192	0.0598	5 (2.6) [5]	0.0304	0.0045
GSE23114 PERITONEAL CAVITY B1A BCELL VS SPLEEN BCELL DN	194	5 (2.6) [5]	0.0190	0.0601	4 (2.1) [4]	0.0213	0.029

Table S21: Overlapping enriched MSigDB Immunologic Signature Sets (LC-MS/MS and 2D-DIGE/MS, Day 14).

LC-MS/MS	Global Patterns		Spike-in Variation			
Normalization Method	Median (MAD)	Inter Quartile Range (MAD)	Spike-In LGB (MAD)	Spike-In MB (MAD)	Spike-In RNASE1 (MAD)	Mean Spike-in (MAD)
Not Normalized	0.1981	0.2062	0.7014	0.3732	0.3528	0.4758
Median Normalized	0.0000	0.2062	0.6300	0.5189	0.3353	0.4947

Table S22: Impact of different normalization procedures on distributions and spike-in metrics (LC-MS/MS)

2D-DIGE/MS	Global Patterns		Spike-in Variation			
Normalization Method	Median (MAD)	Inter Quartile Range (MAD)	Spike-In LGB (MAD)	Spike-In MB (MAD)	Spike-In RNASE1 (MAD)	Mean Spike-in (MAD)
Not Normalized	0.0470	0.4909	0.5795	0.4890		0.5343
Median Normalized	0.0000	0.4910	0.5613	0.4859		0.5236
LOESS Normalized	0.0476	0.0887	0.5800	0.3478		0.4639
LOESS and Median Normalized	0.0000	0.0887	0.5451	0.4373		0.4912

Table S23: Impact of different normalization procedures on distributions and spike-in metrics (2D-DIGE/MS)

Gel Spot ID	Protein ID	Gene Name	Protein Description	50% Protein Cluster ID (Gene Name)	LC-MS/MS Time Point	2D-DIGE/MS Time Point	LC-MS/MS Log_2 Fold Change	2D-DIGE/MS Log_2 Fold Change
S0588	P33176	KIF5B	Kinesin-1 heavy chain	Q12840 (KIF5A)	Day 7	Day 7	1.110	0.330
S0588	P33176	KIF5B	Kinesin-1 heavy chain	Q12840 (KIF5A)	Day 7	Day 14	1.110	0.347
S0452	P07900	HSP90AA1	Heat shock protein HSP 90-alpha	P07900 (HSP90AA1)	Day 14	Day 7	1.126	0.997
S0917	P60174	TPI1	Triosephosphate isomerase	P60174 (TPI1)	Day 14	Day 7	1.117	0.580
S0452	P07900	HSP90AA1	Heat shock protein HSP 90-alpha	P07900 (HSP90AA1)	Day 14	Day 14	1.126	1.026
S0790	P52907	CAPZA1	F-actin-capping protein subunit alpha-1	P52907 (CAPZA1)	Day 14	Day 14	0.555	0.584

Table S24: Overlapping differentially abundant proteins (LC-MS/MS and 2D-DIGE/MS). Protein annotations are based on UniProt anotations (March 16, 2016)

Package Name	Version	Package Name	Version	Package Name	Version
nlme	3.1-125	Matrix	1.2-4	GenomeInfoDb	1.6.3
bitops	1.0-6	munsell	0.4.3	IRanges	2.4.8
pbkrtest	0.4-6	stringi	1.1.1	S4Vectors	0.8.11
RColorBrewer	1.1-2	QuasiSeq	1.0-8	erccdashboard	1.4.0
tools	3.2.5	zlibbioc	1.16.0	gridExtra	2.2.1
affyio	1.40.0	qvalue	2.2.2	ggplot2	2.1.0
rpart	4.1-10	grid	3.2.5	gtools	3.5.0
KernSmooth	2.23-15	gdata	2.17.0	pvclust	2.0-0
Hmisc	3.17-4	Biostrings	2.38.4	edgeR	3.12.1
mgcv	1.8-12	splines	3.2.5	stringr	1.0.0
colorspace	1.2-6	annotate	1.48.0	affy	1.48.0
ade4	1.7-4	GenomicFeatures	1.22.13	Biobase	2.30.0
nnet	7.3-12	locfit	1.5-9.1	BiocGenerics	0.16.1
preprocessCore	1.32.0	geneplotter	1.48.0	goseq	1.22.0
chron	2.3-47	reshape2	1.4.1	geneLenDataBase	1.6.0
quantreg	5.26	codetools	0.2-14	BiasedUrn	1.07
formatR	1.4	futile.options	1.0.0	impute	1.44.0
SparseM	1.7	XML	3.98-1.4	seqinr	3.3-3
rtracklayer	1.30.4	evaluate	0.9	biomaRt	2.26.1
caTools	1.17.1	latticeExtra	0.6-28	R.utils	2.3.0
scales	0.4.0	data.table	1.9.6	R.oo	1.20.0
genefilter	1.52.1	lambda.r	1.1.9	R.methodsS3	1.7.1
quadprog	1.5-5	nloptr	1.0.4	vegan	2.4-0
digest	0.6.9	MatrixModels	0.4-1	lattice	0.20-33
Rsamtools	1.22.0	gtable	0.2.0	permute	0.9-0
foreign	0.8-66	pracma	1.9.5	plyr	1.8.4
minqa	1.2.4	survival	2.38-3	limma	3.26.9
XVector	0.10.0	GenomicAlignments	1.6.3	MASS	7.3-45
lme4	1.1-12	AnnotationDbi	1.32.3	gplots	3.0.1
BiocInstaller	1.20.3	cluster	2.0.3	car	2.1-2
BiocParallel	1.4.3	ROCR	1.0-7	sqldf	0.4-10
acepack	1.3-3.3	PROPER	1.2.0	RSQLite	1.0.0
RCurl	1.95-4.8	DESeq2	1.10.1	DBI	0.4-1
magrittr	1.5	RcppArmadillo	0.7.400.2.0	gsubfn	0.6-6
Formula	1.2-1	Rcpp	0.12.6	proto	0.3-10
GO.db	3.2.2	SummarizedExperiment	1.0.2	xtable	1.8-2
futile.logger	1.4.3	GenomicRanges	1.22.4	knitr	1.13

Table S25: List of R packages and versions used for the analyses presented in this report. R version 3.2.5 (2016-04-14) 'Very, Very Secure Dishes' run on Ubuntu (release 13.04, x86-64-pc-linux-gnu (64-bit) platform).
